# A sterically encumbered photoredox catalyst enables the unified synthesis of the classical lignan family of natural products†
†Electronic supplementary information (ESI) available. CCDC 1920090. For ESI and crystallographic data in CIF or other electronic format see DOI: 10.1039/c9sc02682g


**DOI:** 10.1039/c9sc02682g

**Published:** 2019-07-05

**Authors:** Edwin Alfonzo, Aaron B. Beeler

**Affiliations:** a Department of Chemistry , Boston University , Boston , Massachusetts 02215 , USA . Email: beelera@bu.edu

## Abstract

Herein, we detail a unified synthetic approach to the classical lignan family of natural products that hinges on divergence from a common intermediate that was strategically identified from nature's biosynthetic blueprints. Efforts toward accessing the common intermediate through a convergent and modular approach resulted in the discovery of a sterically encumbered photoredox catalyst that can selectively generate carbonyl ylides from electron-rich epoxides. These can undergo concerted [3 + 2] dipolar cycloadditions to afford tetrahydrofurans, which were advanced (2–4 steps) to at least one representative natural product or natural product scaffold within all six subtypes in classical lignans. The application of those synthetic blueprints to the synthesis of heterolignans bearing unnatural functionality was demonstrated, which establishes the potential of this strategy to accelerate structure–activity-relationship studies of these natural product frameworks and their rich biological activity.

## Abstract 2

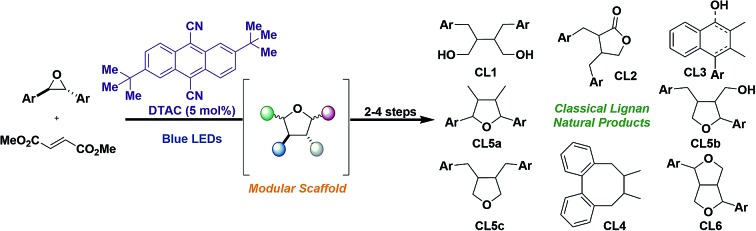
Herein, we detail a unified synthetic approach to the classical lignan family of natural products that hinges on divergence from a common intermediate that was strategically identified from nature's biosynthetic blueprints.

## Introduction

**Scheme 1:**
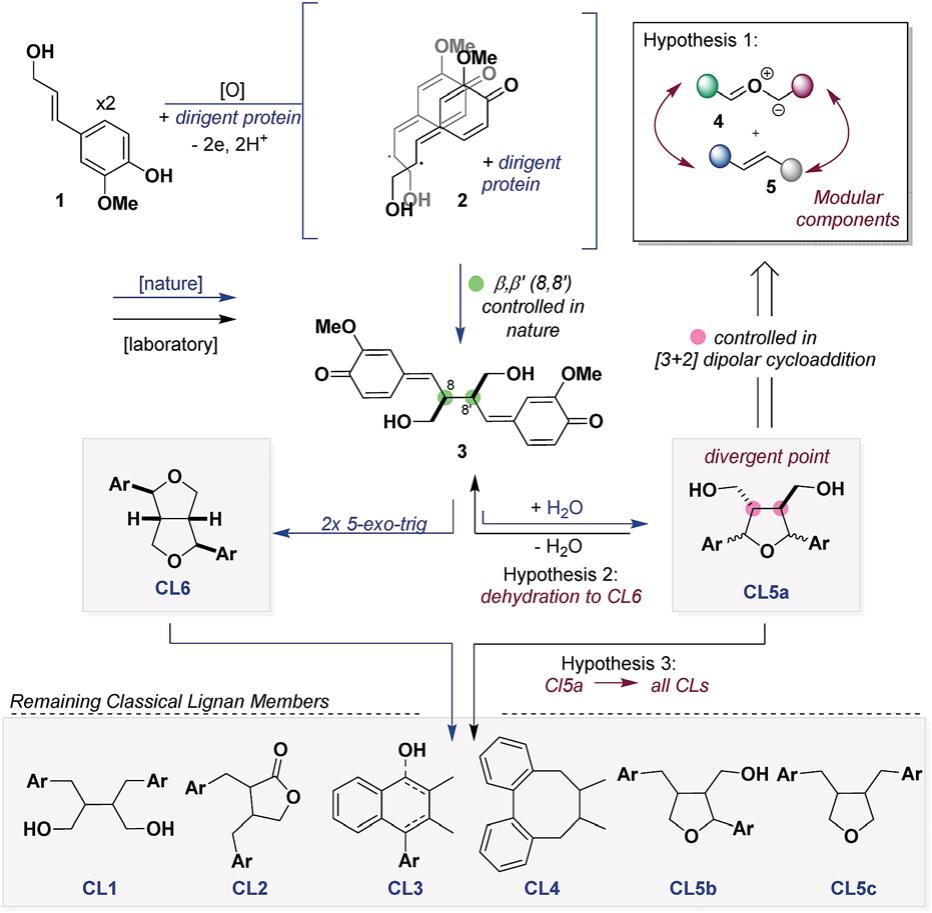
Biosynthesis of the classical lignan family of natural products and proposed strategy to unify them in a laboratory setting.

Classical lignans (CLs) represent one of the oldest known and most sought after families of secondary metabolites found *in planta*.^1^ The interest in these molecules is well merited, as they possess a broad spectrum of promising biological actions, some of which have already significantly impacted society.^2^ Although their carbon frameworks are only composed of two phenylpropane units like **1** (Scheme 1), CLs exhibit wide structural diversity and oxidation patterns that extend to six different subtypes, namely, dibenzylbutane (**CL1**), dibenzylbutyrolactone (**CL2**), arylnaphthalene and its derivatives (**CL3**), dibenzocyclooctadiene (**CL4**), furan (**CL5a–c**), and furofuran (**CL6**). While these subtypes differ greatly in connectivity, they all share a common carbon–carbon β–β′ (8,8′) bond in their propane side chain, a birthmark that is the consequence of a common biosynthesis.

We became interested in developing a convergent and modular approach to access all CL subtypes that could be susceptible to structure–activity-relationship (SAR) studies. Although elegant biosynthetic and total synthetic routes have been established towards the synthesis of CLs, rarely have these strategies shown the flexibility that nature displays in its biosynthesis, resulting in routes that can only access a single or few subtypes and in some instances are not practical for intensive SAR investigation.^3^–^6^ Herein, we report a unified synthetic approach to CLs and demonstrate how the outlined synthetic blueprints are amenable to the synthesis of heterolignans, which can accelerate the investigation of promising biological functions that each subtype displays outside its natural framework.

## Results and discussion

Our retrosynthetic analysis was greatly influenced by a bifurcation that occurs early in the biosynthesis of CLs. One electron oxidation and deprotonation of two units of **1** by an oxidative enzyme produce phenoxy radicals that are trapped by a dirigent protein forming a ternary complex **2**.4*a* Within this complex a regio- and stereoselective carbon–carbon (8,8′) bond coupling event ensues, affording bis *para*-quinone-methide (*p*QM) **3** that is known to bifurcate into two CL subtypes. Two sequential 5-*exo*-trig cyclization steps of **3** furnish the furofuran (**CL6**), which has been shown in most cases to serve as the parent molecule for the remaining CLs.^7^ Alternatively, **3** can be trapped by an equivalence of water to deliver the furan (**CL5a**).^8^

We hypothesized that **CL5a** could be accessed through a concerted [3 + 2] dipolar cycloaddition from the linear carbonyl ylide **4**, derived from epoxides, and a dipolarophile **5** (Scheme 1). A strategic aspect of this reaction is that retention of the stereochemistry of the dipolarophile recapitulates the stereoselective carbon–carbon bond coupling event that occurs in the biosynthesis of CLs. We further posit that we could leverage **CL5a** into **CL6** through the intermediacy of a bis-*p*QM *via* dehydration. Lastly, although **CL6** and **CL5a** differ greatly in connectivity, they possess identical oxidation states. Thus, we thought that if nature could leverage **CL6** into other CLs then we could find selective transformations to do the same with **CL5a**. With this outlined strategy, CLs can be segmented into their peripheral components (aryl groups and pendant oxygen), which would enable a modular approach to synthesize these molecules and facilitate SAR studies.

**Scheme 2:**
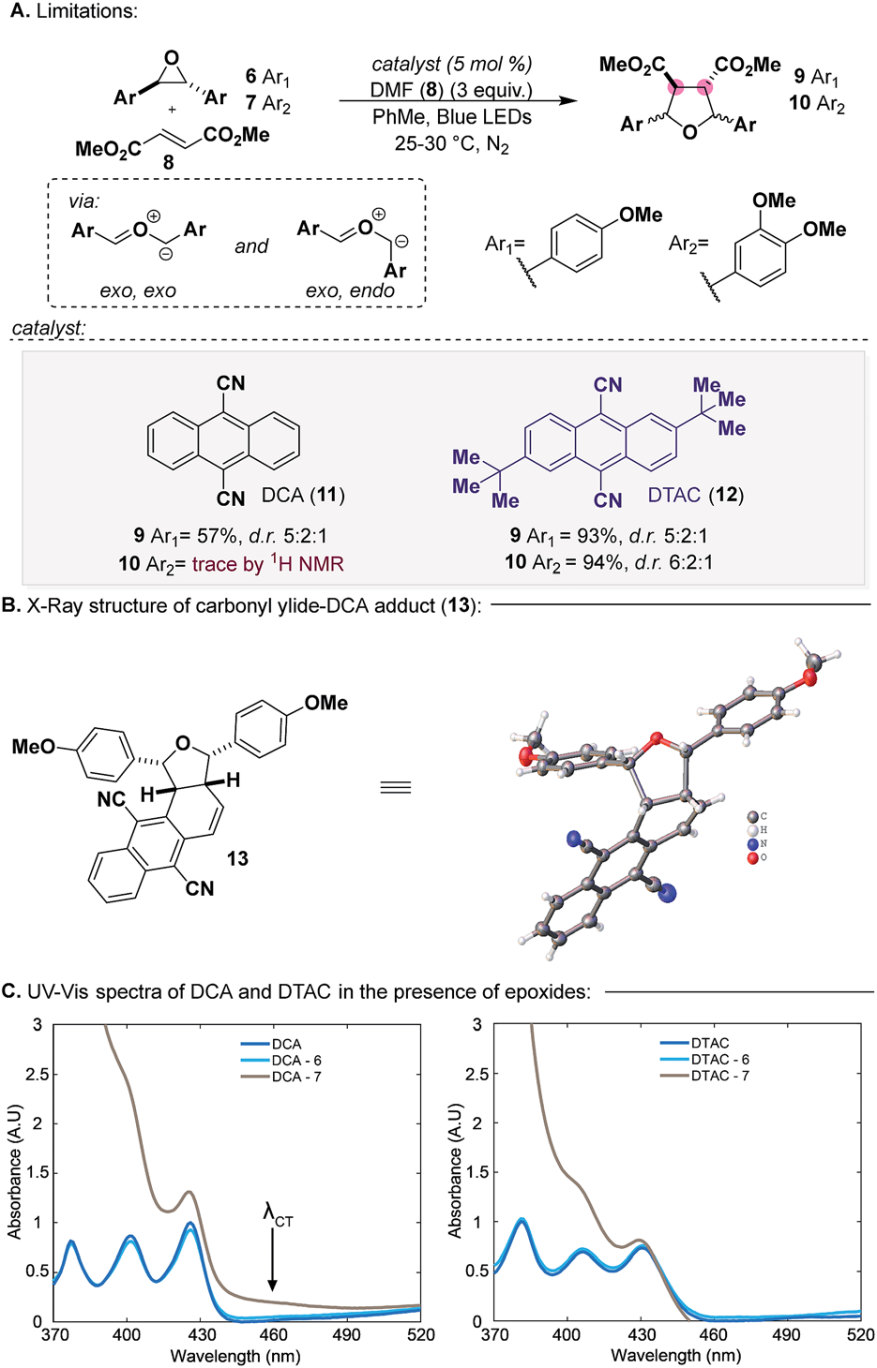
Generation of electron-rich carbonyl ylides.

To this end, seminal work by Whiting established that 9,10-dicyanoanthracene (DCA, **11**) (*λ*_max_ = 422 nm, [DCA*/DCA˙^–^] = +1.99 V *vs.* SCE) (Scheme 2), a photoredox catalyst, can catalyze the formation of HOMO carbonyl ylides from epoxides, which can be trapped with LUMO dipolarophiles.^9^ An important feature of this reaction is that although carbonyl ylide formation proceeds through two distinct conformers (*exo*,*exo* and *exo*,*endo*), the resulting diastereomeric products retain the relative stereochemistry of the dipolarophile. In the context of CLs, we decided to enter **CL5a** with this reaction since conversion to **CL6** would proceed through a stereoablative process, which would provide a single bis-*p*QM, like **3**.

In 2017, we reported our efforts on exploring the scope of carbonyl ylide formation and its [3 + 2] dipolar cycloaddition.^10^ We found some success using DCA, but when expanding the scope to more electron-rich epoxides bearing multiple aryl methoxy groups, characteristic of CL natural products, the reaction failed.^11^ For example, whereas the carbonyl ylide from epoxide **6** (Scheme 2a) could be trapped with modest efficiency with dimethyl fumarate (**8**), epoxide **7** would remain unreacted, only providing trace amounts of the desired product **10**. We have since been able to identify 2,6-di-*tert*-butylanthracene-9,10-dicarbonitrile (DTAC, **12**) (*λ*_max_ = 431 nm, [DTAC*/DTAC˙^–^] = +1.81 V *vs.* SCE), a heretofore unknown catalyst in photoredox methodologies, which can selectively generate carbonyl ylides from electron-rich epoxides.^12^,^13^ When using DTAC as the catalyst for the cycloaddition of epoxide **6** or **7** with dipolarophile **8** the reaction proceeded in nearly quantitative yields (93–94%) with modest selectivity in both cases for the *exo*,*exo* conformer. Notably, after every reaction was performed the catalyst was recovered unscathed and recycled for future use.

Evidence for the superior efficacy of DTAC to DCA was in two forms. First, isolation of adduct **13** under catalytic conditions (Scheme 2b) established the capacity of DCA to participate in the reaction as a dipolarophile, alluding to less undesired reactivity in this reaction setting. Secondly, in the case of epoxide **7**, where only a trace amount of product was detected, we observed a distinct colour formation when it was mixed with DCA which is representative of CT complex formation.^14^ Subsequent UV-Vis spectroscopy studies of DCA in the presence of epoxide **6** or **7** verified the presence of a CT band (*λ*_CT_) in the case of epoxide **7** but not **6** (Scheme 2c, left). When performing an identical UV-Vis experiment with DTAC (Scheme 2c, right), CT band formation was not observed with epoxide **7**. We speculate that in the event where a CT complex is formed, inner-sphere SET and sequential back electron transfer preclude cage escape and carbonyl ylide formation, a known unproductive pathway of catalyst **11**.15*a* Thus, DTAC functions by sterically inhibiting CT complex formation and primarily engaging epoxides through an outer-sphere SET, leading to efficient cage escape and, consequently, carbonyl ylide formation.15*b*

### Synthesis of furofuran (**CL6**)

We began our synthetic efforts towards validating our hypothesis for the dehydration of **CL5a** by targeting methyl piperitol (**19**) (Table 1).^16^ Leveraging previous biomimetic strategies that have relied on bis-*p*QM formation to access **CL6**, it was reasoned that if we could generate the bis-*p*QM from **18** then the cyclization that would follow would prefer to proceed through the conformer where the quinone methide is oriented away from the first formed tetrahydrofuran (**18d***vs.***18c**) to afford **19**.^17^

**Table 1:** Total synthesis of methyl piperitol (**19**) and screened conditions for formation of bis-*para*-quinone methide^*a*^

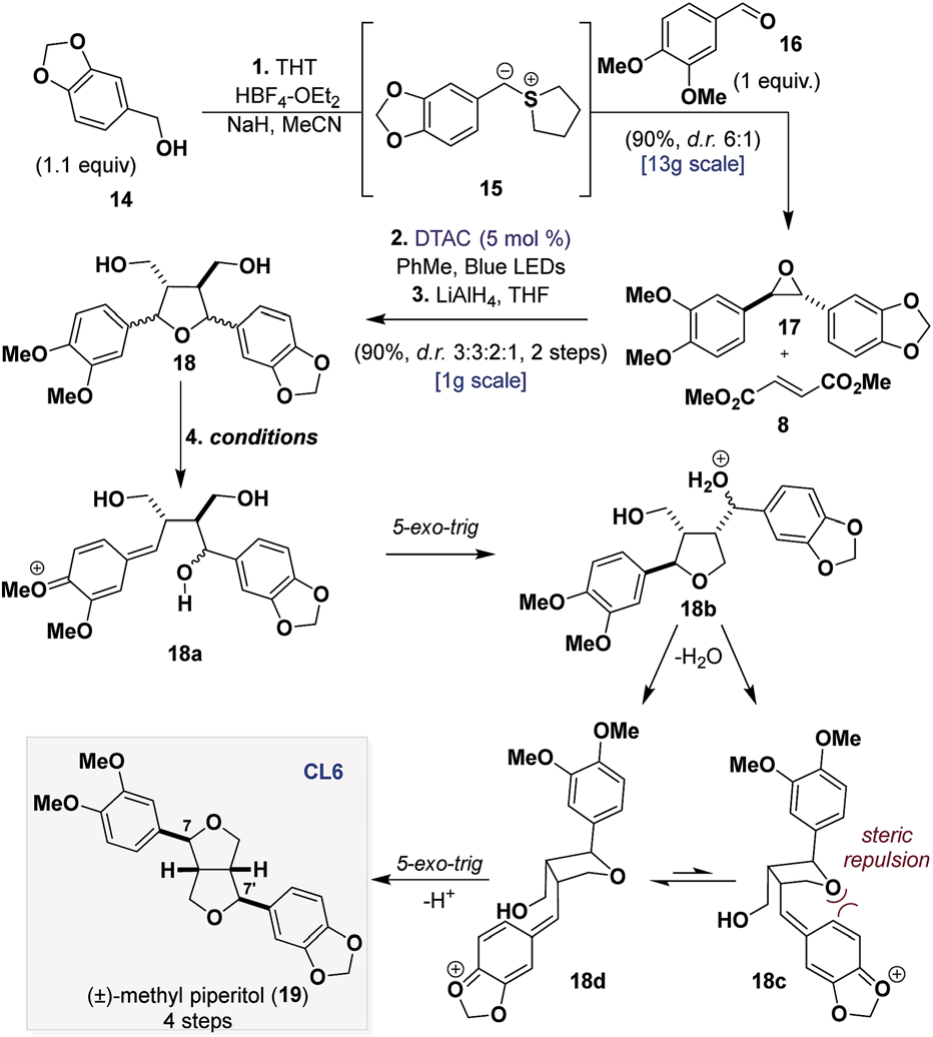			
Entry	Conditions^*a*^	d.r. (**19**, C7,C7′)	% Conversion^*b*^ (IY, RSM)
1	FeCl_3_ (20 mol%), DCM, 12 h	45 : 29 : 26	100
2	HCl (10 mol%), DCM/HFIP, 12 h	59 : 21 : 19	100
3	TMSCl (5 equiv.), DCM, 72 h	67 : 28 : 1	72
4^*c*^	2 M HCl (3 equiv.), 48 h (cycle 1)	93 : 7 : 0	40 (40,58)
5^*c*^ ^,^^*d*^	2 M HCl (3 equiv.), 48 h (cycle 2)	97 : 3 : 0	33 (30,60)

^*a*^23 °C.

^*b*^Isolated yield (IY) and recovered starting material (RSM).

^*c*^2 M HCl in Et_2_O (3 equiv.), CHCl_3_.

^*d*^RSM from entry 4 was used.

Epoxide **17** was synthetized on a decagram scale (95%, d.r. 6 : 1), using a one-pot sulfonium-ylide (**15**) mediated epoxidation of aldehyde **16** with alcohol **14** (Table 1).^18^ Sequentially, **17** was subjected to the [3 + 2] dipolar cycloaddition with **8**, and the resulting product was reduced with LiAlH_4_, providing diol **18** in 90% yield as a mixture of four inconsequential diastereomers (d.r. 3 : 3 : 2 : 1), which served as the substrate for the investigation of the proposed bis-*p*QM formation.

Early success was found when performing the reaction with catalytic amounts of FeCl_3_ (Table 1, entry 1), but unfortunately the poor selectivity, providing the desired product and two of its epimers at C7 and C7′ (which themselves are natural products), led us to consider Brønsted acids over Lewis acids. Under catalytic HCl (10 mol%) in a binary solution of DCM and hexafluoroisopropyl alcohol (HFIP), we observed full conversion of diol **18** with a minor improvement in the selectivity (Table 1, entry 2). A more thorough screening of Brønsted acids led to the discovery that the presence of water significantly slowed the reaction but efforts to remove water with drying agents were futile, as they inhibited reactivity.^19^ We found that generating HCl *in situ* with TMSCl provided higher conversions but only with a modest improvement in selectivity (Table 1, entry 3). Eventually, we found that the use of 2 M HCl in diethyl ether could reconcile the yield and selectivity providing **19** in 40% yield and excellent selectivity (d.r. > 10 : 1) after 48 h (Table 1, entry 4). More importantly, the remaining mass balance of the reaction (58% recovered diol) could be resubmitted to the same conditions and similar efficiency for formation of **19** was observed (Table 1, entry 5). Interestingly, resubmission of **19** to the same conditions resulted in the formation of C7 and C7′ epimers, which demonstrates that while the cyclization may be selective, prolonged exposure to the acidic media leads to epimerization.^19^ Ultimately, our synthetic approach provides (±)-methyl piperitol (**19**) in 4 steps and 32% overall yield.

### Synthesis of dibenzylbutanes (**CL1**)

**Scheme 3:**
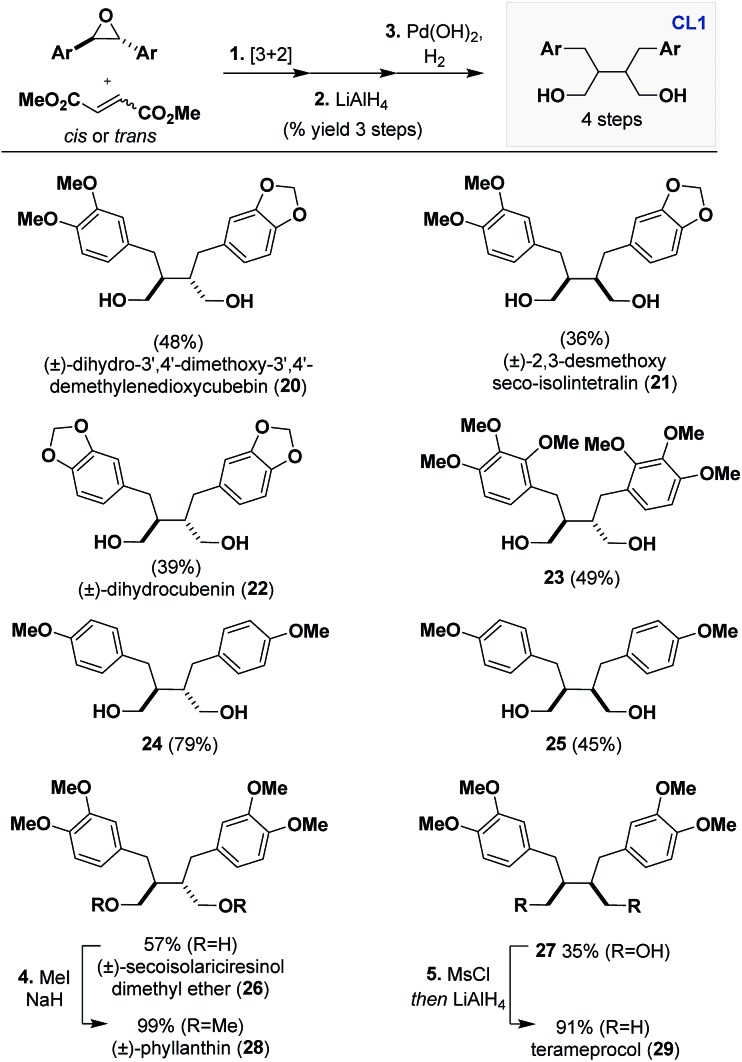
Synthesis of dibenzylbutanes (**CL1**) through hydrogenation of **CL5a**. Reagents and conditions: (1) DTAC (5 mol%), epoxide (1 equiv.), dipolarophile (3 equiv.), PhMe, Blue LEDs, 25–30 °C, N_2_, 1–10 days. (2) LiAlH_4_ (6 equiv.), THF, 0 to 23 °C, 1 h. (3) Pd(OH)_2_ (10–20 mol%), EtOH or 1 : 1 EtOH/EtOAc, H_2_ (balloon), 1–7 days. (4) MeI (10 equiv.), NaH (5 equiv.), THF, 0 to 23 °C. (5) MsCl (3 equiv.), TEA (3 equiv.), DCM, 0 to 23 °C *then*, LiAlH_4_ (8 equiv.), THF, 0 °C to *Δ*.

Continuing the theme of engaging **CL5a** in stereoablative processes, we identified Pd(OH)_2_, Pearlman's reagent,^20^ as a highly active catalyst that can indiscriminately reduce all stereoisomers obtained from the cycloaddition to **CL1** scaffolds. A route was established that consists of [3 + 2] dipolar cycloaddition, LiAlH_4_ reduction, and Pd(OH)_2_ hydrogenation under atmospheric pressure that gives access to numerous **CL1** scaffolds in a preparative scale (Scheme 3).

An advantage of our strategy over previous methods to access **CL1** is that it proceeds with retention of the dipolarophile stereochemistry, eliminating potential ambiguity of the β–β′ relative stereochemistry. For example, isolation and total synthesis of **20** through Stobbe condensation^21^ has been reported but our obtained ^1^H and ^13^C data did not match the reported values. Gratifyingly, (±)-dihydro-3′,4′-dimethoxy-3′,4′-demethylenedioxycubebin (**20**) has been reported elsewhere and our obtained spectra matched.^22^ We believe this is due to a misassignment in the earlier report, which occurred because the Stobbe condensation route proceeds through a non-selective hydrogenation, furnishing both the *trans* and *cis* scaffolds. Thus, the natural product isolated therein was (±)-2,3-desmethoxy seco-isolintetralin (**21**). We were able to confirm this through synthesis of **21** using dimethyl maleate as the dipolarophile, which indeed matched the reported spectral values, leading to its structural revision.

Other **CL1** scaffolds that can be accessed through this sequence are (±)-dihydrocubenin (**22**)^23^ and 2,3,4-trimethoxy substituted **23**. The latter example showcases the tolerability of the cycloaddition and hydrogenation to substitution in all positions of the aryl groups. Other notable examples include, 4-methoxy substituted *trans***24** and *cis***25** and 3,4-dimethoxy substituted (±)-secoisolariciresinol dimethyl ether (**26**)^24^ and **27**, which were obtained in good yields over 3 steps (35–79%). The latter two examples can be further elaborated to other **CL1** scaffolds. For example, **26** can be methylated to form (±)-phyllanthin (**28**),^25^ which has been found to have promising anti-hyperalgesia effects, and **27** can be deoxygenated to access the *meso* compound terameprocol (**29**), which is currently in a clinical trial for high grade glioma.^26^

### Synthesis of dibenzylbutyrolactones (**CL2**)

**Scheme 4:**
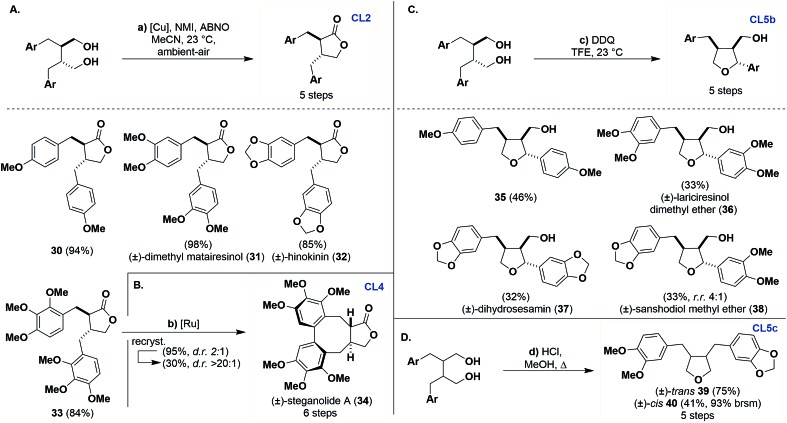
Synthesis of dibenzylbutyrolactones (**CL2**), dibenzocyclooctadienes (**CL4**) and furan (**Cl5b** and **Cl5c**) from dibenzylbutanes (**CL1**). Reagents and conditions: (a) Cu(MeCN)_4_OTf (5 mol%), 2,2′-bpy (5 mol%), ABNO (1 mol%), NMI (10 mol%), MeCN, ambient-air, 23 °C, 12 h. (b) RuO_2_·*x*H_2_O (2 equiv.), TFA (30 equiv.), TFAA (15 equiv.), BF_3_–OEt_2_ (8 equiv.), DCM, –10 to 23 °C, N_2_, 24 h. (c) DDQ (1.5 equiv.), TFE, 23 °C, 6–8 h. (d) HCl (5–10 drops), MeOH, *Δ*, 4–7 days.

Previously **CL1** scaffolds have been oxidized to **CL2** using stoichiometric Fétizon's reagent or using catalytic ruthenium complexes.^27^ We found that the catalytic protocol for aerobic oxidative lactonization of diols reported by Stahl^28^ was exceedingly effective in conversion of **CL1** to **CL2** products (Scheme 4a). The generality of this transformation is quite broad when varying the aryl oxidation patterns, as demonstrated by monomethoxy **30** and trimethoxy **33** as well as natural products (±)-dimethyl matairesinol (**31**) and (±)-hinokinin (**32**), which were obtained in excellent to good yields (84–98%).^29^ The operational simplicity of this reaction in comparison to previous methods is noteworthy and, to the best of our knowledge, represents its first use in the synthesis of bioactive natural products.

### Synthesis of dibenzocyclooctadienes (**CL4**)

We can access **CL4** products using oxidative coupling conditions reported by Robin,^30^ which is demonstrated by the synthesis of (±)-steganolide A (**34**) from lactone **33** in 95% yield (Scheme 4b). Although reported as atroposelective in our hands this reaction produces two atropisomers (d.r. 2 : 1). The selectivity of the product can be improved by recrystallization (30%, d.r. > 20 : 1).

### Synthesis of furans (**CL5a–c**)

Entry into **CL5b** from **CL1** has been previously described by Ward with the use of 2,3-dichloro-5,6-dicyanobenzoquinone (DDQ) in acetic acid on a single substrate.^31^ This work was reproducible but the isolation of the desired product was tedious, requiring several purifications to access pure materials, resulting in low yields (<10%). A solvent screening revealed that 2,2,2-trifluoroethanol (TFE) gave full conversion of the starting material and a relatively cleaner reaction profile (Scheme 4c). With these conditions, we can access 4-methoxy substituted **35**, (±)-lariciresinol dimethyl ether (**36**),^32^ and (±)-dihydrosesamin (**37**)^33^ in modest yields (32–46%). Interestingly, we can also use an unsymmetrical example and gain access to pure (±)-sanshodiol methyl ether (**38**)6*b* in 33% yield and with good selectivity (r.r. 4 : 1).^34^

Entry into **CL5c** can be accomplished from **20** and **21** through dehydration under refluxing methanolic HCl to afford **39** (75%) and **40** (41%, 93% brsm) in good yields (Scheme 4d).^35^ These examples again highlight the opportunities available for the stereodivergent synthesis of CLs controlled by the stereochemistry of the dipolarophile.

**Scheme 5:**
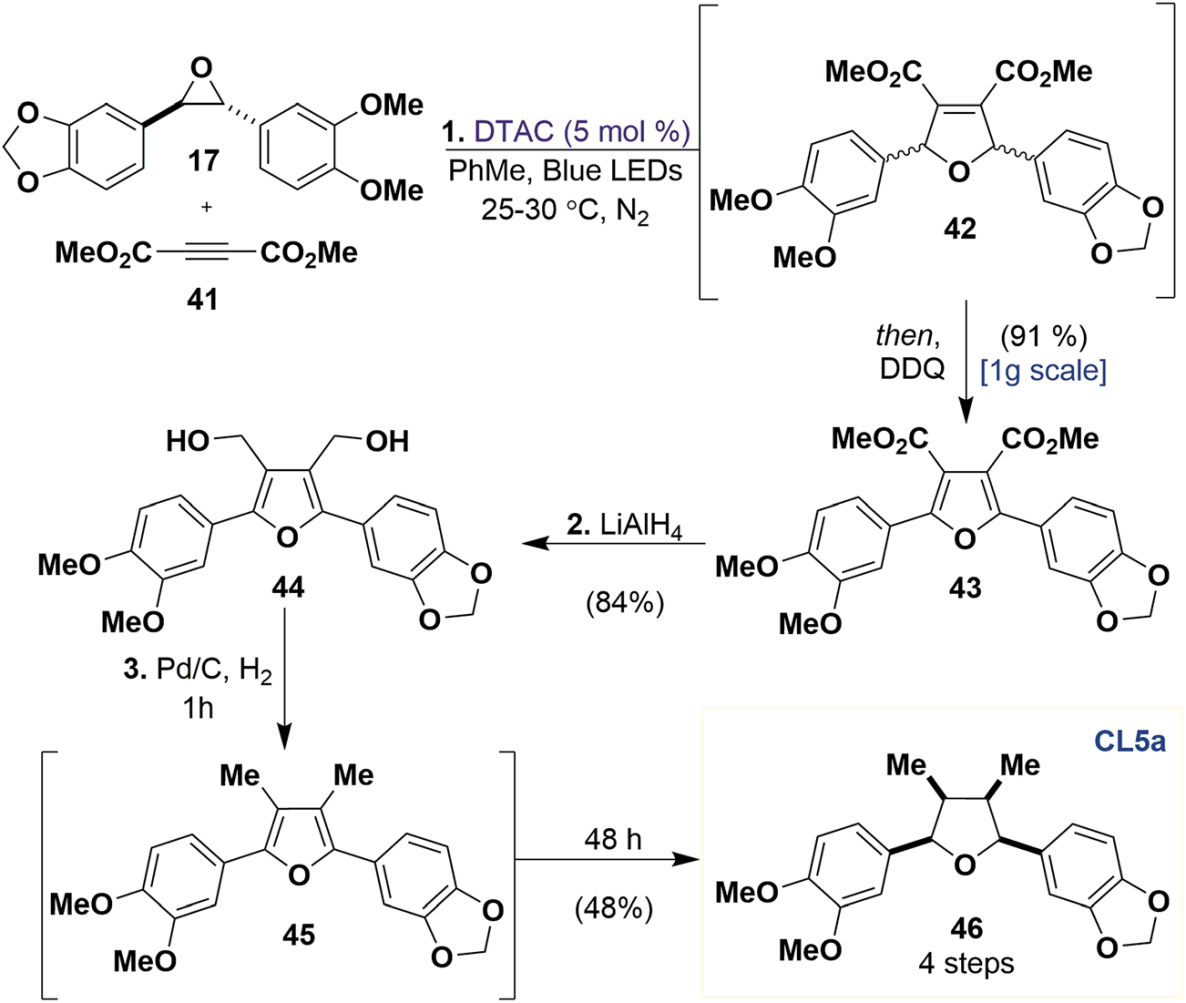
Total synthesis of a proposed **CL5a** natural product. Reagents and conditions: (1) DTAC (5 mol%), **17** (1 equiv.), **41** (2.5 equiv.), PhMe, Blue LEDs, 25–30 °C, N_2_, 8 days, *then* DDQ (1.6 equiv.), 24 h. (2) LiAlH_4_ (6 equiv.), THF, 0 to 23 °C. (3) Pd/C (10 mol%), 3 : 1 EtOH/EtOAC, H_2_ (balloon), 23 °C, 48 h.

Lastly, **CL5a** represents the greatest challenge for the effective use of the [3 + 2] dipolar cycloaddition, as it can be found in nature possessing all possible variations in its relative stereochemistry.^36^ We set out to access the all-*cis***CL5a** scaffold due to its known ability to promote neuronal differentiation and neurite growth.^37^ In particular, we targeted tetrahydrofuran **46** (Scheme 5), a reported natural product.^38^

The synthesis commences with [3 + 2] dipolar cycloaddition between **17** and dimethyl acetylenedicarboxylate (DMAD) (**41**). At the completion of this reaction, in a one-pot fashion, DDQ is added to promote aromatization to furan **43** in 91% yield. Furan **43** was reduced to diol **44** with LiAlH_4_ in 84% yield on a gram scale. It was found that diol **44** could be directly hydrogenated to **46** with Pd/C under a hydrogen atmosphere, which proceeds through the intermediacy of furan **45**, in 48% yield (35% yield over 4 steps). Unfortunately, the obtained ^1^H and ^13^C data of synthetic **46** did not match the natural product isolation values. This is due to misassignment of the isolated natural product as we observed *C*_1_ symmetry in the ^1^H NMR spectra of **46**, which is characteristic of other known all-*cis***CL5a** compounds.^39^ Although **46** was ultimately not a reported natural product, this route represents the shortest and most efficient to date to access all *cis*-**CL5a** scaffolds.^36^

### Synthesis of arylnaphthalenes and their derivatives (**CL3**)

**Scheme 6:**
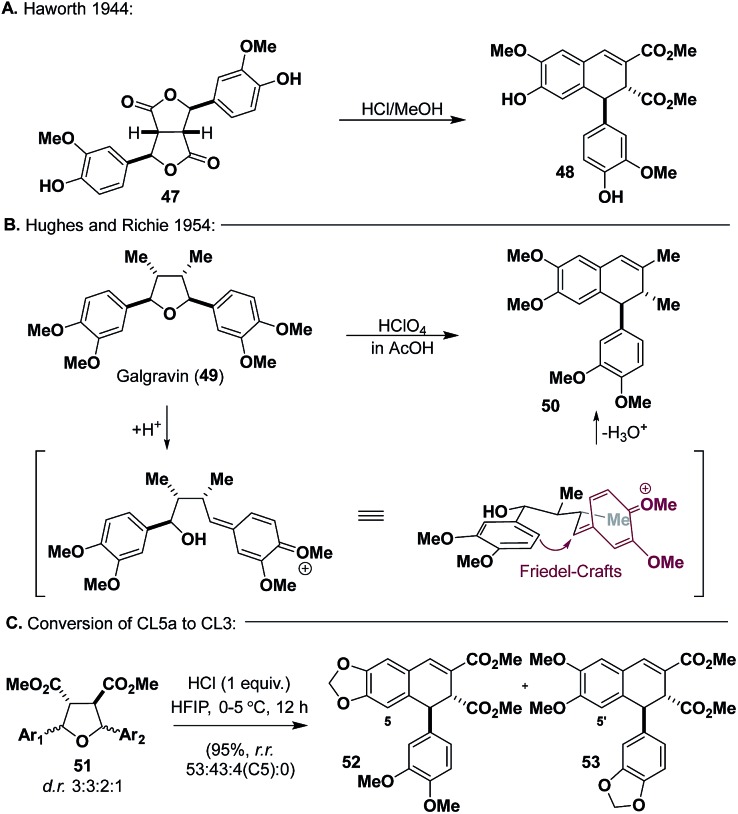
Instructive examples for conversion of **CL5a** to **CL3**.

We wanted to take advantage of the oxidation state already present on **CL5a** (**51**) for the synthesis of aryltetralin natural products. Inspiration for the conversion of **51** into **CL3** was reported by Haworth and co-workers (Scheme 6a), wherein they used an acid-mediated cyclization of bislactone **47**, which is isoelectronic with **51**, to synthesize dihydronaphthalene **48**.^40^ Additionally, Hughes and Richie (Scheme 6b) demonstrated that galgravin (**49**), a **CL5a** natural product, undergoes rearrangement to form dihydronaphthalene **50** under acidic conditions.^41^ Seemingly, both **48** and **50** arise from *p*QM formation, Friedel–Crafts alkylation, and dehydration.

**Scheme 7:**
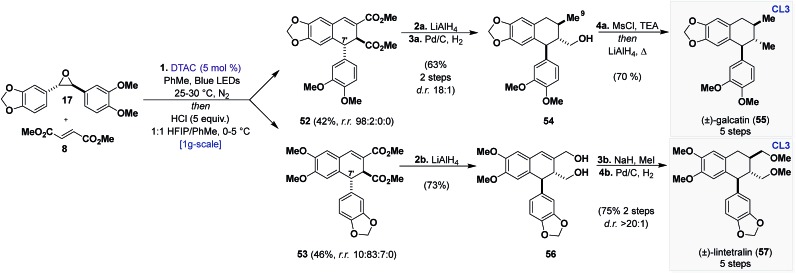
Total synthesis of aryltetralin (**CL3**) natural products (±)-galcatin (**55**) and (±)-lintetralin (**57**). Reagents and conditions: (1) DTAC (5 mol%), **17** (1 equiv.), **8** (3 equiv.), PhMe, Blue LEDs, 25–30 °C, N_2_, 5 days, *then* HCl (5 equiv.), 1 : 1 HFIP/PhMe, 0–5 °C, 36 h. (2a) LiAlH_4_ (6 equiv.), THF, 0 °C, 1 h. (3a) Pd/C (10 mol%), EtOH, H_2_ (balloon), 23 °C, 12 h. (4a) MsCl (1.5 equiv.), TEA (1.5 equiv.), THF, 0 to 23 °C, 12 h *then* LiAlH_4_ (6 equiv.), *Δ*, 1 h. (2b) LiAlH_4_ (6 equiv.), THF, 0 °C, 1 h. (3b) MeI (10 equiv.), NaH (5 equiv.), THF, 0 to 23 °C, 24 h. (4b) Pd/C (10 mol%), EtOAc, H_2_ (balloon), 23 °C, 12 h.

An extensive screening of Lewis and Brønsted acids^19^ revealed that HCl (1 equiv.) in HFIP could promote the desired reaction in high yields (Scheme 6c) (95%), affording **52** and **53** as the two major products (as single diastereomers) with trace amounts of a regioisomer of **52** (C5) from **51**. Although inseparable through chromatography, it was eventually found that the mixture could be resolved through trituration with diethyl ether or after LiAlH_4_ reduction and flash chromatography. With access to both regioisomers secured, we proceeded to advance them to known aryl tetralin natural products, principally, (±)-galcatin (**55**) (from **52**) and (±)-lintetralin (**57**) (from **53**) (Scheme 7).

We developed a one-pot procedure beginning with cycloaddition between **17** and **8**, followed by addition of HFIP and HCl (5 equiv.) at 0 °C, which virtually gave a 1 : 1 mixture of **52** and **53**. Flash chromatography followed by trituration afforded **52** in 42% yield and with excellent selectivity (r.r. 98 : 2 : 0 : 0) and **53** in 46% yield and with good selectivity (r.r. 10 : 83 : 7 : 0).

Sequential reduction of **52** with LiAlH_4_ and Pd/C under a hydrogen atmosphere reduces the esters to alcohols and the endocyclic alkene to the tetralin core, the latter occurs on the face opposite of the C7′ aryl group. This furnished the all-*trans* tetralin **54** in 63% yield over 2 steps with high selectivity (d.r. 18 : 1). Interestingly, the reduction of the alkene is also accompanied by deoxygenation of the allylic alcohol (C9). Although not planned, this deoxygenation was welcomed, as early attempts of a global deoxygenation proved cumbersome. To complete the synthesis deoxygenation of **54** was achieved through *in situ* activation with MsCl followed by reduction with LiAlH_4_, providing (±)-galcatin (**55**) in 5 steps in an overall 33% yield.^42^ Reduction of **53** with LiAlH_4_ gave diol **56** in 73% yield as a single regioisomer after flash chromatography. Successive methylation (MeI) and hydrogenation with Pd/C in EtOAc gave (±)-lintetralin (**57**) in 75% yield over 2 steps (45% overall yield over 5 steps).^43^

### Application to the synthesis of heterolignans

**Scheme 8:**
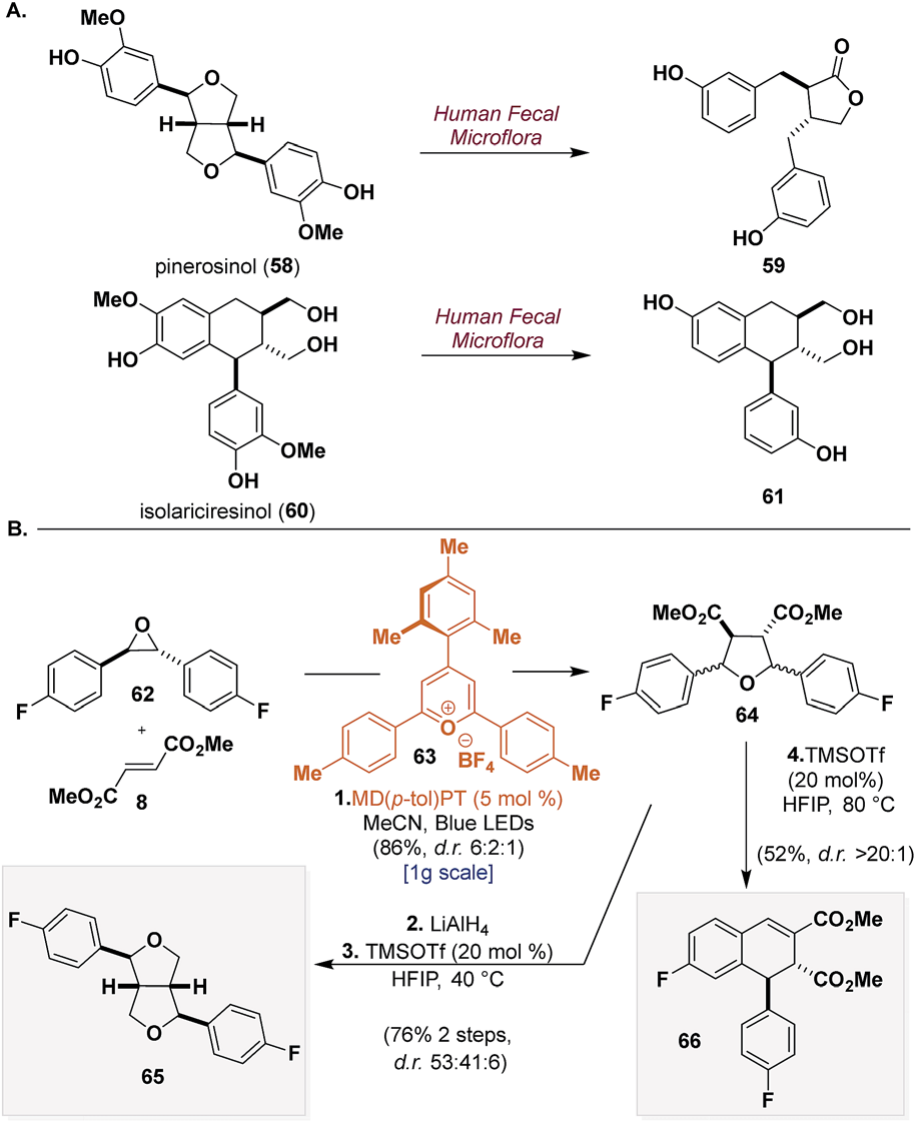
Application of the outlined synthetic blueprints to the synthesis of fluorinated heterolignans. Reagents and conditions: (1) MD(*p*-tol)PT (5 mol%), **62** (1 equiv.), **8** (1.2 equiv.), MeCN, Blue LEDs, 25–30 °C, N_2_, 12 h. (2) LiAlH_4_ (6 equiv.), THF, 0 to 23 °C, 1 h. (3) TMSOTf (20 mol%), HFIP, 40 °C, 24 h. (4) TMSOTf (20 mol%), HFIP, 80 °C, 24 h.

To demonstrate a final application of this unified synthetic platform we sought to synthesize CL scaffolds bearing unnatural functionality. This is particularly difficult for most synthetic strategies because they rely on the reactivity of electron-rich aryl groups to access crucial intermediates. Pinerosinol (**58**) and isolariciresinol (**60**) are two CLs that have shown promising biological activities but both have also been shown to be metabolically unstable to human fecal microflora, affording enterolactone (**59**) and metabolite (**61**), respectively (Scheme 8a).^44^ A reasonable modification to improve their metabolic stability would be replacement of the oxygen appendages on the aryl groups with fluorine.^45^

To this end (Scheme 8b), using 4-mesityl-2,6-di-*p*-tolylpyrylium tetrafluoroborate (MD(*p*-tol)PT) (**63**) (*λ*_max_ = 420 nm, [24^+^*/24˙] = +2.27 V *vs.* SCE), a catalyst that was identified in our earlier work that can generate electron-deficient carbonyl ylides,^10^ we can submit **62** to [3 + 2] dipolar cycloaddition with dimethyl fumarate (**8**) and isolate **64** in 86% yield as a mixture of inconsequential diastereomers (d.r. 6 : 2 : 1). LiAlH_4_ reduction and TMSOTf (20 mol%) catalyzed cyclization in HFIP of **64** furnish **65** in 76% yield over 2 steps. Although not selective (d.r. 53 : 41 : 6), the two major compounds can be easily separated providing two new notable analogues of the furofuran scaffold. Lastly, from **64** under similar acidic conditions but with elevated temperatures, we can induce rearrangement to form dihydronaphthalene **66** in 52% yield and with excellent selectivity (d.r. > 20 : 1). These molecules exemplify the modularity of our strategy to access CLs and demonstrate how our synthetic blueprints are suited to access natural product scaffolds possessing unnatural functionality.

## Conclusion

We have identified short synthetic routes to all subtypes of the CL family of natural products from common building blocks. Key to the success of our strategy was the identification of a highly convergent [3 + 2] dipolar cycloaddition that is catalyzed by DTAC, a catalyst that was unknown for photoredox applications. Through the use of UV-Vis spectroscopy it was demonstrated that DTAC engages electron-rich epoxides through an outer-sphere SET by steric inhibition of a CT complex, which would otherwise funnel the reaction pathway through an unproductive inner-sphere SET. Finally, our synthetic strategies to access each subtype of CLs in a modular and convergent manner should make these molecules more available for SAR investigation. Such studies will be more effective due to their ability to introduce unnatural functionality, such as fluorine, which would be challenging using preexisting routes.

## Conflicts of interest

There are no conflicts to declare.

## Note added after first publication

This article replaces the version published on 5th July 2019, which contained errors in Scheme 4 and 5.

## Supplementary Material

Supplementary information

Crystal structure data
