# Supplementary material for: A sterically encumbered photoredox catalyst enables the unified synthesis of the classical lignan family of natural products
Source: Chem Sci. 2019 Jul 5;10(33):7746–54. doi: 10.1039/c9sc02682g (PMC6761868; doi:10.1039/c9sc02682g)
Supplement: Supplementary file 1 [file SC-010-C9SC02682G-s001.pdf]

Electronic Supplementary Material (ESI) for Chemical Science.
This journal is © The Royal Society of Chemistry 2019

Sterically-Encumbered Photoredox Catalyst Enables the Unified Synthesis of the Classical Lignan Family of Natural Products

Edwin Alfonzo †, Aaron B. Beeler †*
† Department of Chemistry, Boston University, Boston, Massachusetts 02215, United States.
Correspondence to: beelera@bu.edu

Table of Contents (Page)

I. General Information (**pp. 2**)

II. Summary of Screened Reaction Conditions for Carbonyl Ylide Formation of Electron-Rich Epoxides (**pp. 3**)

III. Synthesis and Photophysical Characterization of 2,6-di-tert-butylanthracene-9,10-dicarbonitrile (DTAC) (**pp. 4**)

IV. UV-Vis Spectroscopy Studies of 9, 10-dicyanoanthracene (DCA) and DTAC (**pp. 6**)

V. Experimental Procedures and Characterization of Reported Compounds (**pp. 10**)

VI. References (**pp. 50**)

VII. ^{1}H and ^{13}C-NMR spectra of Reported Compounds (**pp. 51)**

VIII. X-Ray Crystal Structure of Compound **29** (**pp. 152**)

General Information:

^1H NMR and ^{13}C NMR spectra were recorded at ambient temperature on a Varian Agilent-500 MHz VNMRS (500 and 126 MHz, respectively), and are internally referenced to the residual protio solvent signal ($CDCl_3$: δ 7.26 and 77.0 ppm). Data for ^1H NMR are reported as follows: chemical shift, integration, multiplicity (brs = broad singlet, s = singlet, d = doublet, t = triplet, q = quartet, m = multiplet, overlap = overlapping peaks) and coupling constants in Hz. Data for ^{13}C NMR are reported in terms of chemical shift and overlapping carbons are noted by an underline. High-resolution mass spectra and UV-Vis spectra were obtained in the Boston University Chemical Instrumentation Center using a Waters Q-TOF APIUS mass spectrometer and a Varian Cary 100 Vio UV-Vis, respectively. Commercial reagents were purified prior to use following the guidelines of Chai and Armarego.[1] All solvents were purified according to the method of Grubbs.[2] Organic solutions were concentrated under reduced pressure on a Büchi rotary evaporaor using a water bath. Chromatographic purification of products was accomplished by flash chromatography on Silicycle F60 silica gel or Sorbtech neutral alumina 32-63 μm according to the method of Still.[3] All reactions were carried out in well ventilated fume hoods. Reaction were monitored by thin-layer chromatography (TLC) using Silicycle 250 μm silica gel plates or Sorbtech neutral alumina 250 μm. Visualization of the developed chromatogram was performed by irradiation with a 254 nm Ultra-Violet (UV) light or treatment with aqueous potassium permanganate ($KMnO_4$) or ethanolic phosphomolybdic acid (PMA) followed by heating. Yields refer to purified compounds unless otherwise noted. Diastereoelectivity and regiochemical selectivity for reactions were determined by crude ^1H NMR prior to purification.

II. Summary of Screened Reaction Conditions for Carbonyl Ylide Formation from Electron-Rich Epoxides:

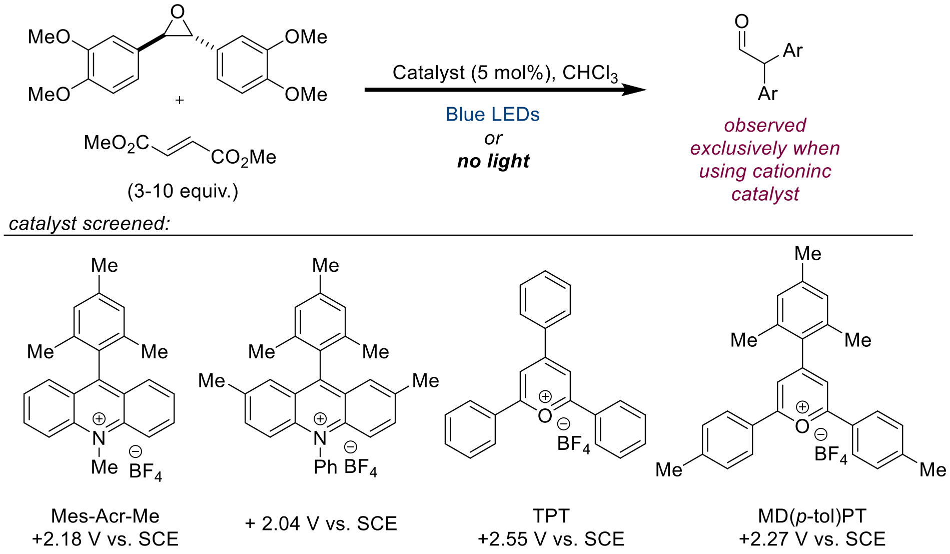

We found that in general cationic photoredox catalysts can behave as ground state Lewis acids, catalyzing the Meinwald rearrangement of epoxides to aldehydes in the absence of light. For examples of pyrylium and acridinium salts behaving as Lewis acids, see: a) Miranda, M.A.; Garcia, H. *Chem. Rev.* **1994**, *94*, 1063-1089; b) Clark, E.R.; Ingleson, M.R. *Angew. Chem. Int. Ed.* **2014**, *53*, 11306-11309.

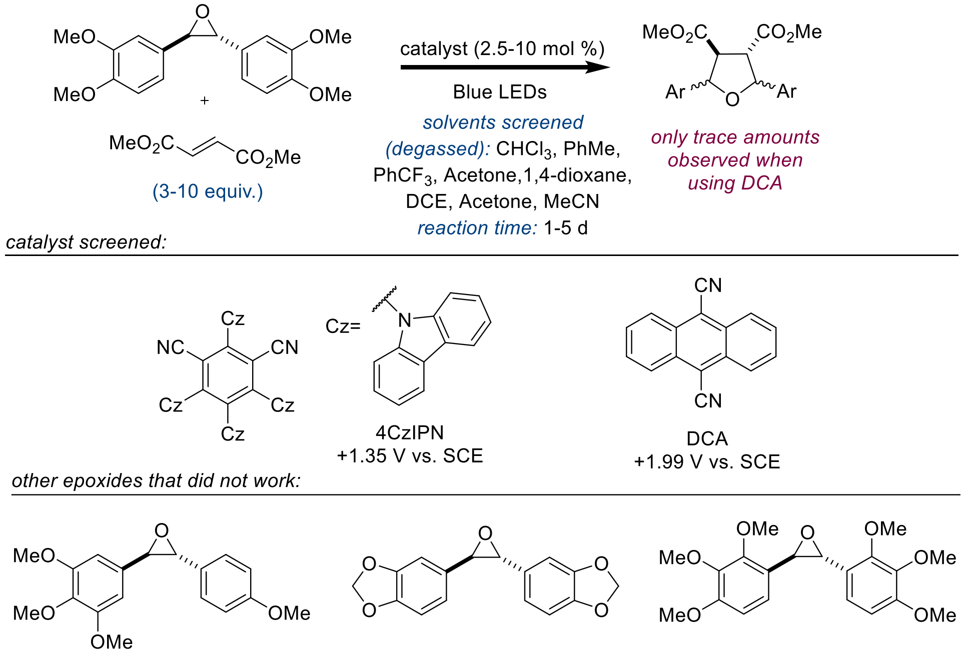

Other catalyst, epoxides, and solvent conditions that were explored are summarized above. Ultimately, we were only able to detect trace amounts of product formation when using DCA as the catalyst.

III. Synthesis and Photophysical Characterization of 2, 6-di-tert-butylanthracene-9, 10-dicarbonitrile (DTAC)

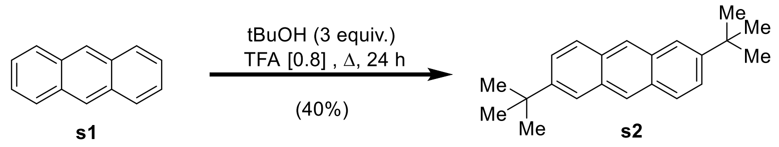

Synthesis of **s2**: A 250-mL round bottom flask with a magnetic stirring bar was charged with **s1** (10 g, 56.10 mmol, 1 equiv.), TFA (70 mL, [0.8 M]), and *t*BuOH (16 mL, 167 mmol, 3 equiv.). The stirred reaction was refluxed and left to stir for a 24 h period. At the completion of the stir period, the reaction was cooled to room temperature and water (100 mL) was added slowly to the reaction mixture. The mixture was carefully introduced into a separatory funnel with the assistance of hexanes and extracted thrice with hexanes. The combined organic fractions, which is heterogeneous, was passed through a fritted funnel and the collected solid was further washed with hexanes. This yielded a white solid which can be used without any further purification.

% yield: 40% (6.4 g)

Physical state: white solid

^{1}H NMR (500 MHz, Chloroform-*d*) δ 8.33 (s, 2H), 7.94 (d, *J* = 8.9 Hz, 2H), 7.88 (s, 2H), 7.55 (dd, *J* = 8.9, 1.5 Hz, 2H), 1.46 (s, 18H).

^{13}C NMR (126 MHz, Chloroform-*d*) δ 147.41, 131.69, 130.60, 127.87, 125.53, 124.91, 122.43, 35.08, 31.15.

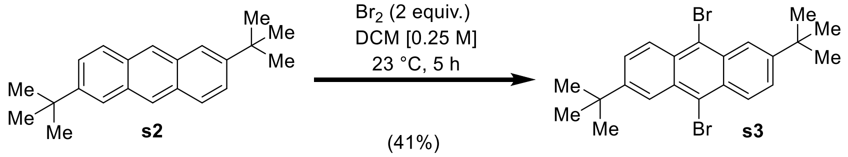

Synthesis of **s3**: A 250-mL round bottom flask with a magnetic stirring bar was charged with **s2** (2 g, 6.88 mmol, 1 equiv.) and DCM (28 mL, [0.25 M]). The reaction was cooled to 0 °C (water/ice bath) and bromine (720 µL, 14 mmol, 2 equiv.) was added dropwise *via* syringe. The reaction was left to warm to room temperature and stirred for a 5 h period. At the completion of the stir period the reaction was quenched through slow addition of saturated $NaHCO_3$, until the red color of the reaction disappeared. The mixture was introduced into a separatory funnel and extracted thrice with DCM. The combined organic fractions were washed with brine and dried over $MgSO_4$. The organic mixture was concentrated *in vacuo* and purified by recrystallization from toluene (PhMe).

% yield: 41% (1.26 g)

Physical state: light yellow crystals

^{1}H NMR (500 MHz, Chloroform-*d*) δ 8.51 (d, *J* = 9.2 Hz, 2H), 8.46 (s, 2H), 7.71 (dd, *J* = 9.2, 1.6 Hz, 2H), 1.49 (s, 18H).

^{13}C NMR (126 MHz, Chloroform-*d*) δ 149.91, 130.77, 130.01, 128.14, 127.04, 123.08, 122.72, 35.44, 31.03.

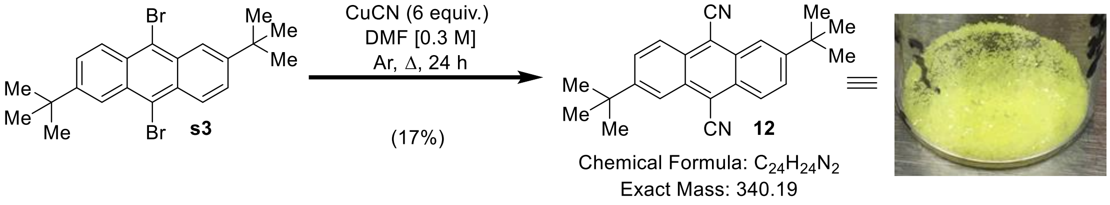

Synthesis of **2,6-di-tert-butylanthracene-9,10-dicarbonitrile (DTAC) (12):** A flame dried 50-mL two neck round bottom flask with a magnetic stirring bar was charged with **s3** (1.0 g, 2.23 mmol, 1 equiv.), CuCN (1.2 g, 13.4 mmol, 6 equiv.) and DMF (7.5 mL, [0.3 M]). One neck of the reaction vessel was adapted with a septum and the other with a reflux condenser. The stirred reaction was then sparged with Argon (Ar) for a 30 min period, adapted with an Ar balloon above the reflux condenser, and then refluxed for 24 h. After completion of the stir period, the reaction was cooled to room temperature and 30 % NH_3 soln. in water (30 mL) was added. After a 5 min stir period, the mixture was filtered through a fritted funnel and the resulting solid was washed three times with 30 % NH_3 in water (10-15 mL). The solid was dissolved in DCM (100-150 mL), dried over $MgSO_4$, and concentrated *in vacuo.* The resultant material was purified by flash chromatography and recrystallized from PhMe.

Scale: 2.23 mmol, 26 mmol

Flash Chromatography: 20:1 Hex/EtOAc

TLC: R_f=0.5 in 20:1 Hex/EtOAc

Recrystallization: PhMe

% yield: 17% (128 mg), 15% (1.3 g)

Physical state: Chartreuse or yellow/green solid

^{1}H NMR (500 MHz, Chloroform-*d*) δ 8.42 (d, *J* = 9.1 Hz, 2H), 8.35 (d, *J* = 1.6 Hz, 2H), 7.91 (dd, *J* = 9.1, 1.6 Hz, 2H), 1.50 (s, 18H).

^{13}C NMR (126 MHz, Chloroform-*d*) δ 152.92, 132.05, 131.10, 129.40, 125.87, 120.57, 116.38, 110.51, 35.75, 30.90.

HRMS-ESI (m/z): calculated for $C_{24}H_{24}N_2$ + H^+: 341.2018 found 341.200

Photophysical Characterization of 2,6-di-tert-butylanthracene-9,10-dicarbonitrile (DTAC, 12): A sample of 10 µM DTAC (**12**) was prepared through serial dilution with dry chloroform and degassed by bubbling N_2 for 10-15 minutes under sonication. The resultant solution was transferred to a 4 mL quartz cell and sealed with a PTFE cap and Parafilm. A UV-Vis spectrum of this solution was obtained and normalized (shown below). An emission spectrum (1 nm step size, 5 nm bandwidth) was obtained by irradiating the sample with the frequency of light corresponding to its longest absorbing wavelength (as determined through UV-Vis spectroscopy λ_{max}=431 nm), followed by collection 15 nm above this wavelength to avoid interference with the light source. The obtained spectrum was normalized (shown below).

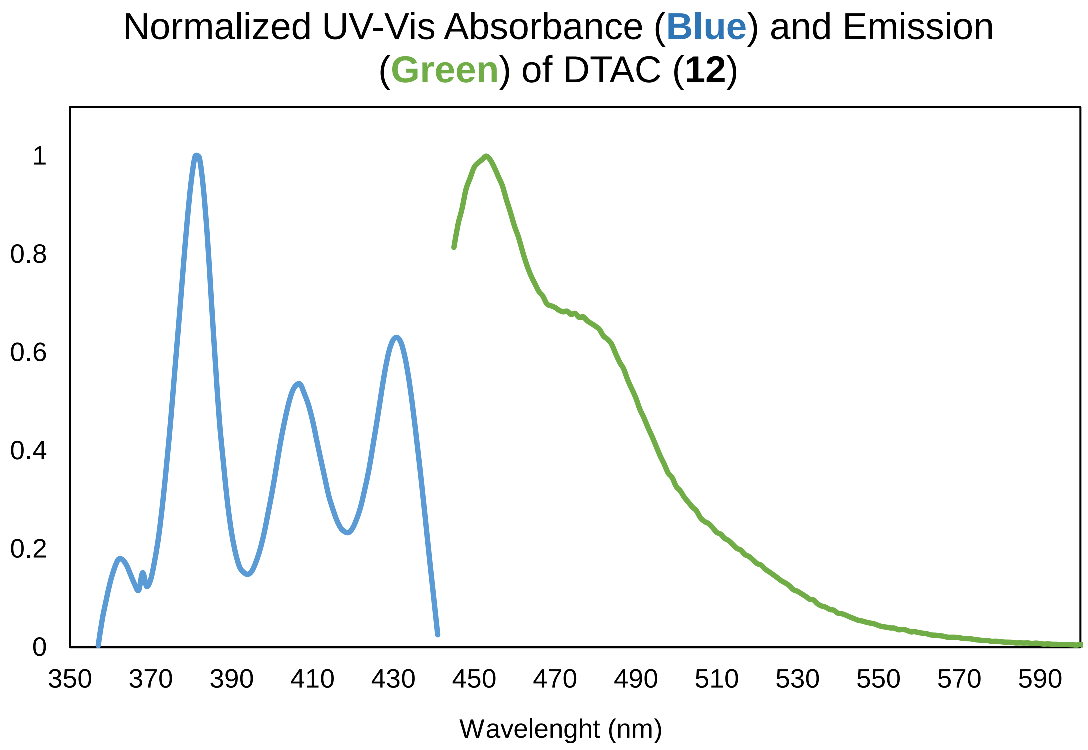

Absorbance λ_{max}=431 nm; Emission λ_{max}=453 nm

Gibbs Energy of Photoinduced Electron Transfer Equation for DTAC:

$$E^*_{red}\ (cat^*/cat\bullet-) = E_{red}\ (cat/cat\bullet-) + E_{0,0}$$

$$E_{0,0} = 2.8 \text{ eV}$$

$$E_{red} = -0.99 \text{ V vs. SCE in DCM (ref. 5)}$$

$$\therefore [DTAC^*/DTAC\bullet-] = +1.81 \text{ V vs. SCE in DCM}$$

IV. UV-Vis Spectroscopy Studies of 9, 10-dicyanoanthracene (DCA) and DTAC:

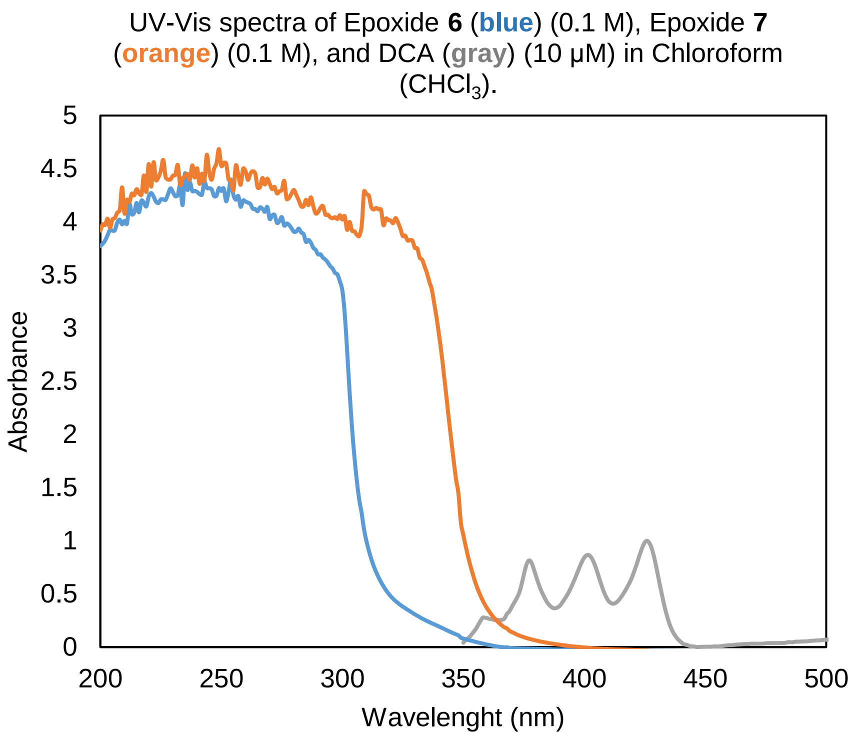

Physical solutions of DCA (left), DCA-Epoxide **6** (center), and DCA-Epoxide **7** (right) in Chloroform ($CHCl_3$).

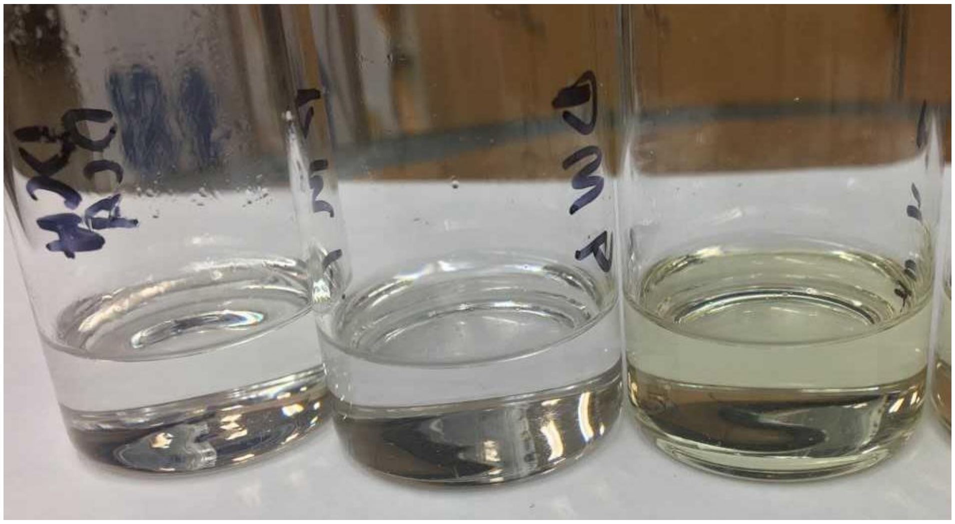

Note: A color change was observed when DCA was mixed with epoxide **7** (right) but not with epoxide **6** (center).

UV-Vis spectra of DCA (10 µM) (**green**), DCA-Epoxide **6** (10 µM and 0.1 M) (**yellow**), and DCA-Epoxide **7** (10 µM and 0.1 M) (**blue**) in Chloroform ($CHCl_3$).

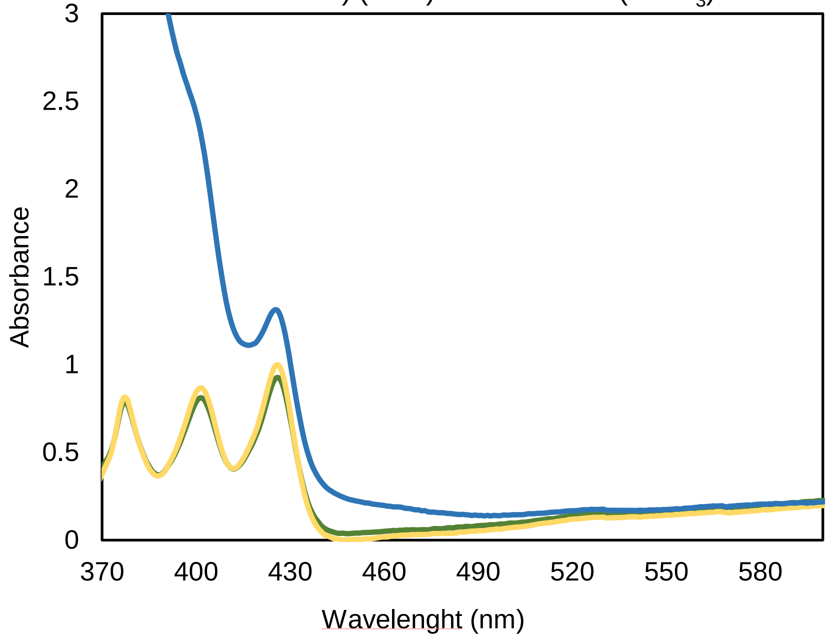

Physical solutions of DTAC (left), DTAC-Epoxide **6** (center), and DCA-Epoxide **7** (right) in Chloroform ($CHCl_3$).

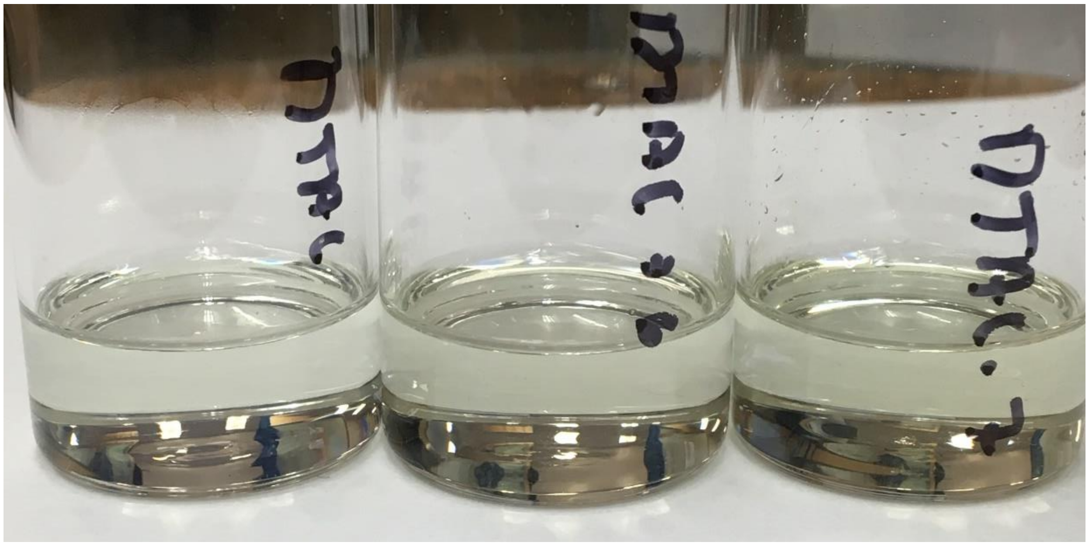

Note: No color change was observed when mixing DTAC with either epoxide **6** or **7**.

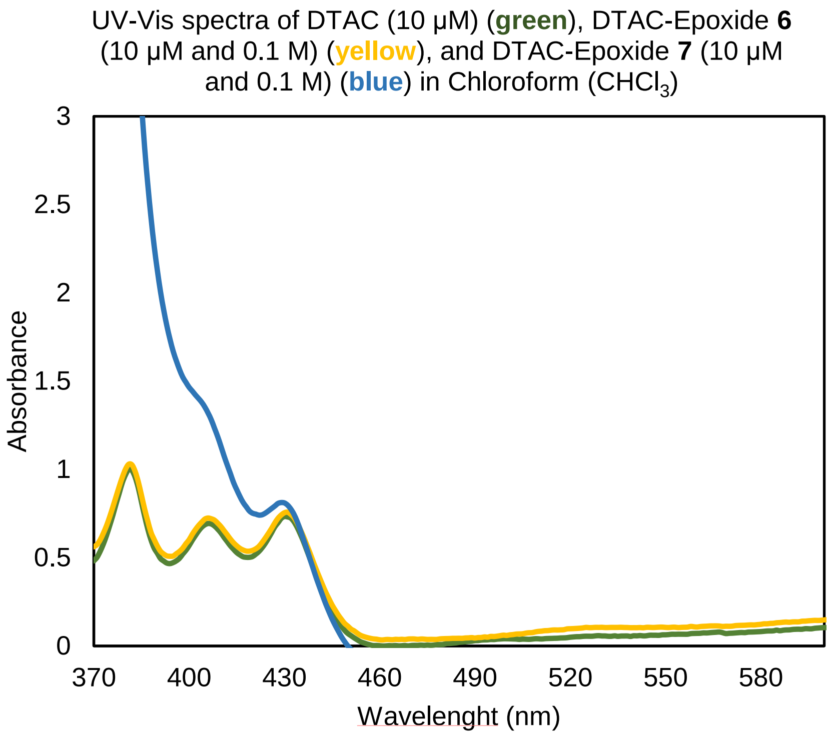

V. Experimental Procedures and Characterization of Reported Compounds

General procedure for sulfonium-ylide mediated epoxidation:[6]

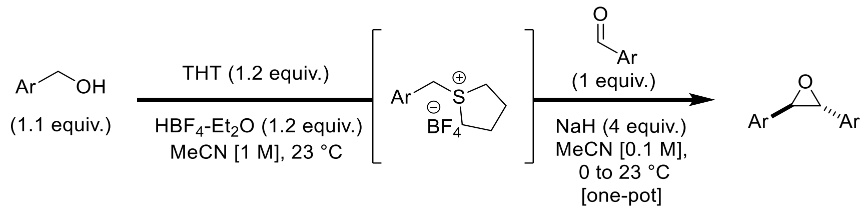

A flame-dried round bottom flask (for 10 mmol: 250-mL, 50 mmol: 1 L) with a magnetic stirring bar was charged with benzyl alcohol (1.1 equiv.), tetrahydrothiophene (THT) (1.2 equiv.), and acetonitrile (MeCN) ([1 M] *with respect to (w.r.t.)* the benzyl alcohol).To the stirred solution was added tetrafluoroboric acid diethyl ether complex (HBF_4-OEt_2) (1.2 equiv.) dropwise (on a 50 mmol scale the reaction was cooled to 0 °C (water/ice bath) prior to addition of the acid) and the reaction was left to stir until full consumption of the alcohol was observed by TLC (1:1 hexanes (Hex)/ethyl acetate (EtOAc)) or for a 12 h period. The reaction was then cooled to 0 °C, diluted with MeCN ([0.1 M] *w.r.t.* aldehyde), and sodium hydride (NaH) (4 equiv.) was added in small portions. After stirring for 5 min the aldehyde (1 equiv.) was added dropwise or in small portions (solids). The reaction was left to stir until full consumption of the aldehyde was seen by TLC (4:1 Hex/EtOAc) or for a 12 h period. Then, the reaction was cooled to 0 °C and water was added dropwise until no further gas evolution was observed. The mixture was concentrated *in vacuo* and the resultant residue was introduced into a separatory funnel with the assistance of water and EtOAc. The mixture was extracted thrice with EtOAc and the combined organic fractions were dried over $MgSO_4$ and concentrated *in vacuo*. The material was purified by flash chromatography. Diastereoselectivity was determined by crude ^{1}H NMR prior to purification unless otherwise noted. No efforts to separate the diastereomers of the epoxides were undertaken, as these were used as mixtures for sequential reactions.

Synthesis of **17** from benzo[d][1,3]dioxol-5-ylmethanol and 3,4-dimethoxybenzaldehyde:

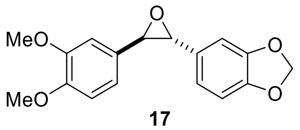

17

Scale: 50 mmol

Flash Chromatography: 4:1 Hex/DCM + 3 % TEA → 4:1 Hex/DCM + 1 % MeOH

TLC: R_f= 0.3 in 4:1 Hex/DCM + 1 % MeOH, Stain: PMA (color: green)

% yield: 90% (13.41 g)

Physical state: light yellow solid

d.r. 6:1

^{1}H NMR (500 MHz, Chloroform-*d*) δ 6.92 (dd, *J* = 8.2, 1.9 Hz, 1H), 6.84 – 6.77 (overlap, 5H), 5.97 (s, 2H), 3.90 (s, 3H), 3.89 (s, 3H), 3.77 (s, 2H).

^{13}C NMR (126 MHz, Chloroform-*d*) δ 149.29, 149.13, 148.06, 147.69, 131.10, 129.53, 119.51, 118.28, 111.07, 108.31, 107.81, 105.42, 101.16, 62.70, 62.68, 55.98, 55.89.

Synthesis of **s4** from benzo[d][1,3]dioxol-5-ylmethanol and benzo[d][1,3]dioxole-5-carbaldehyde:

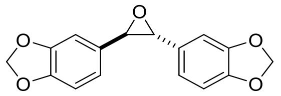

s4

Scale: 10 mmol

Flash Chromatography: 4:1 Hex/EtOAc + 3 % TEA → 4:1 Hex/EtOAc

TLC: R_f= 0.3 in 4:1 Hex/EtOAc, Stain: PMA (color: green)

% yield: 100% (2.84 g)

Physical state: white solid

d.r. 5:1

^{1}H NMR (500 MHz, Chloroform-*d*) δ 6.83 (dd, *J* = 8.0, 1.6 Hz, 2H), 6.80 (d, *J* = 8.0 Hz, 2H), 6.78 (d, *J* = 1.6 Hz, 2H), 5.97 (s, 5H), 3.74 (s, 2H).

^{13}C NMR (126 MHz, Chloroform-*d*) δ 148.20, 147.85, 131.14, 119.66, 108.44, 105.55, 101.31, 62.77.

Synthesis of **7** from (3,4-dimethoxyphenyl)methanol and 3,4-dimethoxybenzaldehyde:

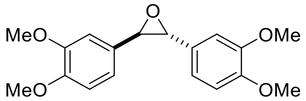

7

Scale: 10 mmol

Flash Chromatography: 4:1 Hex/DCM + 3 % TEA → 1:1 Hex/DCM + 1 % MeOH

TLC: R_f= 0.4 in 4:1 Hex/DCM, Stain: PMA (color: green)

% yield: 86% (2.72 g)

Physical state: white solid

d.r. 7:1

^{1}H NMR (500 MHz, Chloroform-*d*) δ 6.93 (dd, *J* = 8.2, 1.9 Hz, 2H), 6.87 (d, *J* = 8.2 Hz, 2H), 6.84 (d, *J* = 2.0 Hz, 2H), 3.90 (s, 6H), 3.89 (s, 6H), 3.81 (s, 2H).

^{13}C NMR (126 MHz, Chloroform-*d*) δ 149.49, 149.31, 129.82, 118.44, 111.30, 108.06, 62.92, 56.17, 56.07.

Synthesis of **s5** from (2,3,4-trimethoxyphenyl)methanol and 2,3,4-trimethoxybenzaldehyde:

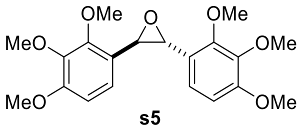

s5

Scale: 10 mmol

Flash Chromatography: Basic Dry Loading → 4:1 Hex/EtOAc + 3 % TEA → 4:1 Hex/EtOAc → 2:1 Hex/EtOAc

TLC: R_f = 0.3 in 4:1 Hex/EtOAc

% yield: 70% (2.6 g)

Physical state: white solid

d.r. 2:1

^{1}H NMR (500 MHz, Chloroform-*d*) δ 6.97 (d, *J* = 8.6 Hz, 1H), 6.70 (d, *J* = 8.7 Hz, 1H), 4.05 (s, 1H), 3.89 (s, 3H), 3.88 (s, 3H), 3.87 (s, 3H).

^{13}C NMR (126 MHz, Chloroform-*d*) δ 153.64, 152.77, 142.01, 123.49, 119.85, 107.62, 61.58, 61.06, 57.94, 56.19.

HRMS-ESI (m/z): calculated $C_{20}H_{24}O_7$ + Na^+: 399.1420 found 399.1418.

Note: Basic dry loading is performed by neutralizing 15 g of silica with 100 mL of DCM + 3 % TEA. The mixture is concentrated *in vacuo* and then the compound is introduced to the neutralized silica as a solution in DCM. The mixture is again concentrated *in vacuo* and dried under high vacuum prior to chromatography.

Synthesis of **6** from (4-methoxyphenyl)methanol and 4-methoxybenzaldehyde:

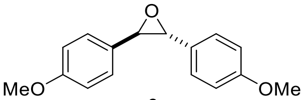

6

Scale: 10 mmol

Flash Chromatography: Basic Dry Loading → 20:1 Hex/EtOAc + 3 % TEA → 10:1 Hex/EtOAc

TLC: R_f = 0.6 in 10:1 Hex/EtOAc, Stain: PMA (color:green)

% yield: 100% (2.64 g)

Physical state: white solid (trans), yellow solid (mixed)

d.r. 3:1

^{1}H NMR (500 MHz, Chloroform-*d*) δ 7.27 (d, *J* = 8.7 Hz, 4H), 6.91 (d, *J* = 8.6 Hz, 4H), 3.82 (s, 6H), 3.81 (s, 2H).

^{13}C NMR (126 MHz, Chloroform-*d*) δ 159.87, 129.44, 126.91, 114.17, 62.68, 55.49.

Synthesis of **62** from (4-fluorophenyl)methanol and 4-fluorobenzaldehyde:

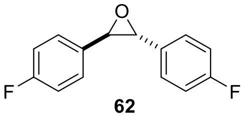

62

Scale: 10 mmol

Flash Chromatography: 20:1 Hex/EtOAc

TLC: R_f = 0.6 in 10:1 Hex/EtOAc, Stain: PMA (color:green)

% yield: 61% (1.43 g)

Physical state: white solid

d.r. >20:1

^{1}H NMR (500 MHz, Chloroform-*d*) δ 7.38 – 7.27 (m, 4H), 7.13 – 7.02 (m, 4H), 3.82 (s, 2H).

^{13}C NMR (126 MHz, Chloroform-*d*) δ 163.94, 161.97, 132.76, 132.74, 127.34, 127.28, 115.83, 115.66, 62.37.

General procedure for catalyzed [3+2] dipolar cycloaddition:

Photoredox reaction set-up: Reaction were irradiated with a Kessil A160WE Controllable LED Aquarium Light and a Chanzon High Power LED Chip 100W Royal Blue Plant Grow Light (440 nm-450 nm). Reaction temperature was controlled by using multiple fans and compressed air.

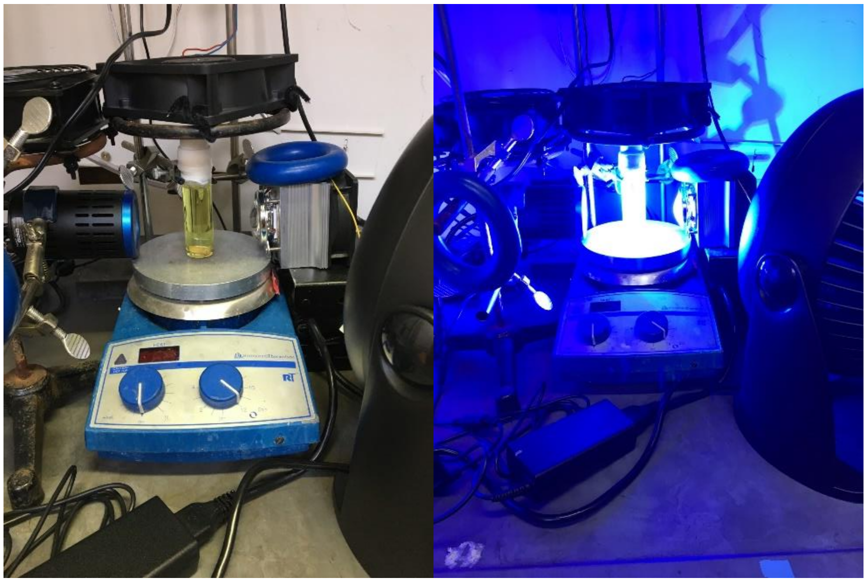

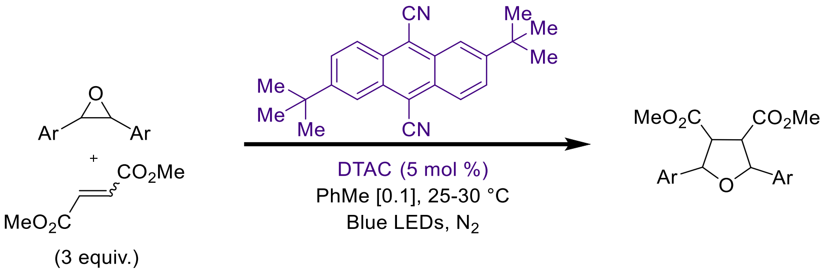

A flame dried 40-mL scintillation vial with a magnetic stirring bar was charged with epoxide (1 equiv.), dipolarophile (3 equiv.), DTAC (0.05 equiv), and PhMe [0.1 M]. The vial was sealed and sparged with N_2 for 15-20 minutes under sonication. The resultant mixture was placed under stirring and irradiated with blue LEDs until full consumption of the epoxide was observed by TLC. At the completion of the reaction, the resultant mixture is concentrated *in vacuo* and purified *via* flash chromatography.

^1H NMR spectra of the resulting mixture of diastereomeric tetrahydrofuran products are reported with a focus on the benzylic protons. These were used as diagnostic protons to measure conversion or observe disappearance of the tetrahydrofuran products for all subsequent reactions.

Synthesis of **s6** from **17** and dimethyl fumarate (**8**):

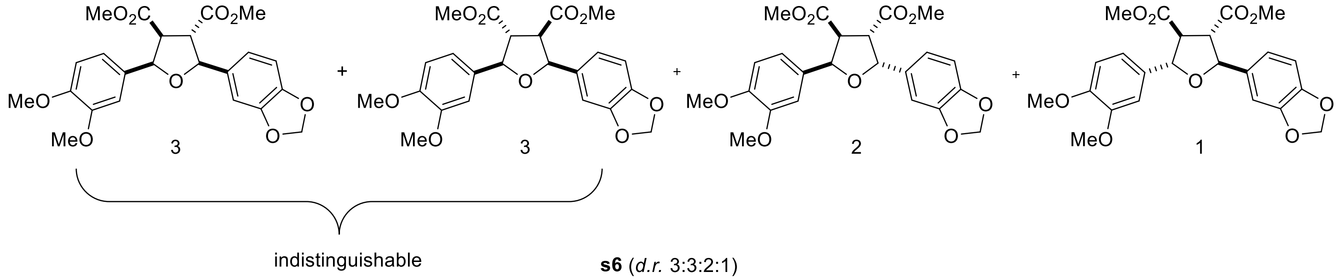

s6 (*d.r.* 3:3:2:1)
Chemical Formula: $C_{23}H_{24}O_9$
Exact Mass: 444.14

Reaction Time: 5 d

Scale: 3 mmol

Flash Chromatography: 10:1 → 4:1 → 1:1 Hex/EtOAc

TLC: R_f= 0.2 in 4:1 Hex/EtOAc, Stain: PMA (color: green)

% yield: 95% (1.27 g)

Physical state: yellow oil

d.r. 3:3:2:1

Major 1:

^{1}H NMR (500 MHz, Chloroform-*d*) δ 7.20 (d, *J* = 2.0 Hz, 1H), 7.11 (dd, *J* = 8.2, 2.0 Hz, 1H), 6.98 – 6.73 (m, 4H), 5.94 (s, 2H), 5.25 (d, *J* = 8.2 Hz, 1H), 4.99 (d, *J* = 8.6 Hz, 1H), 3.94 (s, 3H), 3.90 (s, 3H), 3.77 (dd, *J* = 8.2, 6.1 Hz, 1H), 3.70 (s, 3H), 3.68 (dd, *J* = 8.6, 6.1 Hz, 1H), 3.30 (s, 3H).

^{13}C NMR (126 MHz, Chloroform-*d*) δ 172.62, 171.69, 149.35, 149.27, 147.56, 147.48, 131.62, 131.19, 120.30, 119.65, 111.06, 110.23, 108.05, 107.27, 101.18, 83.70, 82.47, 56.06, 56.05, 54.76, 54.69, 52.50, 51.98.

Major 2:

^{1}H NMR (500 MHz, Chloroform-*d*) δ 7.18 (d, *J* = 1.7 Hz, 1H), 7.01 (dd, *J* = 8.0, 1.7 Hz, 1H), 6.97 – 6.74 (m, 4H), 5.94 (s, 2H), 5.26 (d, *J* = 8.2 Hz, 1H), 4.96 (d, *J* = 8.8 Hz, 1H), 3.89 (s, 3H), 3.87 (s, 3H), 3.76 (dd, *J* = 8.2, 6.0 Hz, 1H), 3.70 (s, 3H), 3.67 (dd, *J* = 8.8, 6.0 Hz, 1H), 3.24 (s, 3H).

^{13}C NMR (126 MHz, Chloroform-*d*) δ 172.56, 171.81, 148.96, 148.85, 148.74, 147.89, 133.09, 129.71, 120.92, 119.21, 110.79, 109.83, 108.26, 107.51, 101.26, 83.66, 82.56, 56.05, 56.01, 54.87, 54.72, 52.52, 51.98.

Middle:

^{1}H NMR (500 MHz, Chloroform-*d*) δ 7.05 – 6.69 (m, 6H), 5.98 (s, 2H), 5.82 (AB, 2H), 3.92 (AB, 2H), 3.88 (s, 3H), 3.87 (s, 3H), 3.39 (s, 3H), 3.34 (s, 3H).

^{13}C NMR (126 MHz, Chloroform-*d*) δ 170.83, 170.75, 148.76, 147.69, 147.31, 133.01, 131.43, 119.62, 118.58, 110.78, 109.32, 108.05, 106.74, 101.16, 82.66, 82.58, 55.97, 55.99, 52.24, 52.21, 51.95, 51.94.

Minor:

^{1}H NMR (500 MHz, Chloroform-*d*) δ 6.92 – 6.75 (m, 6H), 5.96 (s, 2H), 5.33 (AB, 2H), 3.90 (s, 3H), 3.88 (s, 3H), 3.72 (s, 3H), 3.71 (s, 3H), 3.60 (AB, 2H).

^{13}C NMR (126 MHz, Chloroform-*d*) δ 171.68, 171.67, 149.31, 149.11, 148.14, 147.64, 134.28, 132.48, 119.78, 118.68, 111.17, 109.22, 108.37, 106.63, 101.28, 83.34, 83.09, 56.81, 56.56, 56.06, 56.05, 52.64, 52.60.

HRMS-ESI (m/z): calculated $C_{23}H_{24}O_9 + Na^+$: 467.1318 found 467.1312.

Synthesis of **s7** from **17** and dimethyl maleate**:**

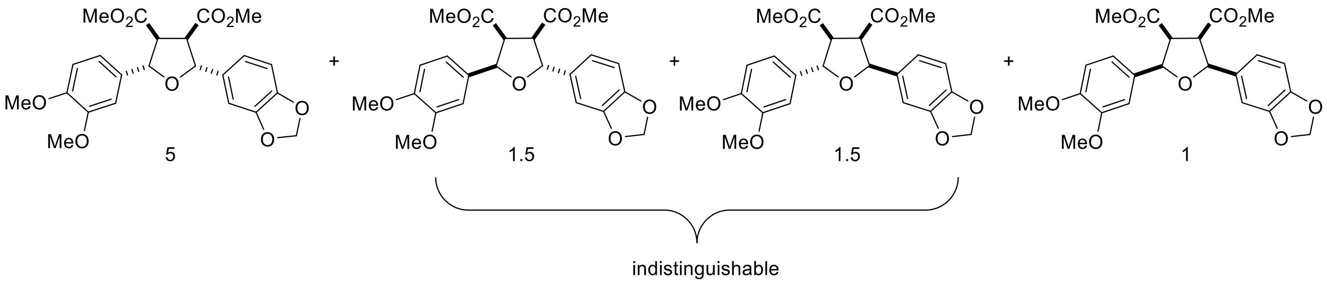

s7 (*d.r.* 5:1.5:1.5:1)
Chemical Formula: $C_{23}H_{24}O_9$
Exact Mass: 444.14

Reaction Time: 10 d

Scale: 3 mmol

Flash Chromatography: 10:1 → 4:1 → 1:1 Hex/EtOAc

TLC: R_f= 0.2 in 4:1 Hex/EtOAc, Stain: PMA (color: green)

% yield: 87% (1.16 g)

Physical state: yellow oil

d.r. 5:1.5:1.5:1

Major:

^{1}H NMR (500 MHz, Chloroform-*d*) δ 7.02 (dd, *J* = 8.2, 2.0 Hz, 1H), 6.98 (dd, *J* = 9.6, 1.9 Hz, 2H), 6.94 (dd, *J* = 8.0, 1.7 Hz, 1H), 6.87 (d, *J* = 8.2 Hz, 1H), 6.80 (d, *J* = 8.0 Hz, 1H), 5.96 (s, 2H), 5.28 (<u>A</u>B, *J* = 9.5, 7.0 Hz, 2H), 3.89 (s, 3H), 3.88 (s, 3H), 3.70 (s, 3H), 3.70 (s, 3H), 3.38 (A<u>B</u>, *J* = 7.7, 6.7 Hz, 2H).

^{13}C NMR (126 MHz, Chloroform-*d*) δ 171.39, 171.34, 149.15, 149.11, 148.03, 147.67, 133.72, 132.23, 120.20, 118.76, 111.28, 109.79, 108.38, 106.87, 101.27, 82.53, 82.51, 56.07, 56.04, 55.11, 55.00, 52.38, 52.36.

Middle 1 + Middle 2:

^{1}H NMR (500 MHz, Chloroform-*d*) δ 7.13 – 6.71 (overlap, 12H), 5.94 (overlap, 4 H), 5.88 – 5.80 (overlap, 2H), 5.58 – 5.49 (overlap, 2H), 3.90 (s, 3H), 3.87 (s, 9H), 3.75 – 3.71 (overlap, 2H), 3.68 (s, 3H), 3.67 (s, 3H), 3.48 (dd, *J* = 8.7, 7.2 Hz, 1H), 3.45 (dd, *J* = 8.5, 7.3 Hz, 1H), 3.37 (s, 3H), 3.31 (s, 3H).

^{13}C NMR (126 MHz, Chloroform-*d*) δ 170.90, 170.68, <u>170.62</u>, 149.15, 148.93, 148.86, 148.73, 147.98, 147.68, 147.40, 147.22, 136.75, 135.14, 131.52, 130.01, 119.65, 119.50, 118.57, 118.33, 111.28, 110.81, 109.51, 108.93, 108.36, 108.14, 106.68, 106.63, <u>101.16</u>, 83.03, 82.92, 81.19, 81.16, 56.25, 56.08, 56.06, 56.03, <u>56.01</u>, 54.99, 54.95, 52.37, 52.34, 51.82, 51.81.

Minor:

^{1}H NMR (500 MHz, Chloroform-*d*) δ 7.13 – 6.71 (overlap, 6H), 5.93 (s, 2H), 5.21 (<u>A</u>B, *J* = 12.8, 7.5 Hz, 2H), 3.91 (s, 3H), 3.88 (s, 3H), 3.75 (A<u>B</u>, *J* = 12.8, 7.5 Hz, 2H), 3.39 (s, 3H), 3.32 (s, 3H).

^{13}C NMR (126 MHz, Chloroform-*d*) δ 170.02, 169.95, 148.77, 148.71, 147.48, 147.24, 131.32, 129.98, 120.75, 119.86, 110.78, 110.55, 108.06, 107.81, 101.08, 81.48, 81.44, 56.09, 55.97, 52.65, 52.31, 51.63, 51.62.

HRMS-ESI (m/z): calculated $C_{23}H_{24}O_9$ + Na^+: 467.1318 found 467.1329.

Synthesis of **9** from **6** and dimethyl fumarate (**8**):

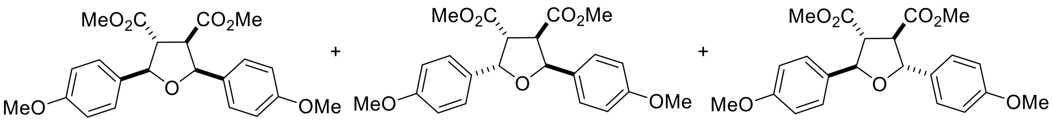

5 2.5 1

9 (*d.r.* 5:2.5:1)
Chemical Formula: $C_{22}H_{24}O_7$
Exact Mass: 400.15

Reaction Time: 2 d

Scale: 3 mmol

Flash Chromatography: 10:1 → 4:1 → 1:1 Hex/EtOAc

TLC: R_f= 0.2 in 4:1 Hex/EtOAc, Stain: PMA (color: green)

% yield: 93% (1.11 g)

Physical state: yellow oil

d.r. 5:2.5:1

Major:

^{1}H NMR (500 MHz, Chloroform-*d*) δ 7.53 (d, *J* = 8.7 Hz, 2H), 7.31 (d, *J* = 8.6 Hz, 2H), 6.94 (d, *J* = 8.7 Hz, 2H), 6.86 (d, *J* = 8.7 Hz, 2H), 5.30 (d, *J* = 8.4 Hz, 1H), 5.00 (d, *J* = 8.7 Hz, 1H), 3.82 (s, 3H), 3.79 (s, 3H), 3.78 (dd, 8.7, 6.6 Hz, 1H), 3.69 (dd, *J* = 8.4, 6.6 Hz, 1H), 3.69 (s, 3H), 3.21 (s, 3H).

^{13}C NMR (126 MHz, Chloroform-*d*) δ, 172.63, 170.90, 159.86, 159.44, 131.32, 129.50, 128.45, 127.94, 114.06, 113.67, 83.49, 82.32, 55.37, 55.36, 54.84, 54.79, 52.42, 51.84.

Middle:

^{1}H NMR (500 MHz, Chloroform-*d*) δ 7.25 (d, *J* = 8.5 Hz, 4H), 6.86 (d, *J* = 8.7 Hz, 4H), 5.84 (A̲B, *J* = 6.0, 1.7 Hz, 2H), 3.96 (AB̲, *J* = 6.0, 1.7 Hz, 2H), 3.79 (s, 6H), 3.33 (s, 6H).

^{13}C NMR (126 MHz, Chloroform-*d*) δ 171.84, 159.55, 131.28, 127.49, 113.54, 82.49, 55.41, 52.14, 51.86.

Minor:

^{1}H NMR (500 MHz, Chloroform-*d*) δ 7.37 (d, *J* = 8.6 Hz, 4H), 6.90 (d, *J* = 8.7 Hz, 4H), 5.34 (A̲B, *J* = 5.7, 2.5 Hz, 2H), 3.81 (6, 2H), 3.69 (s, 6H), 3.62 (AB̲, *J* = 5.7, 2.5 Hz, 2H).

^{13}C NMR (126 MHz, Chloroform-*d*) δ 171.76, 159.64, 132.33, 127.57, 114.11, 83.00, 56.66, 55.36, 52.53.

HRMS-ESI (m/z): calculated $C_{22}H_{24}O_7$ + Na^+: 423.1420 found 423.1413.

Synthesis of **s8** from **6** and dimethyl maleate**:**

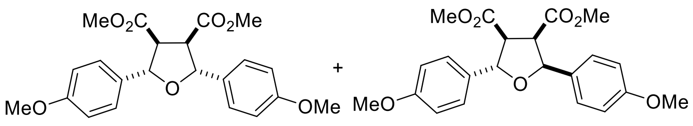

1.4 1

s8 (*d.r.* 1.4:1)
Chemical Formula: $C_{22}H_{24}O_7$
Exact Mass: 400.15

Reaction Time: 24 h

Scale: 1 mmol

Flash Chromatography: 4:1 Hex/EtOAc

TLC: R_f= 0.2 in 4:1 Hex/EtOAc, Stain: PMA (color: green)

% yield: 93% (371 mg)

Physical state: yellow oil

d.r. 1.4:1

Major:

^{1}H NMR (500 MHz, Chloroform-*d*) δ 7.40 (d, *J* = 8.5 Hz, 4H), 6.91 (d, *J* = 8.5 Hz, 4H), 5.30 (AB, *J* = 5.3, 2.2 Hz, 2H), 3.81 (s, 6H), 3.69 (s, 6H), 3.39 (AB, *J* = 5.3, 2.3 Hz, 2H).

^{13}C NMR (126 MHz, Chloroform-*d*) δ 171.34, 159.54, 131.80, 127.67, 113.94, 82.26, 55.27, 55.05, 52.15.

Minor:

^{1}H NMR (500 MHz, Chloroform-*d*) δ 7.45 (d, *J* = 8.7 Hz, 2H), 7.29 (d, *J* = 8.5 Hz, 2H), 6.90 (d, *J* = 8.5 Hz, 2H), 6.86 (d, *J* = 8.7 Hz, 2H), 5.85 (d, *J* = 8.8 Hz, 1H), 5.56 (d, *J* = 5.8 Hz, 1H), 3.80 (s, 3H), 3.79 (s, 3H), 3.73 (dd, *J* = 7.2, 5.8 Hz, 1H), 3.67 (s, 3H), 3.47 (dd, *J* = 8.8, 7.2 Hz, 1H), 3.30 (s, 3H).

^{13}C NMR (126 MHz, Chloroform-*d*) δ 170.87, 170.71, 159.48, 159.31, 134.86, 129.76, 127.50, 127.23, 114.01, 113.64, 82.83, 81.02, 56.19, 55.41, 55.36, 54.92, 52.28, 51.67.

HRMS-ESI (m/z): calculated $C_{22}H_{24}O_7$ + Na^+: 423.1420 found 423.1406.

Synthesis of **10** from **7** and dimethyl fumarate (**8**):

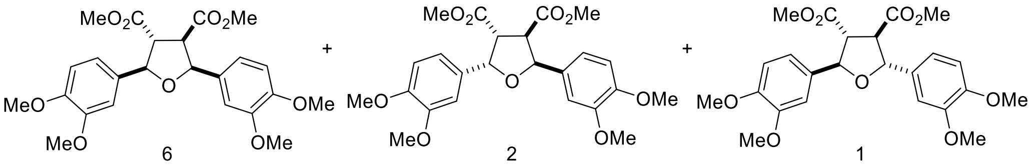

6 2 1

10 (*d.r.* 6:2:1)
Chemical Formula: $C_{24}H_{28}O_9$
Exact Mass: 460.17

Reaction Time: 10 d

Scale: 3 mmol

Flash Chromatography: 10:1 → 4:1 → 1:1 Hex/EtOAc

TLC: R_f= 0.2 in 4:1 Hex/EtOAc, Stain: PMA (color: green)

% yield: 99% (1.36 g)

Physical state: yellow oil

d.r. 6:2:1

Major:

^{1}H NMR (500 MHz, Chloroform-*d*) δ 7.23 (d, *J* = 2.0 Hz, 1H), 7.11 (dd, *J* = 8.2, 2.0 Hz, 1H), 7.02 – 6.78 (m, 4H), 5.28 (d, *J* = 8.2 Hz, 1H), 5.00 (d, *J* = 8.5 Hz, 1H), 3.93 (s, 3H), 3.89 (s, 3H), 3.87 (s, 3H), 3.86 (s, 3H), 3.78 (dd, *J* = 8.2, 6.0 Hz, 1H), 3.71 (dd, *J* = 8.5, 6.0 Hz, 1H), 3.70 (s, 3H), 3.23 (s, 3H).

^{13}C NMR (126 MHz, Chloroform-*d*) δ 172.53, 171.71, 149.16, 149.10, 148.78, 148.56, 131.66, 129.67, 119.47, 119.04, 110.89, 110.63, 110.09, 109.67, 83.52, 82.41, 55.89, 55.86, 55.82, 55.79, 54.63, 54.61, 52.34, 51.80.

Middle:

^{1}H NMR (500 MHz, Chloroform-*d*) δ 7.02 – 6.80 (overlap, 6H), 5.85 (<u>A</u>B, *J* = 6.0, 1.4 Hz, 2H), 3.91 (A<u>B</u>, *J* = 6.0, 1.4 Hz, 2H), 3.88 (s, 6H), 3.87 (s, 6H), 3.86 (s, 6H).

^{13}C NMR (126 MHz, Chloroform-*d*) δ 171.02, 148.83, 148.80, 131.49, 118.65, 110.86, 109.44, 82.75, 55.99, 55.92, 52.54, 51.93.

Minor:

^{1}H NMR (500 MHz, Chloroform-*d*) δ 7.05 – 6.76 (overlap, 6H), 5.35 (<u>A</u>B, *J* = 5.6, 2.5 Hz, 2H), 3.89 (s, 6H), 3.87 (s, 6H), 3.70 (s, 6H), 3.62 (A<u>B</u>, *J* = 5.6, 2.5 Hz, 2H).

^{13}C NMR (126 MHz, Chloroform-*d*) δ 171.76, 149.29, 149.07, 132.64, 119.47, 111.15, 109.21, 83.17, 56.55, 56.03, 55.95, 52.57.

HRMS-ESI (m/z): calculated $C_{24}H_{28}O_9$ + Na^+: 483.1613 found 483.1605.

Synthesis of **s9** from **7** and dimethyl maleate**:**

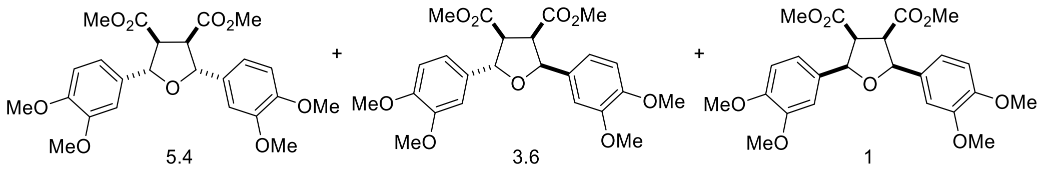

s9 (*d.r.* 5.4:3.6:1)
Chemical Formula: $C_{24}H_{28}O_9$
Exact Mass: 460.17

Reaction Time: 10 d

Scale: 1.5 mmol

Flash Chromatography: 10:1 → 4:1 → 1:1 Hex/EtOAc

TLC: R_f= 0.2 in 4:1 Hex/EtOAc, Stain: PMA (color: green)

% yield: 87% (612 mg)

Physical state: yellow oil

d.r. 5.4:3.6:1

Major:

^{1}H NMR (500 MHz, Chloroform-*d*) δ 7.15 – 6.77 (overlap, 6H), 5.33 (AB, *J* = 5.1, 2.2 Hz, 2H), 3.88 (s, 6H), 3.87 (s, 6H), 3.70 (s, 6H), 3.42 (AB, *J* = 5.1, 2.2 Hz, 2H).

^{13}C NMR (126 MHz, Chloroform-*d*) δ 171.45, 149.10, 149.05, 132.36, 118.71, 111.18, 109.75, 82.45, 56.05, 55.97, 54.92, 52.38.

Middle:

^{1}H NMR (500 MHz, Chloroform-*d*) δ 7.15 – 6.79 (overlap, 6H), 5.87 (d, *J* = 8.7 Hz, 1H), 5.56 (d, *J* = 5.6 Hz, 1H), 3.90 (s, 6H), 3.88 (s, 6H), 3.74 (dd, 5.6, 7.2 Hz, 1H), 3.67 (s, 3H), 3.48 (dd, *J* = 8.6, 7.2 Hz, 1H), 3.31 (s, 3H).

^{13}C NMR (126 MHz, Chloroform-*d*) δ 170.92, 170.68, 149.11, 148.89, 148.81, 148.68, 135.23, 130.09, 118.54, 118.31, 111.22, 110.74, 109.42, 108.88, 82.99, 81.18, 56.11, 56.04, 55.99, 55.00, 52.45, 51.62.

Minor:

^{1}H NMR (500 MHz, Chloroform-*d*) δ 7.14 – 6.78 (overlap, 6H), 5.23 (AB, *J* = 5.4, 1.9 Hz, 1H), 3.88 (s, 6H), 3.87 (s, 6H), 3.76 (AB, *J* = 5.4, 1.9 Hz, 1H), 3.32 (s, 6H).

^{13}C NMR (126 MHz, Chloroform-*d*) δ 170.05, 148.66, 148.63, 130.03, 119.76, 110.68, 110.46, 81.48, 56.01, 55.93, 52.35, 51.82.

HRMS-ESI (m/z): calculated $C_{24}H_{28}O_9 + Na^+$: 483.1613 found 483.1627.

Synthesis of **s10** from **s4** and dimethyl fumarate (**8**):

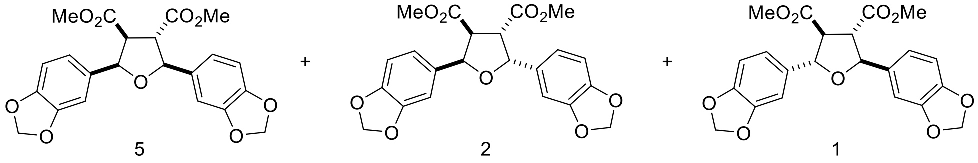

s10 (*d.r.* 5:2:1)
Chemical Formula: $C_{22}H_{20}O_9$
Exact Mass: 428.11

Reaction Time: 5 d

Scale: 3 mmol

Flash Chromatography: 10:1 → 4:1 → 1:1 Hex/EtOAc

TLC: R_f= 0.2 in 4:1 Hex/EtOAc, Stain: PMA (color: green)

% yield: 98% (1.26 g)

Physical state: yellow oil

d.r. 5:2:1

Major:

^{1}H NMR (500 MHz, Chloroform-*d*) δ 7.15 (d, *J* = 1.7 Hz, 1H), 7.00 (dd, *J* = 8.0, 1.7 Hz, 1H), 6.91 – 6.73 (m, 4H), 5.94 (s, 4H), 5.23 (d, *J* = 8.3 Hz, 1H), 4.94 (d, *J* = 8.6 Hz, 1H), 3.75 (dd, *J* = 8.3, 6.1 Hz, 1H), 3.70 (s, 3H), 3.64 (dd, *J* = 8.6, 6.1 Hz, 1H), 3.29 (s, 3H).

^{13}C NMR (126 MHz, Chloroform-*d*) δ 172.47, 171.61, 148.04, 147.87, 147.54, 147.46, 132.96, 131.05, 120.86, 120.26, 108.24, 108.03, 107.47, 107.26, 101.25, 101.17, 83.58, 82.40, 54.82, 54.67, 52.49, 51.99.

Middle:

^{1}H NMR (500 MHz, Chloroform-*d*) δ 6.92 – 6.70 (overlap, 6H), 5.97 (s, 4H), 5.79 (AB, *J* = 5.9, 1.7 Hz, 2H), 3.93 (AB, *J* = 5.9, 1.7 Hz, 2H), 3.40 (s, 6H)

^{13}C NMR (126 MHz, Chloroform-*d*) δ 170.60, 147.74, 147.37, 133.06, 119.64, 108.08, 106.76, 101.25, 83.19, 52.49, 51.96.

Minor:

^{1}H NMR (500 MHz, Chloroform-*d*) δ 6.91 – 6.74 (overlap, 6H), 5.96 (s, 4H), 5.30 (AB, *J* = 5.7, 2.6 Hz, 2H), 3.70 (s, 6H), 3.56 (AB, *J* = 5.7, 2.6 Hz, 2H).

^{13}C NMR (126 MHz, Chloroform-*d*) δ 171.51, 148.10, 147.63, 134.08, 119.77, 108.33, 106.59, 83.19, 56.75, 52.60, 51.97.

HRMS-ESI (m/z): calculated $C_{22}H_{20}O_9$ + Na^+: 451.1005 found 451.0998.

Synthesis of **s11** from **s5** and dimethyl fumarate (**8**):

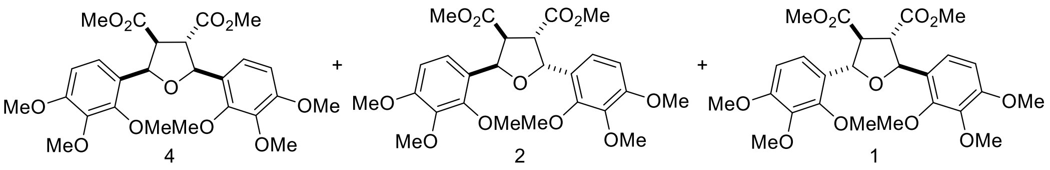

s11 (*d.r.* 4:2:1)
Chemical Formula: $C_{26}H_{32}O_{11}$
Exact Mass: 520.19

Reaction Time: 4 d

Scale: 3 mmol

Flash Chromatography: 4:1 → 1:1 Hex/EtOAc

TLC: R_f= 0.4 in 1:1 Hex/EtOAc, Stain: PMA (color: green)

% yield: 94% (1.47 g)

Physical state: yellow oil

d.r. 4:2:1

Major:

^{1}H NMR (500 MHz, Chloroform-*d*) δ 7.49 (d, *J* = 8.7 Hz, 1H), 7.15 (d, *J* = 8.7 Hz, 1H), 6.77 (d, *J* = 8.7 Hz, 1H), 6.62 (d, *J* = 8.7 Hz, 1H), 5.54 (d, *J* = 8.0 Hz, 1H), 5.35 (d, *J* = 8.5 Hz, 1H), 3.98 (s, 3H), 3.98 (s, 3H), 3.88 (s, 3H), 3.86 (s, 3H), 3.85 (s, 3H), 3.83 (s, 3H), 3.79 (dd, *J* = 8.0, 5.3 Hz, 1H), 3.69 (s, 3H), 3.63 (dd, *J* = 8.5, 5.3 Hz, 1H), 3.18 (s, 3H).

^{13}C NMR (126 MHz, Chloroform-*d*) δ 172.83, 172.08, 153.38, 153.23, 150.91, 150.79, 141.39, 141.25, 125.17, 123.22, 122.06, 120.72, 107.69, 106.67, 77.63, 77.42, 61.13, 60.88, 60.72, 60.68, 60.66, 55.96, 54.35, 53.80, 52.14, 51.53.

Middle:

^{1}H NMR (500 MHz, Chloroform-*d*) δ 7.11 (d, *J* = 8.7 Hz, 2H), 6.62 (d, *J* = 8.7 Hz, 2H), 6.09 (AB, *J* = 6.5, 1.7 Hz, 2H), 3.91 (AB, *J* = 6.5, 1.7 Hz, 2H), 3.88 (s, 6H), 3.86 (s, 6H), 3.83 (s, 6H), 3.25 (s, 6H).

^{13}C NMR (126 MHz, Chloroform-*d*) δ 171.65, 153.53, 151.62, 141.66, 124.83, 121.28, 106.63, 78.35, 60.79, 60.68, 60.66, 52.40, 51.48.

Minor:

^{1}H NMR (500 MHz, Chloroform-*d*) δ 7.20 (d, *J* = 8.7 Hz, 2H), 6.68 (d, *J* = 8.7 Hz, 2H), 5.56 (AB, *J* = 6.2, 2.7 Hz, 2H), 3.88 (s, 6H), 3.87 (s, 6H), 3.86 (s, 6H), 3.67 (s, 6H), 3.61 (AB, *J* = 6.2, 2.7 Hz, 2H).

^{13}C NMR (126 MHz, Chloroform-*d*) δ 171.77, 153.63, 151.56, 142.01, 125.90, 121.44, 107.11, 79.15, 60.88, 60.85, 60.72, 55.98, 52.19.

HRMS-ESI (m/z): calculated $C_{26}H_{32}O_{11}$ + Na^{+}: 543.1842 found 543.1828.

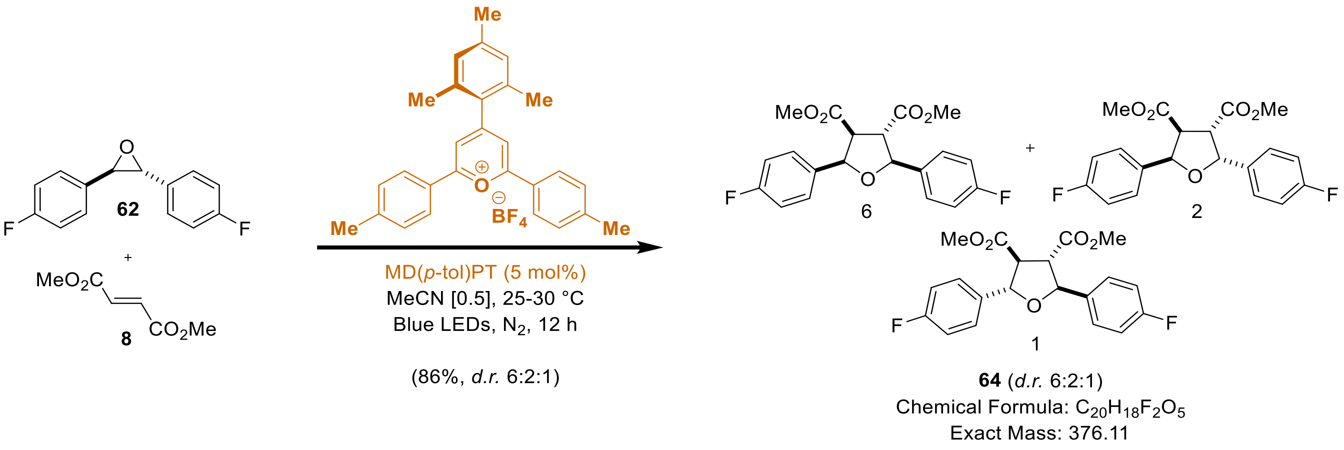

Synthesis of **64**: A flame dried 10-mL scintillation vial with a magnetic stirring bar was charged with **62** (697 mg, 3 mmol, 1 equiv.), **8** (519 mg, 3.6 mmol, 1.2 equiv.), and MD(*p*-tol)PT (70 mg, 0.15 mmol, 0.05 equiv.). The vial was sealed, placed under a N_2 atmosphere, and degassed MeCN (6 mL, [0.5 M]) was added via syringe. The resultant mixture was placed under stirring and irradiated with blue LEDs until full consumption of the epoxide was observed by TLC. At the completion of the reaction, the resultant mixture was concentrated *in vacuo* and purified *via* flash chromatography.

Reaction Time: 12 h

Scale: 3 mmol

Flash Chromatography: 4:1 → 1:1 Hex/EtOAc

TLC: R_f= 0.5 in 4:1 Hex/EtOAc, Stain: PMA (color: green)

% yield: 86% (970 mg)

Physical state: off-orange solid

d.r. 6:2:1

Major:

^{1}H NMR (500 MHz, Chloroform-*d*) δ 7.59 (dd, *J* = 8.5, 5.5 Hz, 2H), 7.37 (dd, *J* = 8.5, 5.5 Hz, 2H), 7.10 (t, *J* = 8.7 Hz, 2H), 7.04 (t, *J* = 8.6 Hz, 2H), 5.32 (d, *J* = 8.2 Hz, 1H), 5.05 (d, *J* = 8.5 Hz, 1H), 3.80 (dd, *J* = 8.3, 5.8 Hz, 1H), 3.71 (s, 3H), 3.67 (dd, *J* = 8.5, 5.8 Hz, 1H), 3.21 (s, 3H).

^{13}C NMR (126 MHz, Chloroform-*d*) δ 172.32, 171.62, 163.95, 163.69, 161.99, 161.72, 135.00, 134.98, 132.89, 132.86, 128.93, 128.86, 128.40, 128.34, 115.73, 115.56, 115.26, 115.09, 83.15, 82.17, 54.98, 54.62, 52.58, 51.90.

Middle:

^{1}H NMR (500 MHz, Chloroform-*d*) δ 7.32 (dd, *J* = 8.6, 5.5 Hz, 4H), 7.04 (dd, *J* = 8.6, 5.5 Hz, 4H), 5.90 (AB, *J* = 6.4 Hz, 2H), 3.92 (AB, *J* = 6.4 Hz, 2H), 3.30 (s, 6H).

^{13}C NMR (126 MHz, Chloroform-*d*) δ 170.76, 163.58, 161.62, 134.71, 134.69, 127.95, 127.88, 115.34, 115.17, 82.60, 52.62, 51.92.

Minor:

^{1}H NMR (500 MHz, Chloroform-*d*) δ 7.42 (dd, *J* = 8.5, 5.5 Hz, 4H), 7.04 (dd, *J* = 8.5, 5.5 Hz, 4H), 5.38 (AB, *J* = 5.7, 2.5 Hz, 2H), 3.71 (s, 6H), 3.60 (AB, *J* = 5.7, 2.5 Hz, 2H).

^{13}C NMR (126 MHz, Chloroform-*d*) δ 171.41, 163.74, 161.78, 135.94, 135.92, 127.94, 127.87, 115.78, 115.61, 82.76, 56.75, 52.69.

HRMS-ESI (m/z): calculated $C_{20}H_{18}F_2O_5$ + Na^+: 399.1020 found 399.1011.

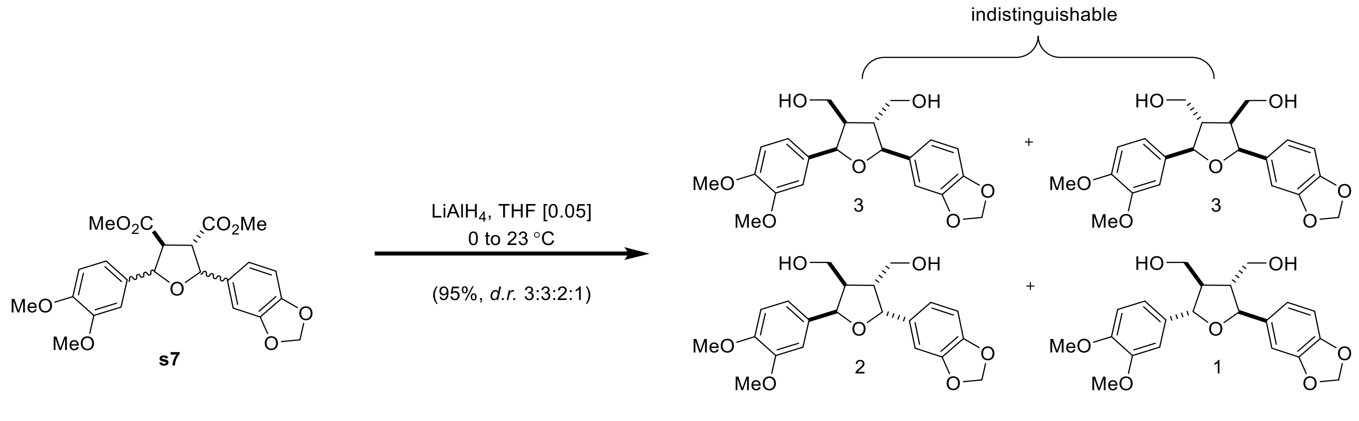

18
Chemical Formula: $C_{21}H_{24}O_7$
Exact Mass: 388.15

Synthesis of **18**: A 100-mL round bottom flask with a magnetic stirring bar was charged with compound **s7** (1.2 g, 2.7 mmol, 1 equiv.) and THF (54 mL, [0.05 M]). The stirred reaction was cooled to 0 °C (water/ice bath) and $LiAlH_4$ (615 mg, 16.2 mmol, 6 equiv.) was added in one portion. The reaction was left to warm to room temperature and after a 1 h stir period it was cooled to 0 °C and quenched by dropwise addition of acetone. Thereafter, saturated solution of Rochelle's salt (50 mL) was added and the reaction was left to stir until the gray solution became clear. The resultant mixture was introduced into a separatory funnel and extracted with Et_2O (3x50 mL). The combined organic fractions were dried over $MgSO_4$ and reduced *in vacuo*. The resultant material was dried under high vacuum and was used without any further purification.

Reaction Time: 1 h

Flash Chromatography: 1:1 → 4:1 EtOAc/Hex (optional)

TLC: R_f= 0.5 in 4:1 EtOAc/Hex, Stain: PMA (color: green)

% yield: 95% (996 mg)

Physical state*:* fluffy off-white solid (hygroscopic)

d.r. 3:3:2:1

Major 1 + Major 2:

^{1}H NMR (500 MHz, Chloroform-*d*) δ 7.06 – 6.67 (overlap, 12H), 5.94 (overlap, 4H), 5.08 (overlap, 2H), 4.70 (overlap, 2H), 4.45 (overlap, 3H), 3.87 (s, 6 H), 3.86 (s, 6 H), 3.70 (overlap, 2H), 3.54 (overlap, 2H), 3.30 (overlap, 2H), 3.09 (overlap, 2H), 2.60 – 2.47 (overlap, 2H), 2.32 – 2.15 (overlap, 2H).

^{13}C NMR (126 MHz, Chloroform-*d*) δ 149.21, 149.11, 148.90, 148.59, 148.09, 147.76, 147.61, 147.15, 134.07, 132.94, 132.48, 131.44, 120.52, 119.91, 119.23, 118.81, 111.28, 111.09, 110.15, 109.83, 108.29, 108.16, 107.11, 107.03, 101.23, 101.18, 82.64, 81.31, 81.27, 63.75, 63.01, 62.93, 56.05, 56.05, 56.02, 56.00, 55.34, 55.21, 51.04, 50.88.

Middle:

^{1}H NMR (500 MHz, Chloroform-*d*) δ 7.06 – 6.67 (overlap, 6H), 5.95 (s, 2H), 5.46 (dd, *J* = 7.1, 4.3 Hz, 2H), 3.89 (s, 3H), 3.86 (s, 3H), 3.46 (ddd, *J* = 11.1, 3.7, 2.4 Hz, 2H), 3.00 (td, *J* = 11.1, 8.2 Hz, 2H), 2.71 – 2.61 (m, 2H).

^{13}C NMR (126 MHz, Chloroform-*d*) δ 148.92, 148.55, 147.84, 147.08, 134.50, 132.99, 119.46, 118.48, 111.09, 109.53, 108.15, 106.79, 101.23, 82.76, 82.69, 62.72, 62.69, 56.06, 56.02, 49.26, 49.19.

Minor:

^{1}H NMR (500 MHz, Chloroform-*d*) δ 7.06 – 6.67 (overlap, 6H), 5.93 (s, 2H), 4.70 (t, *J* = 9.2 Hz, 2H), 3.87 (s, 3H), 3.87 (s, 3H), 3.74-3.67 (overlap, 2H), 3.60 – 3.53 (overlap, 2H), 2.32 – 2.15 (overlap, 2H).

^{13}C NMR (126 MHz, Chloroform-*d*) δ 149.28, 148.96, 147.84, 147.42, 135.75, 133.94, 119.98, 118.93, 111.11, 109.49, 108.18, 106.69, 101.19, 83.36, 83.23, 62.88, 62.84, 57.32, 56.97, 56.02, 56.00.

HRMS-ESI (m/z): calculated $C_{21}H_{24}O_7 + Na^+$: 411.1420 found 411.1429.

Screened Lewis and Brønsted Acids to Convert 18 to Methyl Piperitol (19)

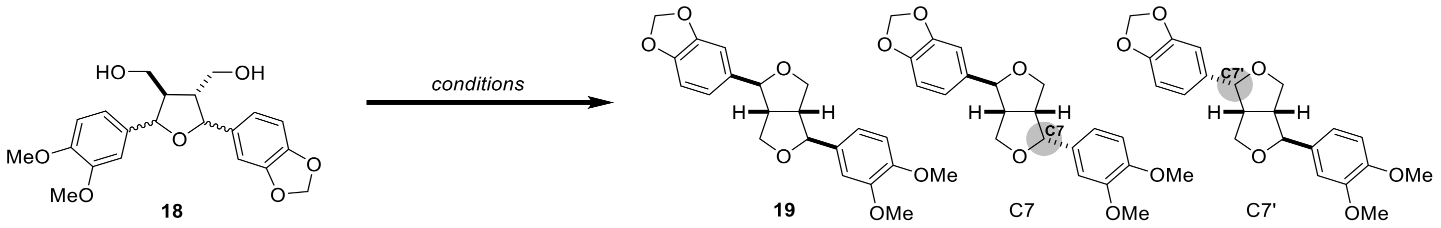

entry	*conditions*	*d.r.* (19:C7:C7')	% conversion
		***Best conditions (*23 °C)**	
**1**	$FeCl_3$ (20 mol%), DCM, 12 h	45 : 29 : 26	100
**2**	HCl (10 mol%), 4:1 DCM/HFIP, 12 h	59 : 21 : 19	100
**3**	TMSCl (5 equiv.), DCM, 3 d	67 : 28 : 01	72
**4**	[2 M] HCl (3 equiv.) in Et_2O, $CHCl_3$, 48 h	93 : 7 : 0	40
		***Lewis Acids:* (DCM [0.05 M], 12 h, 23 °C)**	
**5**	$AlCl_3$ (1 equiv.)	N/A	decomposition
**6**	$InCl_3$ (1 equiv.)	70 : 27 : 3	31
**7**	ZnI_2 (1 equiv.)	N/A	no reaction
**8**	$SnCl_4$ (1 equiv.)	N/A	trace/decomposition
**9**	$GaCl_3$ (1 equiv.)	65 : 30 : 5	100
**10**	BF_3-OEt_2 (1 equiv.)	50 : 25 : 25	100
		Brønsted Acids:	
**10**	TMSOTf (0.2 equiv.), DCM, 23 °C, 12 h	50 : 25 : 25	100

**11**	TMSOTf (0.2 equiv.), DCM, -40 °C, 12 h	50 : 25 : 25	10
**12**	H_2SO_4 (1 equiv.), DCM, 23 °C, 12 h	*N/A*	*polymerization*
**13**	TFA (1 equiv.), HFIP, 23 °C, 12 h	50 : 25 : 25	77

Other Brønsted (*p*-TsOH, TfOH, Tf_2NH, (±)-BPA) and Lewis acids ($Sc(OTf)_3$, $B(C_6F_5)_3$, BCl_3) that were screened did not yield superior results. Efforts to remove forming water with drying agents (molecular sieves, $MgSO_4$, CaO) were futile, as they inhibited reactivity.

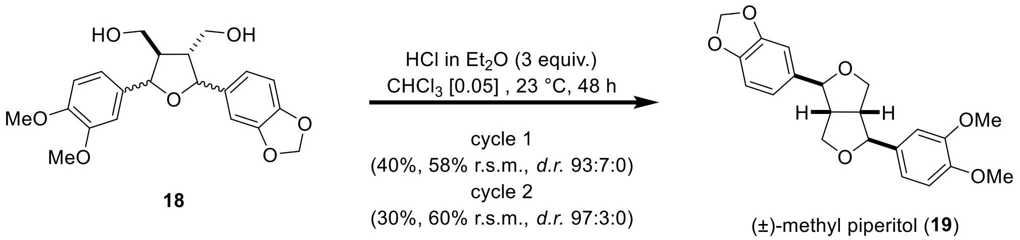

(±)-methyl piperitol (**19**)
Chemical Formula: $C_{21}H_{22}O_6$
Exact Mass: 370.14

Synthesis of (±)-methyl piperitol (**19**)[7]: A 10-mL scintillation vial with a magnetic stirring bar was charged with **18** (117 mg, 0.3 mmol, 1 equiv.) and $CHCl_3$ (6 mL, [0.05 M]). To the stirred reaction was added [2 M] HCl in Et_2O (450 µL, 0.9 mmol, 3 equiv.) dropwise, at which point the reaction turned from clear to light yellow. The reaction was left to stir for a 48 h period and quenched by dropwise addition of saturated $NaHCO_3$ (3 mL). The resultant solution was introduced into a separatory funnel with the assistance of DCM and the mixture was washed with saturated $NaHCO_3$, brine, and dried over $MgSO_4$. The resultant solution was reduced *in vacuo* and further purified through flash chromatography. For the additional cycle, the reaction was performed as described above with the recovered starting material.

Reaction Time: 48 h

Flash Chromatography: 1:1 Hex/Et_2O

TLC: R_f= 0.3 in 1:1 Hex/Et_2O, Stain: PMA (color: green)

% yield: [cycle 1] 40% (44 mg), 58% r.s.m. (68 mg) → **[cycle 2]** 30% (20 mg), 60% r.s.m. (41 mg)

Physical state: clear oil

d.r. >10:1 (cycle 1); >20:1 (cycle 2)

^{1}H NMR (500 MHz, Chloroform-*d*) δ 6.90 (d, *J* = 1.6 Hz, 1H), 6.89 – 6.83 (overlap, 3H), 6.81 (dd, *J* = 8.1, 1.3 Hz, 1H), 6.78 (d, *J* = 8.1 Hz, 1H), 5.95 (s, 2H), 4.74 (t, *J* = 5.1 Hz, 2H), 4.25 (dt, *J* = 9.0, 6.6 Hz, 2H), 3.90 (s, 3H), 3.89 – 3.85 (overlap, 2H), 3.88 (s, 3H), 3.09 (m, 2H).

^{13}C NMR (126 MHz, Chloroform-*d*) δ 149.30, 148.73, 148.09, 147.22, 135.19, 133.59, 119.49, 118.38, 111.11, 109.28, 108.32, 106.63, 101.21, 85.95, 85.89, 71.88, 71.80, 56.08, 56.04, 54.45, 54.28.

HRMS-ESI (m/z): calculated $C_{21}H_{22}O_6$ + Na^+: 393.1314 found 393.1327.

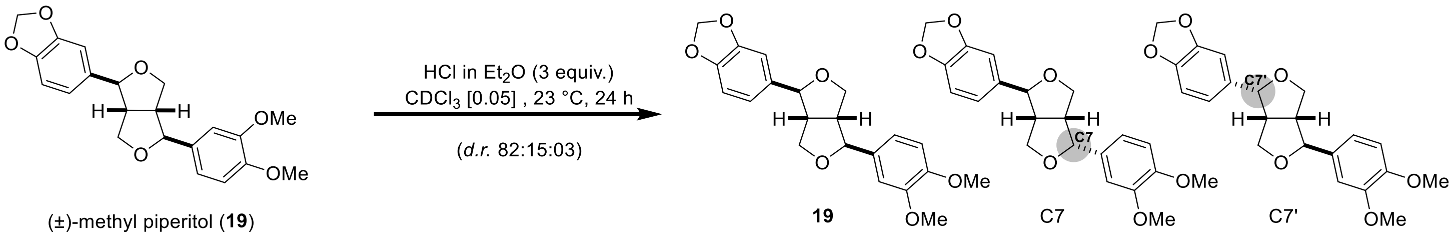

Epimerization study on (±)-methyl piperitol (**19**): A 1-mL scintillation vial with a magnetic stirring bar was charged with **19** (10 mg, 0.026 mmol, 1 equiv.) and dissolved in $CDCl_3$ (540 µL, [0.05 M]). To the stirred reaction was added [2 M] HCl in Et_2O (40 µL, 0.08 mmol, 3 equiv.) dropwise, at which point the reaction turned from clear to light yellow. The reaction was left to stir for 24 h and then transferred into an NMR tube. ^1H NMR analysis showed epimerization of **19** into its C7 and C7' epimer respectively (*d.r.* 100:0(C7):0(C7') → 82:15(C7):03(C7')).

General Procedure for the Synthesis of Dibenzylbutanes (CL1) from CL5a scaffolds:

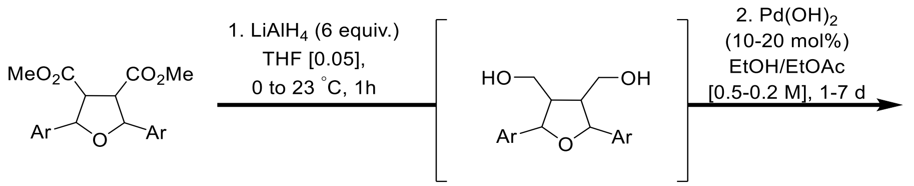

A 100-mL round bottom flask with a magnetic stirring bar was charged with diester (1 equiv.) and THF ([0.05 M]). The stirred reaction was cooled to 0 °C (ice/water bath) and $LiAlH_4$ (6 equiv.) was added in one portion. The reaction was left to warm to room temperature and stirred for 30 min. Once full consumption of diester was obtained by TLC (1:1 Hex/EtOAc), the reaction was cooled to 0 °C, diluted with Et_2O, and quenched by dropwise addition of acetone. Thereafter, saturated solution of Rochelle's salt was added and the reaction was left to stir until the gray solution turned clear. The resultant mixture was introduced into a separatory funnel and extracted with Et_2O (3x50 mL). The combined organic fractions were dried over $MgSO_4$ and reduced *in vacuo*. The resultant diol was used without further purification.

A 50-mL round bottom flask with a magnetic stirring bar was charged with diols, solvent (EtOH [0.5 M] or 1:1 EtOH/EtOAc [0.5-0.2 M]), and 20% wt. $Pd(OH)_2$ (10 mol %, assuming quantitative conversion). The reaction vessel was sealed, stirred, and adapted with an H_2 balloon every 12 h until full consumption of the diols was observed by TLC (3:1 EtOAc/Hex). At the completion of the reaction the mixture was passed through a celite pad with the assistance of EtOAc and the collected organic solution was concentrated *in vacuo* and further purified by flash chromatography. Faster reaction times can be obtained by running the reaction under pressurized conditions (400 psi), but the results presented here reflect the yields obtained using a balloon with H_2.

Synthesis of **24** from **9:**

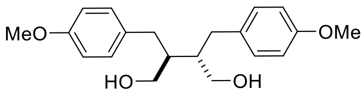

24
Chemical Formula: $C_{20}H_{26}O_4$
Exact Mass: 330.18

Conditions: 1:1 EtOH/EtOAc [0.2 M]

Scale: 2.32 mmol

Reaction Time: 1h + 48 h

Flash Chromatography: 1:1 Hex/EtOAc

TLC: R_f= 0.2 in 1:1 Hex/EtOAc, Stain: PMA (color: green)

% yield: 85 % (653 mg) 2-steps or 79% 3-steps

Physical state: white solid

^{1}H NMR (500 MHz, Chloroform-*d*) δ 7.07 (d, *J* = 8.6 Hz, 4H), 6.81 (d, *J* = 8.6 Hz, 4H), 3.83-3.74 (overlap, 8H), 3.49 (dd, *J* = 11.4, 4.2 Hz, 2H), 2.77 (dd, *J* = 13.8, 8.7 Hz, 2H), 2.66 (dd, *J* = 13.8, 5.7 Hz, 2H), 1.94 – 1.80 (m, 2H).

^{13}C NMR (126 MHz, Chloroform-*d*) δ 157.96, 132.73, 130.04, 113.91, 60.46, 55.39, 44.42, 35.37.

HRMS-ESI (m/z): calculated $C_{20}H_{26}O_4 + Na^+$: 353.1729 found 353.1725.

Synthesis of **25** from **s8**:

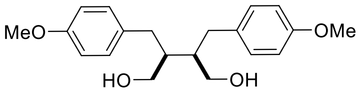

25
Chemical Formula: $C_{20}H_{26}O_4$
Exact Mass: 330.18

Conditions: EtOH [0.5 M]

Scale: 0.5 mmol

Reaction Time: 1h + 48 h

Flash Chromatography: 1:1 Hex/EtOAc

TLC: R_f= 0.2 in 1:1 Hex/EtOAc, Stain: PMA (color: green)

% yield: 48% (79 mg) 2-steps or 45% 3-steps

Physical state: clear oil

^{1}H NMR (500 MHz, Chloroform-*d*) δ 7.10 (d, *J* = 8.6 Hz, 4H), 6.83 (d, *J* = 8.7 Hz, 4H), 3.79 (s, 6H), 3.58 (dd, *J* = 11.1, 7.0 Hz, 2H), 3.49 (dd, *J* = 11.1, 2.9 Hz, 2H), 2.72 – 2.55 (m, 4H), 2.00 (td, *J* = 10.9, 9.6, 4.5 Hz, 2H).

^{13}C NMR (126 MHz, Chloroform-*d*) δ 158.04, 132.57, 130.07, 113.99, 63.27, 55.40, 45.47, 32.98.

HRMS-ESI (m/z): calculated $C_{20}H_{26}O_4 + Na^+$: 353.1729 found 353.1724.

Synthesis of (±)-secoisolariciresinol dimethyl ether (**26**)[8] from **10**:

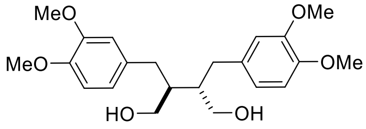

(±)-secoisolariciresinol dimethyl ether (**26**)
Chemical Formula: $C_{22}H_{30}O_6$
Exact Mass: 390.20

Conditions: EtOH [0.5 M]

Scale: 2.71 mmol

Reaction Time: 1h + 6d

Flash Chromatography: 1:1 Hex/EtOAc +1% MeOH→ 1:2 Hex/EtOAc + 1% MeOH

TLC: R_f= 0.2 in 1:1 Hex/EtOAc + 1% MeOH, Stain: PMA (color: green)

% yield: 58% (618 mg) 2-steps or 57 % 3-steps

Physical state: white solid

^{1}H NMR (500 MHz, Chloroform-*d*) δ 6.77 (d, *J* = 8.1 Hz, 2H), 6.68 (d, *J* = 8.1 Hz, 2H), 6.65 (s, 2H), 3.85 (s, 6H), 3.82 (s, 6H), 3.63 – 3.47 (m, 2H), 3.00 (brs, 2H), 2.78 (dd, *J* = 13.4, 8.3 Hz, 2H), 2.68 (dd, *J* = 13.4, 6.0 Hz, 2H), 1.99 – 1.79 (m, 2H).

¹³C NMR (126 MHz, Chloroform-*d*) δ 148.96, 147.41, 133.24, 121.11, 112.25, 111.23, 60.80, 56.04, 55.97, 44.06, 35.95.

HRMS-ESI (m/z): calculated $C_{22}H_{30}O_6$ + Na^+: 413.1940 found 413.1937.

Synthesis of **27** from **s9**:

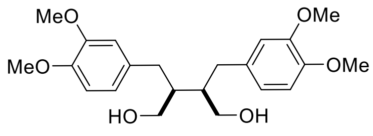

27
Chemical Formula: $C_{22}H_{30}O_6$
Exact Mass: 390.20

Conditions: EtOH [0.5 M]

Scale: 1.22 mmol

Reaction Time: 1h + 6d

Flash Chromatography: 1:1 Hex/EtOAc +1% MeOH→ 1:2 Hex/EtOAc + 1% MeOH

TLC: R_f= 0.2 in 1:1 Hex/EtOAc + 1% MeOH, Stain: PMA (color: green)

% yield: 40% (190 mg) 2-steps or 35% 3-steps

Physical state: clear oil

¹H NMR (500 MHz, Chloroform-*d*) δ 6.78 (d, *J* = 8.1 Hz, 2H), 6.73 (dd, *J* = 8.1, 1.9 Hz, 2H), 6.68 (d, *J* = 1.9 Hz, 2H), 3.85 (s, 6H), 3.84 (s, 6H), 3.61 (dd, *J* = 11.1, 6.9 Hz, 2H), 3.52 (dd, *J* = 11.0, 2.9 Hz, 2H), 3.16 (s, 2H), 2.68 (dd, *J* = 13.7, 9.2 Hz, 2H), 2.61 (dd, *J* = 13.7, 6.2 Hz, 2H), 2.11 – 1.97 (m, 2H).

¹³C NMR (126 MHz, Chloroform-*d*) δ 149.07, 147.52, 133.09, 121.10, 112.30, 111.32, 63.43, 56.06, 55.98, 45.15, 33.46.

HRMS-ESI (m/z): calculated $C_{22}H_{30}O_6$ + Na^+: 413.1940 found 413.1950.

Synthesis of (±)-dihydrocubenin (**22**)[9] from **s10**:

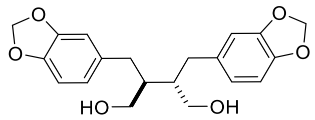

(±)-dihydrocubenin (**22**)
Chemical Formula: $C_{20}H_{22}O_6$
Exact Mass: 358.14

Conditions: 1:1 EtOH/EtOAc [0.5 M], Pd $(OH)_2$ (20 mol %)

Scale: 1.22 mmol

Reaction Time: 1h + 6d

Flash Chromatography: 1:1 Hex/EtOAc

TLC: R_f= 0.3 in 1:1 Hex/EtOAc, Stain: PMA (color: green)

% yield: 40% (405 mg) 2-steps or 39% 3-steps

Physical state: off-white solid

¹H NMR (500 MHz, Chloroform-*d*) δ 6.71 (d, *J* = 7.9 Hz, 2H), 6.64 (d, *J* = 1.4 Hz, 2H), 6.60 (dd, *J* = 7.9, 1.4 Hz, 2H), 5.92 (s, 4H), 3.78 (dd, *J* = 11.4, 1.9 Hz, 2H), 3.50 (dd, *J* = 11.4, 4.2 Hz, 2H), 3.07 (s, 2H), 2.75 (dd, *J* = 13.8, 8.6 Hz, 2H), 2.61 (dd, *J* = 13.8, 5.8 Hz, 2H), 1.91 – 1.78 (m, 2H).

¹³C NMR (126 MHz, Chloroform-*d*) δ 147.72, 145.86, 134.45, 121.99, 109.45, 108.25, 100.93, 60.37, 44.43, 36.06.

HRMS-ESI (m/z): calculated $C_{22}H_{30}O_6$ + H^+: 359.1495 found 359.1483.

Synthesis of **23** from **s11**:

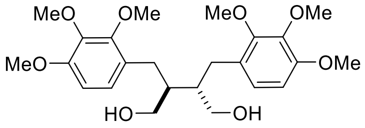

23
Chemical Formula: $C_{24}H_{34}O_8$
Exact Mass: 450.23

Conditions: 1:1 EtOH/EtOAc [0.2 M]

Scale: 2.81 mmol

Reaction Time: 1h + 2d

Flash Chromatography: 1:1 Hex/EtOAc + 1 % MeOH

TLC: R_f= 0.2 in 1:1 Hex/EtOAc + 1 % MeOH, Stain: PMA (color: green)

% yield: 52% (659 mg) 2-steps or 49% 3-steps

Physical state: clear oil

^{1}H NMR (500 MHz, Chloroform-*d*) δ 6.82 (d, *J* = 8.5 Hz, 2H), 6.61 (d, *J* = 8.5 Hz, 2H), 3.88 (s, 6H), 3.86 (s, 6H), 3.84 (s, 6H), 3.77 (d, *J* = 12.0 Hz, 2H), 3.42 (dd, *J* = 12.2, 4.0 Hz, 2H), 3.08 (bs, 2H), 2.79 (dd, *J* = 13.3, 9.8 Hz, 2H), 2.59 (dd, *J* = 13.4, 4.7 Hz, 2H), 1.90 – 1.71 (m, 2H).

^{13}C NMR (126 MHz, Chloroform-*d*) δ 152.26, 151.82, 142.18, 126.57, 125.02, 107.73, 61.33, 60.95, 60.01, 56.14, 45.14, 30.05.

HRMS-ESI (m/z): calculated $C_{24}H_{34}O_8$ + Na^+: 473.2151 found 473.2136.

Synthesis of (±)-dihydro-3',4'-dimethoxy-3'4'-demethylenedioxycubebin (**20**)[10] from **s7**:

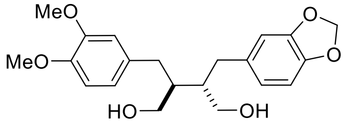

(±)-dihydro-3',4'-dimethoxy-3',4'-demethylenedioxycubebin (**20**)
Chemical Formula: $C_{21}H_{26}O_6$
Exact Mass: 374.17

Conditions: EtOH [0.5 M]

Scale: 2.85 mmol

Reaction Time: 1h + 6d or 48 h (400 psi)

Flash Chromatography: 4:1 Hex/EtOAc → 1:1 Hex/EtOAc

TLC: R_f= 0.2 in 4:1 Hex/EtOAc, Stain: PMA (color: green)

% yield: 50% (535 mg) 2-steps or 48% 3-steps

Physical state: clear oil

^{1}H NMR (500 MHz, Chloroform-*d*) δ 6.77 (d, *J* = 8.0 Hz, 1H), 6.72 – 6.66 (m, 2H), 6.64 (dd, *J* = 12.1, 1.5 Hz, 2H), 6.59 (dd, *J* = 8.0, 1.5 Hz, 1H), 5.94 – 5.88 (m, 2H), 3.85 (s, 3H), 3.83 (s, 3H), 3.79 (dt, *J* = 11.3, 2.5 Hz, 2H), 3.51 (dt, *J* = 11.3, 4.4 Hz, 2H), 3.46 (broad, 2H), 2.76 (dt, *J* = 13.7, 9.4 Hz, 2H), 2.64 (dt, *J* = 13.7, 11.0 Hz, 2H), 1.86 (m, 2H).
^{13}C NMR (126 MHz, Chloroform-*d*) δ 148.93, 147.69, 147.37, 145.82, 134.47, 133.19, 121.98, 121.12, 112.16, 111.21, 109.43, 108.20, 100.92, 60.53, 60.39, 56.01, 55.92, 44.23, 44.10, 36.03, 35.91.
HRMS-ESI (m/z): calculated $C_{21}H_{26}O_6$ + Na^+: 397.1627 found 397.1622.

Synthesis of (±)-2,3-desmethoxy-isolintetralin (**21**)[11] from **s8**:

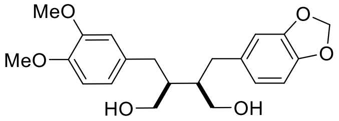

(±)-2,3-desmethoxy seco-isolintetralin (**21**)
Chemical Formula: $C_{21}H_{26}O_6$
Exact Mass: 374.17

Conditions: EtOH [0.5 M]

Scale: 2.47 mmol

Reaction Time: 1h + 6d

Flash Chromatography: 1:1 → 2:1 EtOAc/Hex
TLC: R_f= 0.3 in 2:1 EtOAc/Hex, Stain: PMA (color: green)

% yield: 41% (380 mg) 2-steps or 36% 3-steps

Physical state: clear oil

^{1}H NMR (500 MHz, Chloroform-*d*) δ 6.79 (d, *J* = 8.1 Hz, 1H), 6.72 (d, *J* = 7.8 Hz, 2H), 6.68 (s, 2H), 6.64 (d, *J* = 7.9 Hz, 1H), 5.92 (s, 2H), 3.85 (s, 3H), 3.85 (s, 3H), 3.59 (dt, *J* = 10.8, 7.5 Hz, 2H), 3.50 (d, *J* = 10.9 Hz, 2H), 2.91 (s, 2H), 2.66 (ddd, *J* = 13.3, 9.3, 4.0 Hz, 2H), 2.58 (dt, *J* = 13.2, 6.1 Hz, 2H), 2.08 – 1.95 (m, 2H).
^{13}C NMR (126 MHz, Chloroform-*d*) δ 149.01, 147.82, 147.46, 145.96, 134.29, 133.07, 122.02, 121.11, 112.16, 111.28, 109.43, 108.28, 100.98, 63.36, 63.28, 56.04, 55.93, 45.35, 45.21, 33.64, 33.36.
HRMS-ESI (m/z): calculated $C_{21}H_{26}O_6$ + Na^+: 397.1627 found 397.1626.

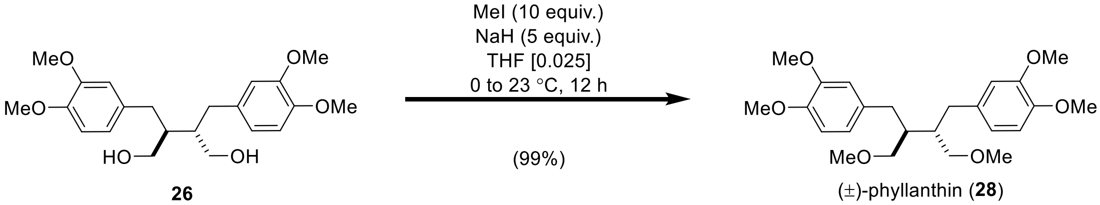

(±)-phyllanthin (**28**)
Chemical Formula: $C_{24}H_{34}O_6$
Exact Mass: 418.24

Synthesis of (±)-phyllanthin (**28**)[12]: A 10-mL scintillation vial with a magnetic stirring bar was charged with **26** (40 mg, 0.1 mmol, 1 equiv.) and THF (4 mL, [0.025 M]). The stirred reaction was cooled to 0 °C (ice/water bath) and NaH (22 mg, 0.5 mmol, 5 equiv.) was added in one portion. The reaction was stirred for 5 minutes and MeI (63 µL, 1 mmol, 10 equiv.) was added dropwise. The reaction was left to warm to room temperature (23 °C) and left to stir for a 12 h period. At the end of the stir period the reaction was quenched via dropwise addition of water until gas evolution seized. The resultant mixture was introduced into a separatory funnel with the assistance of Et_2O and further washed with water. The water layer was extracted twice with Et_2O (2x10 mL) and the combined organic layers were dried over $MgSO_4$. The organic layer was reduced *in vacuo* and further dried under high vacuum. The resultant solid was characterized without any further purification.

Reaction Time: 12 h

Flash Chromatography: 2:1 EtOAc/Hex (optional)

TLC: R_f= 0.2 in 2:1 Hex/EtOAc, Stain: PMA (color:green)

Physical state: off-white solid

% yield: 99 % (41 mg)

^{1}H NMR (500 MHz, Chloroform-*d*) δ 6.75 (d, *J* = 8.1 Hz, 2H), 6.64 (dd, *J* = 8.1, 2.0 Hz, 2H), 6.61 (d, *J* = 1.9 Hz, 2H), 3.85 (s, 6H), 3.80 (s, 6H), 3.40 – 3.22 (overlap, 10H), 2.65 (qd, *J* = 13.7, 7.1 Hz, 4H), 2.09 – 1.97 (m, 2H).

^{13}C NMR (126 MHz, Chloroform-*d*) δ 148.78, 147.18, 133.69, 121.16, 112.24, 111.03, 72.70, 58.86, 55.98, 55.83, 40.83, 35.05.

HRMS-ESI (m/z): calculated $C_{24}H_{34}O_6 + Na^+$: 441.2253 found 441.2268.

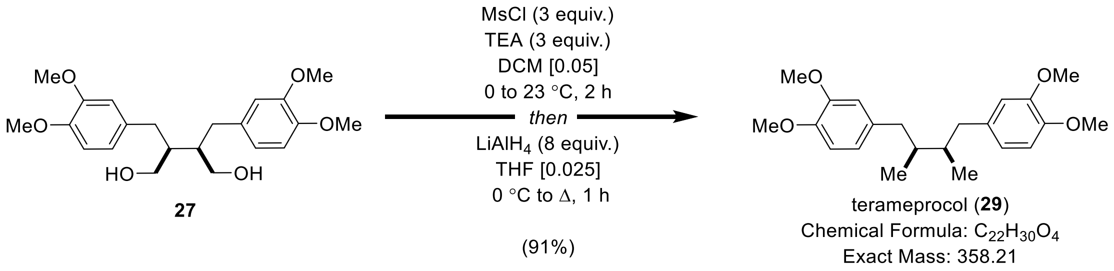

Synthesis of terameprocol (**29**)[13]: A 50-mL round bottom flask with a magnetic stirring bar was charged with **27** (140 mg, 0.36 mmol, 1 equiv.) and DCM (7.2 mL, [0.05 M]). The stirred reaction was cooled to 0 °C (ice/water bath) and triethylamine (TEA) (154 µL, 1.1 mmol, 3 equiv.) and methanesulfonyl chloride (MsCl) (86 µL, 1.1 mmol, 3 equiv.) were added dropwise. The reaction was stirred for 2 h or until TLC analysis (1:1 Hex/EtOAc) showed full consumption of **27**. The mixture was reduced *in vacuo* and re-dissolved in THF (14.4 mL, [0.025 M]). The resultant heterogeneous solution was cooled to 0 °C and $LiAlH_4$ (110 mg, 2.88 mmol, 8 equiv.) was added in small portions. The mixture was warmed to room temperature and refluxed in an oil bath. The reaction was refluxed for 1 h, cooled to room temperature, and then cooled to 0 °C. The reaction was quenched by dropwise addition of acetone. Thereafter, saturated solution of Rochelle's salt (30 mL) was added and the reaction was left to stir until the gray solution became clear. The reaction mixture was introduced into a separatory funnel and extracted thrice with Et_2O. The combined organic fractions were dried over $MgSO_4$ and reduced *in vacuo*. The resultant material was dried under high vacuum and was used without any further purification.

Reaction Time: 2 h + 1 h

Flash Chromatography: 4:1 Hex/EtOAc

TLC: R_f= 0.3 in 4:1 Hex/EtOAc, Stain: PMA (color:green)

Physical state: off-white solid

% yield: 91 % (117 mg)

^{1}H NMR (500 MHz, Chloroform-*d*) δ 6.78 (d, *J* = 8.1 Hz, 2H), 6.70 (dd, *J* = 8.1, 1.9 Hz, 2H), 6.65 (d, *J* = 1.9 Hz, 2H), 3.86 (s, 6H), 3.85 (s, 6H), 2.75 (dd, *J* = 13.4, 5.0 Hz, 2H), 2.30 (dd, *J* = 13.5, 9.3 Hz, 2H), 1.84 – 1.71 (m, 2H), 0.85 (d, *J* = 6.6 Hz, 6H).

^{13}C NMR (126 MHz, Chloroform-*d*) δ 148.86, 147.20, 134.60, 121.07, 112.40, 111.15, 56.05, 55.94, 39.33, 38.98, 16.37.

HRMS-ESI (m/z): calculated $C_{22}H_{30}O_4 + Na^+$: 381.2042 found 381.2047.

General Procedure for Conversion of Dibenzylbutanes (CL1) to Dibenzylbutyrolactones (CL2):[14]

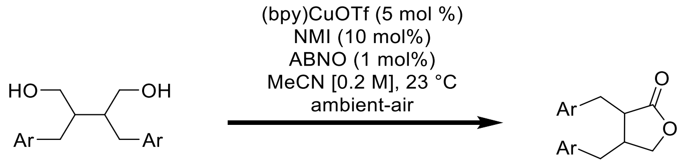

A 10-mL scintillation vial with a stirring bar was charged with $Cu(MeCN)_4OTf$ (0.05 equiv.), 2.2'-bpy (0.05 equiv.) and MeCN (1 part of 3, [0.2 M] total volume based on diol). This mixture was left to stir for 5 minutes to prepare the (bpy)CuOTf *in situ*. On a separate 10-mL vial with a stirring bar the diol (1 equiv.) was added and dissolved in MeCN (2 parts of 3). To this stirred solution, the (bpy)CuOTf solution prepared previously was added dropwise ([0.2 M] total). After a 5 minute stir period ABNO (0.01 equiv., from a 1 mg/mL solution in MeCN) and NMI (0.1 equiv.) were added to the reaction mixture in that order. The reaction which commenced as a burgundy solution turned green after a 12 h stirring period at which point it was reduced *in vacuo*. The resultant residue was dissolved in DCM and washed with water. The water layer was extracted twice with DCM and the combined organic fractions were dried over $MgSO_4$. The resultant solution was reduced *in vacuo* and further purified by flash chromatography.

Synthesis of **30** from **24**:

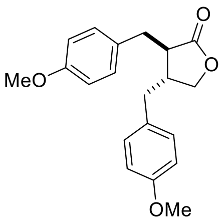

30
Chemical Formula: $C_{20}H_{22}O_4$
Exact Mass: 326.15

Conditions: 0.5 mL of DMF was added in addition to MeCN.

Scale: 0.3 mmol

Reaction Time: 12 h

Flash Chromatography: 4:1 Hex/EtOAc → 2:1 Hex/EtOAc

TLC: R_f= 0.2 in 4:1 Hex/EtOAc, Stain: PMA (color: green)

% yield: 94% (92 mg)

Physical state: clear oil

^{1}H NMR (500 MHz, Chloroform-*d*) δ 7.08 (d, *J* = 8.6 Hz, 2H), 6.92 (d, *J* = 8.6 Hz, 2H), 6.83 (d, *J* = 8.6 Hz, 2H), 6.80 (d, *J* = 8.6 Hz, 2H), 4.06 (dd, *J* = 9.1, 7.1 Hz, 1H), 3.84 (dd, *J* = 9.1, 7.6 Hz, 1H), 3.79 (s, 3H), 3.78 (s, 3H), 2.99 (dd, *J* = 14.2, 5.3 Hz, 1H), 2.91 (dd, *J* = 14.2, 6.8 Hz, 1H), 2.67 – 2.59 (m, 1H), 2.59 – 2.53 (m, 1H), 2.52 – 2.42 (m, 2H).

^{13}C NMR (126 MHz, Chloroform-*d*) δ 178.76, 158.64, 158.53, 130.44, 130.11, 129.80, 129.72, 114.24, 114.18, 71.30, 55.41, 55.39, 46.71, 41.40, 37.79, 34.23.

HRMS-ESI (m/z): calculated $C_{20}H_{22}O_4 + Na^+$: 349.1416 found 349.1431.

Synthesis of (±)-dimethyl matairesinol (**31**)[15] from **26**:

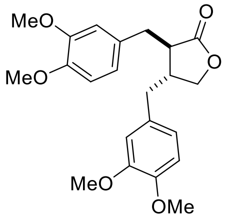

(±)-dimethyl matairesinol (**31**)
Chemical Formula: $C_{22}H_{26}O_6$
Exact Mass: 386.17

Scale: 0.3 mmol

Reaction Time: 12 h

Flash Chromatography: 1:1 Hex/EtOAc

TLC: R_f= 0.3 in 1:1 Hex/EtOAc, Stain: PMA (color: green)

% yield: 98% (114 mg)

Physical state: clear oil

^{1}H NMR (500 MHz, Chloroform-*d*) δ 6.76 (t, *J* = 8.3 Hz, 2H), 6.68 (d, *J* = 1.3 Hz, 1H), 6.65 (dd, *J* = 8.3, 1.3 Hz, 1H), 6.55 (dd, *J* = 8.3, 1.3 Hz, 1H), 6.48 (d, *J* = 1.3 Hz, 1H), 4.12 (dd, *J* = 8.9, 7.5 Hz, 1H), 3.88 (overlap, 1H), 3.85 (s, 3H), 3.85 (s, 3H), 3.83 (s, 3H), 3.82 (s, 3H), 3.01 – 2.88 (m, 2H), 2.72 – 2.55 (m, 2H), 2.55 – 2.43 (m, 2H).

^{13}C NMR (126 MHz, Chloroform-*d*) δ 178.84, 149.14, 149.11, 148.03, 147.95, 130.55, 130.29, 121.46, 120.67, 112.43, 111.91, 111.39, 111.15, 71.37, 56.03, 56.01, 55.98, 55.95, 46.69, 41.19, 38.31, 34.61.

HRMS-ESI (m/z): calculated $C_{22}H_{26}O_6 + Na^+$: 409.1627 found 409.1638.

Synthesis of (±)-hinokinin (**32**)[16] from **22**:

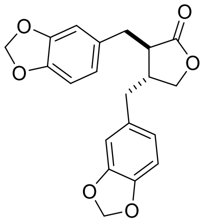

(±)-hinokinin (**32**)
Chemical Formula: $C_{20}H_{18}O_6$
Exact Mass: 354.11

Scale: 0.15 mmol

Reaction Time: 12 h

Flash Chromatography: 4:1 Hex/EtOAc

TLC: R_f= 0.3 in 4:1 Hex/EtOAc, Stain: PMA (color: green)

% yield: 85 % (45 mg)

Physical state: clear oil

^{1}H NMR (500 MHz, Chloroform-*d*) δ 6.73 (d, *J* = 7.8 Hz, 1H), 6.70 (d, *J* = 8.3 Hz, 1H), 6.63 (d, *J* = 1.7 Hz, 1H), 6.60 (dd, *J* = 7.8, 1.7 Hz, 1H), 6.49 – 6.44 (m, 2H), 5.97 – 5.90 (m, 4H), 4.13 (dd, *J* = 9.2, 6.8 Hz, 1H), 3.86 (dd, *J* = 9.2, 7.1 Hz, 1H),

2.98 (dd, J = 14.1, 5.1 Hz, 1H), 2.84 (dd, J = 14.1, 7.4 Hz, 1H), 2.64 – 2.56 (m, 1H), 2.56 – 2.50 (m, 1H), 2.50 – 2.40 (m, 2H).

^{13}C NMR (126 MHz, Chloroform-*d*) δ 178.54, 148.02, 148.00, 146.61, 146.48, 131.73, 131.46, 122.36, 121.68, 109.58, 108.95, 108.49, 108.42, 101.16, 71.28, 46.63, 41.43, 38.52, 34.98.

HRMS-ESI (m/z): calculated $C_{20}H_{18}O_6 + Na^+$: 377.1001 found 377.1004.

Synthesis of **33** from **23**:

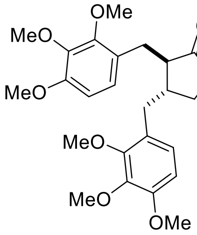

33
Chemical Formula: $C_{24}H_{30}O_8$
Exact Mass: 446.19

Scale: 0.825 mmol

Reaction Time: 12 h

Flash Chromatography: 4:1 Hex/EtOAc → 2:1 Hex/EtOAc

TLC: R_f= 0.1 in 4:1 Hex/EtOAc, Stain: PMA (color: green)

% yield: 84 % (309 mg)

Physical state: clear oil

^{1}H NMR (500 MHz, Chloroform-*d*) δ 6.89 (d, J = 8.5 Hz, 1H), 6.64 (d, J = 8.5 Hz, 1H), 6.60 (d, J = 8.5 Hz, 1H), 6.53 (d, J = 8.5 Hz, 1H), 4.09 (dd, J = 9.2, 7.3 Hz, 1H), 3.90 (s, 3H), 3.87 (overlap, 1 H), 3.86 (s, 3H), 3.83 (s, 3H), 3.82 (s, 6H), 3.77 (s, 3H), 3.17 (dd, J = 13.9, 5.3 Hz, 1H), 2.79 (dd, J = 13.9, 8.1 Hz, 1H), 2.64 (td, J = 8.3, 5.3 Hz, 1H), 2.58 (dd, J = 12.9, 4.9 Hz, 1H), 2.51 (dq, J = 12.9, 7.9 Hz, 1H), 2.42 (dd, J = 12.9, 8.9 Hz, 1H).

^{13}C NMR (126 MHz, Chloroform-*d*) δ 179.25, 152.89, 152.73, 152.26, 151.94, 142.29, 142.28, 125.04, 124.40, 124.26, 124.16, 107.35, 107.20, 71.42, 60.96, 60.91, 60.82, 56.10, 45.88, 41.73, 32.36, 29.73.

HRMS-ESI (m/z): calculated $C_{24}H_{30}O_8 + Na^+$: 469.1838 found 469.1820.

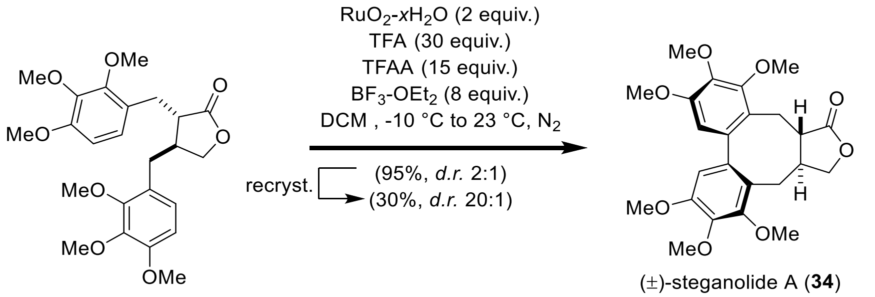

(±)-steganolide A (**34**)
Chemical Formula: $C_{24}H_{28}O_8$
Exact Mass: 444.18

Synthesis of (±)-steganolide A (**34**)[17]: A flame dried 50-mL round bottom flask with a stirring bar was charged with RuO_2-xH_2O (174 mg, 0.720 mmol, 2 equiv., 55.2 % Ru) and placed under a N_2 atmosphere. Thereafter, DCM (12 mL) was added *via* syringe and the stirred mixture was cooled to -10 °C (acetone/immersion cooler). TFA (823 µL, 10.74 mmol, 30 equiv.) and TFAA (1.13 mL, 5.37 mmol, 15 equiv.) were added in that order and the reaction was stirred for a 5 minute period. At the conclusion of the stir period, **33** (160 mg, 0.358 mmol, 1 equiv.) dissolved in DCM (6 mL) was added dropwise in concert with BF_3-OEt_2 (354 µL, 2.86 mmol, 8 equiv.). The reaction was left to cool to room temperature and stirred for a 24 h period. The resultant heterogeneous mixture was quenched via addition of saturated $NaHCO_3$ (20 mL) and the mixture was filtered through a celite pad. The resultant solution was extracted thrice with DCM and the combined organic layers were washed with brine and dried over $MgSO_4$. The solution was reduced *in vacuo* and the resultant residue was further purified via flash chromatography. Although reported as atroposelective, in our hands this reaction produced two atropisomers (*d.r.* 2:1). **34** can be obtained in high selectivity (*d.r.* 20:1) through sequential recrystallization with isopropyl alcohol.

Reaction Time: 26 h

Flash Chromatography: 20:1 DCM/EtOAc → 10:1 DCM/EtOAc

TLC: R_f= 0.7 in 10:1 DCM/EtOAc, Stain: PMA (color:green)

Recrystallization: isopropyl alcohol (3 times)

% yield: 95 % (151 mg, d.r. 2:1) → 30% (48 mg, d.r. 20:1)

Physical state: white solid

^{1}H NMR (500 MHz, Chloroform-*d*) δ 6.52 (s, 2H), 4.38 (dd, *J* = 8.4, 6.7 Hz, 1H), 3.95 (s, 3H), 3.93 (s, 3H), 3.92 (s, 3H), 3.92 (s, 3H), 3.85 (s, 6H), 3.79 (dd, *J* = 11.2, 8.4 Hz, 1H), 3.65 (d, *J* = 13.0 Hz, 1H), 3.13 (d, *J* = 13.0 Hz, 1H), 2.15 – 2.03 (m, 1H), 2.03 – 1.85 (m, 3H).

^{13}C NMR (126 MHz, Chloroform-*d*) δ 176.47, 151.62, 151.60, 150.72, 150.57, 142.09, 141.85, 136.23, 136.03, 126.60, 125.59, 109.62, 109.48, 70.24, 61.22, 61.11, 60.94, 56.20, 49.53, 46.66, 25.99, 24.26.

HRMS-ESI (m/z): calculated $C_{24}H_{28}O_8$ + H^+: 445.1862 found 445.1874.

General Procedure for Conversion of Dibenzylbutanes (CL1) to Furan (CL5b)[18]:

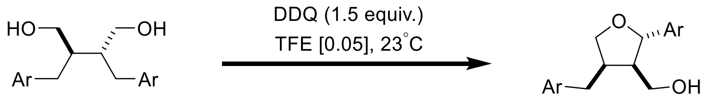

A 10-mL scintillation vial with a magnetic stirring bar was charged with diols (0.3 mmol, 1 equiv.) and dissolved in TFE (6 mL, [0.05 M]). To the stirred reaction was added 2,3-dichloro-5,6-dicyano-1,4-benzoquinone (DDQ) (103 mg, 0.045 mmol, 1.5 equiv.) in one portion at which point the reaction turned into a green color solution. The reaction was left to stir for a 6 h period or until the reaction was pink/white and heterogeneous indicating complete consumption of DDQ. The reaction was introduced into a separatory funnel and diluted with EtOAc (50 mL). The resultant mixture was washed with saturated $NaHSO_3$ (1x30 mL) and the aqueous layer was extracted twice with EtOAc. The combined organic layers were then washed with saturated $NaHCO_3$ (3x30 mL) and brine (1x30 mL). The organic layer was dried over Na_2SO_4 and reduced *in vacuo*. The resultant material was purified through flash chromatography or preparative TLC.

Synthesis of **35** from **24**:

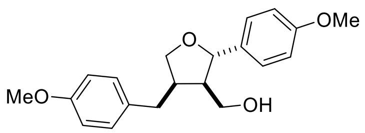

35
Chemical Formula: $C_{20}H_{24}O_4$
Exact Mass: 328.17

Reaction time: 6 h

Flash Chromatography: 1:1 Hex/EtOAc

TLC: R_f= 0.4 in 1:1 Hex/EtOAc, Stain: PMA (color:green)

Physical state: clear oil

% yield: 46 % (45 mg)

^{1}H NMR (500 MHz, Chloroform-*d*) δ 7.25 (d, *J* = 8.95 Hz, 2H), 7.11 (d, *J* = 8.5 Hz, 2H), 6.87 (d, *J* = 8.5 Hz, 2H), 6.84 (d, *J* = 8.5 Hz, 2H), 4.83 (d, *J* = 6.4 Hz, 1H), 4.06 (dd, *J* = 8.5, 6.6 Hz, 1H), 3.91 (dd, *J* = 10.8, 7.0 Hz, 1H), 3.80 (s, 3H), 3.79 (s, 3H), 3.78 – 3.75 (m, 1H), 3.75 – 3.71 (m, 1H), 2.91 (dd, *J* = 13.6, 5.4 Hz, 1H), 2.74 (m, 1H), 2.58 (dd, *J* = 13.6, 10.4 Hz, 1H), 2.39 (m, 1H).

^{13}C NMR (126 MHz, Chloroform-*d*) δ 159.14, 158.16, 135.19, 132.59, 129.68, 127.16, 114.12, 113.98, 82.82, 73.12, 61.14, 55.44, 55.40, 52.77, 42.52, 32.86.

HRMS-ESI (m/z): calculated $C_{20}H_{24}O_4 + H^+$: 351.1572 found 351.1587.

Synthesis of (±)-lariciresinol dimethyl ether (**36**)[19] from **26**:

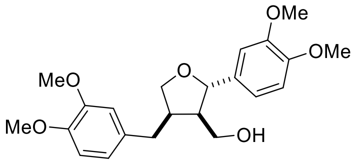

(±)-lariciresinol dimethyl ether (**36**)
Chemical Formula: $C_{22}H_{28}O_6$
Exact Mass: 388.19

Reaction time: 6 h

Flash Chromatography: 2:1 Hex/EtOAc

then **Preparative TLC:** 4:1 $CHCl_3$/EtOAc

TLC: R_f= 0.1 in 2:1 EtOAc/Hex, Stain: PMA (color:green)

Physical state: clear oil

% yield: 33% (39 mg)

^{1}H NMR (500 MHz, Chloroform-*d*) δ 6.90 – 6.85 (m, 2H), 6.84 – 6.78 (m, 2H), 6.77 – 6.69 (m, 2H), 4.82 (d, *J* = 6.5 Hz, 1H), 4.07 (dd, *J* = 8.6, 6.6 Hz, 1H), 3.93 (dd, *J* = 10.5, 7.1 Hz, 1H), 3.88 (s, 3H), 3.87 (s, 3H), 3.87 (s, 3H), 3.86 (s, 3H), 3.79 (dd, *J* = 10.5, 6.0 Hz, 1H), 3.75 (dd, 1H), 2.93 (dd, *J* = 13.6, 5.2 Hz, 1H), 2.75 (tt, *J* = 12.6, 6.3 Hz, 1H), 2.57 (dd, *J* = 13.6, 10.6 Hz, 1H), 2.43 (p, *J* = 6.9 Hz, 1H).

^{13}C NMR (126 MHz, Chloroform-*d*) δ 149.23, 149.10, 148.56, 147.60, 135.56, 133.09, 120.62, 118.16, 112.06, 111.46, 111.14, 109.09, 82.89, 73.09, 61.10, 56.08, 56.05, 56.03, 52.70, 42.49, 33.40.

HRMS-ESI (m/z): calculated $C_{22}H_{28}O_6 + Na^+$: 411.1784 found 411.1275.

Synthesis of (±)-dihydosesamin (**37**)[20] from **22**:

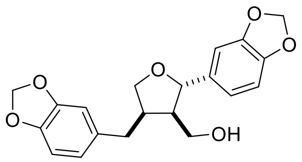

(±)-dihydrosesamin (**37**)
Chemical Formula: $C_{20}H_{20}O_6$
Exact Mass: 356.13

Reaction time: 6 h

Preparative TLC: 2:1 Hex/EtOAc

TLC: R_f= 0.2 in 2:1 Hex/EtOAc, Stain: PMA (color:green)

Physical state: clear oil

% yield: 32% (34 mg)

^{1}H NMR (500 MHz, Chloroform-*d*) δ 6.83 (s, 1H), 6.76 (s, 2H), 6.73 (d, *J* = 7.9 Hz, 1H), 6.68 (s, 1H), 6.64 (d, *J* = 7.9 Hz, 1H), 5.94 (s, 2H), 5.93 (s, 2H), 4.79 (d, *J* = 6.2 Hz, 1H), 4.08 – 4.01 (m, 1H), 3.89 (dd, *J* = 10.7, 6.9 Hz, 1H), 3.76 (dd, *J* = 10.7, 6.7 Hz, 1H), 3.74 – 3.69 (m, 1H), 2.87 (dd, *J* = 13.6, 5.4 Hz, 1H), 2.70 (tt, *J* = 12.3, 6.5 Hz, 1H), 2.54 (dd, *J* = 13.5, 10.5 Hz, 1H), 2.35 (p, *J* = 6.8 Hz, 1H).

^{13}C NMR (126 MHz, Chloroform-*d*) δ 147.98, 147.90, 147.02, 146.07, 137.22, 134.29, 121.56, 119.18, 109.07, 108.43, 108.20, 106.42, 101.13, 101.02, 83.01, 73.05, 61.05, 52.77, 42.47, 33.41.

HRMS-ESI (m/z): calculated $C_{20}H_{20}O_6$ + Na^+: 379.1158 found 379.1161.

Synthesis of (±)-sanshodiol methyl ether (**38**)[21] from **20**:

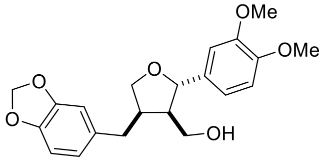

(±)-sanshodiol methyl ether (**38**)
Chemical Formula: $C_{21}H_{24}O_6$
Exact Mass: 372.16

Reaction time: 6 h

Flash Chromatography: 2:1 Hex/EtOAc → *then* **Preparative TLC:** 7:1 $CHCl_3$/EtOAc → *then* **Preparative TLC:** 2:1 Hex/EtOAc

TLC: R_f= 0.2 in 2:1 Hex/EtOAc, Stain: PMA (color:green)

Physical state: clear oil

% yield: 33% (36 mg)

r.r. 4:1

^{1}H NMR (500 MHz, Chloroform-*d*) δ 6.90 – 6.81 (m, 3H), 6.73 (d, *J* = 7.9 Hz, 1H), 6.68 (d, *J* = 1.6 Hz, 1H), 6.64 (dd, *J* = 7.9, 1.7 Hz, 1H), 5.93 (s, 2H), 4.81 (d, *J* = 6.3 Hz, 1H), 4.06 (dd, *J* = 8.6, 6.7 Hz, 1H), 3.91 (dd, *J* = 10.7, 6.9 Hz, 1H), 3.88 (s, 3H), 3.87 (s, 3H), 3.77 (dd, *J* = 10.7, 6.7 Hz, 1H), 3.73 (dd, *J* = 8.6, 6.5 Hz, 1H), 2.89 (dd, *J* = 13.6, 5.3 Hz, 1H), 2.76 – 2.67 (m, 1H), 2.55 (dd, *J* = 13.6, 10.5 Hz, 1H), 2.40 (p, *J* = 6.8 Hz, 1H).

^{13}C NMR (126 MHz, Chloroform-*d*) δ 149.24, 148.56, 147.91, 146.07, 135.58, 134.32, 121.56, 118.13, 111.15, 109.08, 108.43, 101.02, 82.97, 77.16, 73.03, 61.13, 56.09, 56.04, 52.55, 42.50, 33.50.

HRMS-ESI (m/z): calculated $C_{21}H_{24}O_6$ + Na^+: 395.1471 found 395.1482.

General Procedure for Conversion of Dibenzylbutanes (CL1) to Furan (CL5c)[22]:

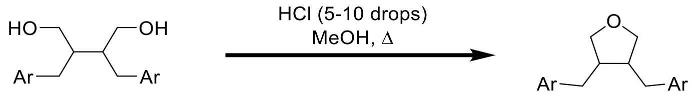

A 100-mL round bottom flask with a magnetic stirring bar was charged with diol (0.3 mmol. 1 equiv.), MeOH (30 mL, [0.01 M]) and HCl (5-10 drops, 12 M). The flask was adapted with a reflux condenser, placed under stirring, and refluxed until full consumption of the starting material was observed by TLC (1:1 Hex/EtOAc). The reaction was reduced *in vacuo* and the resultant residue was introduced into a separatory funnel with the assistance of EtOAc. The resultant organic layer was washed with water (1x 30 mL) and the aqueous layer was extracted with EtOAc (2 x 20 mL). The combined organic layers were washed with saturated $NaHCO_3$ (1 x 30 mL) and water (1 x 30 mL). The resultant organic mixture was then dried over Na_2SO_4 and reduced *in vacuo*. The resultant residue was further purified *via* flash chromatography.

Synthesis of **39** from **20**:

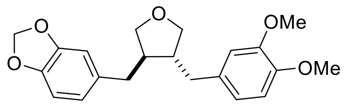

39
Chemical Formula: $C_{21}H_{24}O_5$
Exact Mass: 356.16

Reaction time: 4 d

Flash Chromatography: 4:1 Hex/EtOAc

TLC: R_f= 0.2 in 4:1 Hex/EtOAc, Stain: PMA (color: green)

Physical state: clear oil

% yield: 75% (80 mg)

^{1}H NMR (500 MHz, Chloroform-*d*) δ 6.76 (d, *J* = 8.1 Hz, 1H), 6.72 – 6.67 (m, 1H), 6.64 (dd, *J* = 8.1, 2.0 Hz, 1H), 6.57 (d, *J* = 1.9 Hz, 1H), 6.53 (dd, *J* = 5.9, 1.7 Hz, 2H), 5.98 – 5.83 (m, 2H), 3.90 (ddd, *J* = 8.9, 6.7, 3.0 Hz, 2H), 3.86 (s, 3H), 3.84 (s, 3H), 3.55 – 3.49 (m, 2H), 2.59 (ddd, *J* = 13.6, 10.5, 6.3 Hz, 2H), 2.51 (dt, *J* = 13.7, 7.9 Hz, 2H), 2.24 – 2.10 (m, 2H).

^{13}C NMR (126 MHz, Chloroform-*d*) δ 148.93, 147.74, 147.50, 145.93, 134.27, 133.05, 121.60, 120.68, 111.89, 111.20, 109.08, 108.16, 100.99, 73.45, 73.39, 56.00, 55.87, 46.69, 46.63, 39.30, 39.22.

HRMS-ESI (m/z): calculated $C_{21}H_{24}O_5$ + Na^+: 379.1521 found 379.1521.

Synthesis of **40** from **21**:

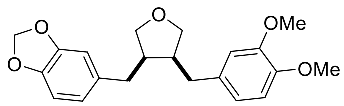

40
Chemical Formula: $C_{21}H_{24}O_5$
Exact Mass: 356.16

Reaction time: 7 d

Flash Chromatography: 4:1 Hex/EtOAc

TLC: R_f= 0.2 in 4:1 Hex/EtOAc, Stain: PMA (color: green)

Physical state: clear oil

% yield: 41% (44 mg), 93% b.r.s.m. (63 mg)

^{1}H NMR (500 MHz, Chloroform-*d*) δ 6.80 (d, *J* = 8.1 Hz, 1H), 6.75 – 6.70 (m, 2H), 6.68 (d, *J* = 1.8 Hz, 1H), 6.66 (d, *J* = 1.4 Hz, 1H), 6.63 (dd, *J* = 7.9, 1.4 Hz, 1H), 5.93 (s, 2H), 3.87 (s, 3H), 3.86 (s, 3H), 3.78 (ddd, *J* = 8.7, 5.7, 3.4 Hz, 2H), 3.62 (dt, *J* = 10.1, 5.0 Hz, 2H), 2.90 – 2.77 (m, 2H), 2.63 – 2.47 (m, 4H).

^{13}C NMR (126 MHz, Chloroform-*d*) δ 149.06, 147.85, 147.52, 145.97, 134.53, 133.30, 121.65, 120.67, 112.09, 111.43, 109.14, 108.37, 100.99, 72.19, 72.10, 56.06, 56.00, 43.90, 43.84, 33.43, 33.21.

HRMS-ESI (m/z): calculated $C_{21}H_{24}O_5 + Na^+$: 379.1521 found 379.1512.

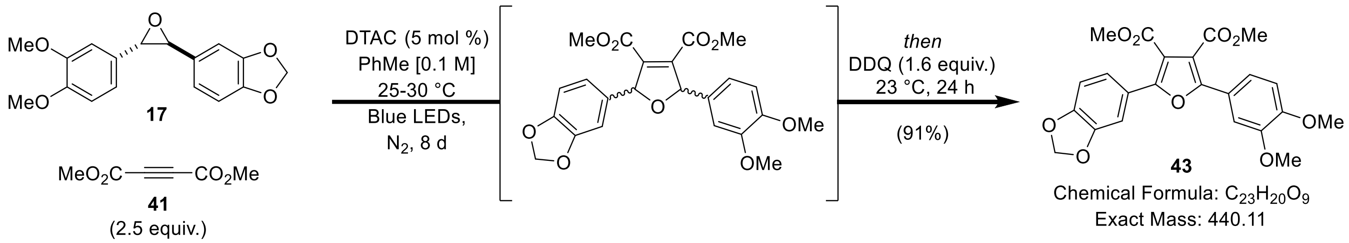

Synthesis of **43**: A 40-mL scintillation vial with a magnetic stirring bar was charged with **17** (901 mg, 3 mmol, 3 equiv.), DMAD (**41**) (920 µL, 7.5 mmol, 2.5 equiv.), DTAC (51 mg, 0.15 mmol, 0.05 equiv.) and PhMe (30 mL, [0.1 M]). Then, the vial was sealed and degassed with N_2 for a 15 minute period with sonication. The resultant mixture was irradiated with Blue LEDs for 8 days or until full consumption of **17** was observed by TLC (4:1 Hex/EtOAc). At the completion of the cycloaddition, irradiation was stopped, and DDQ (1.135 g, 5 mmol, 1.66 equiv.) was added in one portion. The reaction was left to stir for a 24 h period. The resultant material was passed through a basic alumina pad and washed with several portions of DCM (300 mL total). The resultant organic layer was concentrated *in vacuo* and purified by flash chromatography.

Reaction time: 8 d +1 d

Flash Chromatography: 4:1 Hex/EtOAc → 1:1 Hex/EtOAc

TLC: R_f= 0.2 in 4:1 Hex/EtOAc, Stain: PMA (color:green), UV-active: blue

Physical state: orange oil

% yield: 91 % (1.2 g)

^{1}H NMR (500 MHz, Chloroform-*d*) δ 7.49 (d, *J* = 2.0 Hz, 1H), 7.46 (dd, *J* = 8.4, 2.0 Hz, 1H), 7.41 (dd, *J* = 8.2, 1.7 Hz, 1H), 7.35 (d, *J* = 1.7 Hz, 1H), 6.93 (d, *J* = 8.5 Hz, 1H), 6.88 (d, *J* = 8.2 Hz, 1H), 6.02 (s, 2H), 3.94 (s, 3H), 3.93 (s, 3H), 3.87 (s, 3H), 3.86 (s, 3H).

^{13}C NMR (126 MHz, Chloroform-*d*) δ 164.49, 164.48, 153.43, 152.90, 150.36, 148.85, 147.91, 122.91, 122.22, 121.68, 120.80, 114.45, 114.18, 111.03, 110.73, 110.13, 108.58, 107.90, 101.61, 56.12, 56.08, 52.50, 52.45.

HRMS-ESI (m/z): calculated $C_{23}H_{20}O_9 + H^+$: 441.1186 found 441.1179.

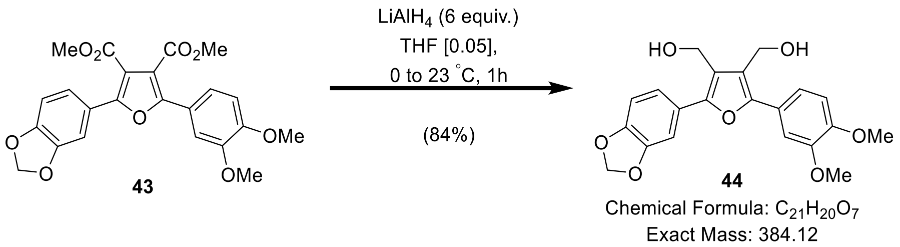

Synthesis of **44**: A 250-mL round bottom flask with a magnetic stirring bar was charged with **43** (700 mg, 1.6 mmol, 1 equiv.) and THF (32 mL, [0.05 M]). The reaction was cooled to 0 °C (ice/water bath) and $LiAlH_4$ (365 mg, 9.6 mmol, 6 equiv.) was added in one portion. The reaction was left to stir for 30 min or until full consumption of the starting material was observed by TLC (1:1 Hex/EtOAc). Then, the reaction was cooled to 0 °C, diluted with Et_2O (40 mL), and acetone was added dropwise until no further gas evolution was observed. Thereafter, saturated solution of Rochelle's salt (50 mL) was added and the reaction was left to stir until the gray solution became clear. The resultant mixture was introduced into a separatory funnel and extracted thrice with Et_2O. The combined organic fractions were dried over $MgSO_4$ and reduced *in vacuo*. The resultant white solid was used without any further purification.

Reaction time: 1 h

Flash Chromatography: 4:1 Hex/EtOAc → 1:1 Hex/EtOAc (optional)

TLC: R_f= 0.2 in 4:1 Hex/EtOAc, Stain: PMA (color: green), UV-active: blue

Physical state: white solid

% yield: 84 % (512 mg)

^{1}H NMR (500 MHz, Chloroform-*d*) δ 7.23 – 7.17 (m, 2H), 7.17 – 7.10 (m, 2H), 6.92 (d, *J* = 8.9 Hz, 1H), 6.88 (d, *J* = 7.9 Hz, 1H), 6.00 (s, 2H), 4.77 (s, 2H), 4.76 (s, 2H), 3.93 (s, 3H), 3.92 (s, 3H), 2.74 (brs, 2H).

^{13}C NMR (126 MHz, Chloroform-*d*) δ 150.13, 149.81, 149.28, 148.14, 147.72, 124.67, 123.49, 121.01, 120.91, 120.81, 119.73, 111.41, 110.15, 110.11, 108.77, 107.42, 101.43, 56.14, 56.12, 55.94, 55.88.

HRMS-ESI (m/z): calculated $C_{21}H_{20}O_7 + Na^+$: 407.1107 found 407.1105.

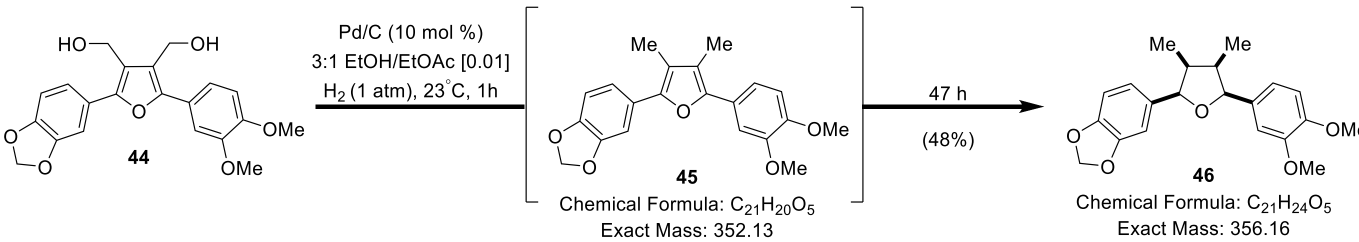

Synthesis of **46**: A 50-mL round bottom flask with a magnetic stirring bar was charged with **44** (116 mg, 0.3 mmol, 1 equiv.), 3:1 EtOH/EtOAc (24 mL, [0.0125 M]), and Pd/C (32 mg, 0.03 mmol, 0.1 equiv.). The reaction vessel was sealed, stirred, and adapted with an H_2 balloon every 12 h until full consumption of the diols was observed by TLC (4:1 EtOAc/Hex). TLC analysis revealed complete conversion of **44** to **45** within 1 h of stirring. On a separate reaction compound **45** was isolated and characterized to aid in the monitoring of the reaction. After 48 h, complete consumption of **45** can be observed and the reaction was stopped and passed through a celite pad with the assistance of EtOAc. The collected organic solution was concentrated *in vacuo* and further purified by flash chromatography.
For **45**

Reaction time: 1 h

Flash Chromatography: 4:1 Hex/EtOAc

TLC: R_f= 0.2 in 4:1 Hex/EtOAc, Stain: PMA (color: green), UV-active: blue

Physical state: off-white solid

% yield: 90% (95 mg)

^{1}H NMR (500 MHz, Chloroform-*d*) δ 7.23 – 7.17 (m, 3H), 7.15 (dd, *J* = 8.1, 1.7 Hz, 1H), 6.93 (d, *J* = 8.1 Hz, 1H), 6.88 (d, *J* = 8.1 Hz, 1H), 5.99 (s, 2H), 3.95 (s, 3H), 3.92 (s, 3H), 2.21 (s, 3H), 2.19 (s, 3H).

^{13}C NMR (126 MHz, Chloroform-*d*) δ 149.11, 148.16, 147.92, 147.04, 146.88, 146.46, 126.39, 125.19, 119.57, 118.48, 118.10, 117.96, 111.36, 109.17, 108.63, 106.43, 101.19, 56.09, 56.08, 10.07, 10.05.

HRMS-ESI (m/z): calculated $C_{21}H_{20}O_5$ + H^+: 353.1389 found 353.1386.

For **46**:

Reaction time: 48 h

Flash Chromatography: 10:1 Hex/EtOAc

TLC: R_f= 0.1 in 10:1 Hex/EtOAc, Stain: PMA (color: green), UV-active: blue

Physical state: off-white solid

% yield: 48 % (50 mg)

^{1}H NMR (500 MHz, Chloroform-*d*) δ 6.98 – 6.91 (m, 3H), 6.90 – 6.83 (m, 2H), 6.80 (d, *J* = 8.0 Hz, 1H), 5.96 (s, 2H), 5.10 (t, *J* = 6.6 Hz, 2H), 3.91 (s, 3H), 3.89 (s, 3H), 2.73 – 2.56 (m, 2H), 0.65 – 0.54 (m, 6H).

^{13}C NMR (126 MHz, Chloroform-*d*) δ 148.68, 147.90, 147.54, 146.40, 134.65, 133.14, 119.63, 118.66, 110.90, 109.87, 108.02, 107.18, 100.98, 82.89, 82.82, 56.01, 41.66, 41.60, 11.92.

HRMS-ESI (m/z): calculated $C_{21}H_{24}O_5$ + Na^+: 379.1521 found 379.1532.

Screened Lewis and Brønsted Acids to Convert CL5a into a Dihydronapthalene (CL3) scaffold.

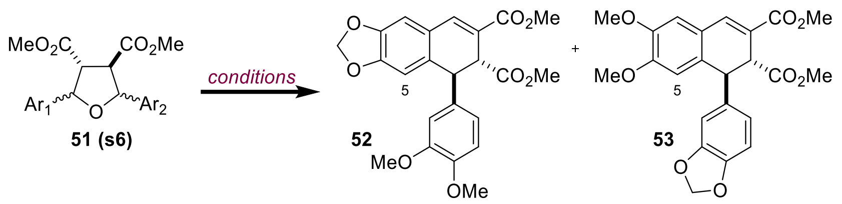

entry	*Conditions*	*r.r.* (73:74:73$_{C5}$:74$_{C5}$)	% conversion (%IY)
		Best conditions	
**1**[a]	$FeCl_3$ (5 equiv.), $CHCl_3$, 12 h, -5 °C	100 : 0 : 0 : 0	100 (47 %)
**2**	$FeCl_3$ (5 equiv.), $CHCl_3$, 1 h, -5 °C	68 : 32 : 0 : 0	100
**3**	HCl (1 equiv.), HFIP, 0-5 °C	50 : 46 : 4 : 0	100 (95 %)
		Other Lewis Acids: (DCM [0.05 M], 12 h, 23 °C)	
**5**	BF_3-OEt_2 (1 equiv.)	74 : 26 : 0 : 0	100
**6**	$InCl_3$ (1 equiv.)	74 : 26 : 0 : 0	100
		Other Brønsted Acids:	
**10**	TMSOTf (1 equiv.), DCM, 12 h, -5 °C	*N/A*	0

**11**	HCl (3 equiv.), DCM, 12 h, 23 °C	*N/A*	0
**12**	TMSCl (5 equiv.) DCM, 12 h, 23 °C	*N/A*	0

a. LCMS analysis of additional fractions collected after flash chromatography of entry 1 revealed accumulation of products whose masses corresponded to the desired product absent of methyl groups and the methylene carbon of the 1,3-benzodioxole. Unfortunately, these products were inseparable and could not be isolated in pure amounts. If the reaction is stopped earlier (1 h, entry 2), the selectivity of the reaction is *d.r.* 68:32:0:0. Therefore, this reaction is not selective for a single product, but the selectivity of the reaction is enhanced through a demethylation decomposition pathway.

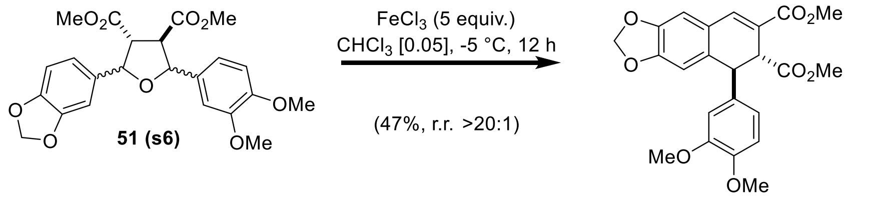

52
Chemical Formula: $C_{23}H_{22}O_8$
Exact Mass: 426.13

Synthesis of **52**: A 50-mL round bottom flask with a stirring bar was charged with **51(s6)** (250 mg, 0.56 mmol, 1 equiv.) and $CHCl_3$ (11.2 mL, [0.05 M]). The reaction was stirred and cooled to -5 °C (acetone/immersion cooler) and $FeCl_3$ (455 mg, 2.88 mmol, 5 equiv.) was added in one portion. The reaction was stirred for 12 h. At the completion of the stir period, saturated Rochelle's salt solution (30-50 mL) was added to the reaction and the mixture was left to stir until all the iron salt dissolved. At the completion of this step, the reaction was biphasic and extracted thrice with DCM. The combined organic fractions were washed with saturated NH_4Cl, brine, and dried over $MgSO_4$. The resultant organic solution is reduced *in vacuo* and further purified by flash chromatography. The selectivity of the reaction was determined by crude ^1H NMR prior to purification.

Reaction time: 12 h

Flash Chromatography: 4:1 Hex/EtOAc → 3:1 Hex/EtOAc

TLC: R_f= 0.2 in 4:1 Hex/EtOAc, Stain: PMA (color: green)

% yield: 47% (113 mg)

Physical state: off-white solid

r.r. >20:1

^{1}H NMR (500 MHz, Chloroform-*d*) δ 7.61 (s, 1H), 6.82 (s, 1H), 6.69 (d, *J* = 8.3 Hz, 1H), 6.64 (d, *J* = 2.0 Hz, 1H), 6.62 (s, 1H), 6.45 (dd, *J* = 8.3, 2.0 Hz, 1H), 5.97 (d, *J* = 2.3 Hz, 2H), 4.60 (d, *J* = 2.9 Hz, 1H), 3.99 (d, *J* = 2.9 Hz, 1H), 3.81 (s, 3H), 3.80 (s, 3H), 3.75 (s, 3H), 3.64 (s, 3H).

^{13}C NMR (126 MHz, Chloroform-*d*) δ 172.91, 167.10, 149.58, 149.00, 148.05, 147.20, 137.61, 134.70, 132.26, 125.56, 122.87, 119.80, 111.14, 110.92, 109.91, 108.95, 101.65, 56.00, 55.96, 52.66, 52.12, 47.17, 46.12.

HRMS-ESI (m/z): calculated $C_{23}H_{22}O_8 + Na^+$: 449.1212 found 449.1197.

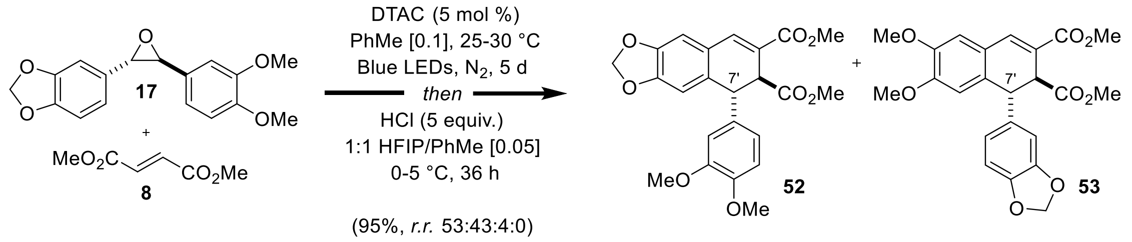

Synthesis of **52** and **53**: A flame dried 100-mL recovery flask with a magnetic stirring bar was charged with **17** (901 mg, 3 mmol, 1 equiv.), **8** (1.3 g, 9 mmol, 3 equiv.), DTAC (52 mg, 0.15 mmol, 0.05 equiv) and dissolved in PhMe [30 mL, 0.1 M]. The flask was sealed and sparged with N_2 for 15-20 minutes under sonication. The resultant mixture was placed under stirring and irradiated with blue LEDs for 5 days or until full consumption of **17** was observed by TLC (4:1 Hex/EtOAc). Once complete, the reaction mixture was removed from irradiation, cooled to 0-5 ℃ (brine/immersion cooler), and diluted with HFIP (30 mL, [0.05 M]). After a 5 min. stir period, 12 M HCl (1.25 mL, 15 mmol, 5 equiv.) was added dropwise at which point a color change from yellow to crimson red was observed. The resultant red solution was stirred for a 36 h period or until full consumption of the [3+2] product was observed by TLC (1:1 Hex/Et_2O). The reaction was reduced *in vacuo* and the resultant residue was introduced into a separatory funnel with the assistance of EtOAc (~100 mL). The organic layer was washed with $NaHCO_3$ and the aqueous layer was extracted twice with EtOAc. The combined organic layers were washed with brine and dried over $MgSO_4$. The solution was reduced *in vacuo* and purified by flash chromatography. After flash chromatography, the resultant material (1.219 g, 95%, *r.r.* 53:43:4:0) was dissolved in Et_2O (30 mL) and placed under stirring for 30 min. After the stir period was complete, the mixture was heterogeneous and the formed solid was collected in a glass frit and further triturated with cold Et_2O (50 mL). The collected solid is **52** (517 mg, *r.r.* 98:2:0:0). The filtrate was reduced *in vacuo* and the resultant yellow solid was **53** (560 mg, *r.r.* 10:83:7:0).

Reaction time: 5 d + 36 h

Flash Chromatography: 3:1 Hex/EtOAc

TLC: R_f= 0.2 in 4:1 Hex/EtOAc, UV-vis active (Blue)

Trituration: Et_2O

Physical state: **52** white solid, **53** yellow solid

% yield: 95% (1.219 g) → 88% (1.077 g) (after trituration)

Major (**73**): 517 mg, *r.r.* 98:2:0:0 (after trituration)

^{1}H NMR (500 MHz, Chloroform-*d*) δ 7.61 (s, 1H), 6.82 (s, 1H), 6.69 (d, *J* = 8.3 Hz, 1H), 6.64 (d, *J* = 2.0 Hz, 1H), 6.62 (s, 1H), 6.45 (dd, *J* = 8.3, 2.0 Hz, 1H), 5.97 (d, *J* = 2.3 Hz, 2H), 4.60 (d, *J* = 2.9 Hz, 1H), 3.99 (d, *J* = 2.9 Hz, 1H), 3.81 (s, 3H), 3.80 (s, 3H), 3.75 (s, 3H), 3.64 (s, 3H).

^{13}C NMR (126 MHz, Chloroform-*d*) δ 172.91, 167.10, 149.58, 149.00, 148.05, 147.20, 137.61, 134.70, 132.26, 125.56, 122.87, 119.80, 111.14, 110.92, 109.91, 108.95, 101.65, 56.00, 55.96, 52.66, 52.12, 47.17, 46.12.

HRMS-ESI (m/z): calculated $C_{23}H_{22}O_8$ + Na^+: 449.1212 found 449.1197.

Minor (**74**): 560 mg, *r.r.* 10:83:7:0 (after trituration)

^{1}H NMR (500 MHz, Chloroform-*d*) δ 7.67 (s, 1H), 6.86 (s, 1H), 6.65 (d, *J* = 8.0 Hz, 1H), 6.64 (s, 1H), 6.49 (dd, *J* = 8.0, 1.8 Hz, 1H), 6.45 (d, *J* = 1.7 Hz, 1H), 5.90 – 5.86 (m, 2H), 4.62 (d, *J* = 2.3 Hz, 1H), 3.96 (d, *J* = 2.3 Hz, 1H), 3.90 (s, 3H), 3.83 (s, 3H), 3.76 (s, 3H), 3.64 (s, 3H).

^{13}C NMR (126 MHz, Chloroform-*d*) δ 172.82, 167.16, 151.02, 148.40, 147.82, 146.50, 137.74, 136.64, 130.20, 124.32, 122.34, 120.79, 112.13, 111.90, 108.33, 108.19, 101.10, 56.15, 56.09, 52.67, 52.06, 47.40, 45.73.

HRMS-ESI (m/z): calculated $C_{23}H_{22}O_8$ + Na^+: 449.1212 found 449.1218.

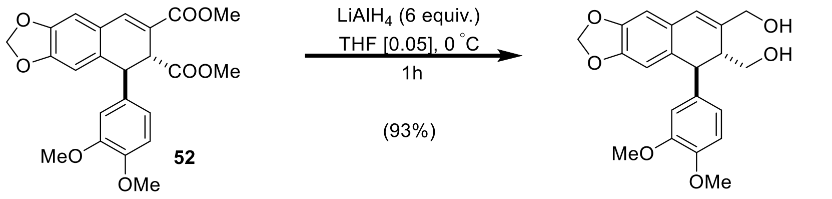

s12
Chemical Formula: $C_{21}H_{22}O_6$
Exact Mass: 370.14

Synthesis of **s12**: A 100-mL round bottom flask with a stir bar was charged with **52** (554 mg, 1.29 mmol, 1 equiv.) and THF (26 mL, [0.05 M]). The reaction was cooled to 0 °C (ice/water bath) and $LiAlH_4$ (294 mg, 7.74 mmol, 6 equiv.) was added in small portions. The reaction was left to stir for 1 h at 0 °C or until full consumption of the starting material was seen by TLC (3:1 EtOAc/Hex). The reaction was quenched by dropwise addition of acetone until gas evolution seized. Then, a saturated solution of Rochelle's salt (~ 20 mL) was added followed by water (20 mL). The reaction mixture was left to stir until two biphasic layers were observed. The mixture was introduced into a separatory funnel and extracted thrice with Et_2O. The combined organic layers were dried over $MgSO_4$, filtered, and concentrated *in vacuo*. The resultant material was further purified *via* flash chromatography.

Reaction time: 1 h

Flash Chromatography: 1:1 Hex/EtOAc → 4:1 EtOAc/Hex

TLC: R_f= 0.1 in 1:1 Hex/EtOAc, Stain: PMA (color: green)

% yield: 93% (445 mg)

Physical state: white foam

^{1}H NMR (500 MHz, Chloroform-*d*) δ 6.69 (d, *J* = 8.3 Hz, 1H), 6.65 (s, 1H), 6.64 (d, *J* = 1.8 Hz, 1H), 6.55 (s, 1H), 6.49 (dd, *J* = 8.3, 1.8 Hz, 1H), 6.43 (s, 1H), 5.92 (s, 2H), 4.13 (d, *J* = 12.8 Hz, 1H), 4.05 (d, *J* = 12.8 Hz, 1H), 4.02 (d, *J* = 2.7 Hz, 1H), 3.81 (s, 3H), 3.78 (s, 3H), 3.64 (d, *J* = 6.7 Hz, 2H), 2.69 (td, *J* = 6.7, 2.8 Hz, 1H).

^{13}C NMR (126 MHz, Chloroform-*d*) δ 148.85, 147.64, 147.23, 146.65, 136.33, 136.32, 129.88, 127.05, 125.66, 119.79, 111.06, 110.14, 110.05, 107.20, 101.11, 66.39, 64.74, 55.97, 55.95, 47.17, 45.65.

HRMS-ESI (m/z): calculated $C_{21}H_{22}O_6$ + Na^+: 393.1314 found 393.1300.

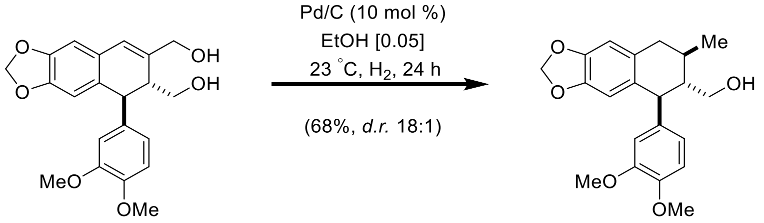

s12 **54**
Chemical Formula: $C_{21}H_{24}O_5$
Exact Mass: 356.16

Synthesis of **54**: A 50-mL round bottom flask with a magnetic stirring bar was charged with **s12** (370 mg, 0.99 mmol, 1 equiv.), EtOH (22 mL, [0.05 M]), and 10% wt. Pd/C (107 mg, 0.1 mmol, 0.1 equiv.). The reaction vessel was sealed, adapted with an H_2 balloon, and stirred for 24 h or until full consumption of **s12** was observed by TLC (1:1 Hex/EtOAc). At the completion of the reaction, the mixture was passed through a celite pad and the resulting solution was reduced *in vacuo* and further purified by flash chromatography.

Reaction time: 24 h

Flash Chromatography: 2:1 → 1:1 Hex/EtOAc

TLC: R_f= 0.2 in 2:1 Hex/EtOAc, Stain: PMA (color: green)

Physical state: white solid

% yield: 68% (240 mg)

d.r. 18:1

¹H NMR (500 MHz, Chloroform-*d*) δ 6.81 (d, *J* = 8.1 Hz, 1H), 6.74 (dd, *J* = 8.2, 1.9 Hz, 1H), 6.61 (s, 1H), 6.54 (s, 1H), 6.20 (s, 1H), 5.82 (s, 2H), 3.96 (d, *J* = 10.8 Hz, 1H), 3.87 (s, 3H), 3.81 (s, 3H), 3.78 (dd, *J* = 11.1, 2.6 Hz, 1H), 3.45 (dd, *J* = 11.1, 2.6 Hz, 1H), 2.77 (dd, *J* = 15.8, 4.6 Hz, 1H), 2.62 (dd, *J* = 15.8, 11.5 Hz, 1H), 2.01 (tp, *J* = 11.5, 6.4 Hz, 1H), 1.56 (tt, *J* = 10.8, 2.6 Hz, 1H), 1.22 (s, 1H), 1.14 (d, *J* = 6.4 Hz, 3H).

¹³C NMR (126 MHz, Chloroform-*d*) δ 149.28, 147.75, 145.81, 145.61, 138.45, 133.57, 129.83, 121.77, 112.19, 111.23, 109.79, 107.76, 100.64, 61.27, 56.08, 56.03, 50.72, 47.78, 39.40, 30.26, 19.85.

HRMS-ESI (m/z): calculated $C_{21}H_{24}O_5 + Na^+$: 379.1521 found 379.1507.

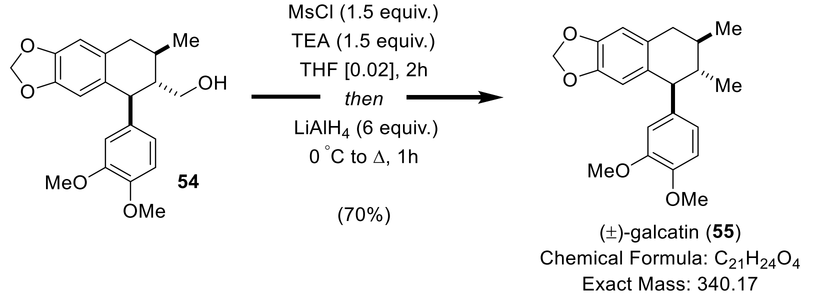

Synthesis of (±)-galcatin (**55**)[23]: A flame dried 50-mL round bottom flask with a magnetic stirring bar was charged with **54** (107 mg, 0.3 mmol, 1 equiv.) and THF (6 mL, [0.05 M]). The stirred solution was cooled to 0 °C (water/ice bath) and TEA (63 µL, 0.45 equiv, 1.5 equiv.) and MsCl (35 µL, 0.45, 1.5 equiv.) were added in that order. The reaction was left to warm to room temperature and after a 2 h stir period, full consumption of **54** was attained as determined by TLC (1:1 Hex/EtOAc). The reaction was again cooled to 0 °C and $LiAlH_4$ (69 mg, 1.8 mmol, 6 equiv) was added in one portion. Once gas evolution seized the resultant mixture was refluxed for 30 min, cooled to 23 °C, and analyzed by TLC (4:1 Hex/EtOAc) to confirm full consumption of the mesylated intermediate. The reaction was quenched by cooling to 0 °C and adding acetone until gas evolution seized. Then, saturated Rochelle's salt solution (30 mL) was added and the mixture was further diluted with Et_2O (30 mL). The resultant mixture was stirred until two clear biphasic layers were obtained, at which point the mixture was introduced into a separatory funnel and the water layer was extracted thrice with Et_2O. The combined organic layers were dried over Na_2SO_4 and reduced *in vacuo*. The resultant material was purified by flash chromatography.

Reaction Time: 2 h + 30 min.

Flash Chromatography: 10:1 Hex/EtOAc

TLC: R_f= 0.3 in 10:1 Hex/EtOAc, Stain: PMA (color: green)

Physical state: white solid

% yield: 70% (71 mg)

¹H NMR (500 MHz, Chloroform-*d*) δ 6.80 (d, *J* = 8.1 Hz, 1H), 6.69 (dd, *J* = 8.1, 1.7 Hz, 1H), 6.60 – 6.55 (m, 1H), 6.53 (s, 1H), 6.15 (s, 1H), 5.81 (s, 2H), 3.88 (s, 3H), 3.82 (s, 3H), 3.39 (d, *J* = 10.5 Hz, 1H), 2.73 (dd, *J* = 16.3, 4.5 Hz, 1H), 2.59 (dd, *J* = 15.9, 11.8 Hz, 1H), 1.63 (qt, *J* = 11.5, 6.5 Hz, 1H), 1.58 – 1.47 (m, 1H), 1.07 (d, *J* = 6.4 Hz, 3H), 0.86 (d, *J* = 6.4 Hz, 3H).

^{13}C NMR (126 MHz, Chloroform-*d*) δ 149.11, 147.57, 145.69, 145.52, 139.19, 133.73, 130.12, 121.94, 112.28, 111.03, 109.71, 107.74, 100.60, 56.06, 56.02, 54.78, 43.85, 39.60, 35.66, 20.08, 17.37.

HRMS-ESI (m/z): calculated $C_{21}H_{24}O_4$ + Na^+: 363.1572 found 363.1570.

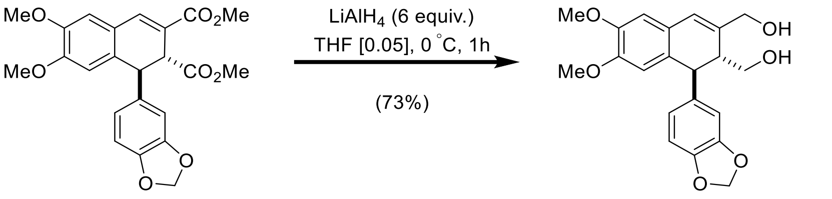

53

56
Chemical Formula: $C_{21}H_{22}O_6$
Exact Mass: 370.14

Synthesis of **56**: A 100-mL round bottom flask with a stir bar was charged with **53** (554 mg, 1.29 mmol, 1 equiv.) and THF (26 mL, [0.05 M]). The reaction was cooled to 0 °C (ice/water bath) and $LiAlH_4$ (294 mg, 7.74 mmol, 6 equiv.) was added in small portions. The reaction was left to stir at 0 °C until full consumption of the starting material was seen by TLC (3:1 EtOAc/Hex). The reaction was quench by dropwise addition of acetone until gas evolution seized. Then, a saturated solution of Rochelle's salt (~ 20 mL) was added followed by water (20 mL). The reaction mixture was left to stir until two biphasic layers were observed. The mixture was introduced into a separatory funnel and extracted thrice with Et_2O. The combined organic layers were dried over $MgSO_4$, filtered, and concentrated *in vacuo*. The resultant material was further purified *via* flash chromatography.

Reaction time: 1 h

Flash Chromatography: 3:1 EtOAc/Hex

TLC: R_f= 0.3 in 3:1 EtOAc/Hex, Stain: PMA (color: green)

Physical state: white foam

% yield: 69% (347 mg)

^{1}H NMR (500 MHz, Chloroform-*d*) δ 6.69 (s, 1H), 6.66 (d, *J* = 7.9 Hz, 1H), 6.58 (s, 1H), 6.51 – 6.46 (m, 3H), 5.88 (d, *J* = 3.9 Hz, 2H), 4.13 (d, *J* = 13.0 Hz, 1H), 4.06 (d, *J* = 13.0 Hz, 1H), 4.03 (s, 1H), 3.89 (s, 3H), 3.80 (s, 3H), 3.62 (qd, *J* = 10.4, 6.8 Hz, 2H), 2.65 (td, *J* = 6.8, 2.1 Hz, 1H), 2.42 (bs, 2H).

^{13}C NMR (126 MHz, Chloroform-*d*) δ 148.85, 148.06, 147.66, 146.05, 138.12, 135.99, 128.11, 125.90, 125.42, 120.72, 112.72, 110.23, 108.31, 108.19, 100.97, 66.45, 64.91, 56.13, 56.05, 47.71, 45.35.

HRMS-ESI (m/z): calculated $C_{21}H_{22}O_6$ + Na^+: 393.1314 found 393.1319.

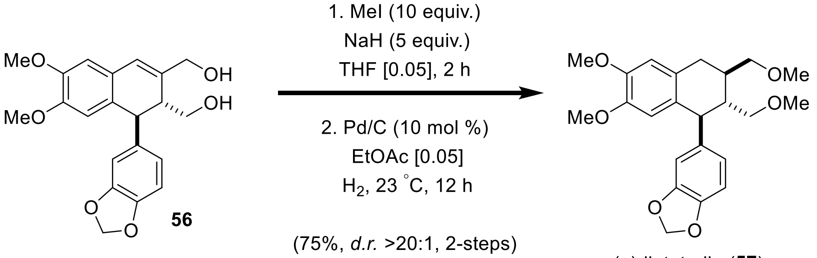

(±)-lintetralin (**57**)
Chemical Formula: $C_{23}H_{28}O_6$
Exact Mass: 400.19

Synthesis of (±)-lintetralin (**57**)[24]: A 25-mL round bottom flask with a magnetic stirring bar was charged with **56** (50 mg, 0.13 mmol, 1 equiv.) and dissolved in THF (2.6 mL, [0.05 M]). The mixture was cooled to 0°C (water/ice bath) and NaH (29 mg,

0.67 mmol, 5 equiv.) was added in one portion. After a 5 min. stir period MeI (41 µL, 0.65 mmol, 5 equiv.) was added and the reaction was left to warm to room temperature. After 1 h, TLC (3:1 EtOAc/Hex) analysis showed multiple spots at which point more MeI (41 µL, 0.65 mmol, 5 equiv.) was added to the reaction. After a 1 h stir period TLC (1:1 Hex/EtOAc) analysis showed full consumption of the starting material and a single spot in the TLC (R_f: 0.3 in 4:1 Hex/EtOAc).The reaction was quenched by cooling 0°C and adding water dropwise until gas evolution seized. The mixture was then introduced into a separatory funnel and extracted thrice with Et_2O. The combined organic fractions were dried over $MgSO_4$ and concentrated *in vacuo* to afford a yellow viscous oil.

The resultant material from the previous reaction was placed in a 10-mL scintillation vial, dissolved in EtOAc (2.6 mL, [0.05 M]), and Pd/C (14 mg, 0.013 mmol, 0.1 equiv) was added in one portion. The mixture was sealed and a H_2 balloon was adapted to it. The reaction mixture was stirred for a 12 h period or until full consumption of the starting material was observed by TLC (R_f: 0.3 in 4:1 Hex/EtOAc).The mixture was passed through a 1:1 celite/silica plug with the assistance of EtOAc and the organic mixture was concentrated *in vacuo*. The resultant material was further purified by flash chromatography.

Reaction Time: 2 h + 12 h

Flash Chromatography: 4:1 Hex/EtOAc

TLC: R_f= 0.4 in 4:1 Hex/EtOAc, Stain: PMA (color: green)

% yield: 75% (38 mg) 2-steps

Physical state: clear oil

d.r. >20:1

^{1}H NMR (500 MHz, Chloroform-*d*) δ 6.74 (d, *J* = 7.9 Hz, 1H), 6.64 (dd, *J* = 7.9, 1.3 Hz, 1H), 6.60 (s, 1H), 6.58 – 6.55 (m, 1H), 6.24 (s, 1H), 5.93 (d, *J* = 3.3 Hz, 2H), 3.99 (d, *J* = 10.3 Hz, 1H), 3.84 (s, 3H), 3.61 (s, 3H), 3.48 (dd, *J* = 9.3, 3.8 Hz, 1H), 3.43 (dd, *J* = 9.2, 6.5 Hz, 1H), 3.40 – 3.36 (overlap, 1H), 3.36 (s, 3H), 3.27 (s, 3H), 3.11 (dd, *J* = 9.6, 3.1 Hz, 1H), 2.82 (d, *J* = 7.8 Hz, 2H), 2.14 (dtq, *J* = 11.9, 8.1, 4.0 Hz, 1H), 1.79 (tt, *J* = 10.1, 3.0 Hz, 1H).

^{13}C NMR (126 MHz, Chloroform-*d*) δ 147.81, 147.26, 147.15, 146.00, 139.81, 131.97, 129.04, 122.81, 112.99, 111.13, 109.49, 107.94, 100.95, 75.39, 71.26, 59.09, 59.03, 56.03, 55.93, 47.37, 45.22, 36.40, 33.33.

HRMS-ESI (m/z): calculated $C_{23}H_{28}O_6$ + Na^+: 423.1784 found 423.1764.

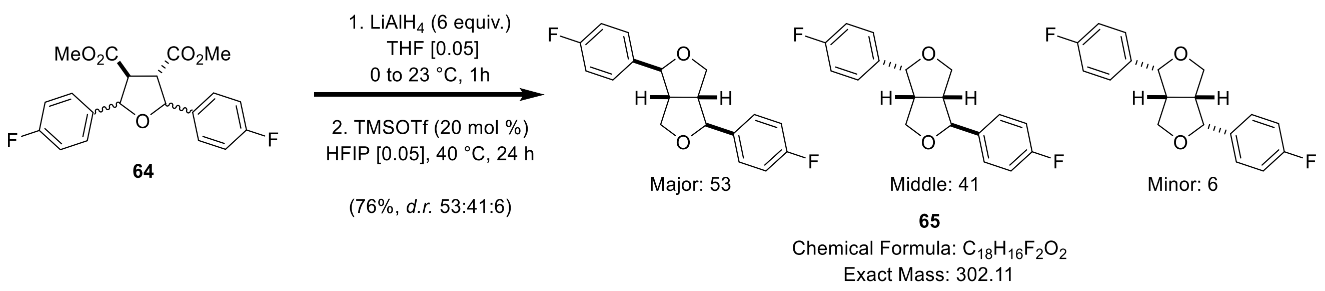

Synthesis of **65**: A 100-mL round bottom flask with a magnetic stirring bar was charged with **64** (511 mg, 1.36 mmol, 1 equiv.) and THF (27 mL, [0.05 M]). The stirred reaction was cooled to 0 °C (ice/water bath) and $LiAlH_4$ (310 mg, 8.16 mmol, 6 equiv.) was added in one portion. The reaction was left to warm to room temperature and stirred for 30 min. Once full consumption of **64** was obtained by TLC (4:1 Hex/EtOAc), the reaction was cooled to 0 °C, diluted with Et_2O, and quenched by dropwise addition of acetone. Thereafter, saturated solution of Rochelle's salt was added and the reaction was left to stir until the gray solution became clear. The resultant mixture was introduced into a separatory funnel and extracted with Et_2O thrice. The combined organic fractions were dried over $MgSO_4$ and reduced *in vacuo*. The resultant diol was used without further purification.

A 100-mL round bottom flask with a magnetic stirring bar was charged with the resultant diol and HFIP (27 mL, [0.05 M]). To the stirred reaction was added TMSOTf (50 µL, 0.272 mmol, 0.2 equiv.) and the reaction was sealed with a

septum adapted with a needle for venting. The reaction was heated to 40 °C and left to stir for 24 h until full consumption of the starting material was observed by TLC (4:1 Hex/EtOAc). The reaction was cooled to room temperature, concentrated *in vacuo*, and introduced to a seperatory funnel with the assistance of EtOAc. The resultant mixture was washed with saturated $NaHCO_3$ and the aqueous layer was further extracted twice with EtOAc. The combined organic layers were dried over $MgSO_4$, reduced *in vacuo*, and further purified by flash chromatography. The major compound was isolated as a mixture of two stereoisomer with the minor compound (224 mg, d.r. 13:1). This material can be further purified by recrystallization with isopropanol alcohol to yield 68 mg of the major in d.r >40:1. The Middle compound was isolated as a pure compound (89 mg, single diastereomer).

Reaction time: 1 h + 24 h

Flash Chromatography: 20:1 → 10:1 Hex/EtOAc

TLC: R_f=0.4 (mid), 0.3 (maj) in 10:1 Hex/EtOAc, Stain: PMA (color: green)

% yield: 76 % (313 mg)

Physical state: white solid (major), white solid (middle)

Major (d.r. >40:1):

^{1}H NMR (500 MHz, Chloroform-*d*) δ 7.32 (dd, *J* = 8.4, 5.5 Hz, 4H), 7.05 (t, *J* = 8.7 Hz, 4H), 4.80 (d, *J* = 3.7 Hz, 2H), 4.26 (dd, *J* = 9.0, 6.8 Hz, 2H), 3.92 (dd, *J* = 9.3, 3.4 Hz, 2H), 3.13 – 3.02 (m, 2H).

^{13}C NMR (126 MHz, Chloroform-*d*) δ 163.43, 161.47, 136.94, 136.92, 127.75, 127.69, 115.68, 115.51, 85.41, 71.91, 54.56.

^{19}F NMR (470 MHz, Chloroform-*d*) δ -114.83 (ddd, *J* = 13.9, 8.7, 5.3 Hz).

GCMS: calculated $C_{18}H_{16}F_2O_2$: 302.1 found 302.1.

Middle (single diastereomer):

^{1}H NMR (500 MHz, Chloroform-*d*) δ 7.40 – 7.28 (m, 4H), 7.05 (td, *J* = 8.7, 3.6 Hz, 4H), 4.89 (d, *J* = 6.0 Hz, 1H), 4.48 (d, *J* = 7.1 Hz, 1H), 4.15 (d, *J* = 9.5 Hz, 1H), 3.87 (dd, *J* = 9.5, 6.3 Hz, 1H), 3.30 – 3.19 (m, 1H), 3.41 – 3.31 (m, 1H), 3.25 (t, 1H), 2.95 – 2.85 (m, 1H).

^{13}C NMR (126 MHz, Chloroform-*d*) δ 163.52, 163.05, 161.56, 161.10, 137.05, 137.02, 134.15, 134.13, 127.87, 127.80, 127.36, 127.30, 115.66, 115.49, 115.44, 115.27, 87.28, 81.82, 71.17, 69.86, 54.90, 50.23.

^{19}F NMR (470 MHz, Chloroform-*d*) δ -114.67 (ddd, *J* = 14.1, 9.0, 5.2 Hz), -115.36 (ddd, *J* = 14.1, 9.1, 5.4 Hz).

GCMS: calculated $C_{18}H_{16}F_2O_2$: 302.1 found 302.1.

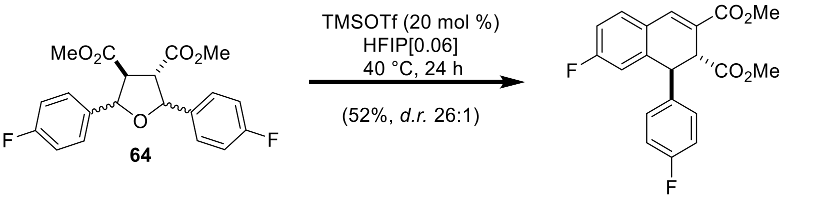

66

Chemical Formula: $C_{20}H_{16}F_2O_4$

Exact Mass: 358.10

Synthesis of **66**: A 5-mL pressure flask with a stirring bar was charged with **64** (120 mg, 0.3 mmol, 1 equiv.) and HFIP (5 mL, [0.06 M]). To the stirred reaction was added TMSOTf (12 uL, 0.064 mmol, 0.2 equiv.) at which point the clear solution turned dark red. The reaction was sealed and heated to 100 °C (oil bath) for a 24 h stir period. Completion of the reaction by TLC is difficult to access because the starting material and the product share the same R_f, but crude NMR can be used to determine full consumption of **64.** At the completion of the stir period the reaction was cooled to room temperature and introduced into a separatory funnel with the assistance of EtOAc. The organic mixture was washed with $NaHCO_3$ and

extracted thrice with EtOAc. The combined organic fractions were dried over $MgSO_4$ and concentrated *in vacuo*. The resultant residue was further purified by flash chromatography.

Reaction time: 24 h

Flash Chromatography: 9:1 Hex/EtOAc → 8:1 Hex/EtOAc

TLC: R_f= 0.2 in 9:1 Hex/EtOAc, Stain: PMA (color: green)

% yield: 52 % (59 mg)

Physical state: white solid

d.r. 26:1

^{1}H NMR (500 MHz, Chloroform-*d*) δ 7.65 (s, 1H), 7.14 – 7.05 (m, 2H), 7.01 (dd, *J* = 8.4, 2.7 Hz, 1H), 6.99 – 6.93 (m, 2H), 6.93 – 6.88 (m, 2H), 4.74 (d, *J* = 2.8 Hz, 1H), 4.04 (d, *J* = 2.8 Hz, 1H), 3.78 (s, 3H), 3.63 (s, 3H).

^{13}C NMR (126 MHz, Chloroform-*d*) δ 172.16, 166.60, 163.16, 162.87, 161.20, 160.91, 137.86, 137.83, 136.65, 136.64, 133.23, 133.16, 132.18, 132.15, 130.85, 130.78, 129.24, 129.17, 126.23, 117.51, 117.34, 115.83, 115.70, 115.66, 115.53, 52.77, 52.38, 47.36, 44.81.

^{19}F NMR (470 MHz, Chloroform-*d*) δ -114.41 (td, *J* = 8.4, 5.3 Hz), -115.72 (ddd, *J* = 14.0, 8.6, 5.3 Hz).

HRMS-ESI (m/z): calculated $C_{20}H_{16}F_2O_4 + Na^+$: 381.0914 found 381.0912.

X-Ray Crystal Structure of Compound 13:

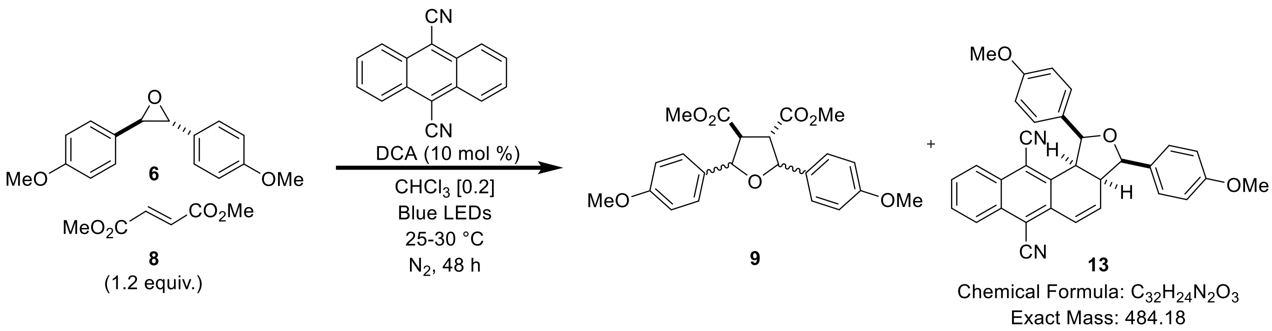

Synthesis of **29**: A 40-mL scintillation vial with a stirring bar was charged with **6** (1.02 g, 4 mmol, 1 equiv.), **8** (692 mg, 4.8 mmol, 1.2 equiv.), DCA (110 mg, 0.4 mmol, 0.1 equiv.) and $CHCl_3$ (20 mL, [0.2 M]). The reaction vessel was sealed, sparged with N_2 for 15-20 minutes, and irradiated with Blue LEDs for 48 h. At the completion of the stir period the reaction was reduced *in vacuo* and further purified *via* flash chromatography. The resulting solid **13** (197 mg) was further purified by recrystallization in PhMe (100 mg).

Crystals of **13** suitable for X-ray crystallography were obtained through slow evaporation of DCM in hexanes.

Reaction time: 48 h

Flash Chromatography: 4:1 Hex/EtOAc → 3:1 Hex/EtOAc

TLC: R_f= 0.3 in 4:1 Hex/EtOAc, Stain: PMA (color: green)

% yield: 52% (100 mg)

Physical state: white solid

^{1}H NMR (500 MHz, Chloroform-*d*) δ 8.05 (dq, *J* = 6.8, 3.5 Hz, 2H), 7.63 (dq, *J* = 6.8, 3.5 Hz, 2H), 7.44 (d, *J* = 8.6 Hz, 2H), 7.15 (d, *J* = 8.6 Hz, 2H), 7.01 (d, *J* = 8.6 Hz, 2H), 6.93 (dd, *J* = 10.1, 2.5 Hz, 1H), 6.46 (d, *J* = 8.6 Hz, 2H), 5.95 (dd, *J* = 10.1, 2.5 Hz, 1H), 5.70 (d, *J* = 10.2 Hz, 1H), 5.45 (d, *J* = 5.0 Hz, 1H), 4.70 (dd, *J* = 10.2, 8.2 Hz, 1H), 3.87 (s, 3H), 3.59 (ddt, *J* = 8.2, 5.0, 2.5 Hz, 1H), 3.54 (s, 3H).

^{13}C NMR (126 MHz, Chloroform-*d*) δ 159.42, 159.08, 139.90, 136.00, 133.96, 132.35, 130.88, 130.62, 129.99, 129.56, 129.41, 129.37, 128.42, 127.53, 126.06, 125.92, 125.46, 116.22, 115.35, 114.60, 114.13, 113.79, 113.58, 113.51, 113.06, 109.58, 85.01, 83.97, 55.49, 55.21, 46.64, 45.32.

HRMS-ESI (m/z): calculated $C_{32}H_{24}N_2O_3 + H^+$: 485.1865 found 485.1887.

VI. References:

1. Chai, C.; Armarego, W. L. F. Purification of Laboratory Chemicals; 6th ed.; Butterworth Heinemann: Oxford, **2009**.
2. Pangborn, A. B.; Giardello, M. A.; Grubbs, R. H.; Rosen, R. K.; Timmers, F. J. *Organometallics* **1996**, *15*, 1518.
3. Still, W. C.; Kahn, M.; Mitra, A. *J. Org. Chem.* **1978**, *43*, 2923.
4. Banerjee, S.; Yang, Y.F.; Jenkins, I.D.; Liang, Y.; Toutov, A.A.; Liu, W.B.; Schuman, D.P.; Grubbs, R.H.; Stoltz, B.M.; Krenske, E.H.; Houk, K.N.; Zare, R.N. *J. Am. Chem. Soc.* **2017**, *139*, 6880-6887.
5. Wang, Y.; Haze, O.; Dinnocenzo, J.P.; Farid, S. *J. Org. Chem.* **2007**, *72*, 6970-6981.
6. Alfonzo, E.; Mendoza, J.W.L.; Beeler, A.B. *Bel. J. Org. Chem.* **2018**, *14*, 2308-2312.
7. Pelter, A.; Ward, R.S.; Rao, E.V.; Sastry, K.V. *Tetrahedron* **1976**, *32*, 2783-2788.
8. Chen, C.-C.; Hsin, W.-C.; Huang, Y.-L. *J. Nat. Prod.* **1998**, *61*, 227-229.
9. Batterbee, J. E.; Burden, R. S.; Crombie, L.; Whiting, D. A. *J. Chem. Soc. C* **1969**, 2470.
10. Kato, M.J.; Yoshida, M.; Gottlieb, O.R. *Phytochemistry* **1992**, *31*, 283-287.
11. Satyanarayana, P.; Venkateswarlu, S. *Tetrahedron* **1991**, *47*, 8931-8940.
12. Krithika, R.; Mohankumar, R.; Verma, R.J.; Shrivastav, P.S., Mohamad, I.L.; Gunasekaran, P.; Narasimhan, S. *Chemico-Biological Interactions.* **2009**, *181*, 351-358.
13. Hwu, J.R.; Tseng, W.N.; Gnabre, J.; Giza, P.; Huang, R.C.C. *J. Med. Chem.* **1998**, *41*, 2994-3000.
14. Xie, X.; Stahl, S.S. *J. Am. Chem. Soc.* **2015**, *173*, 3767-3770.
15. Duan, S.; Huang, S.; Gong, J.; Shen, Y.; Zeng, L.; Feng, Y.; Ren, W.; Leng, Y.; Hu, Y.; *ACS Med. Chem. Lett.*, **2015**, *6*, 386-391.
16. e Silva, M.L.A. *et al.* Bioorg. Med. Chem. Lett. **2017**, *27*, 176-179.
17. Landais,Y.; Robin, J.-P.; Lebrun, A. *Tetrahedron Lett.* **1991**, *47*, 3787-3804.
18. Ward, R.S.; Pelter, A. *Tetrahedron* **1991**, *47*, 1275-1284.
19. Ayoub, S. M. H.; Kingston, D. G. I. *J. Nat. Prod.* **1984**, *47*, 875-876.
20. Lin-Gen, Z.; Seligmann, O.; Lotter, H.; Wagner, H. *Phytochemistry* **1983**, *22*, 265.
21. Roy, S.C.; Rana, K.K.; Guin, C. *J. Org. Chem.* **2002**, *67*, 3242-3248.
22. Ward, R.S.; Hughes, D.D. *Tetrahedron* **2001**, *57*, 2057-2064
23. Liu, J.-S.; Huang, M.-F.; Gao, Y.-L.; Findlay, J.A. *Can. J. Chem.* **1981**, *59*, 1680-1684.
24. Satyanarayana, P.; Row, L.R.; Ward, R.S.; Gopala, B.V.R. *Tetrahedron Lett.* **1979**, 3043.

.

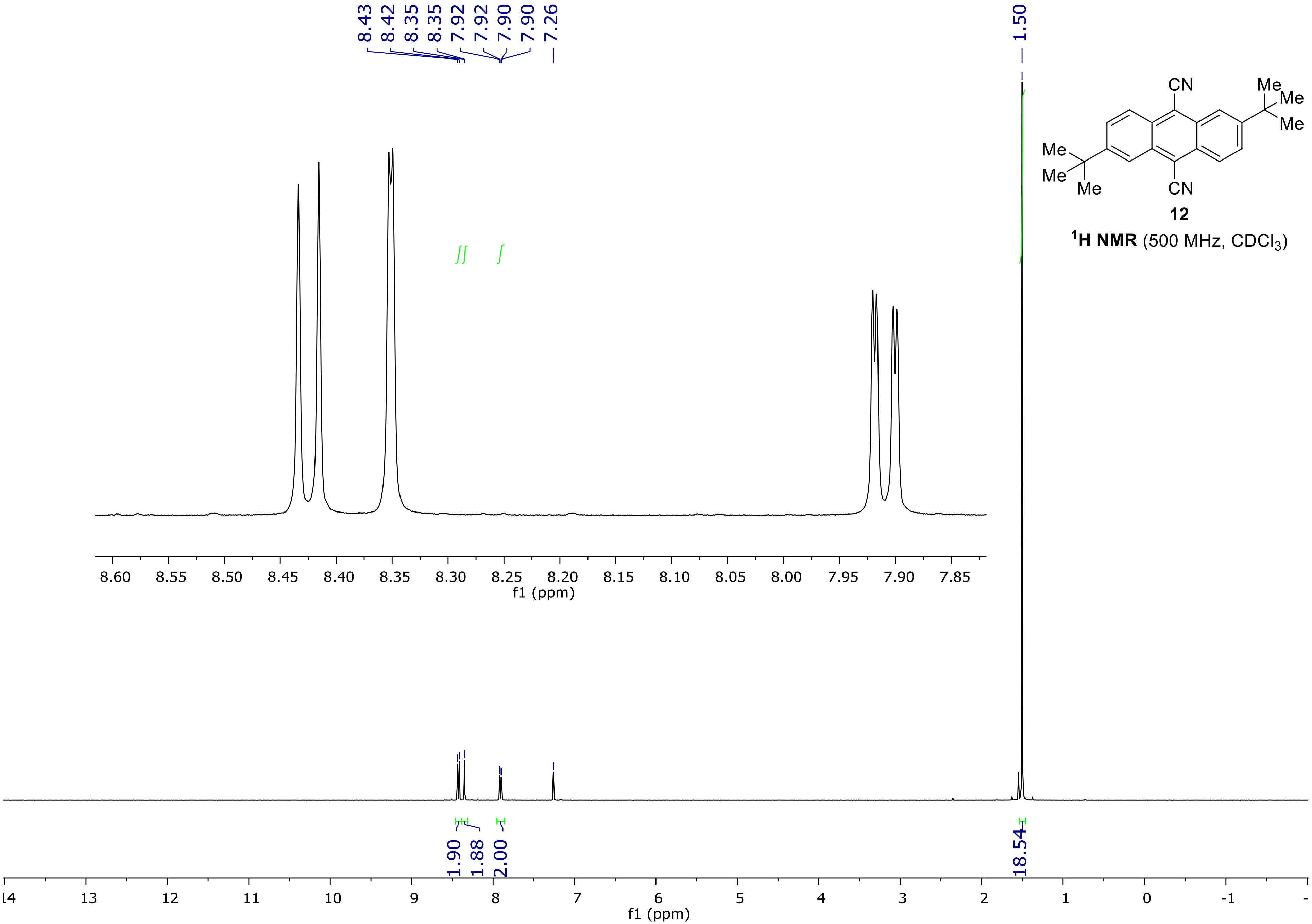

12

^{1}H NMR (500 MHz, $CDCl_3$)

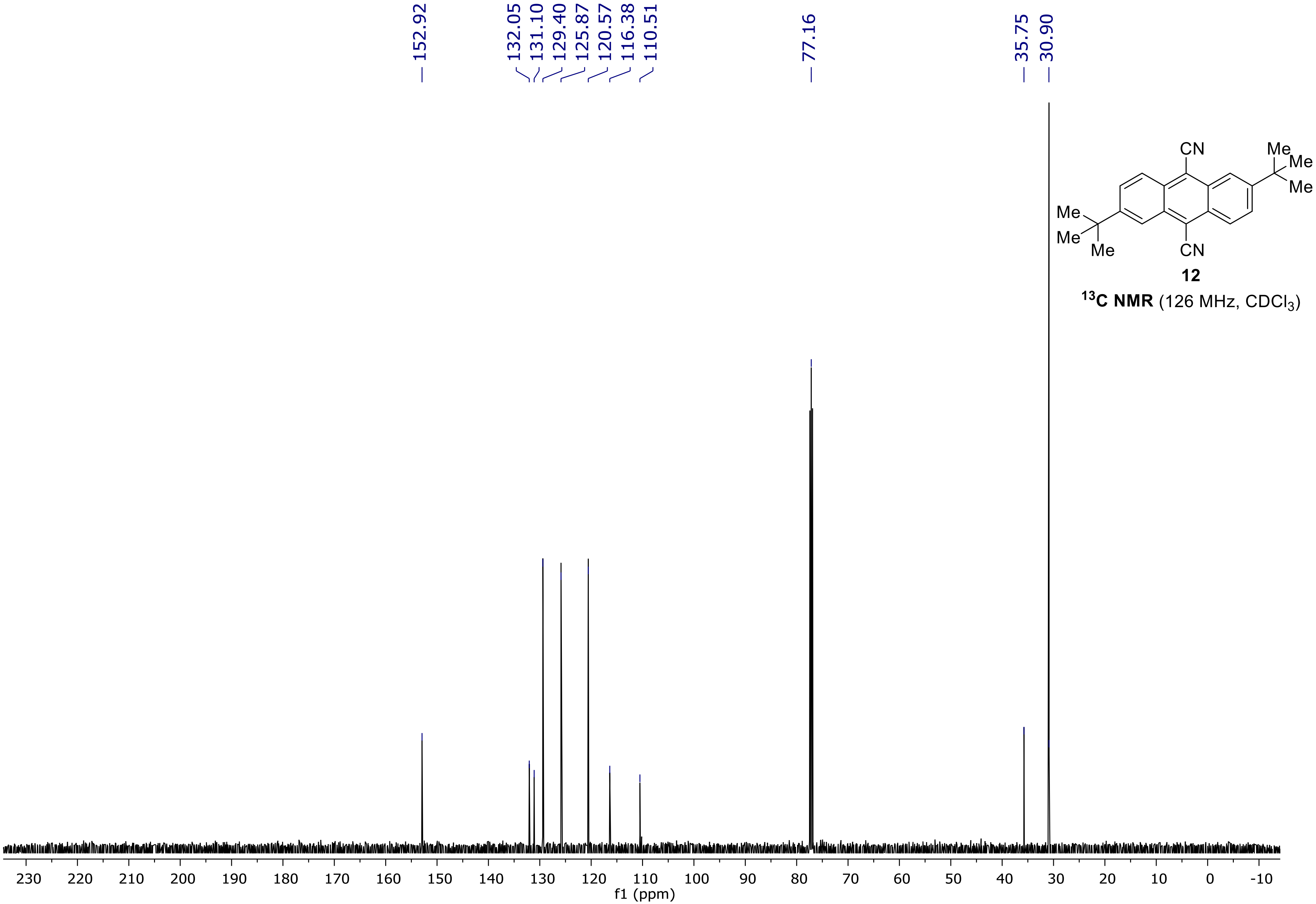

12

^{13}C NMR (126 MHz, $CDCl_3$)

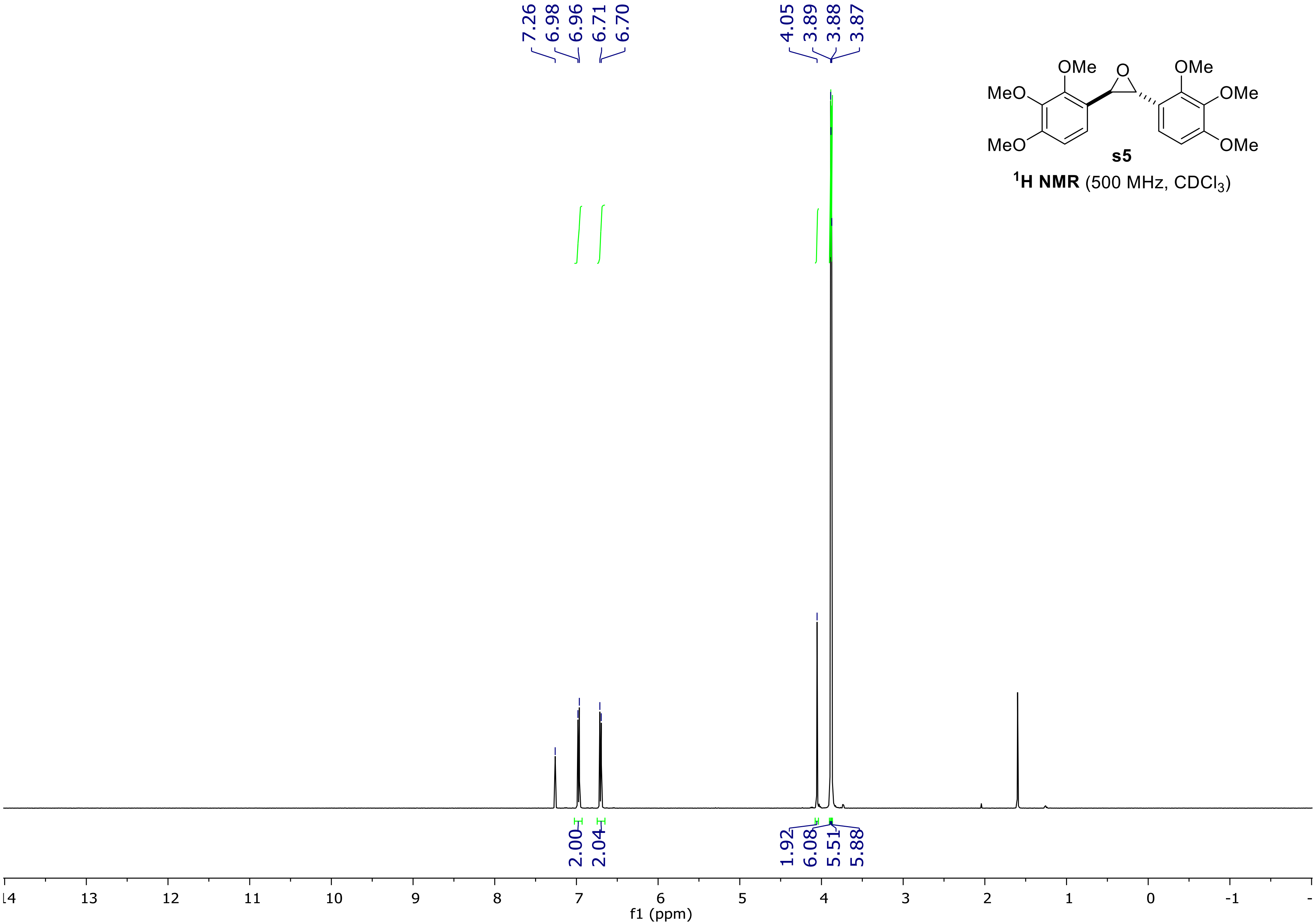

s5

^{1}H NMR (500 MHz, $CDCl_3$)

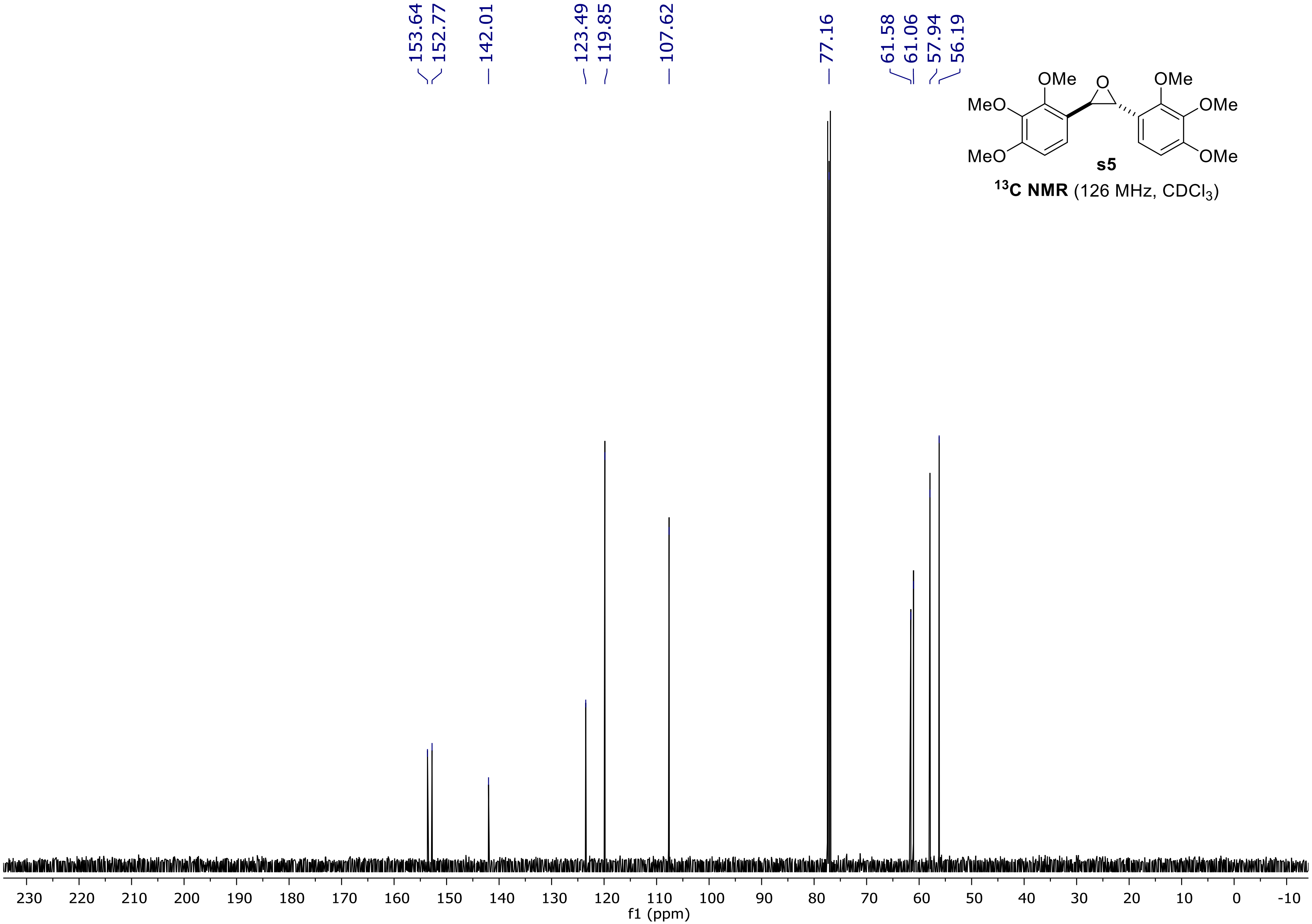

s5

^{13}C NMR (126 MHz, $CDCl_3$)

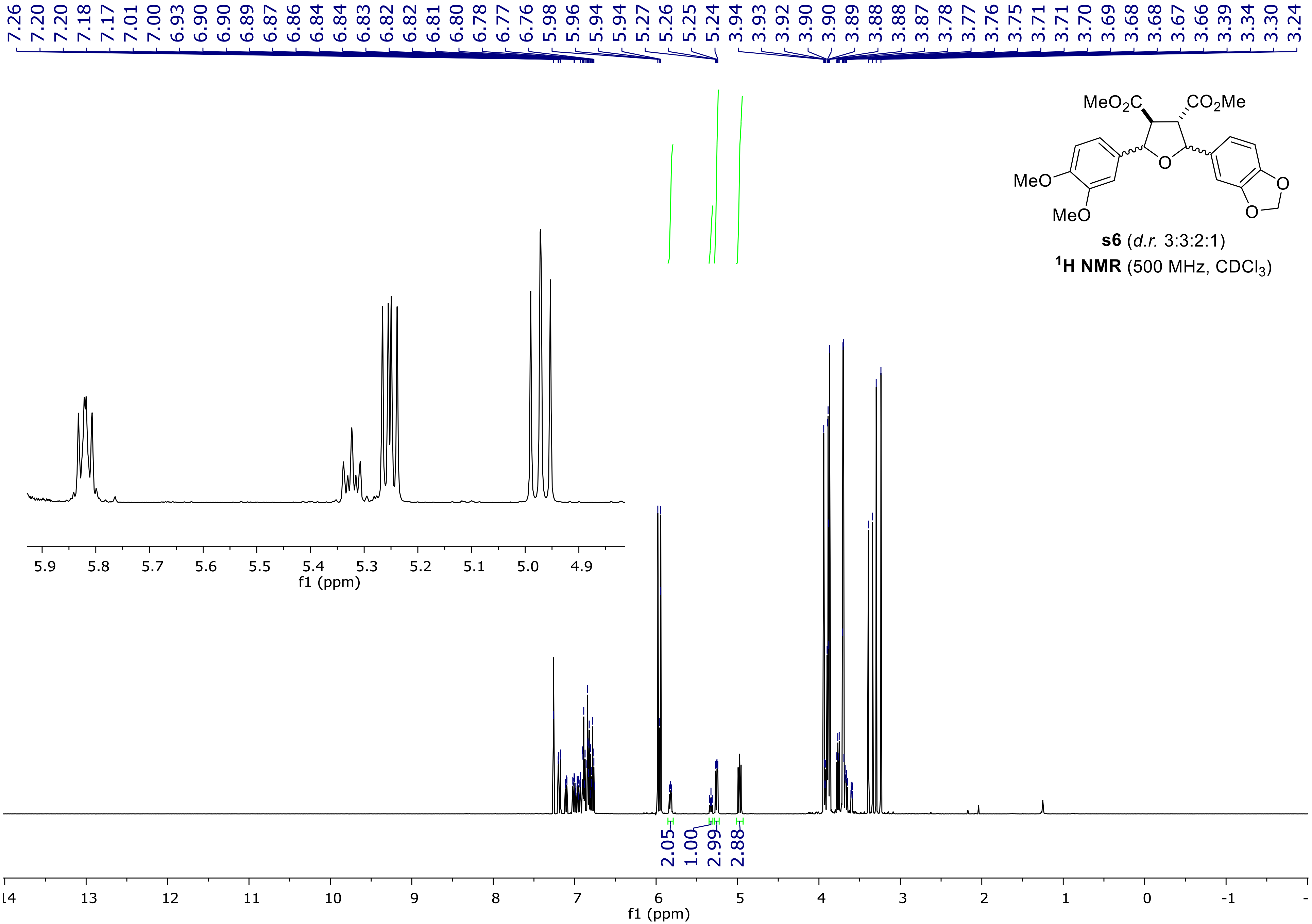
7.26 7.20 7.20 7.18 7.17 7.01 7.00 6.93 6.90 6.90 6.89 6.87 6.86 6.84 6.84 6.83 6.82 6.82 6.81 6.80 6.78 6.77 6.76 5.98 5.96 5.94 5.94 5.27 5.26 5.25 5.24 3.94 3.93 3.92 3.90 3.90 3.89 3.88 3.88 3.87 3.78 3.77 3.76 3.75 3.71 3.71 3.70 3.69 3.68 3.68 3.67 3.66 3.39 3.34 3.30 3.24
MeO2C
CO2Me
MeO
MeO
O
O
O
s6 (d.r. 3:3:2:1)
1H NMR (500 MHz, CDCl3)
5.9 5.8 5.7 5.6 5.5 5.4 5.3 5.2 5.1 5.0 4.9
f1 (ppm)
2.05 1.00 2.99 2.88
13 12 11 10 9 8 7 6 5 4 3 2 1 0 -1
f1 (ppm)

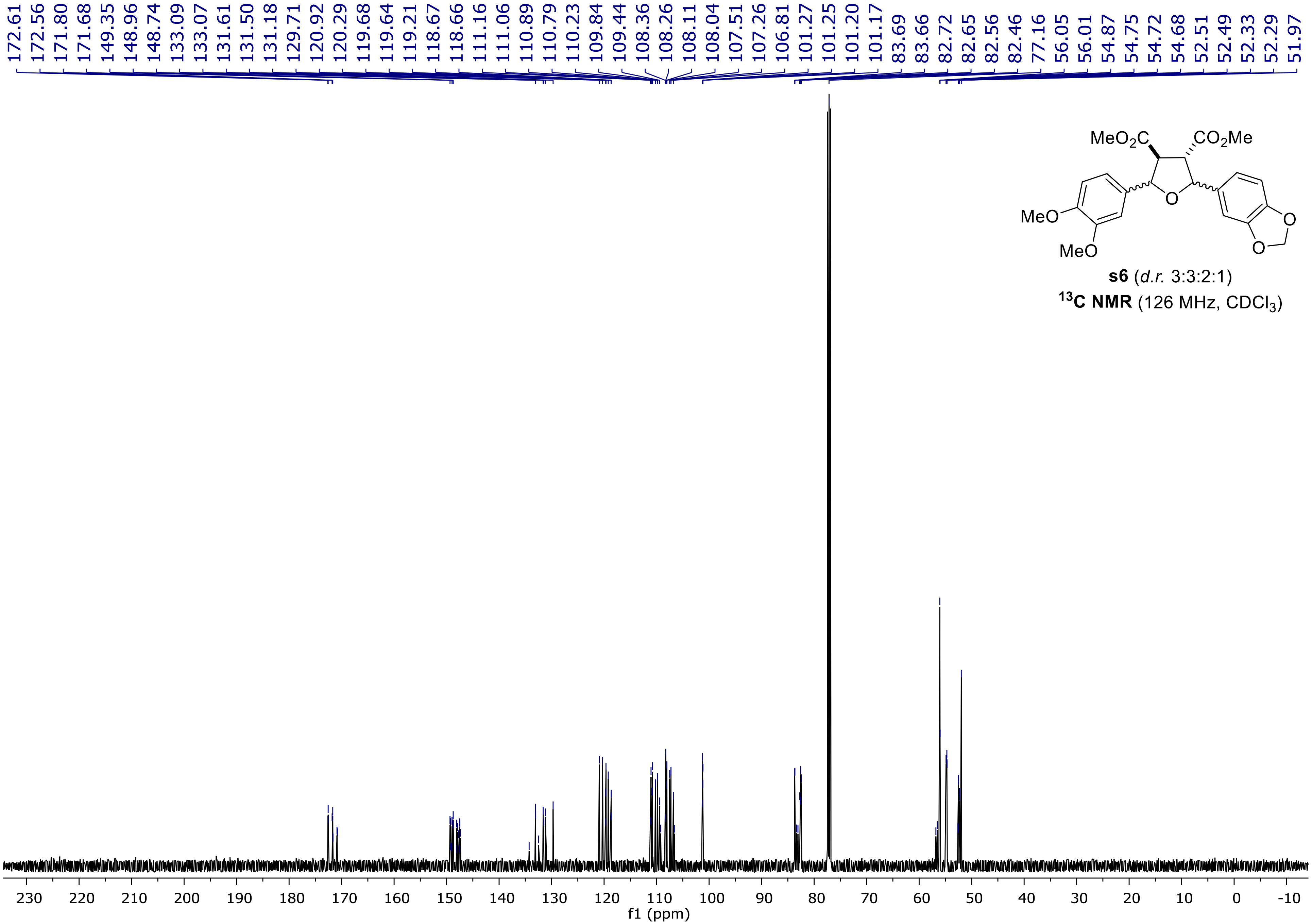

s6 (*d.r.* 3:3:2:1)

^{13}C NMR (126 MHz, $CDCl_3$)

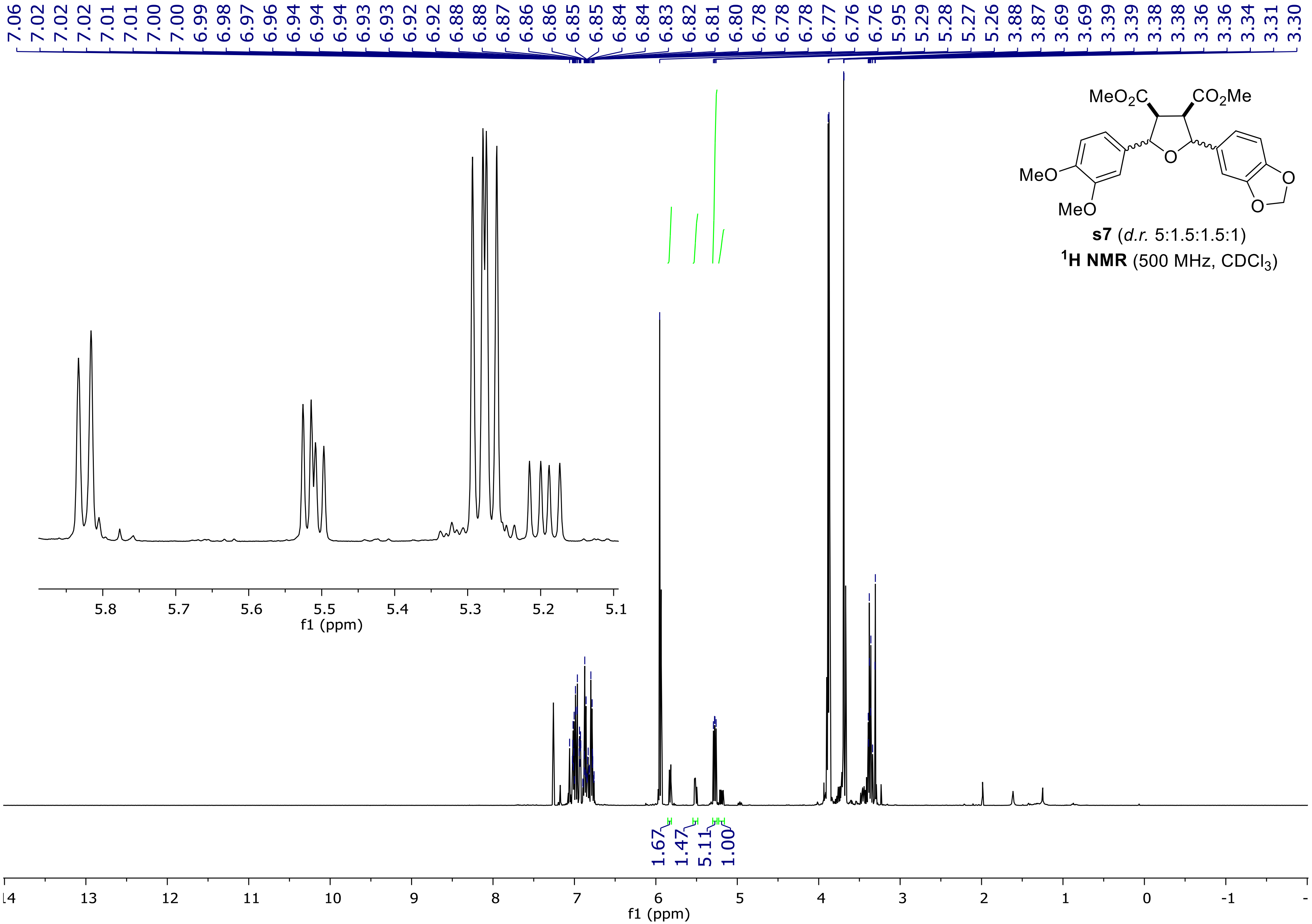

s7 (*d.r.* 5:1.5:1.5:1)

^{1}H NMR (500 MHz, $CDCl_3$)

Alfonzo 58

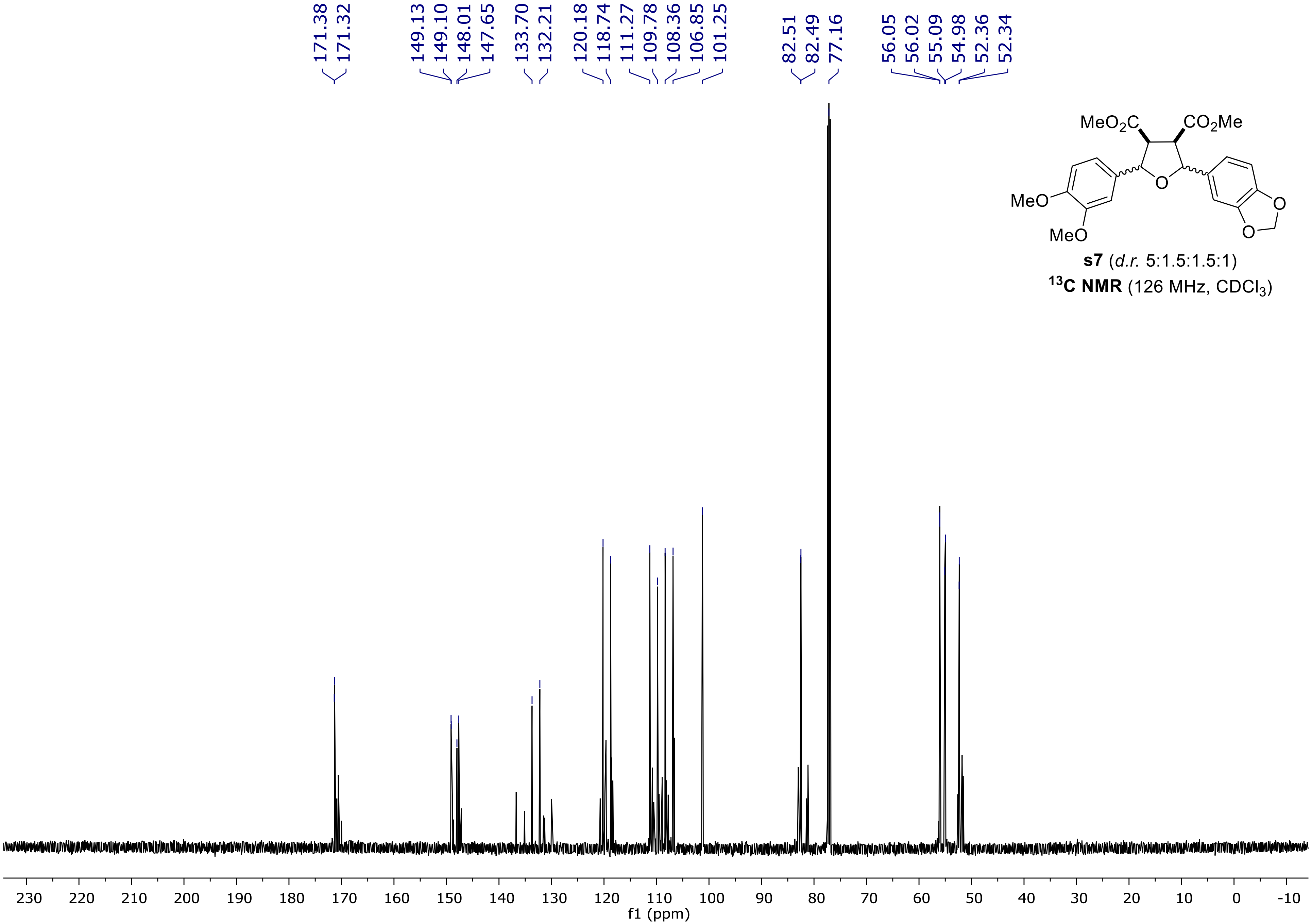

s7 (*d.r.* 5:1.5:1.5:1)

^{13}C NMR (126 MHz, $CDCl_3$)

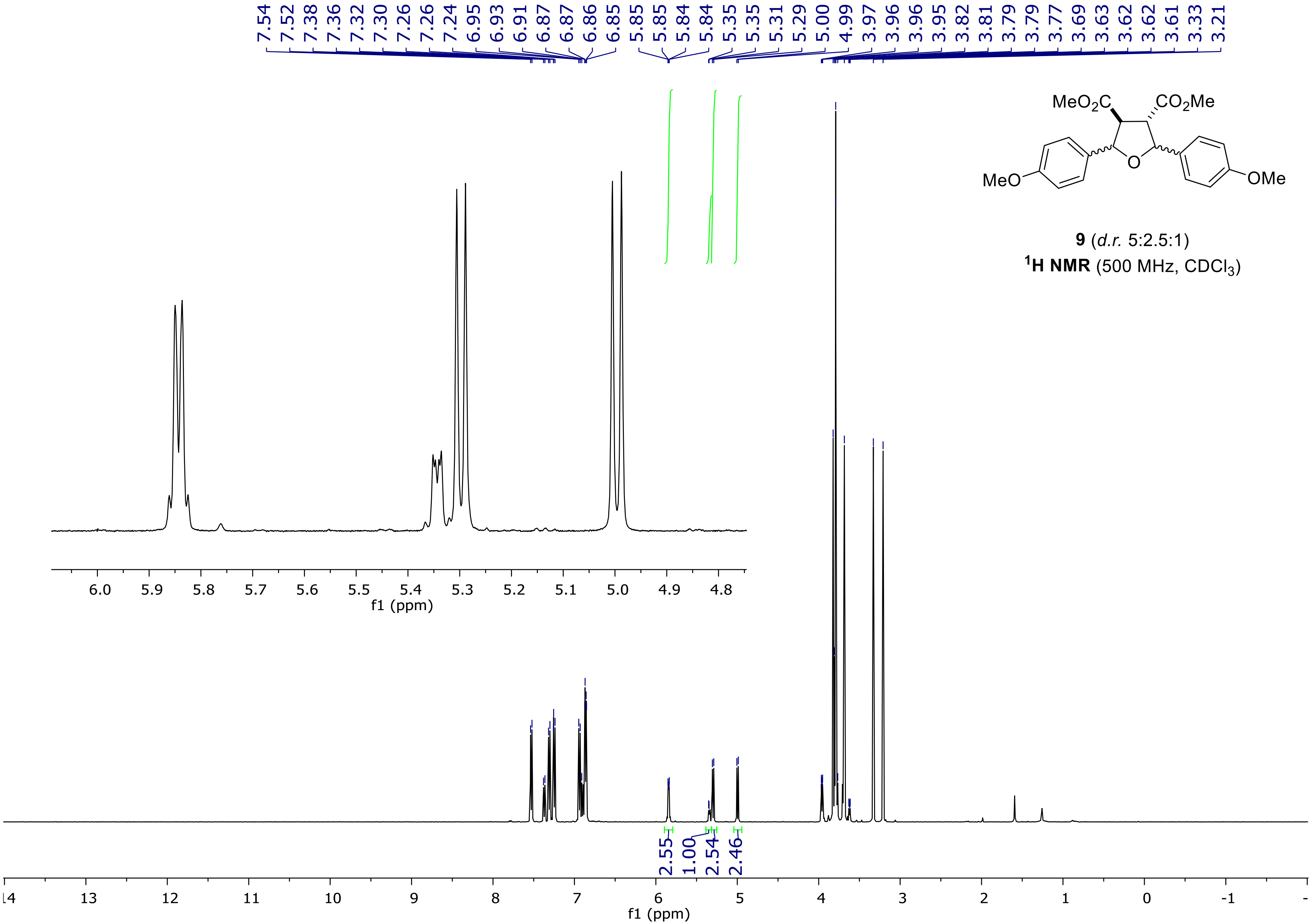

9 (*d.r.* 5:2.5:1)

^{1}H NMR (500 MHz, $CDCl_3$)

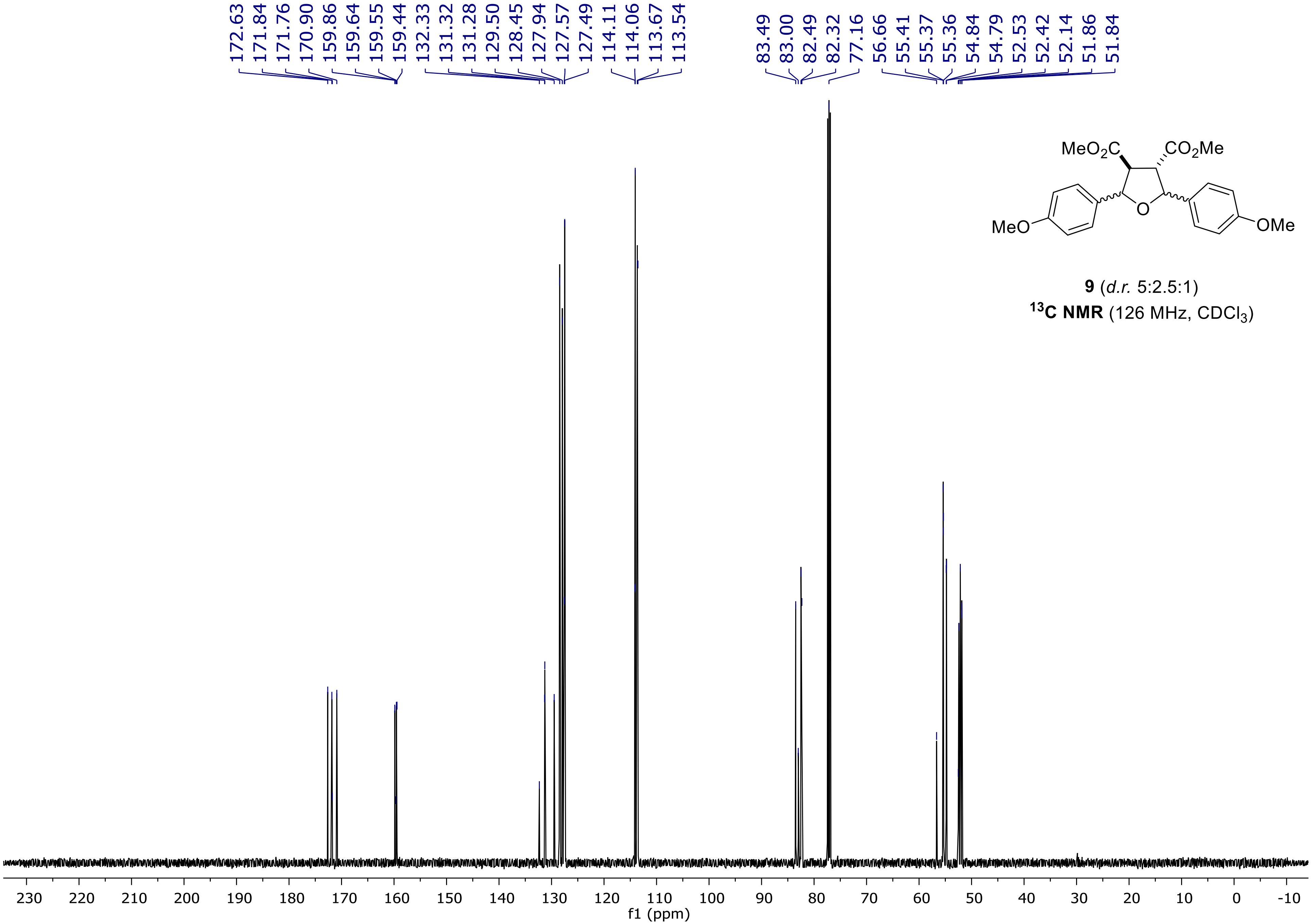

9 (*d.r.* 5:2.5:1)

^{13}C NMR (126 MHz, $CDCl_3$)

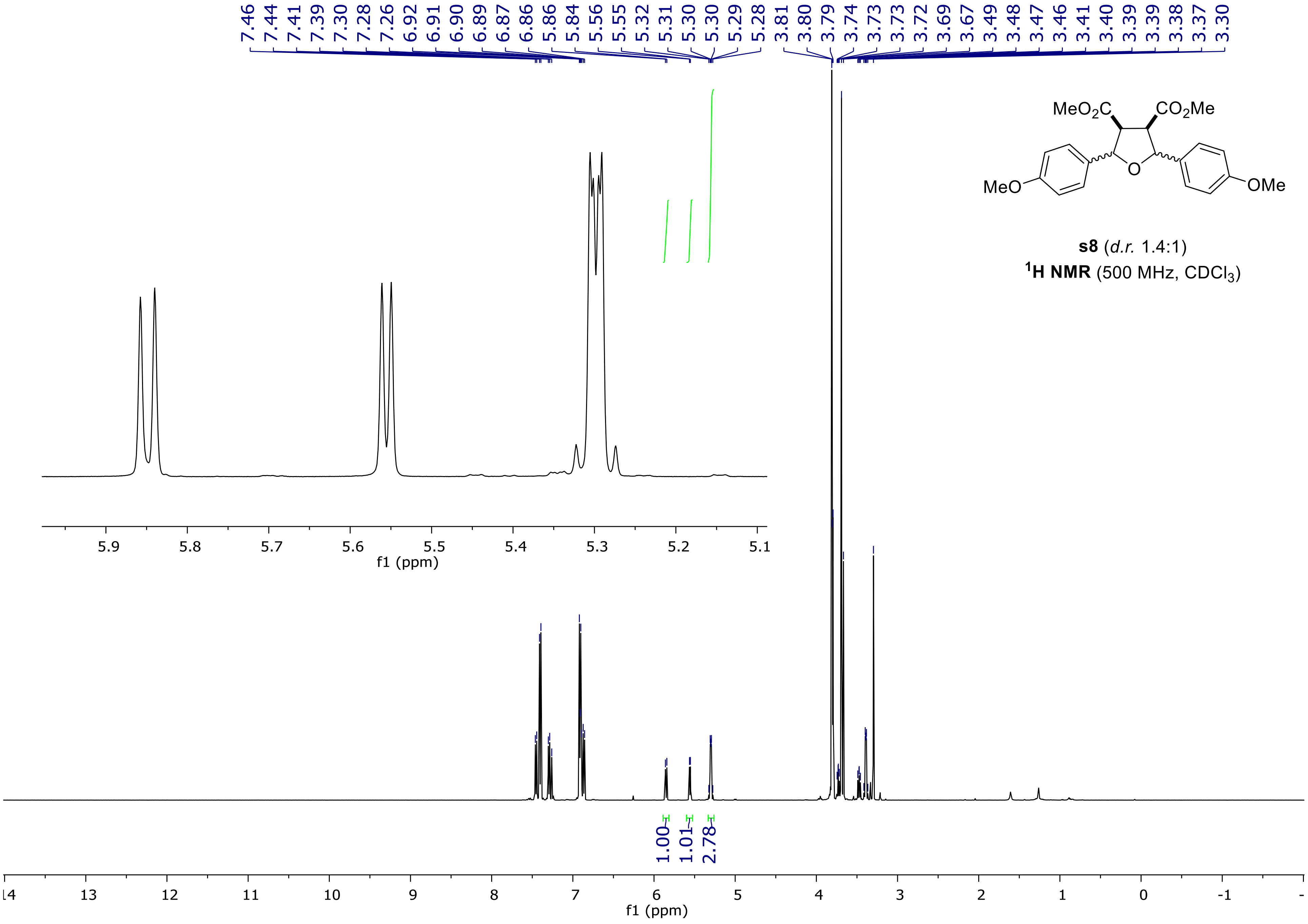

s8 (*d.r.* 1.4:1)

^{1}H NMR (500 MHz, $CDCl_3$)

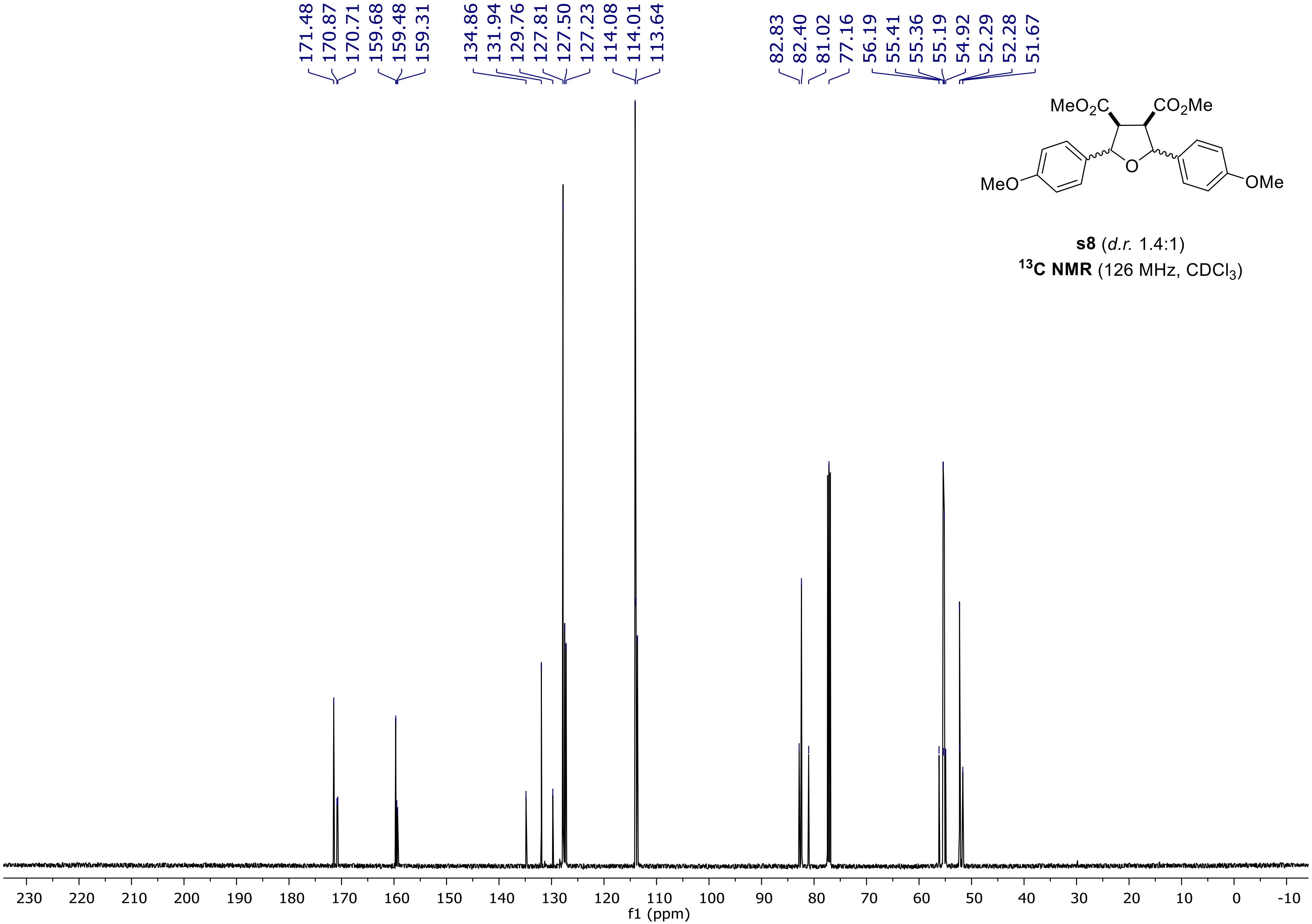

s8 (*d.r.* 1.4:1)

^{13}C NMR (126 MHz, $CDCl_3$)

Alfonzo 63

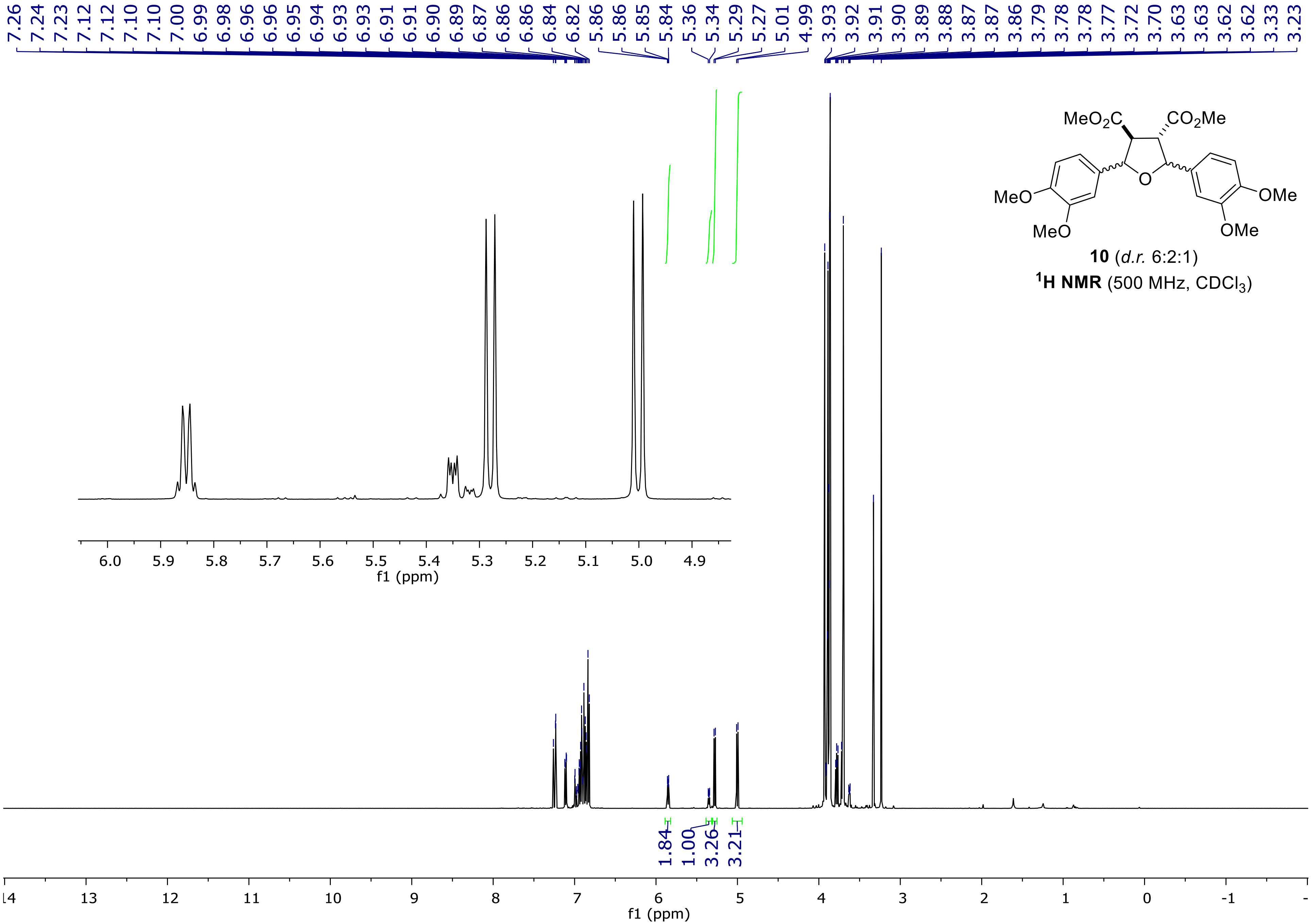

10 (*d.r.* 6:2:1)

^{1}H NMR (500 MHz, $CDCl_3$)

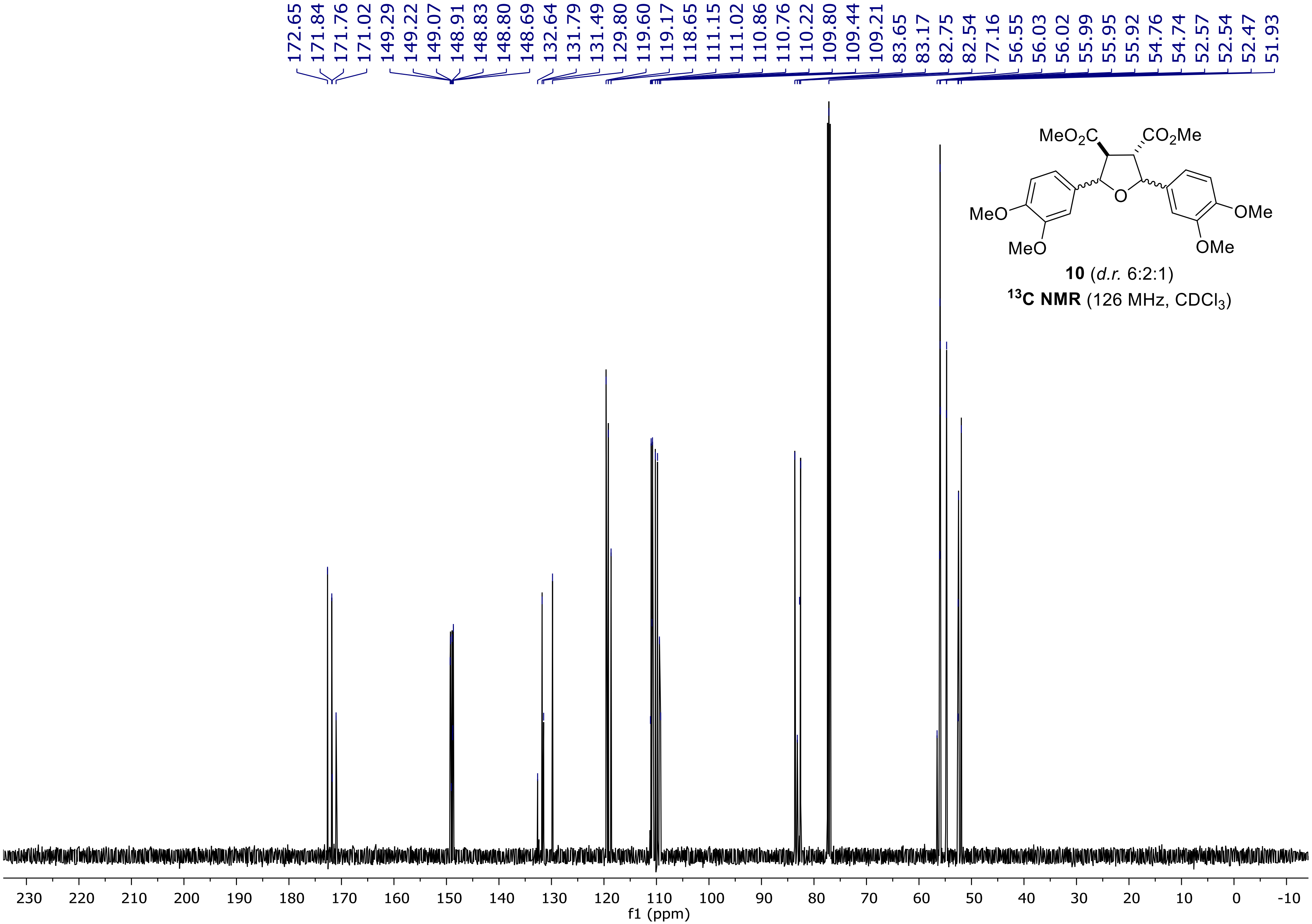

10 (*d.r.* 6:2:1)
^{13}C NMR (126 MHz, $CDCl_3$)

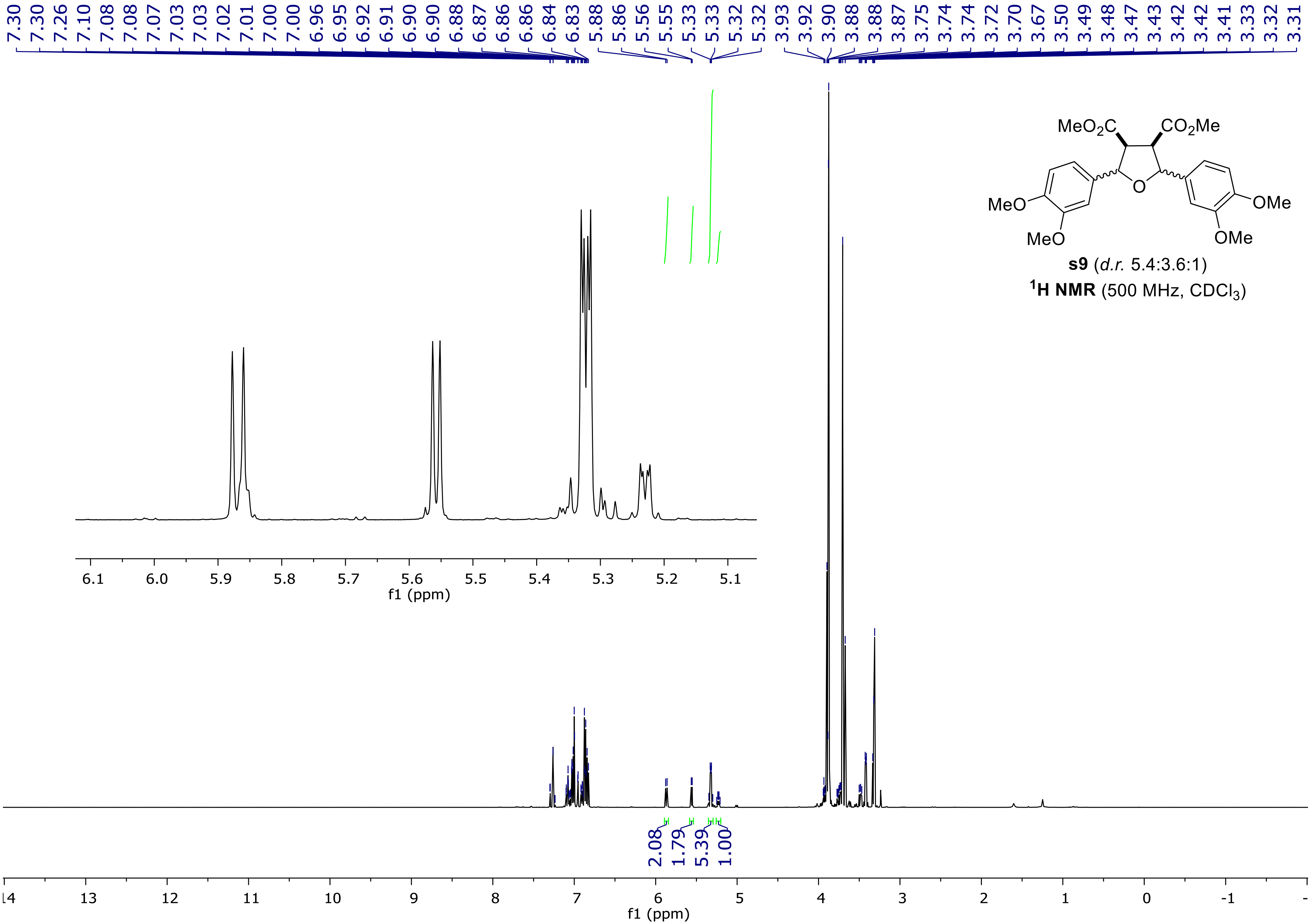

s9 (*d.r.* 5.4:3.6:1)

^{1}H NMR (500 MHz, $CDCl_3$)

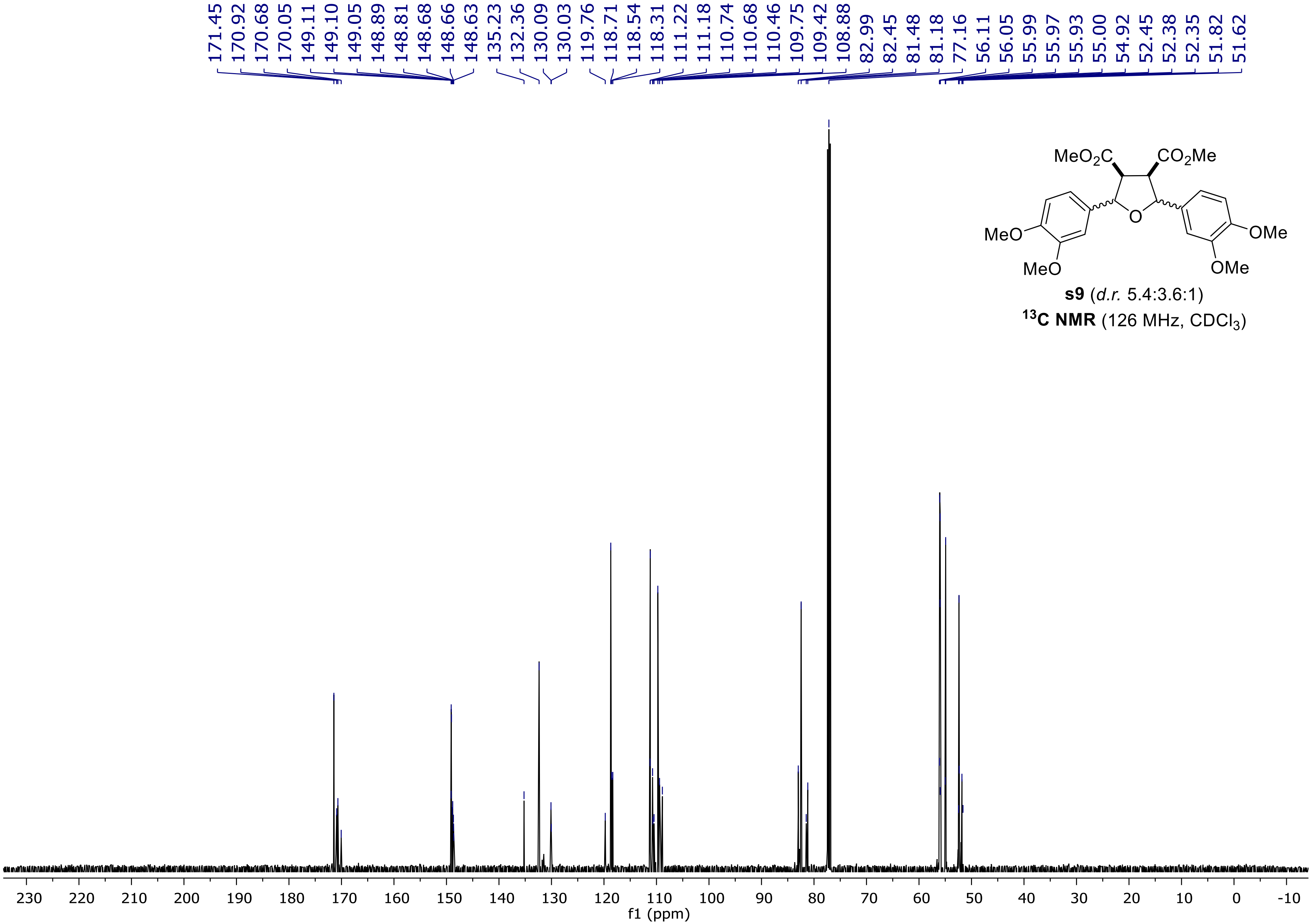

s9 (*d.r.* 5.4:3.6:1)

^{13}C NMR (126 MHz, $CDCl_3$)

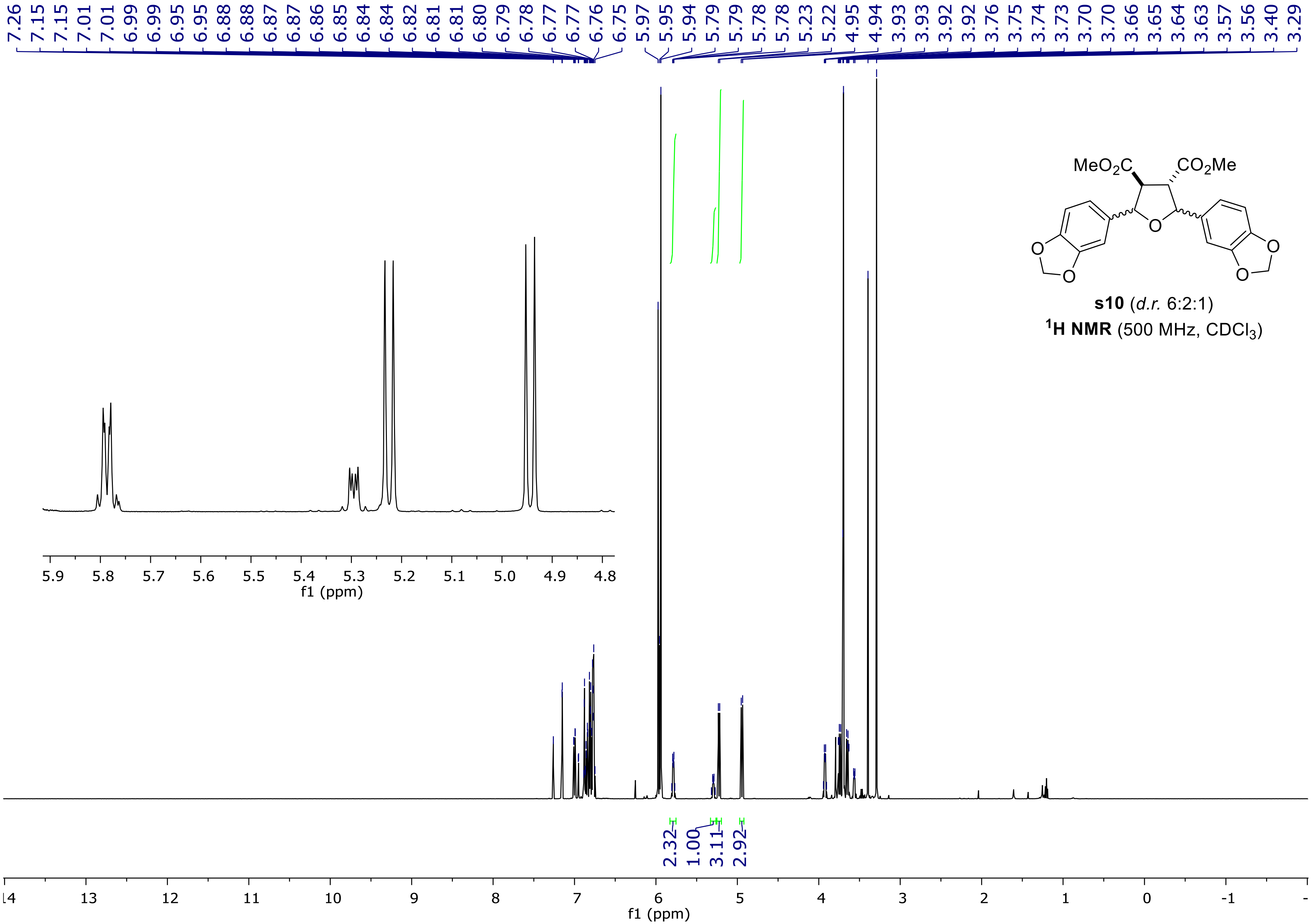
7.26 7.15 7.15 7.01 6.99 6.99 6.95 6.95 6.88 6.88 6.87 6.87 6.86 6.85 6.84 6.84 6.82 6.81 6.81 6.80 6.79 6.78 6.77 6.77 6.76 6.75 5.97 5.95 5.94 5.79 5.79 5.78 5.78 5.23 5.22 4.95 4.94 3.93 3.93 3.92 3.92 3.76 3.75 3.74 3.73 3.70 3.70 3.66 3.65 3.64 3.63 3.57 3.56 3.40 3.29
MeO2C
CO2Me
O
s10 (d.r. 6:2:1)
1H NMR (500 MHz, CDCl3)
5.9 5.8 5.7 5.6 5.5 5.4 5.3 5.2 5.1 5.0 4.9 4.8
f1 (ppm)
2.32
1.00
3.11
2.92
14 13 12 11 10 9 8 7 6 5 4 3 2 1 0 -1
f1 (ppm)

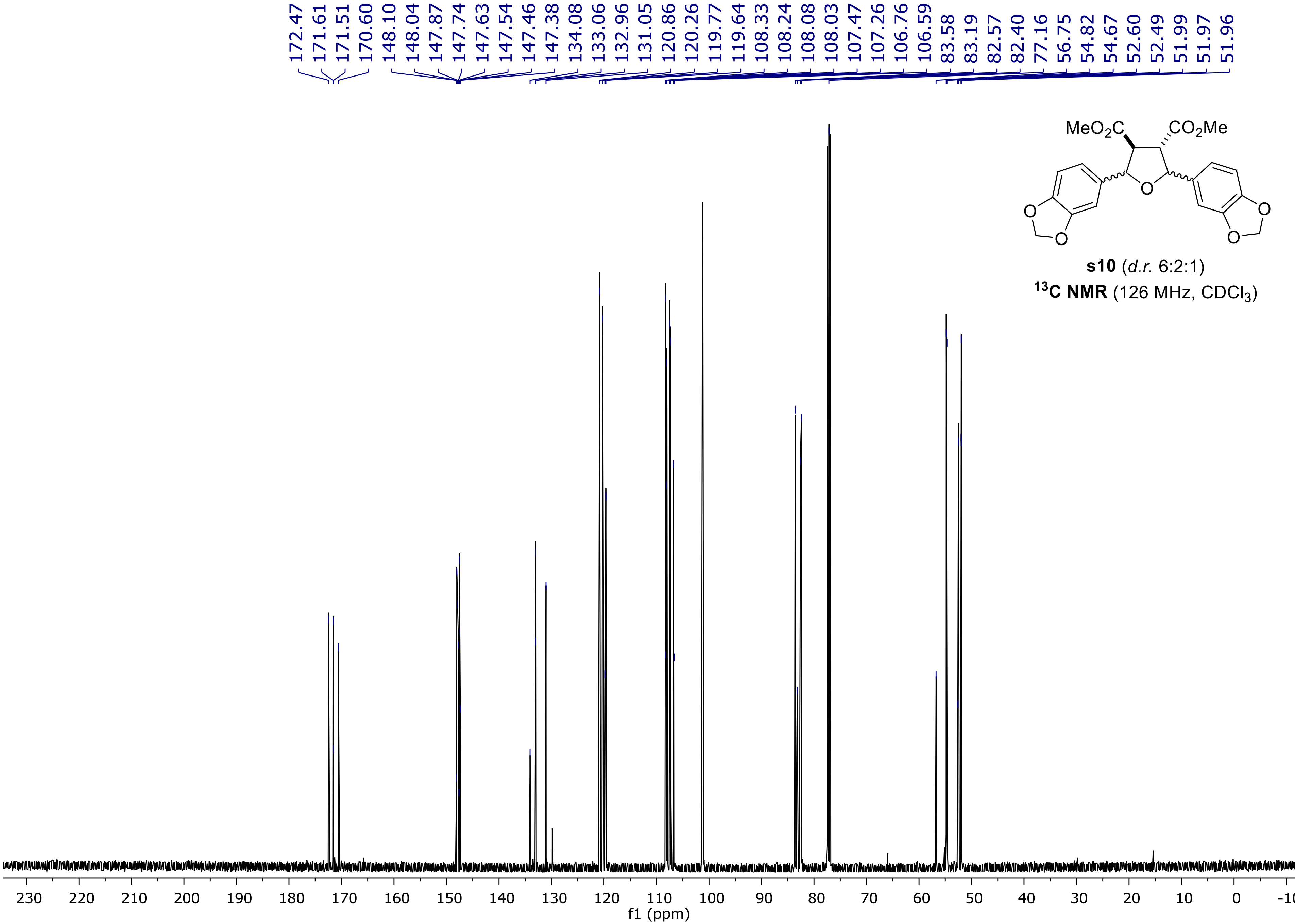

s10 (*d.r.* 6:2:1)

^{13}C NMR (126 MHz, $CDCl_3$)

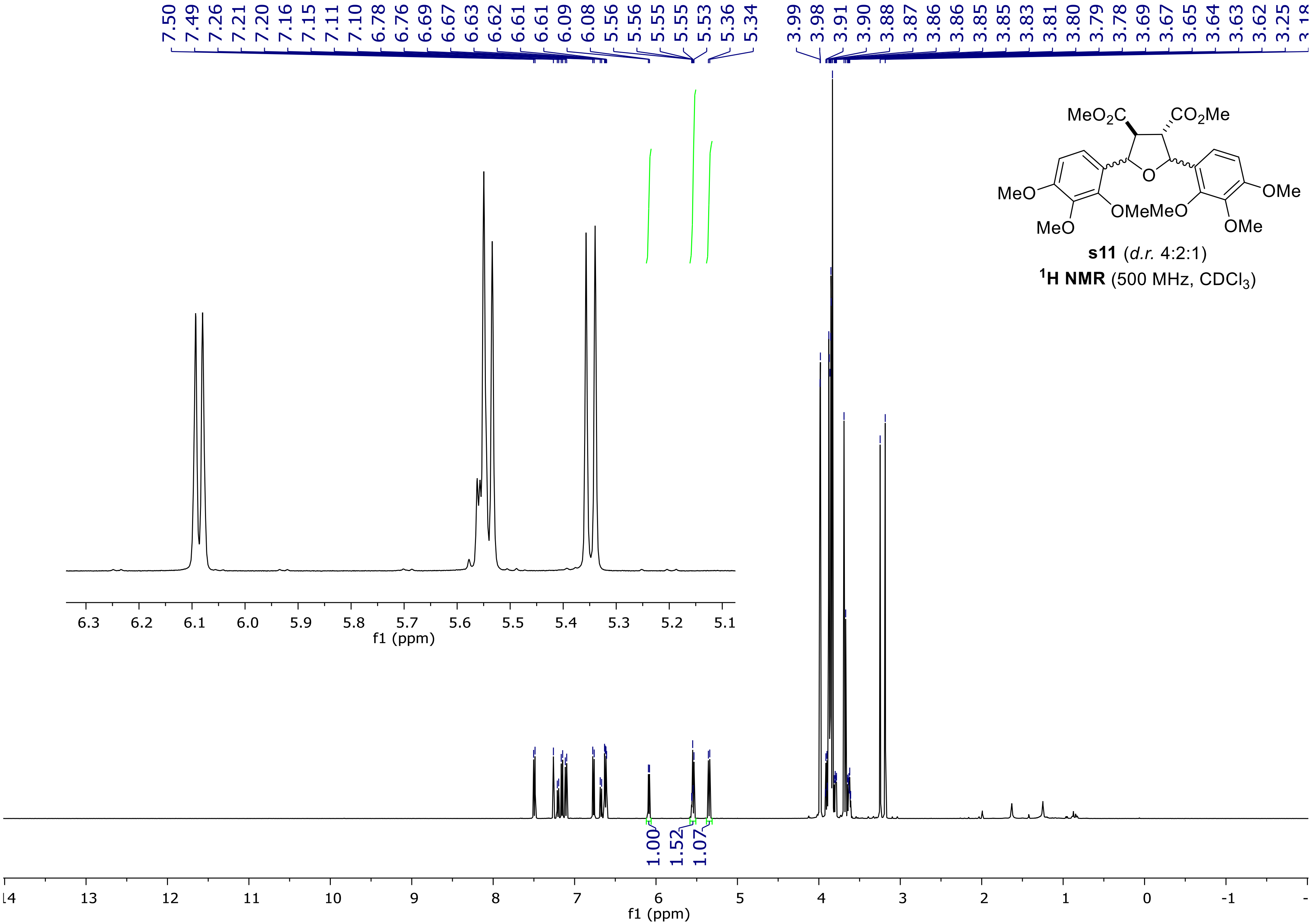

s11 (*d.r.* 4:2:1)

^{1}H NMR (500 MHz, $CDCl_3$)

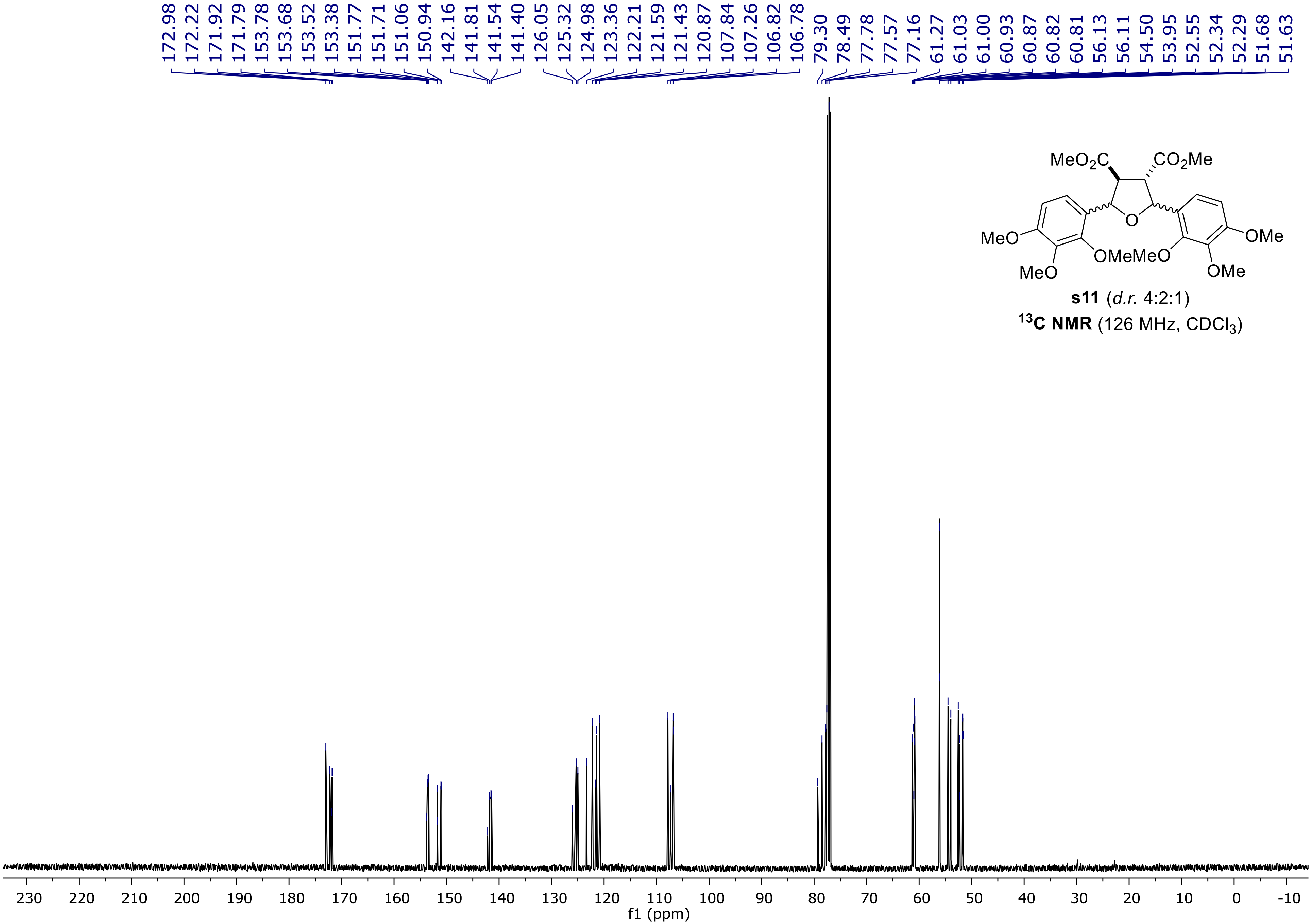

s11 (*d.r.* 4:2:1)

^{13}C NMR (126 MHz, $CDCl_3$)

Alfonzo 71

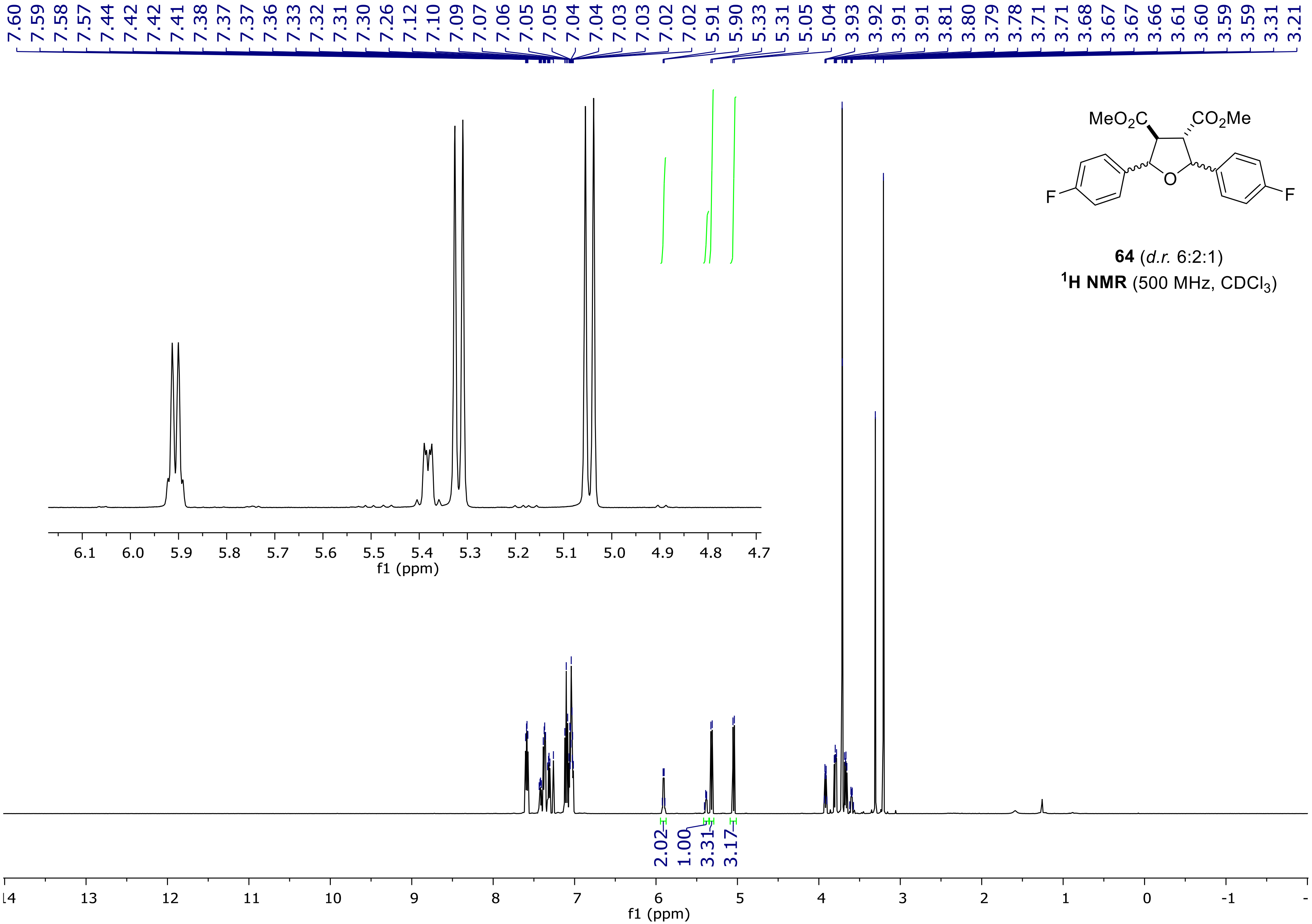

64 (*d.r.* 6:2:1)
^{1}H NMR (500 MHz, $CDCl_3$)

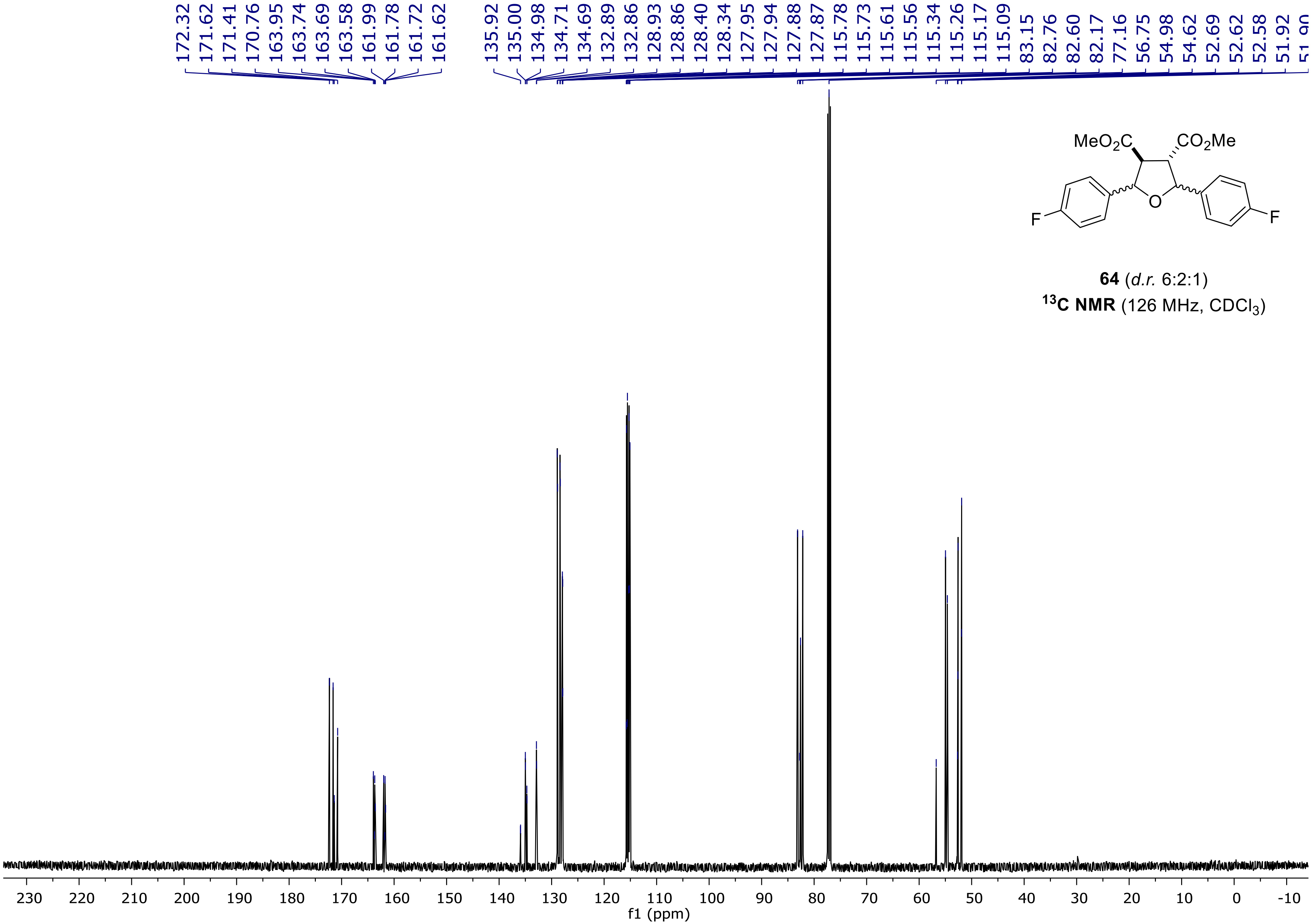

64 (*d.r.* 6:2:1)

^{13}C NMR (126 MHz, $CDCl_3$)

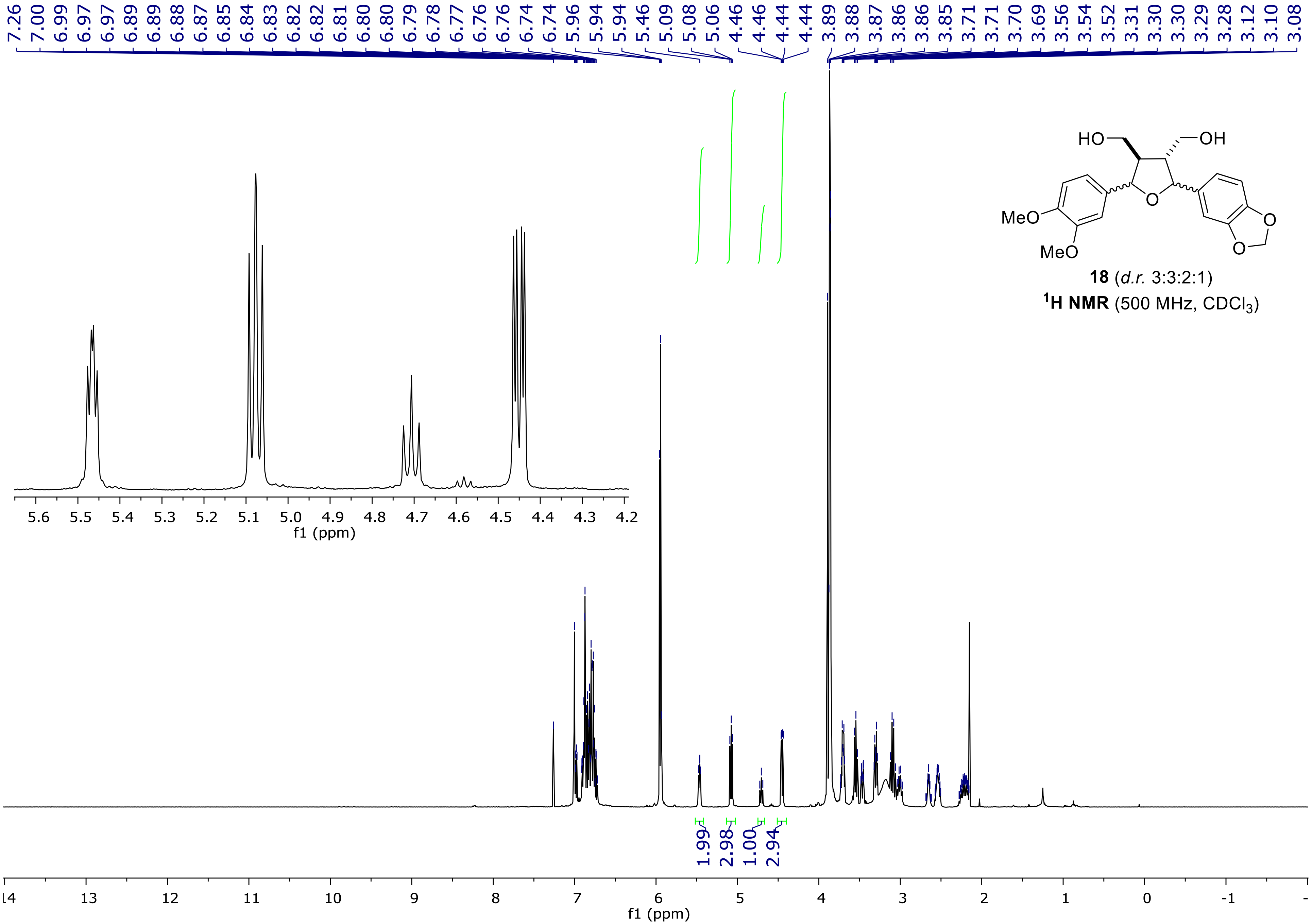

18 (*d.r.* 3:3:2:1)

^{1}H NMR (500 MHz, $CDCl_3$)

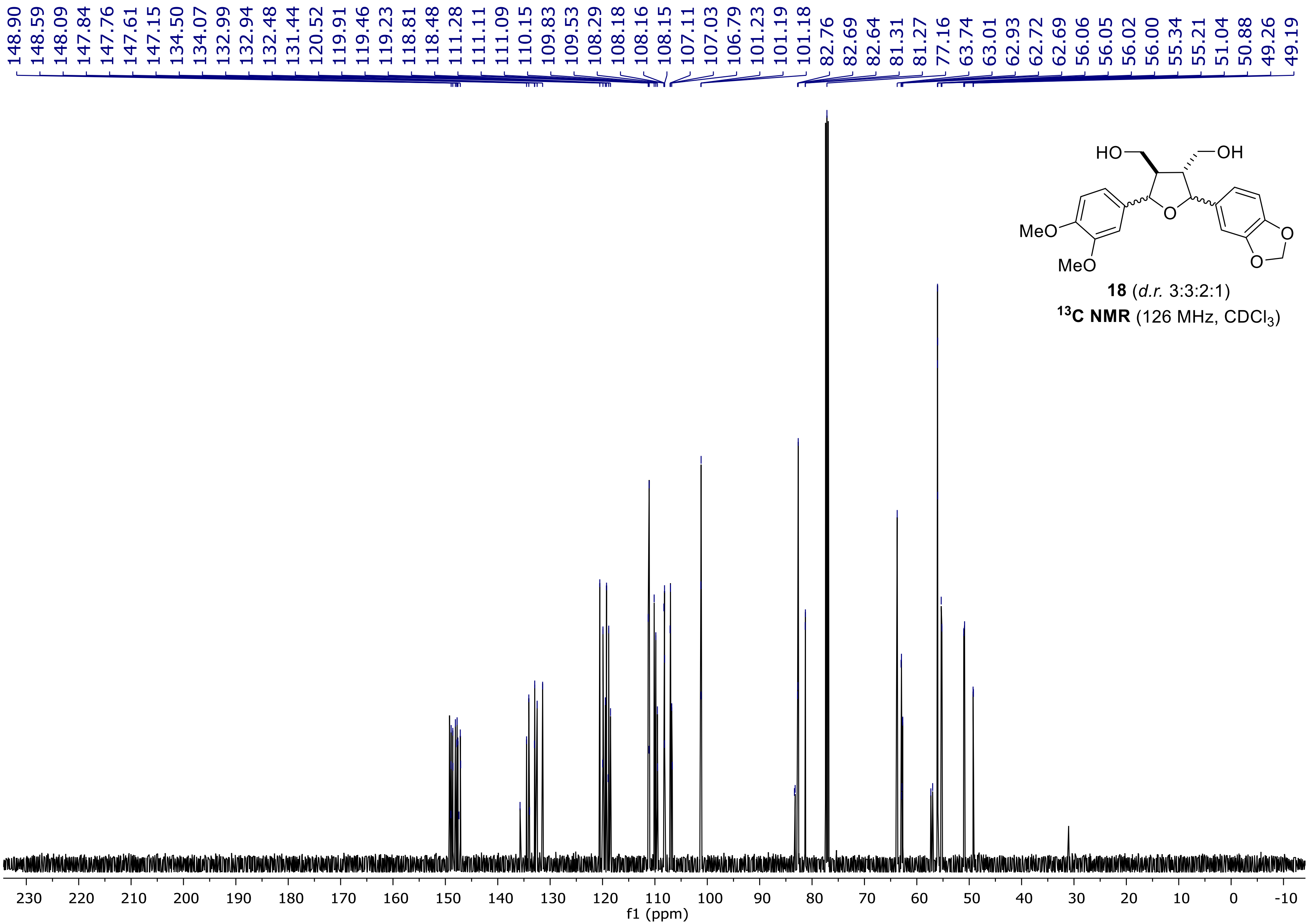
148.90
148.59
148.09
147.84
147.76
147.61
147.15
134.50
134.07
132.99
132.94
132.48
131.44
120.52
119.91
119.46
119.23
118.81
118.48
111.28
111.11
111.09
110.15
109.83
109.53
108.29
108.18
108.16
108.15
107.11
107.03
106.79
101.23
101.19
101.18
82.76
82.69
82.64
81.31
81.27
77.16
63.74
63.01
62.93
62.72
62.69
56.06
56.05
56.02
56.00
55.34
55.21
51.04
50.88
49.26
49.19
HO
OH
MeO
MeO
O
O
O
18 (*d.r.* 3:3:2:1)
^{13}C NMR (126 MHz, $CDCl_3$)
230
220
210
200
190
180
170
160
150
140
130
120
110
100
90
80
70
60
50
40
30
20
10
0
-10
f1 (ppm)

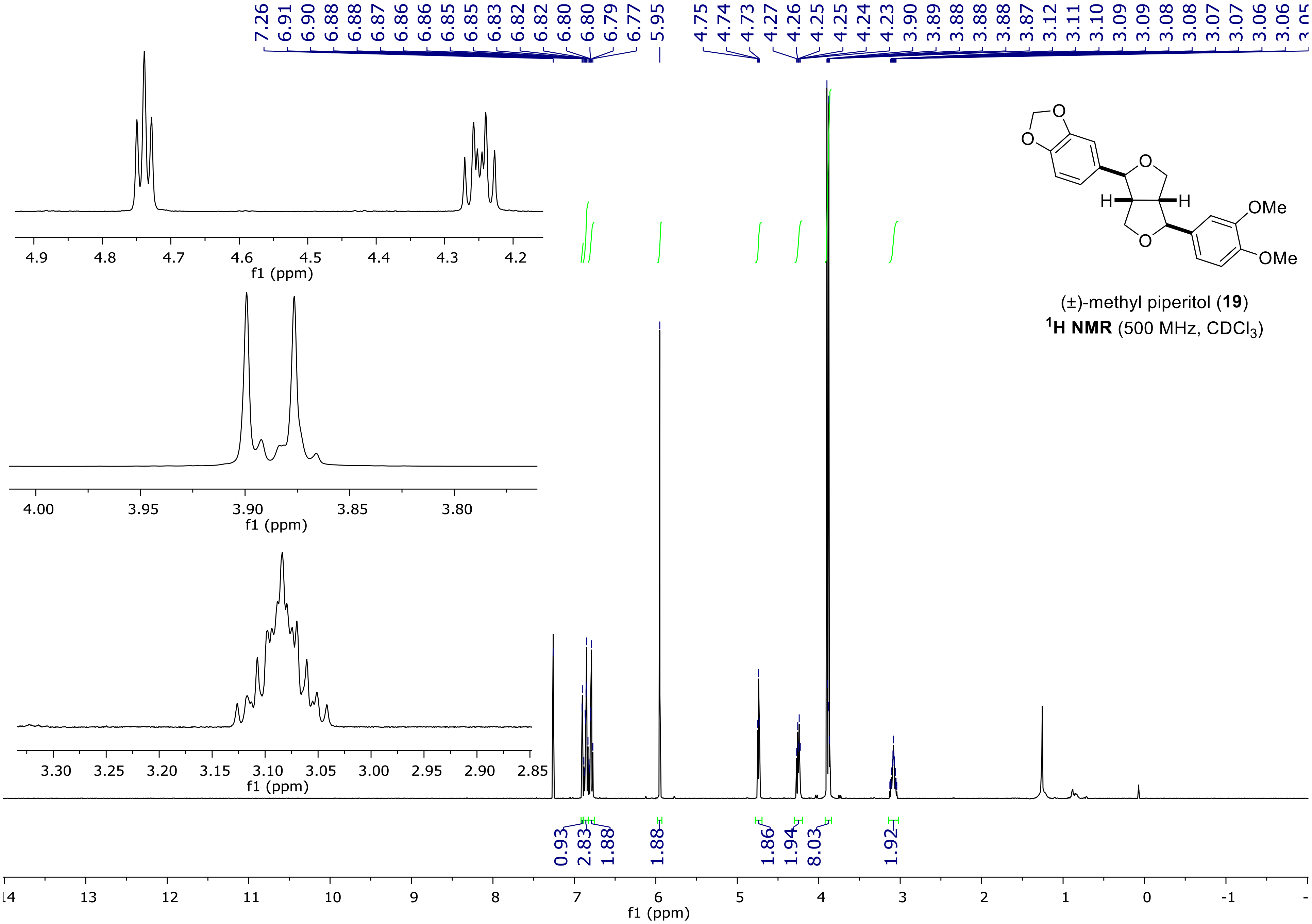

(±)-methyl piperitol (**19**)

^{1}H NMR (500 MHz, $CDCl_3$)

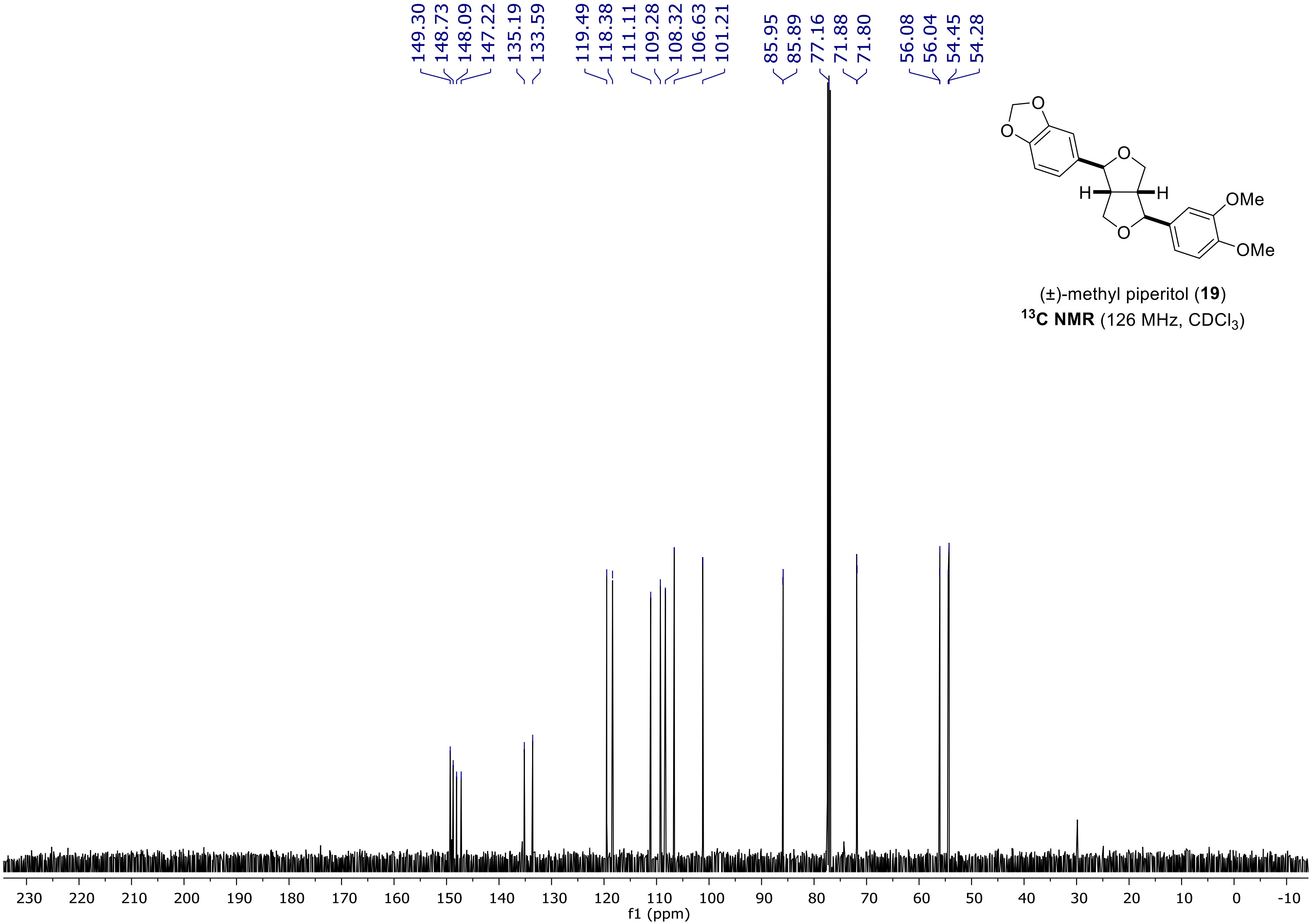

(±)-methyl piperitol (**19**)

^{13}C NMR (126 MHz, $CDCl_3$)

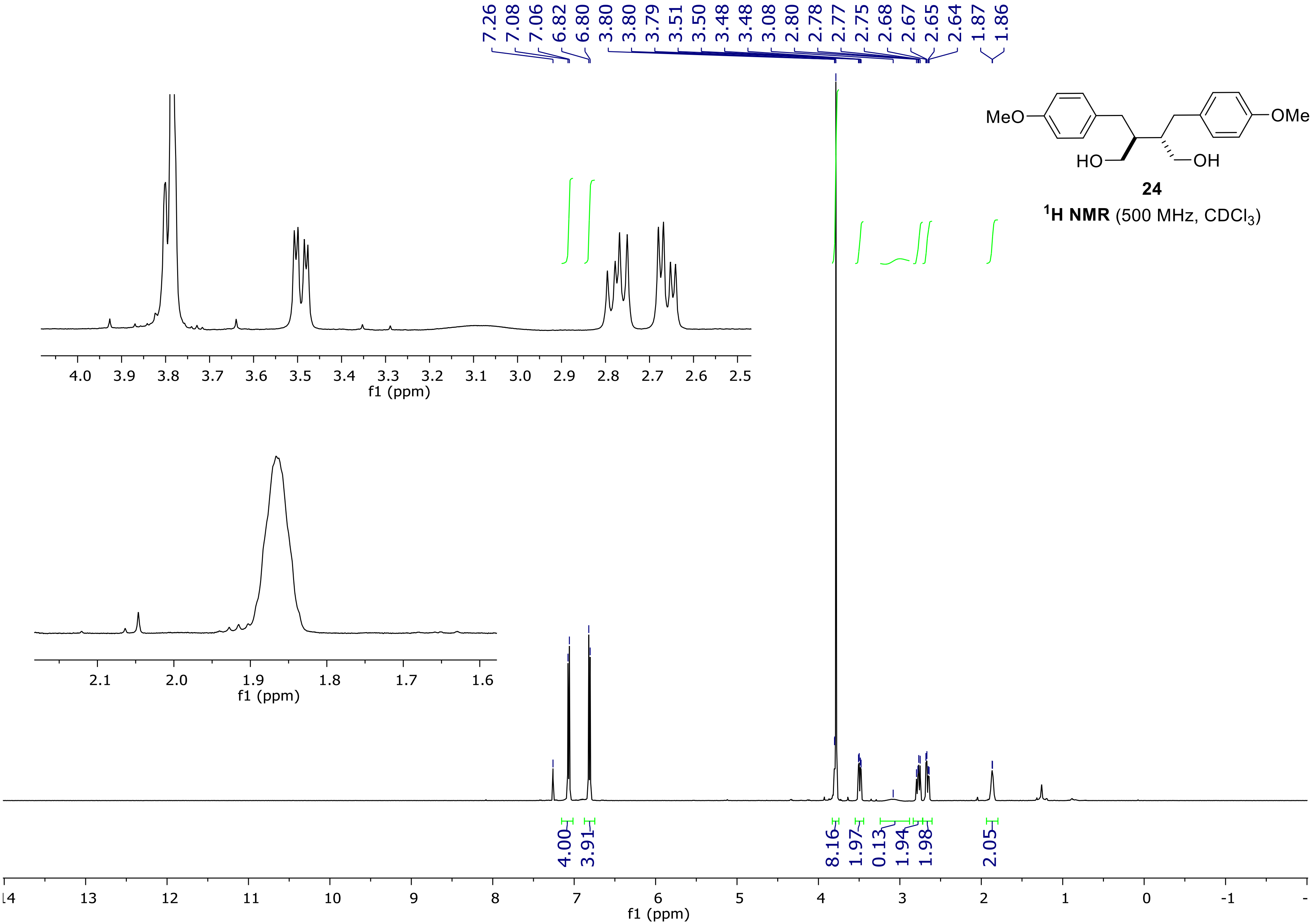

24

^{1}H NMR (500 MHz, $CDCl_3$)

Alfonzo 78

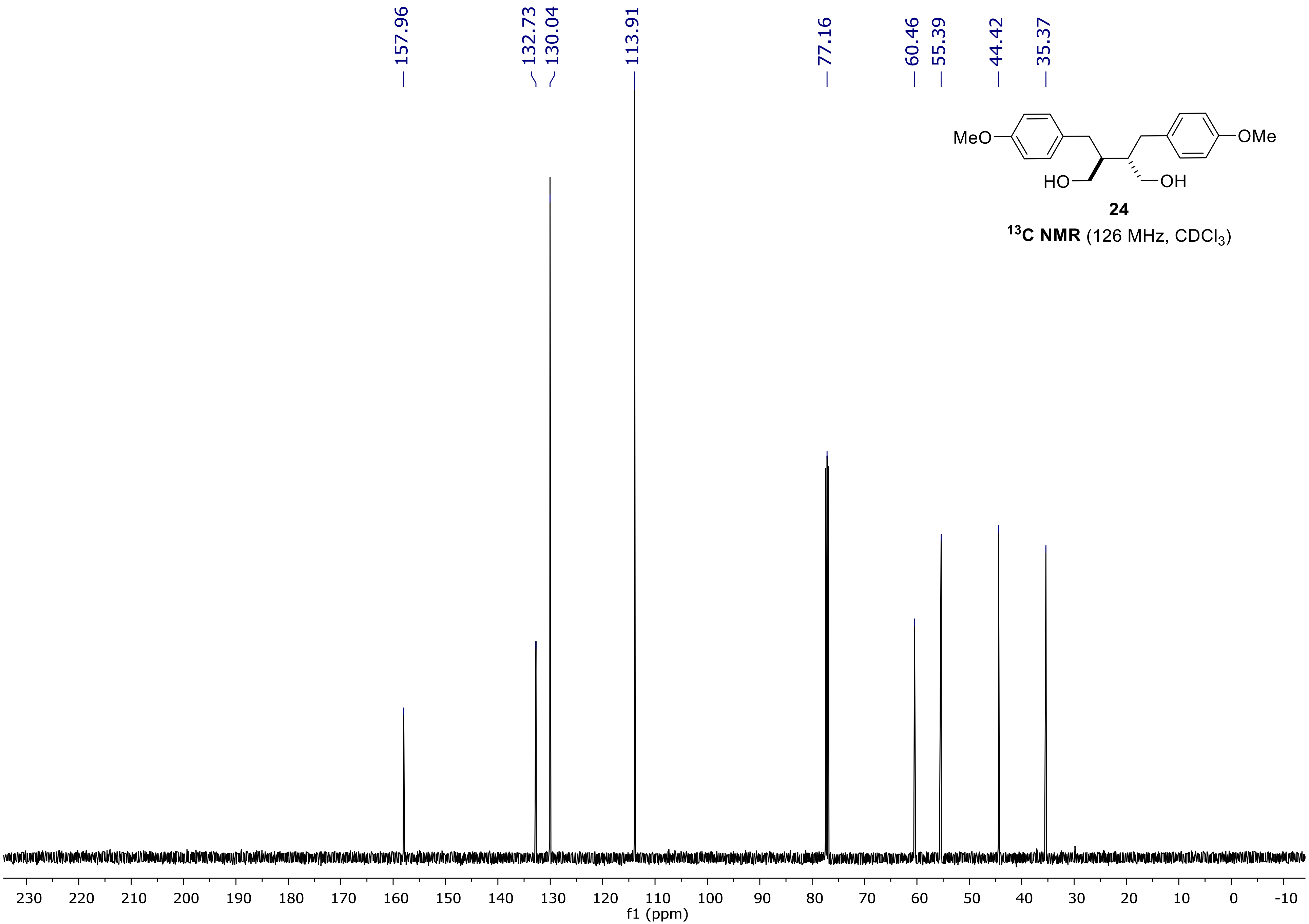
157.96
132.73
130.04
113.91
77.16
60.46
55.39
44.42
35.37
MeO
OMe
HO
OH
24
13C NMR (126 MHz, CDCl3)
230 220 210 200 190 180 170 160 150 140 130 120 110 100 90 80 70 60 50 40 30 20 10 0 -10
f1 (ppm)

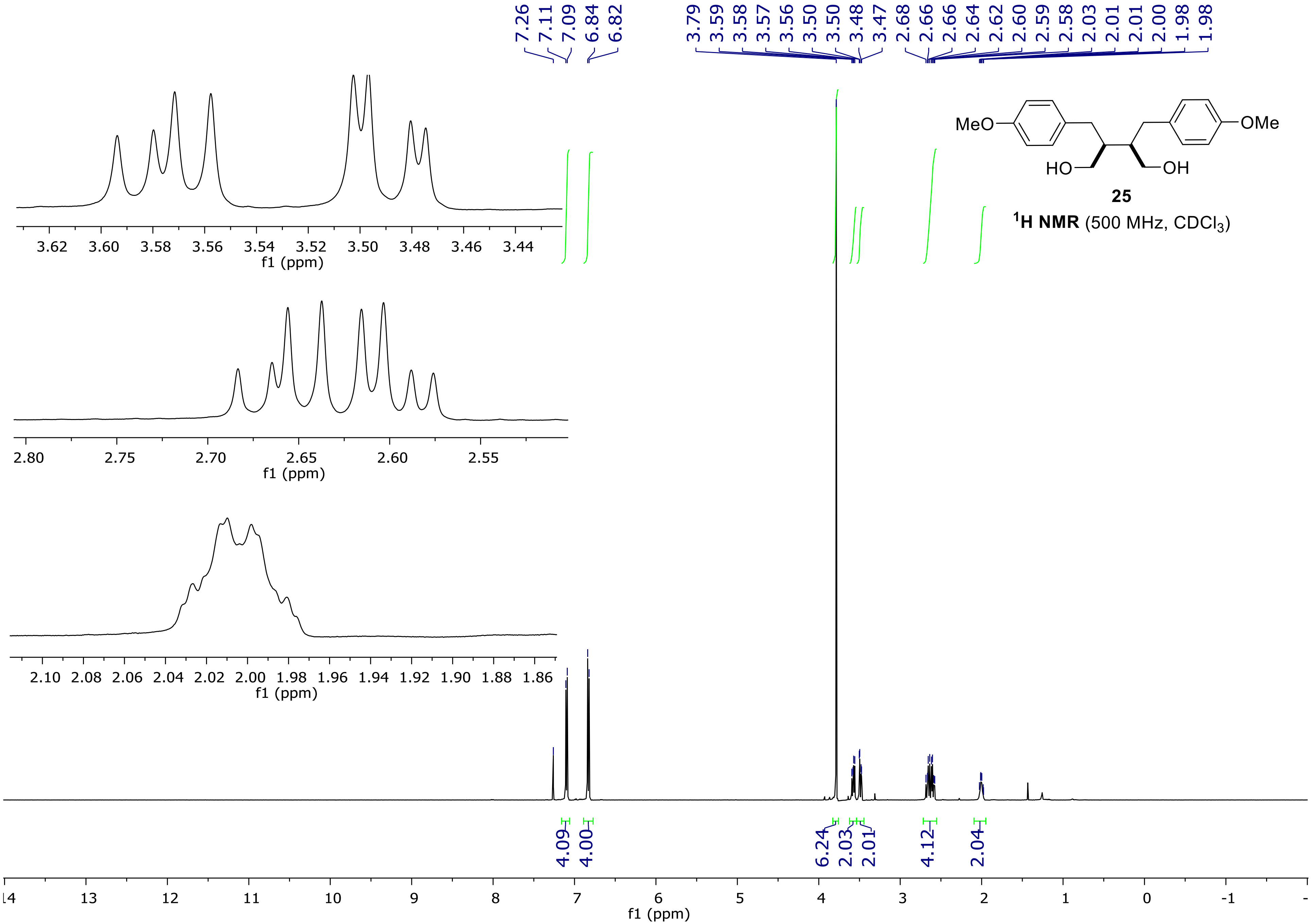

25

^{1}**H NMR** (500 MHz, $CDCl_3$)

Alfonzo 80

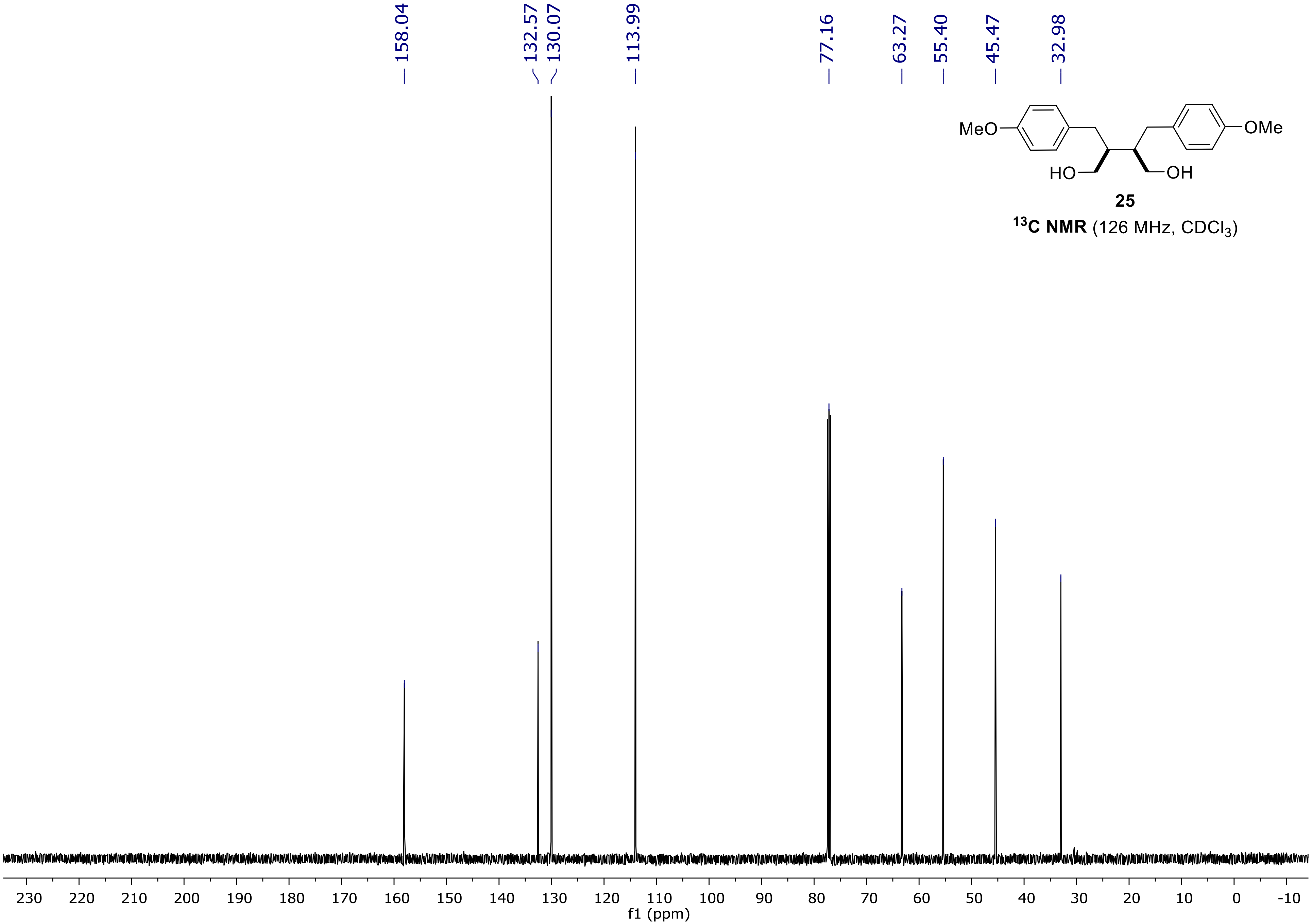

25

^{13}C NMR (126 MHz, $CDCl_3$)

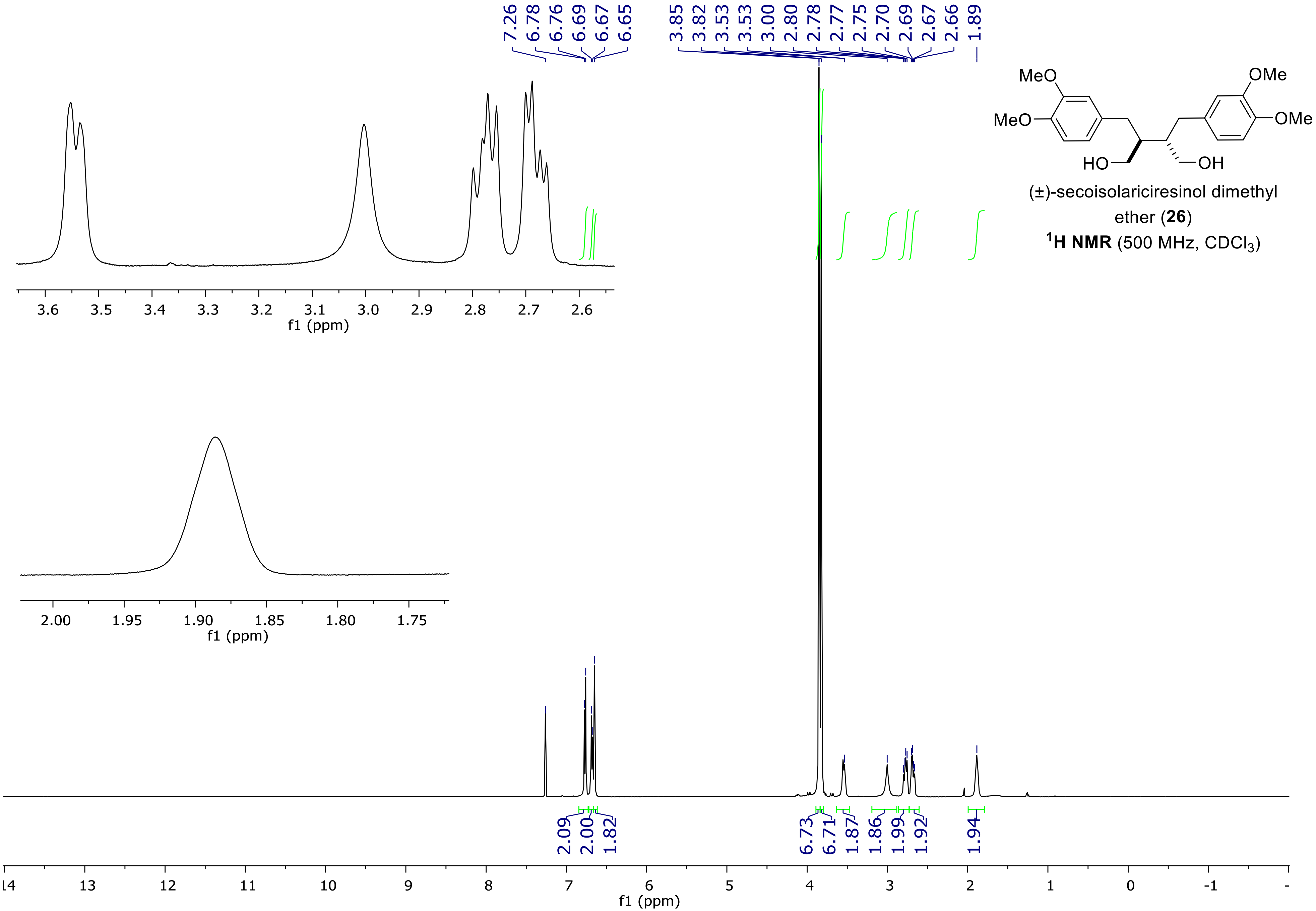

(±)-secoisolariciresinol dimethyl ether (**26**)

^{1}H NMR (500 MHz, $CDCl_3$)

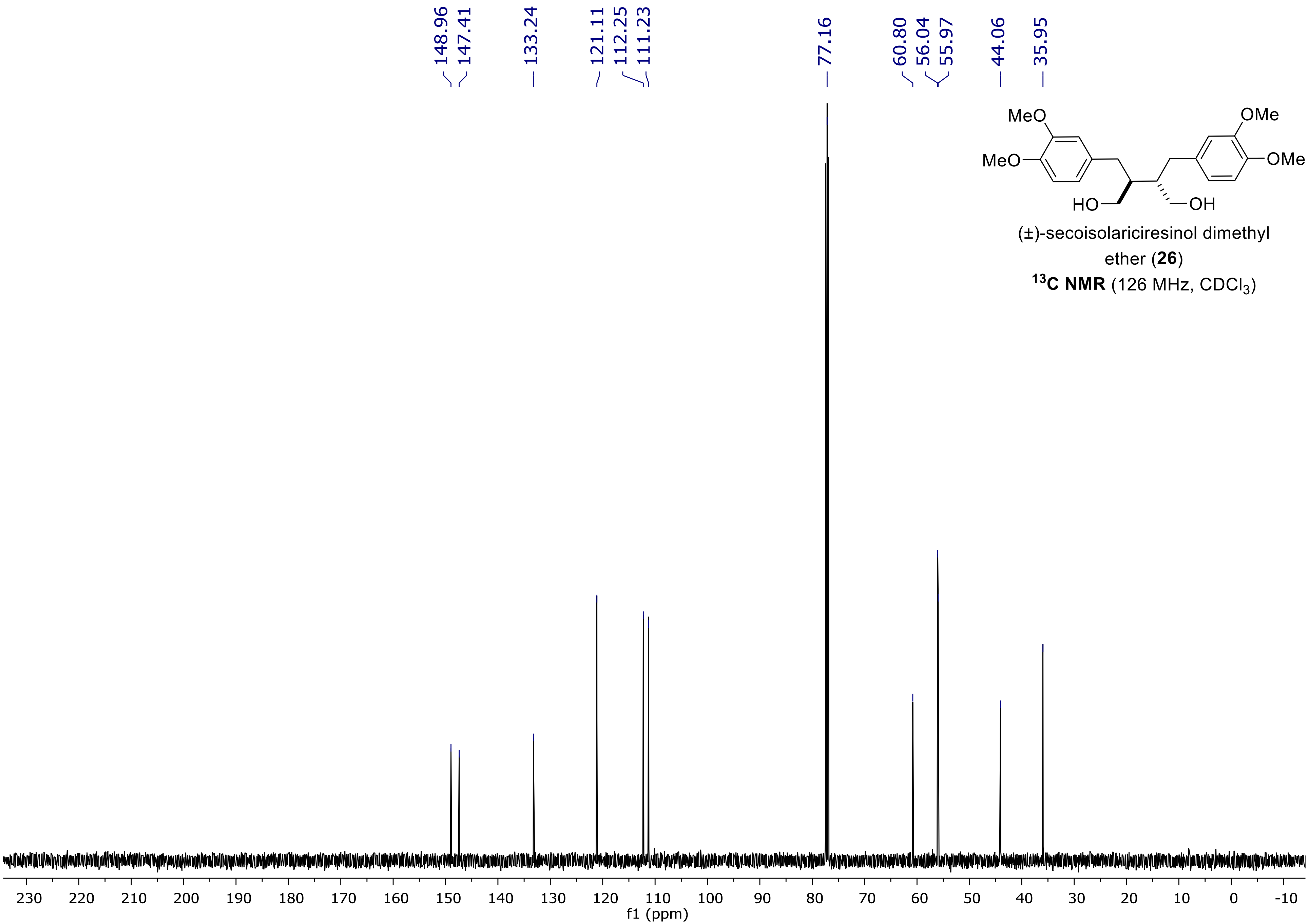

(±)-secoisolariciresinol dimethyl ether (**26**)

^{13}C NMR (126 MHz, $CDCl_3$)

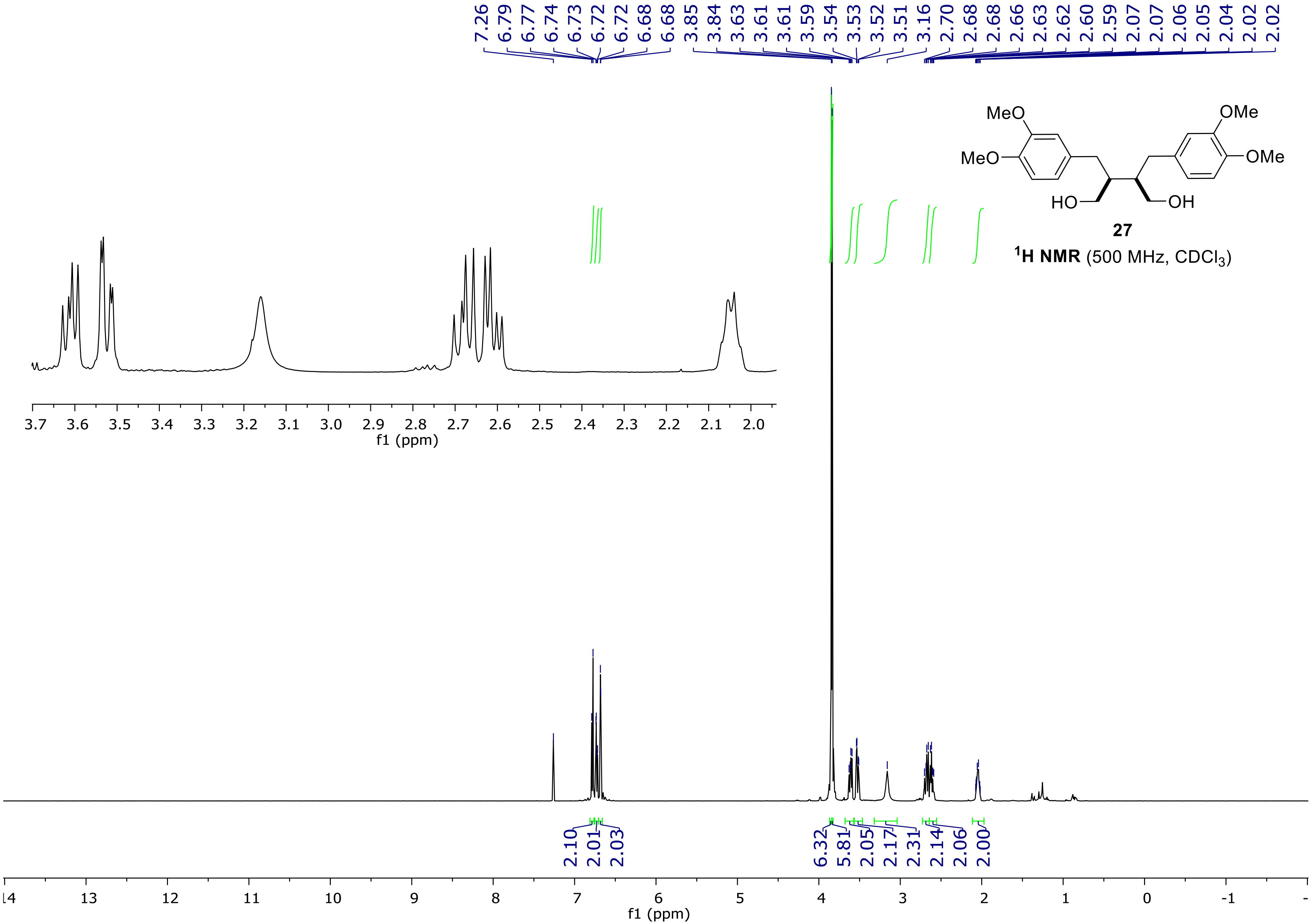
7.26 6.79 6.77 6.74 6.73 6.72 6.72 6.68 6.68 3.85 3.84 3.63 3.61 3.61 3.59 3.54 3.53 3.52 3.51 3.16 2.70 2.68 2.68 2.66 2.63 2.62 2.60 2.59 2.07 2.07 2.06 2.05 2.04 2.02 2.02
MeO
MeO
OMe
OMe
HO
OH
27
^{1}H NMR (500 MHz, $CDCl_3$)
3.7 3.6 3.5 3.4 3.3 3.2 3.1 3.0 2.9 2.8 2.7 2.6 2.5 2.4 2.3 2.2 2.1 2.0
f1 (ppm)
2.10 2.01 2.03 6.32 5.81 2.05 2.17 2.31 2.14 2.06 2.00
13 12 11 10 9 8 7 6 5 4 3 2 1 0 -1
f1 (ppm)

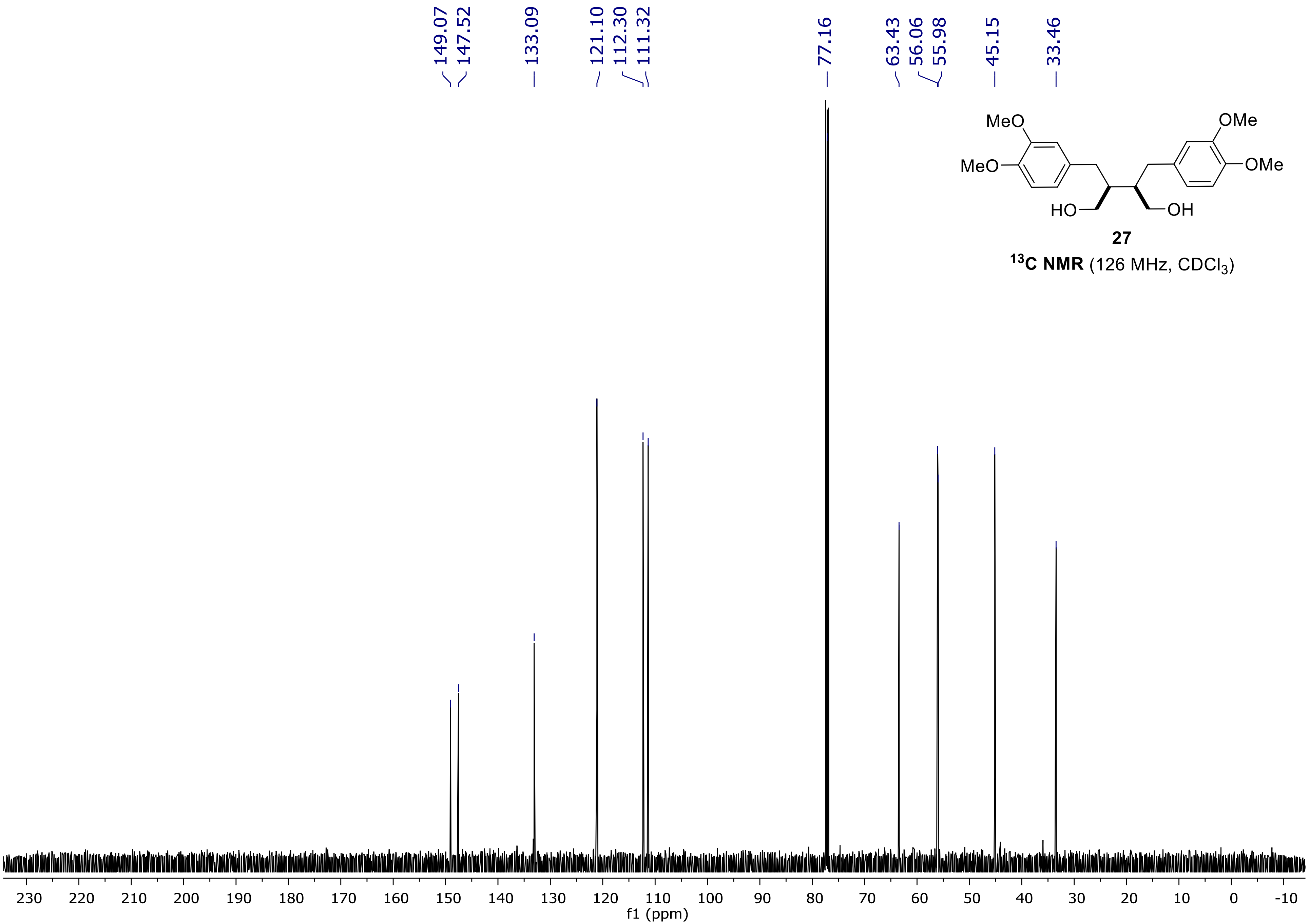

27

^{13}C NMR (126 MHz, $CDCl_3$)

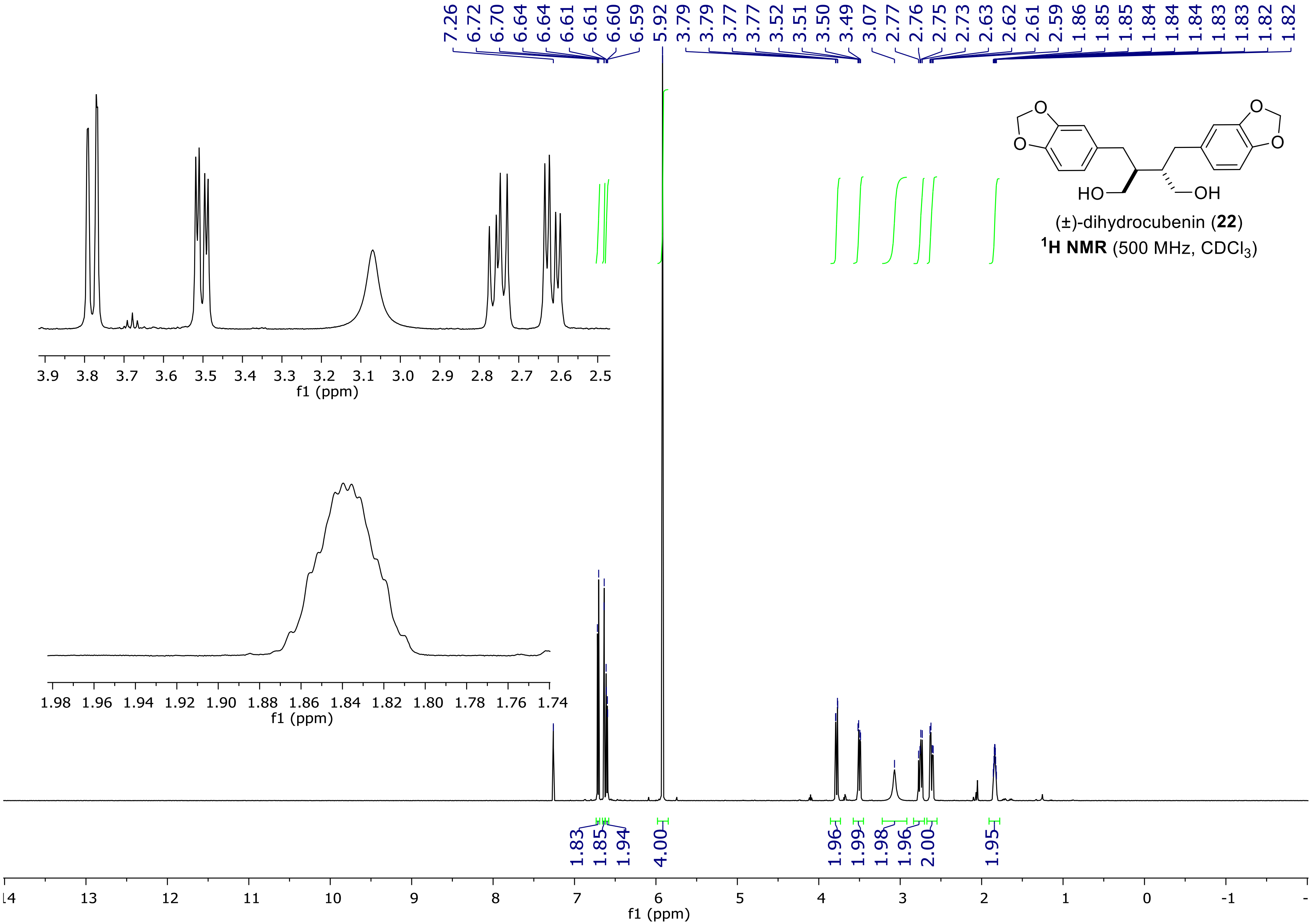

7.26 6.72 6.70 6.64 6.64 6.61 6.61 6.60 6.59 5.92 3.79 3.79 3.77 3.77 3.52 3.51 3.50 3.49 3.07 2.77 2.76 2.75 2.73 2.63 2.62 2.61 2.59 1.86 1.85 1.85 1.84 1.84 1.84 1.83 1.83 1.82 1.82
HO
OH
O
(±)-dihydrocubenin (22)
1H NMR (500 MHz, CDCl3)
3.9 3.8 3.7 3.6 3.5 3.4 3.3 3.2 3.1 3.0 2.9 2.8 2.7 2.6 2.5
f1 (ppm)
1.98 1.96 1.94 1.92 1.90 1.88 1.86 1.84 1.82 1.80 1.78 1.76 1.74
f1 (ppm)
1.83 1.85 1.94 4.00 1.96 1.99 1.98 1.96 2.00 1.95
13 12 11 10 9 8 7 6 5 4 3 2 1 0 -1
f1 (ppm)

Alfonzo 86

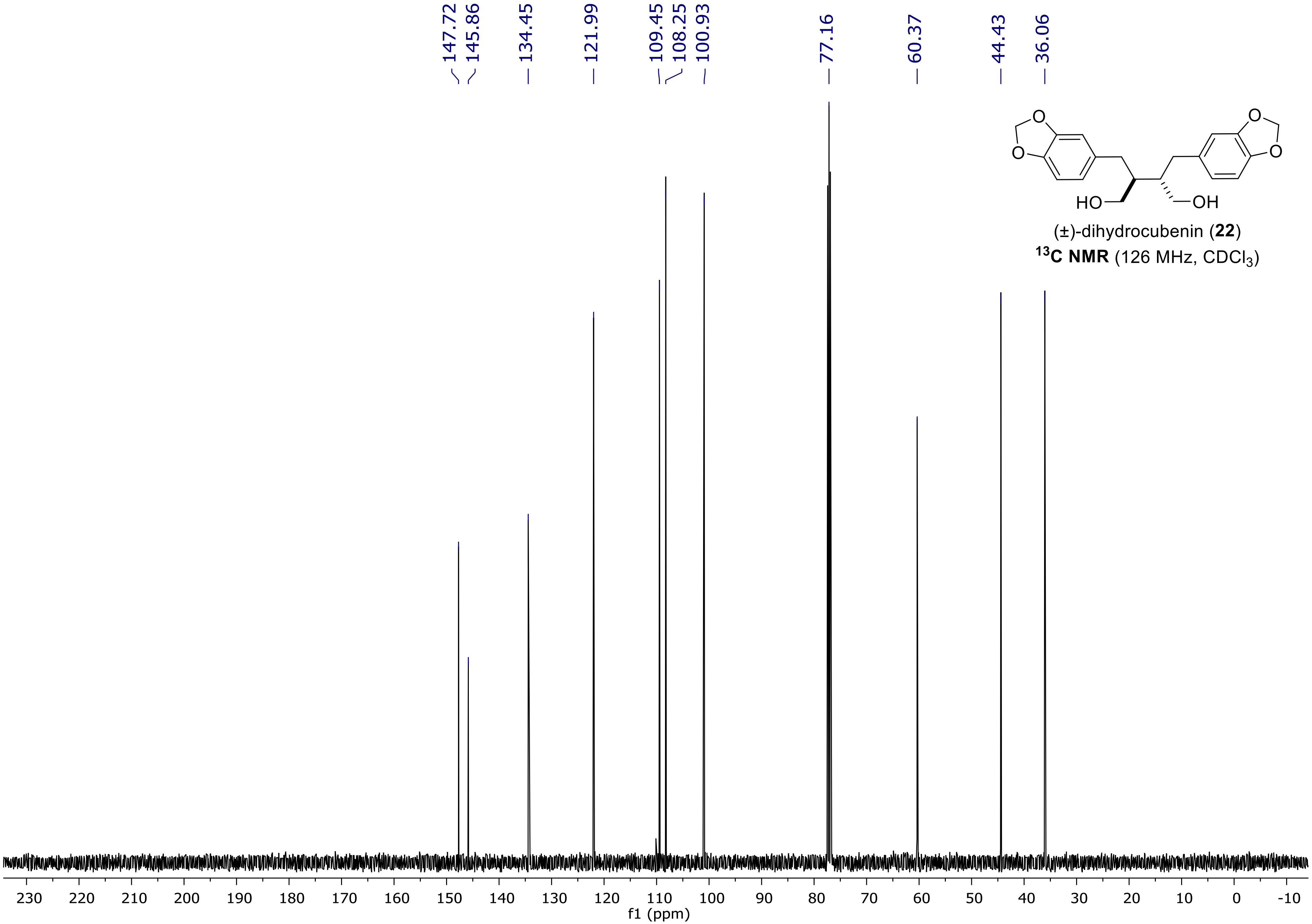

(±)-dihydrocubenin (**22**)

^{13}C NMR (126 MHz, $CDCl_3$)

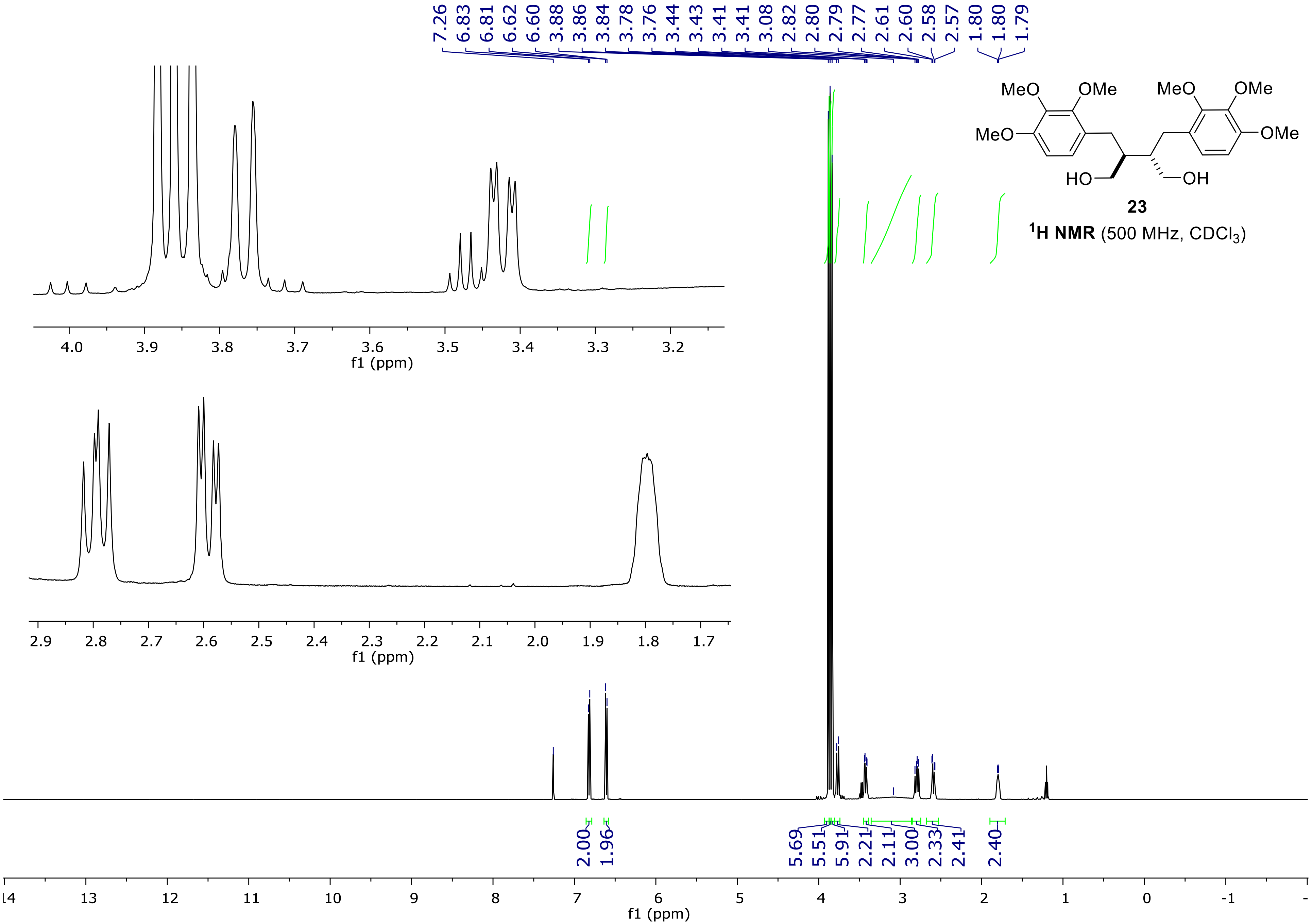

23

^{1}H NMR (500 MHz, $CDCl_3$)

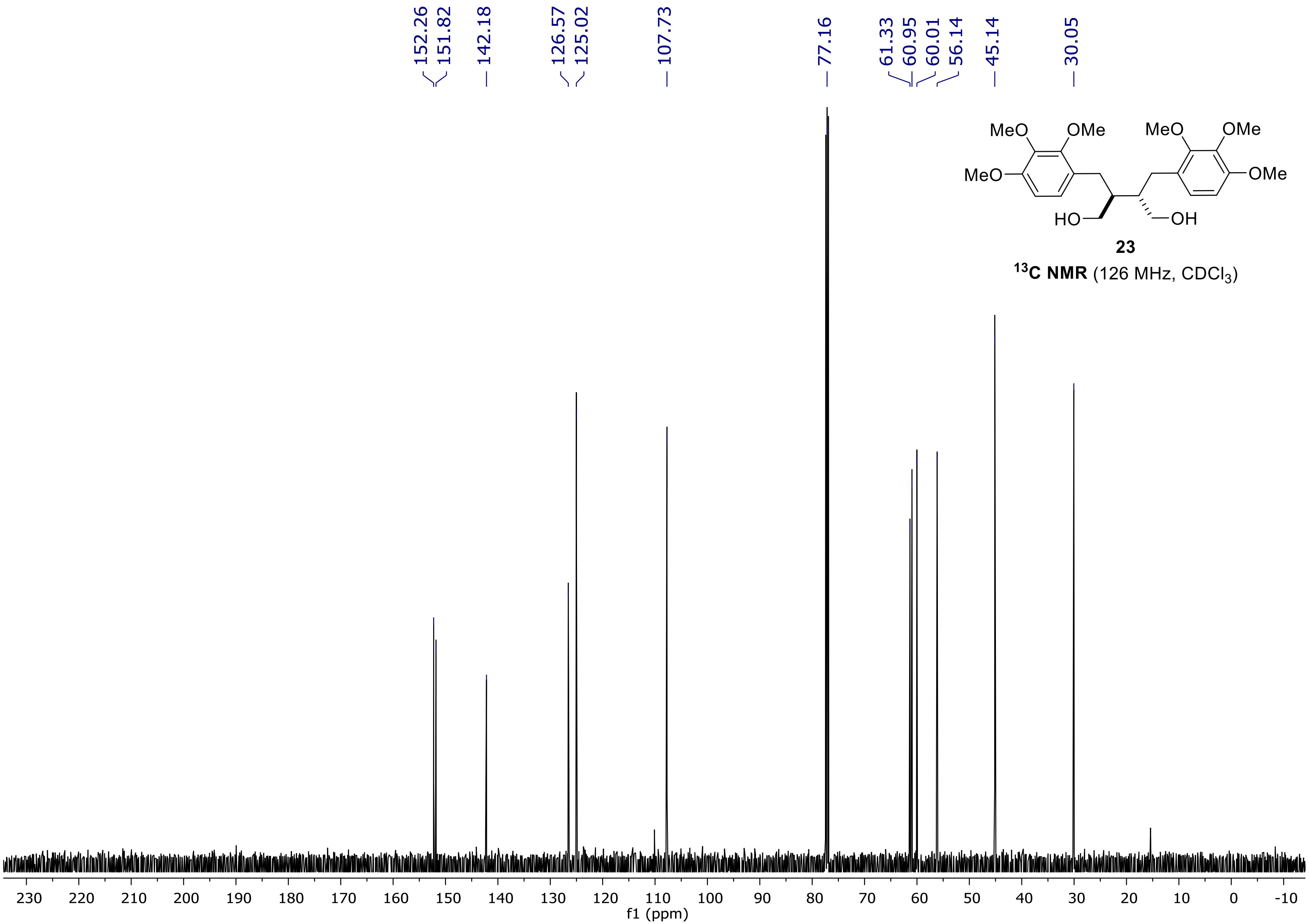
152.26
151.82
142.18
126.57
125.02
107.73
77.16
61.33
60.95
60.01
56.14
45.14
30.05
MeO
OMe
MeO
OMe
MeO
OMe
HO
OH
23
13C NMR (126 MHz, CDCl3)
230 220 210 200 190 180 170 160 150 140 130 120 110 100 90 80 70 60 50 40 30 20 10 0 -10
f1 (ppm)

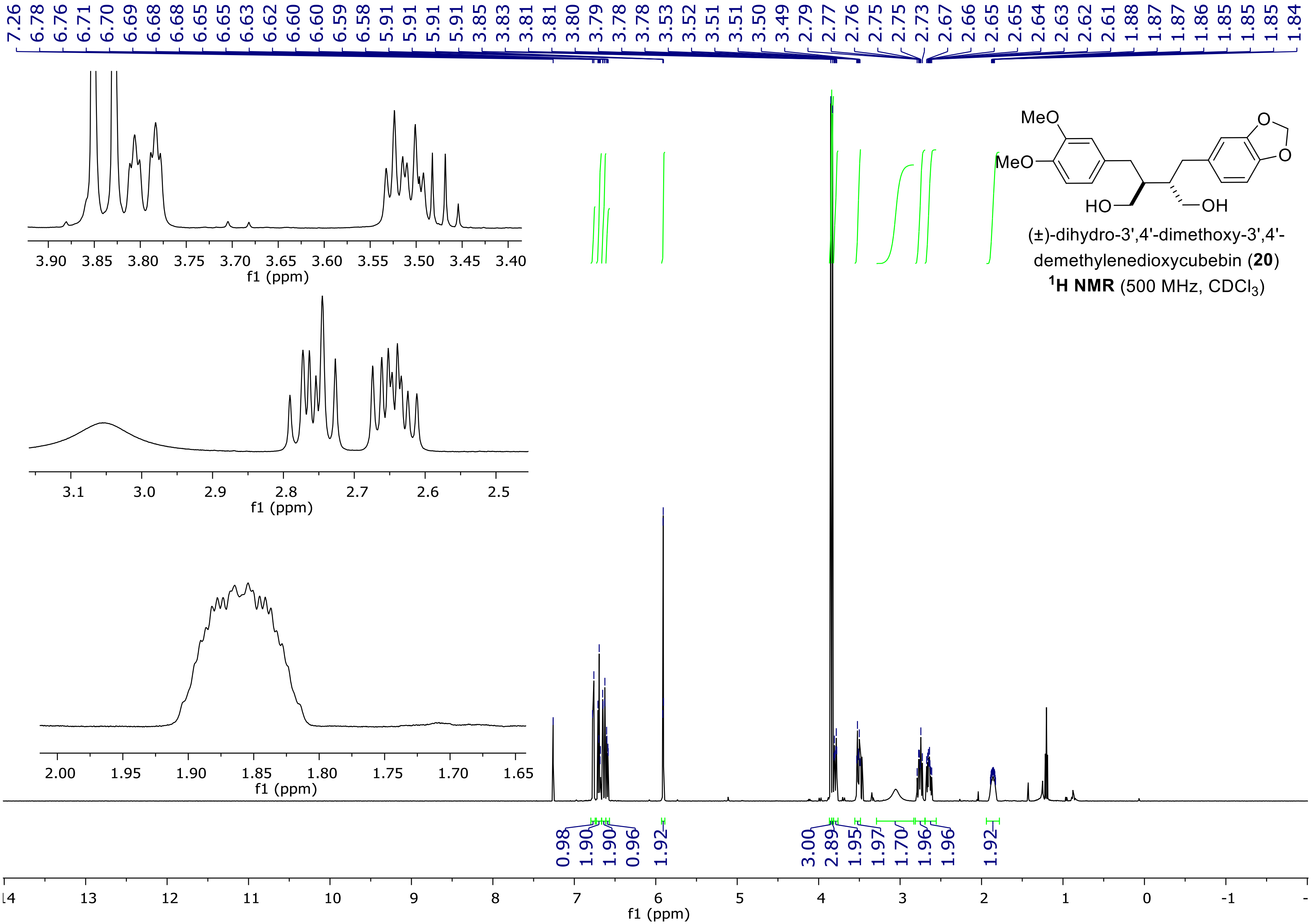

(±)-dihydro-3',4'-dimethoxy-3',4'-demethylenedioxycubebin (**20**)

^{1}H NMR (500 MHz, $CDCl_3$)

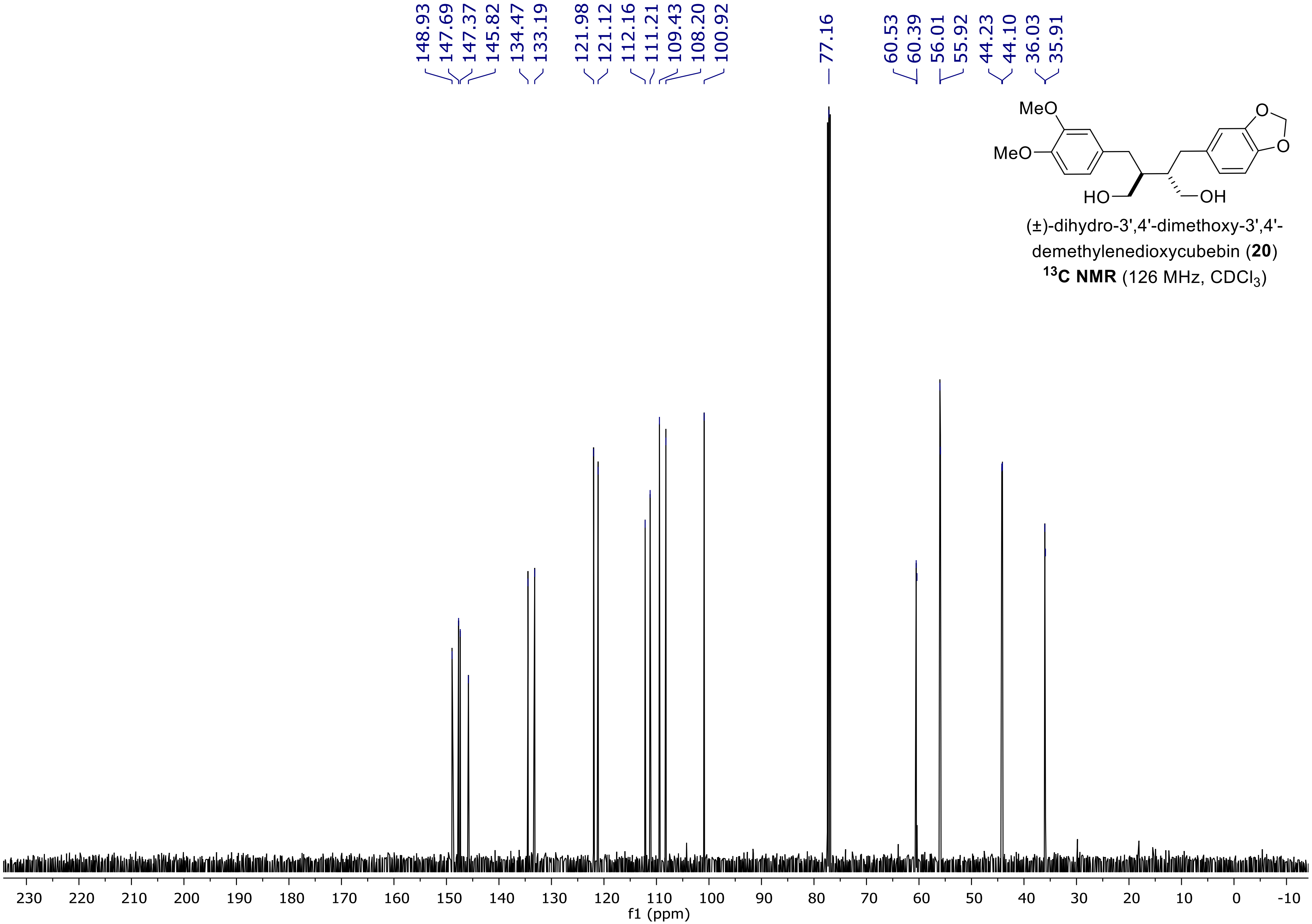

(±)-dihydro-3',4'-dimethoxy-3',4'-demethylenedioxycubebin (**20**)

^{13}C NMR (126 MHz, $CDCl_3$)

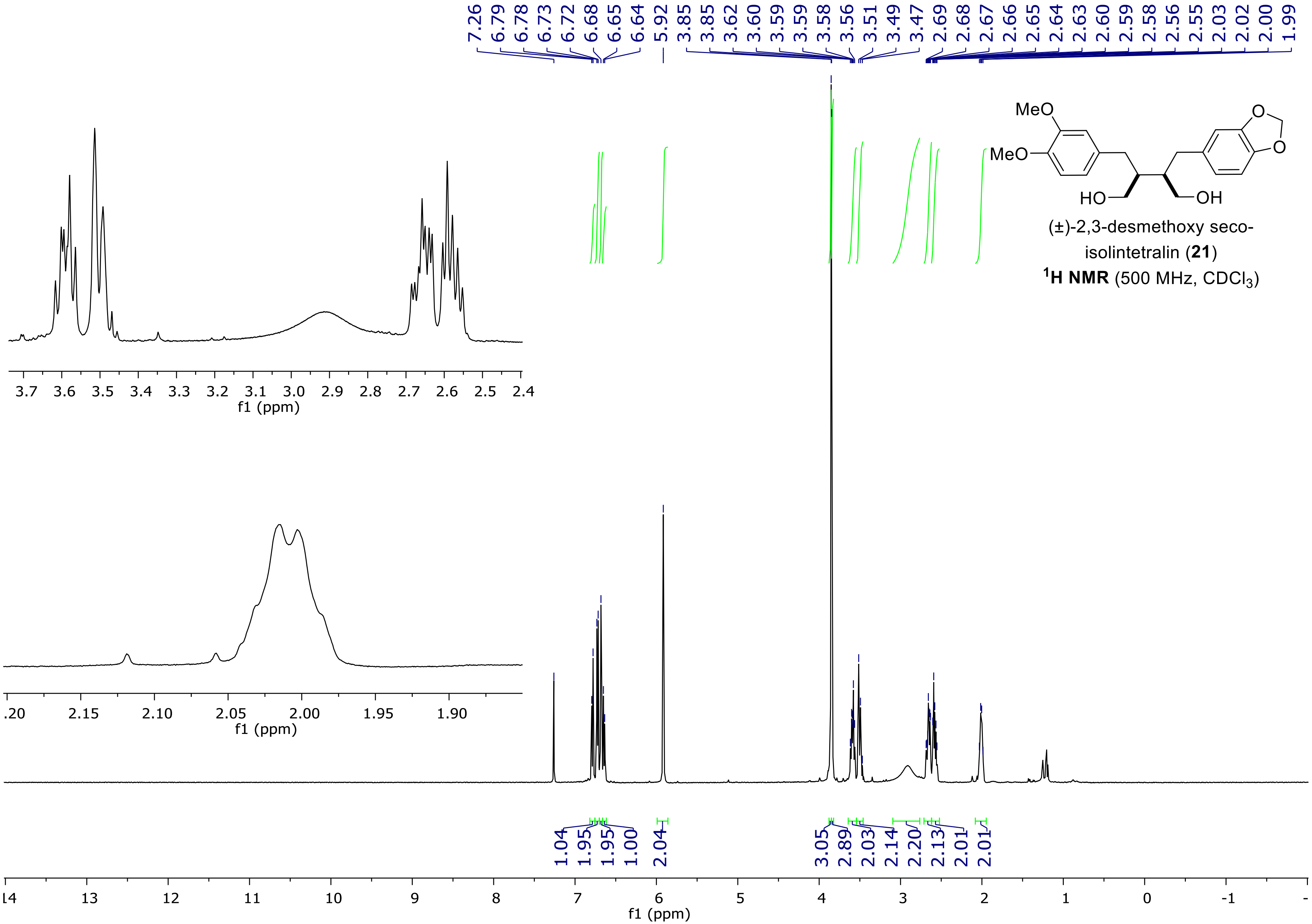

(±)-2,3-desmethoxy seco-isolintetralin (**21**)

^{1}**H NMR** (500 MHz, $CDCl_3$)

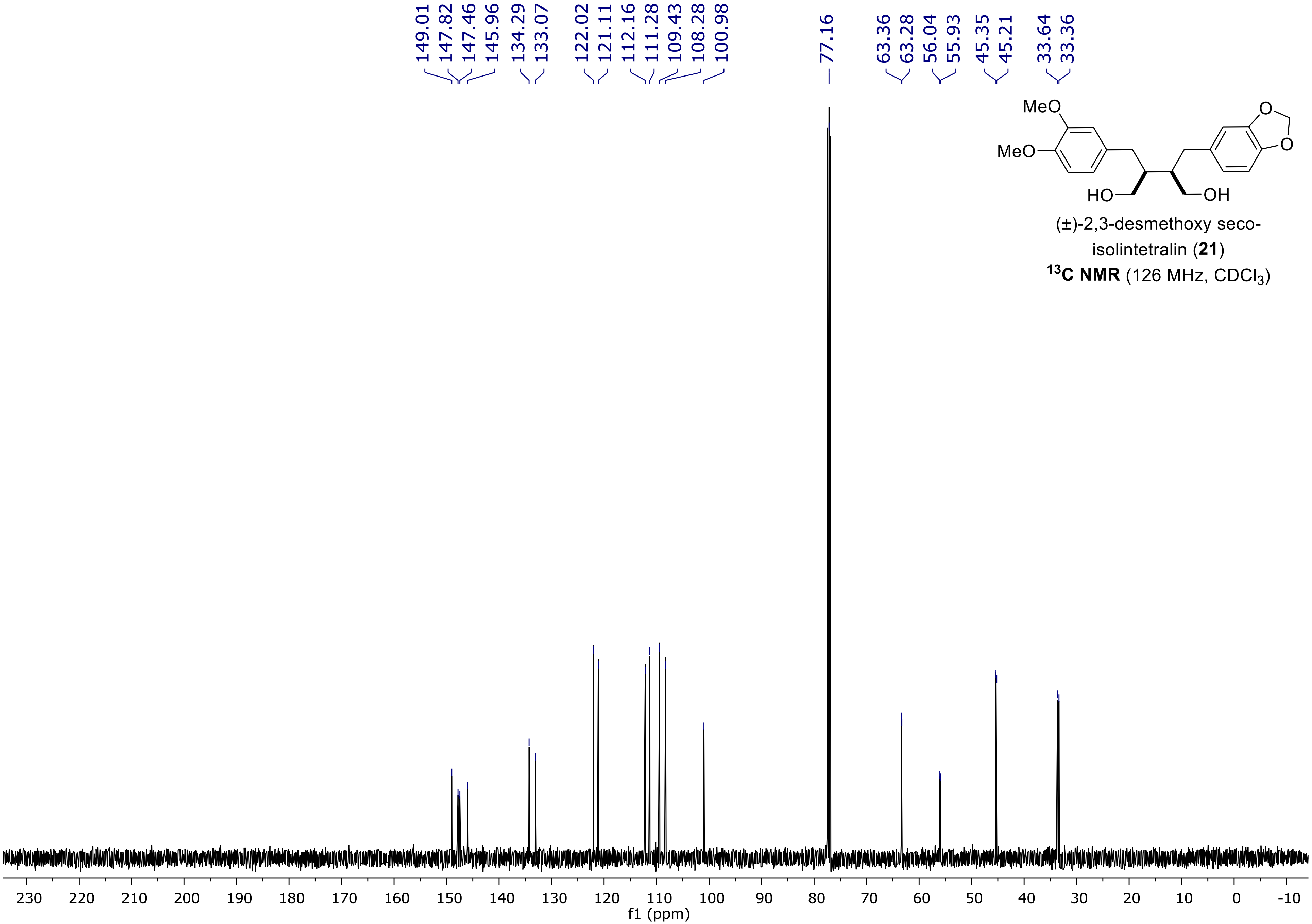

(±)-2,3-desmethoxy seco-isolintetralin (**21**)

^{13}C NMR (126 MHz, $CDCl_3$)

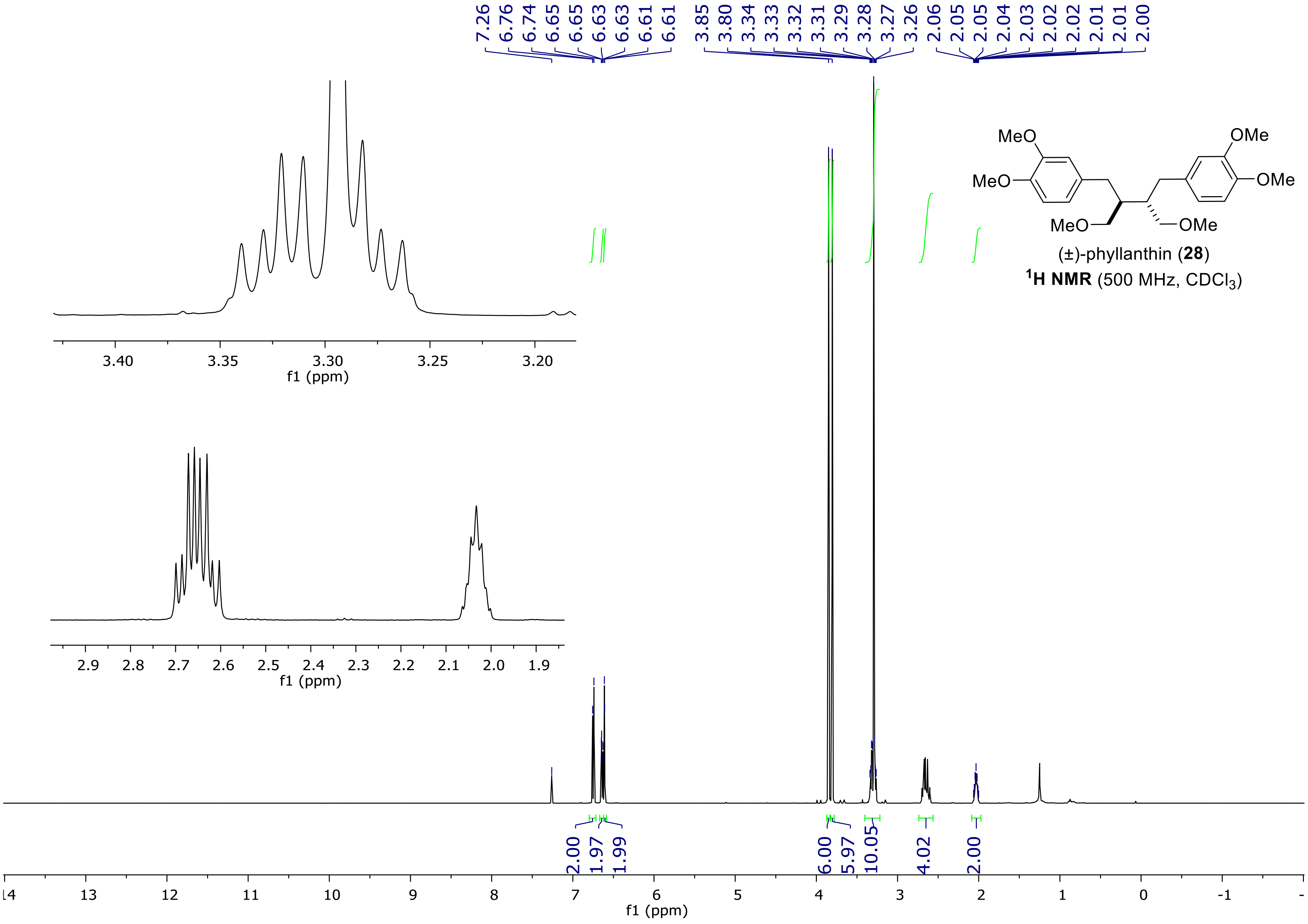
7.26 6.76 6.74 6.65 6.65 6.63 6.63 6.61 6.61 3.85 3.80 3.34 3.33 3.32 3.31 3.29 3.28 3.27 3.26 2.06 2.05 2.05 2.04 2.03 2.02 2.02 2.01 2.01 2.00
MeO
MeO
OMe
OMe
MeO
OMe
(±)-phyllanthin (28)
1H NMR (500 MHz, CDCl3)
2.00 1.97 1.99 6.00 5.97 10.05 4.02 2.00
f1 (ppm)

Alfonzo 94

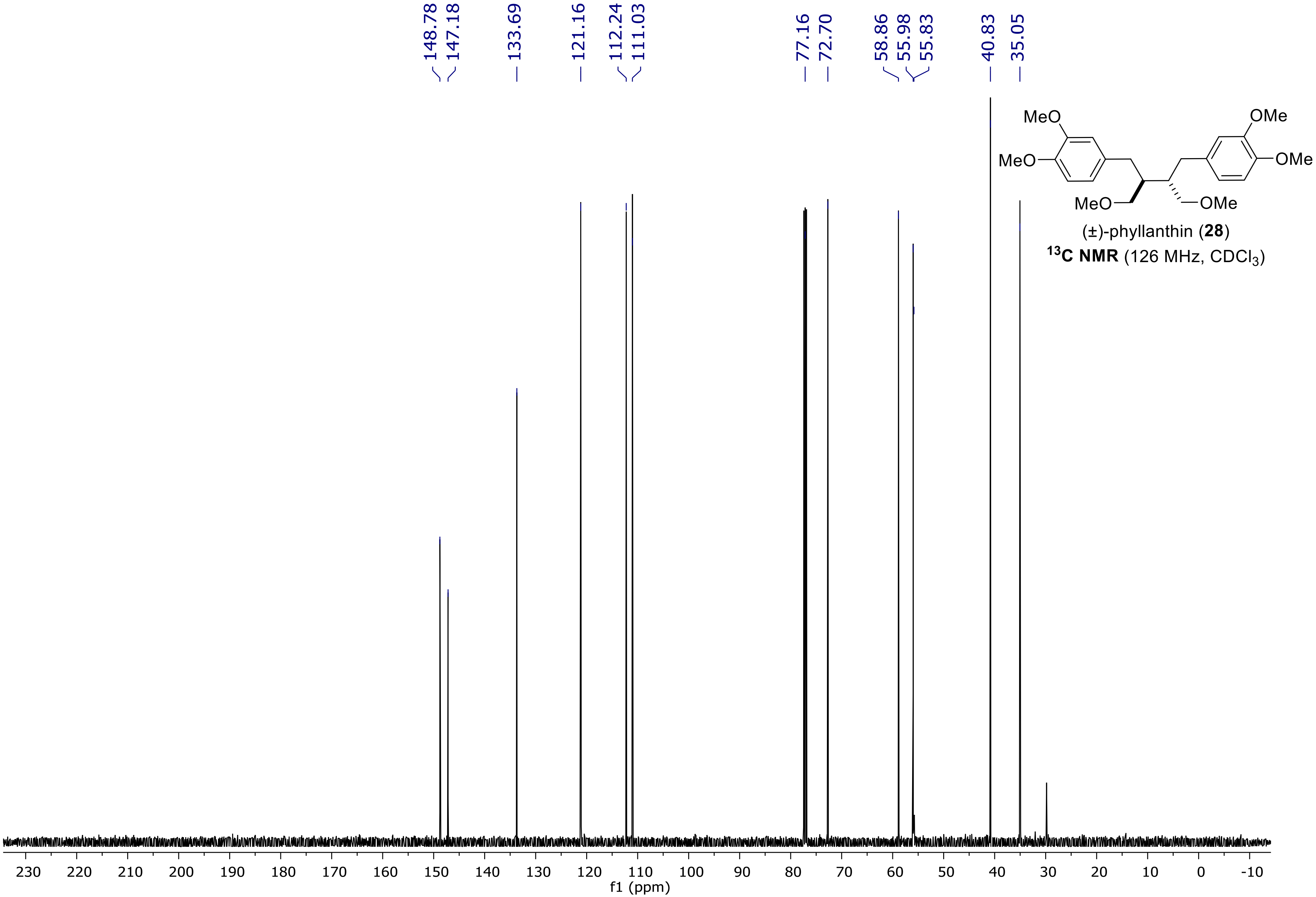

(±)-phyllanthin (**28**)

^{13}C NMR (126 MHz, $CDCl_3$)

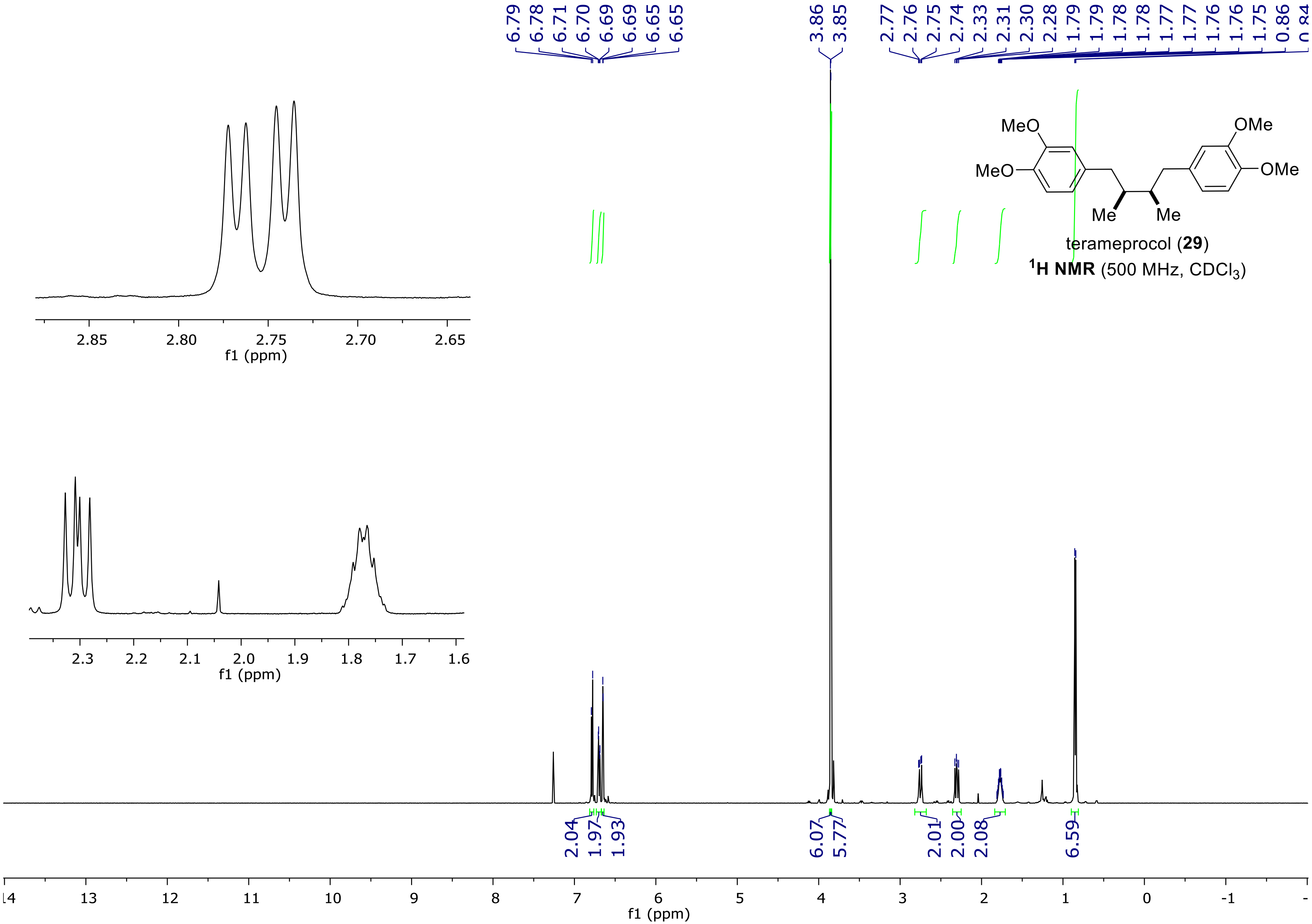
6.79 6.78 6.71 6.70 6.69 6.69 6.65 6.65
3.86 3.85
2.77 2.76 2.75 2.74 2.33 2.31 2.30 2.28 1.79 1.79 1.78 1.78 1.77 1.77 1.76 1.76 1.75 0.86
MeO
MeO
OMe
OMe
Me
Me
terameprocol (**29**)
^{1}**H NMR** (500 MHz, $CDCl_3$)
2.85 2.80 2.75 2.70 2.65
f1 (ppm)
2.3 2.2 2.1 2.0 1.9 1.8 1.7 1.6
f1 (ppm)
2.04 1.97 1.93
6.07 5.77
2.01 2.00 2.08
6.59
13 12 11 10 9 8 7 6 5 4 3 2 1 0 -1
f1 (ppm)

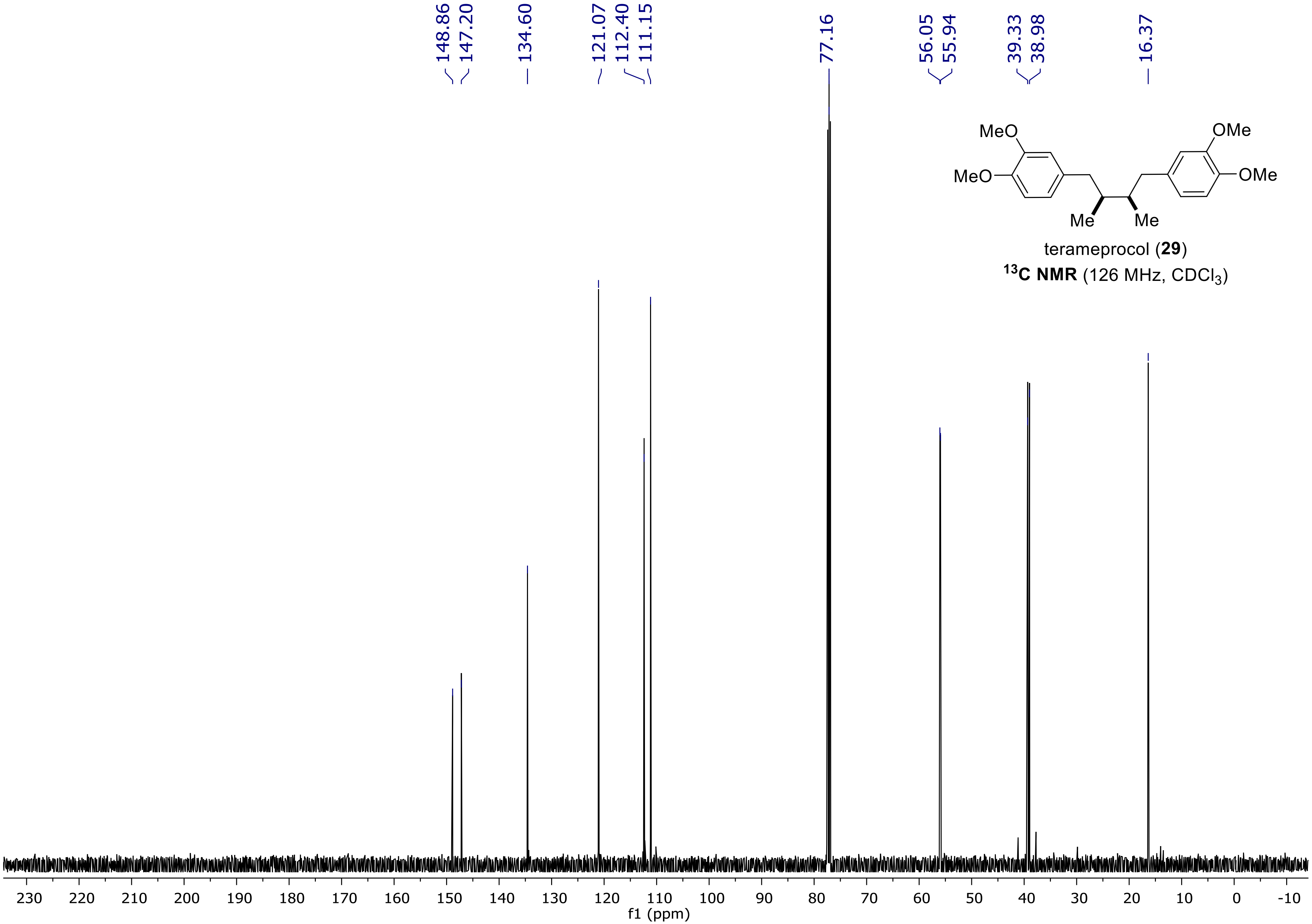

terameprocol (**29**)

^{13}C NMR (126 MHz, $CDCl_3$)

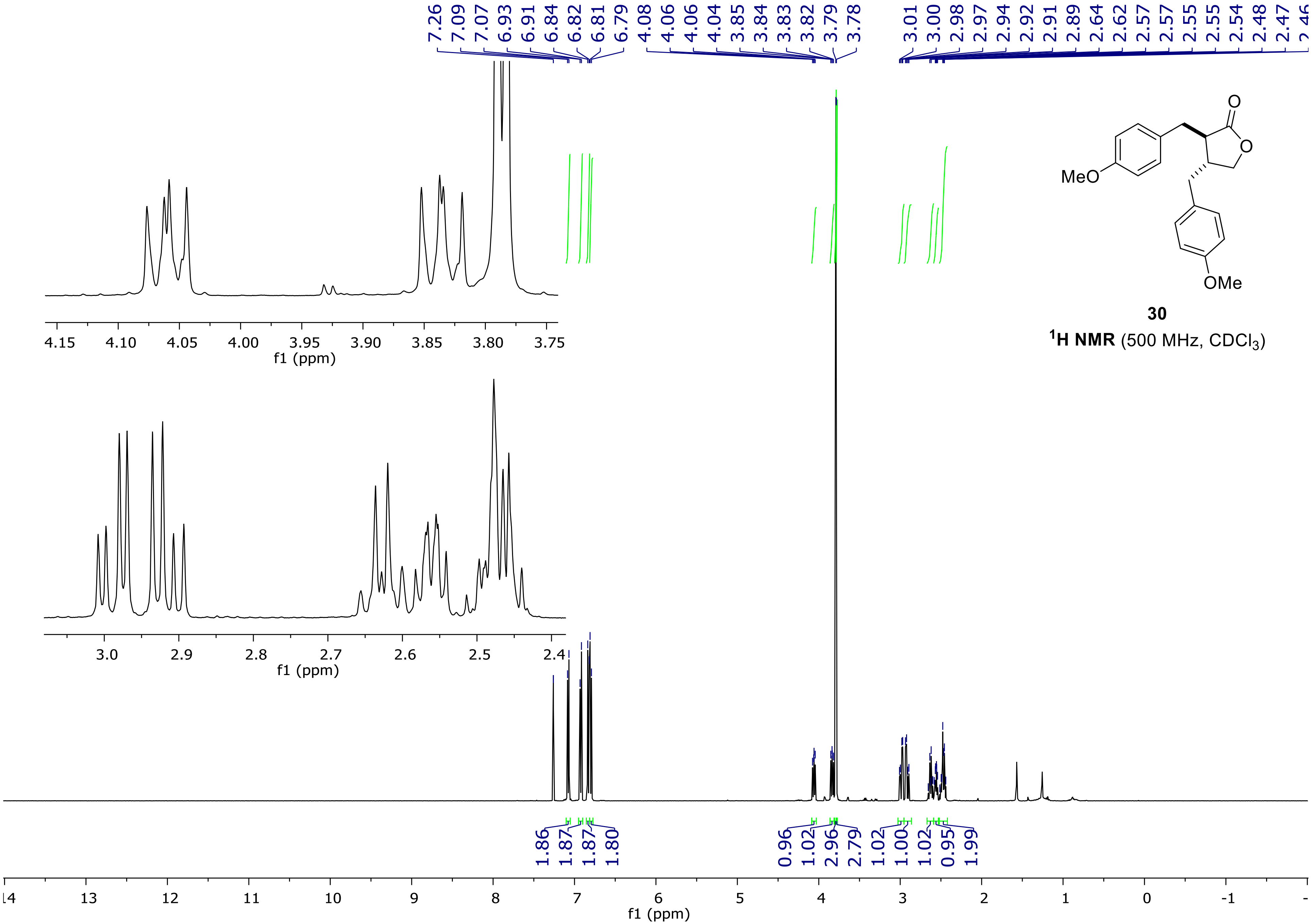

30

^{1}**H NMR** (500 MHz, $CDCl_3$)

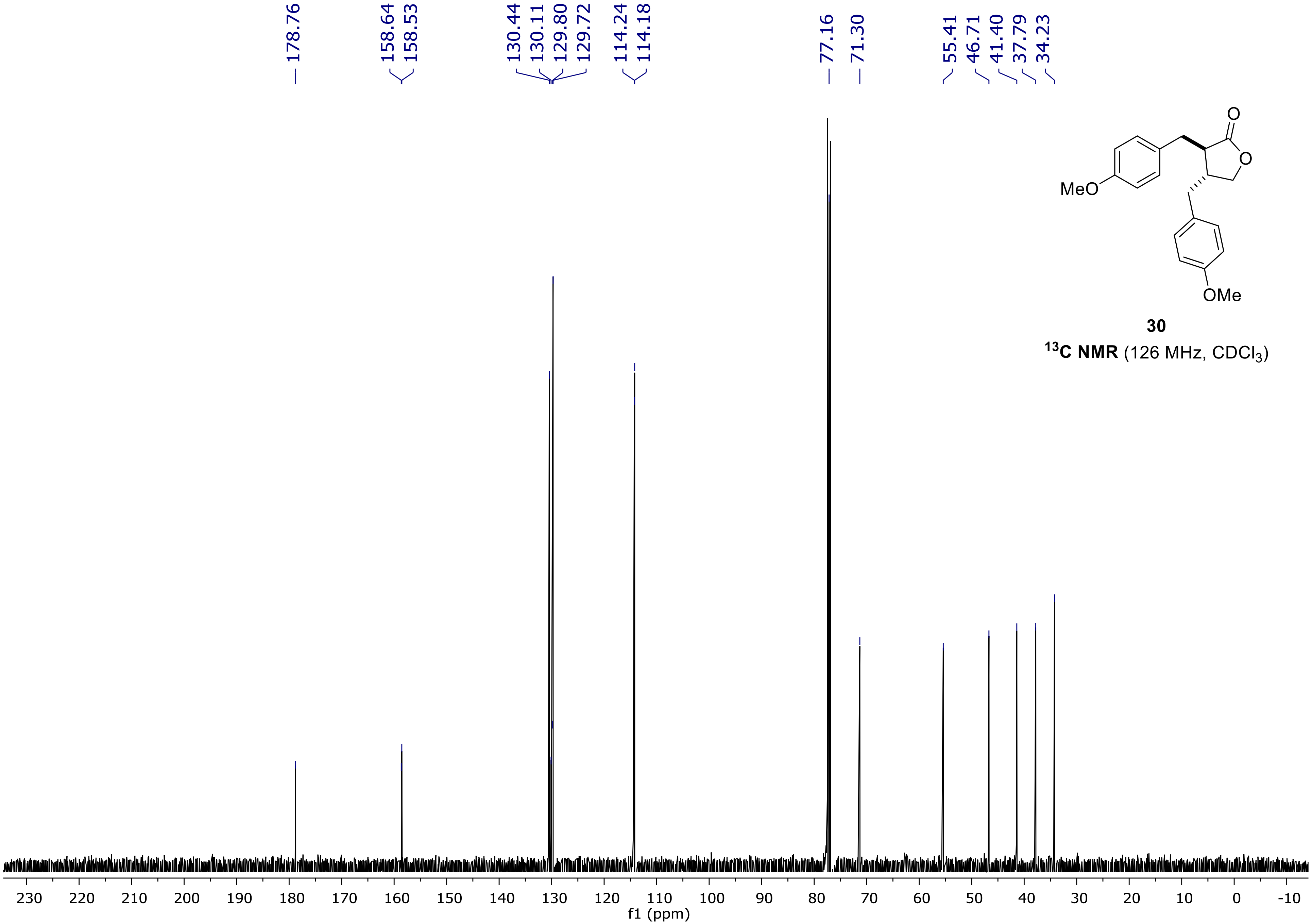

30

^{13}C NMR (126 MHz, $CDCl_3$)

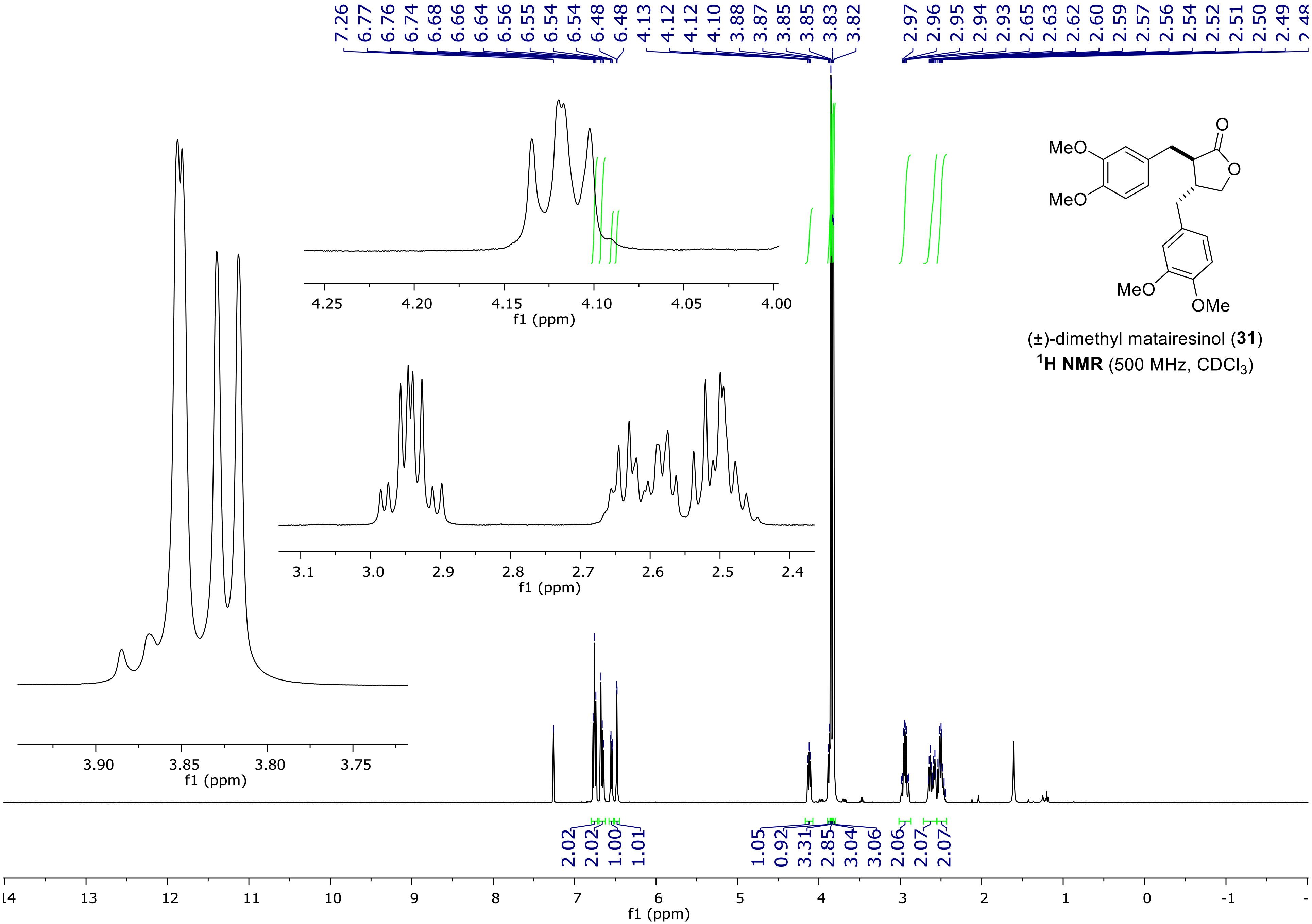

(±)-dimethyl matairesinol (**31**)
^{1}H NMR (500 MHz, $CDCl_3$)

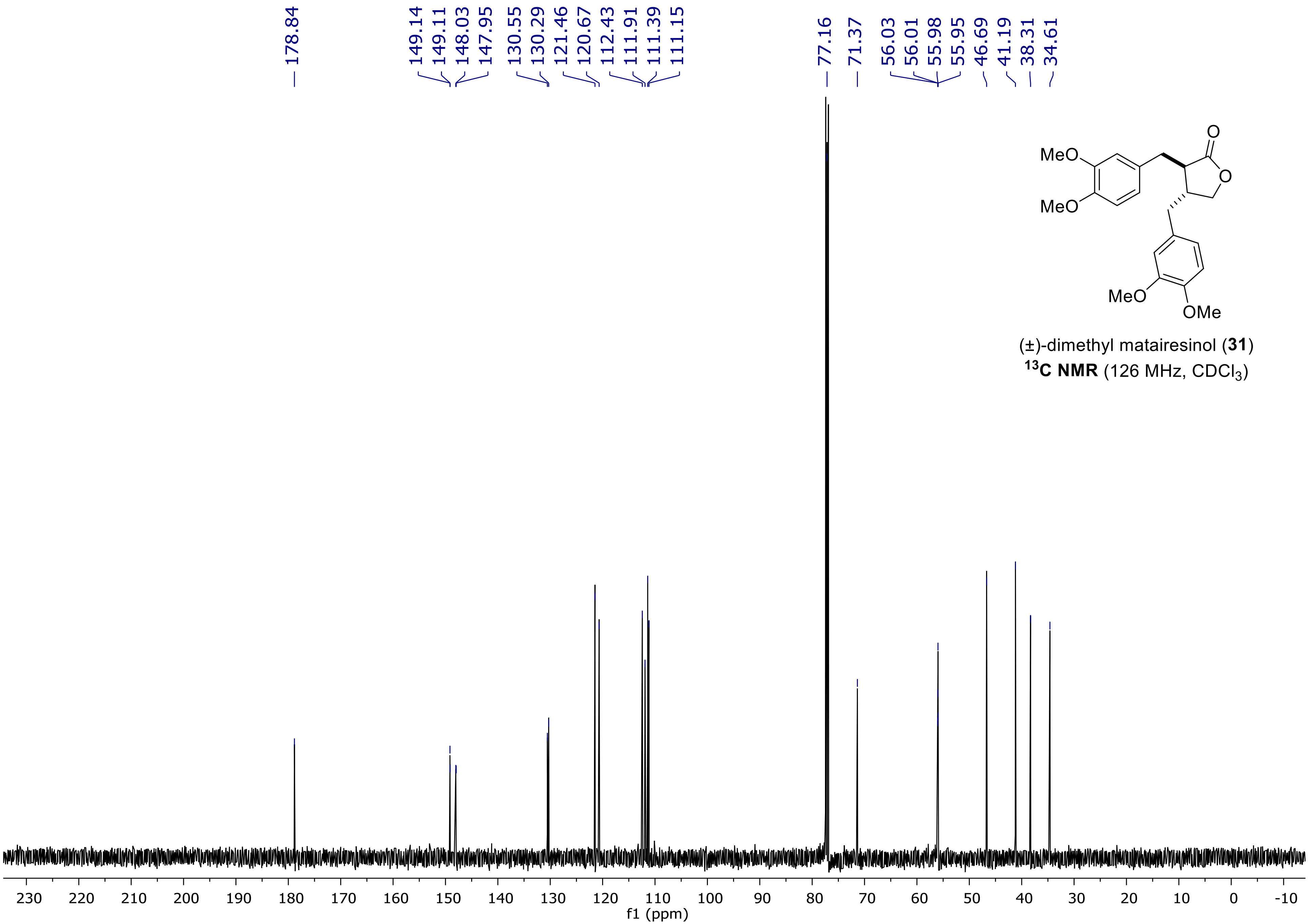

(±)-dimethyl matairesinol (**31**)

^{13}C NMR (126 MHz, $CDCl_3$)

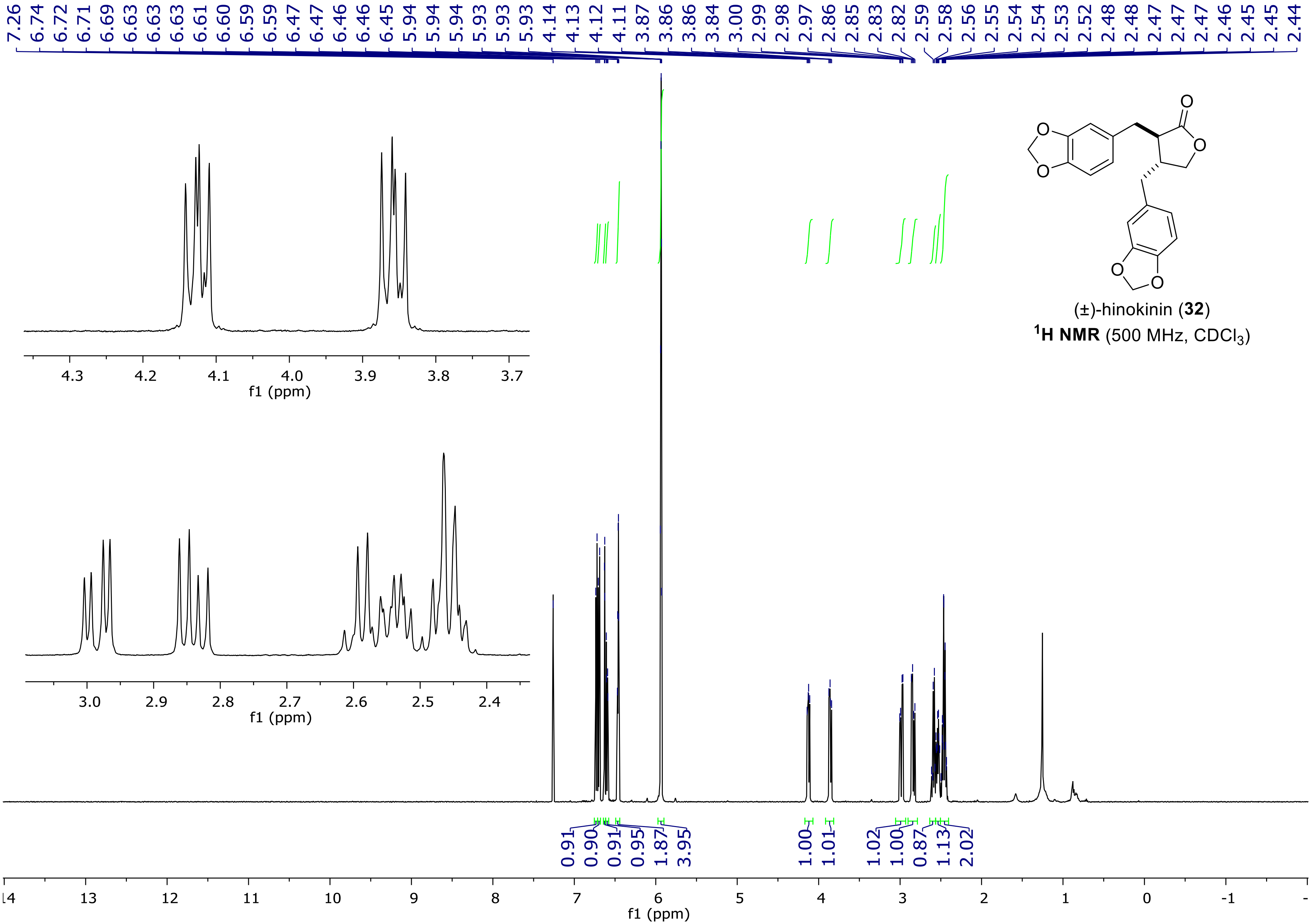

(±)-hinokinin (**32**)

^{1}H NMR (500 MHz, $CDCl_3$)

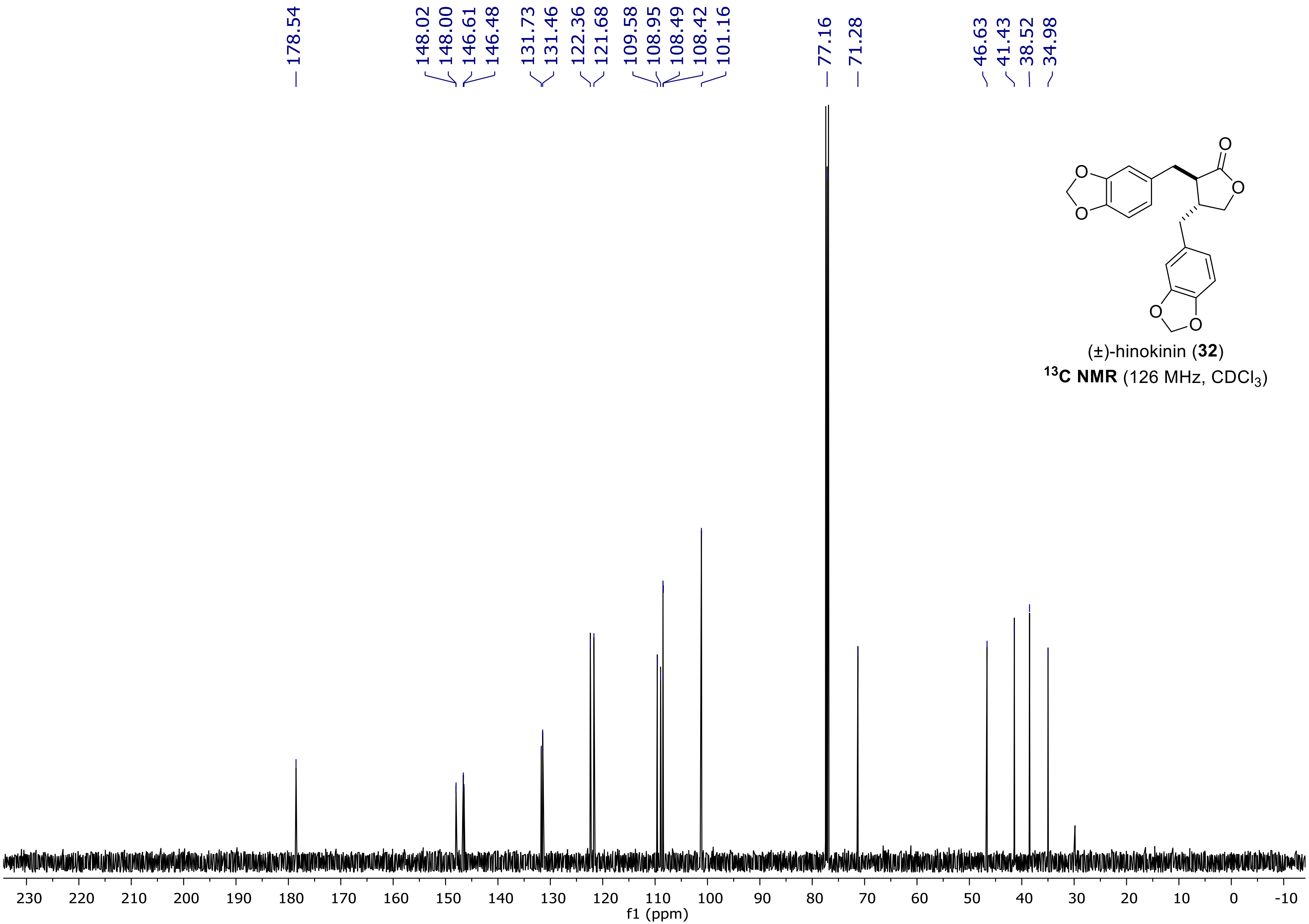

(±)-hinokinin (**32**)

^{13}C NMR (126 MHz, $CDCl_3$)

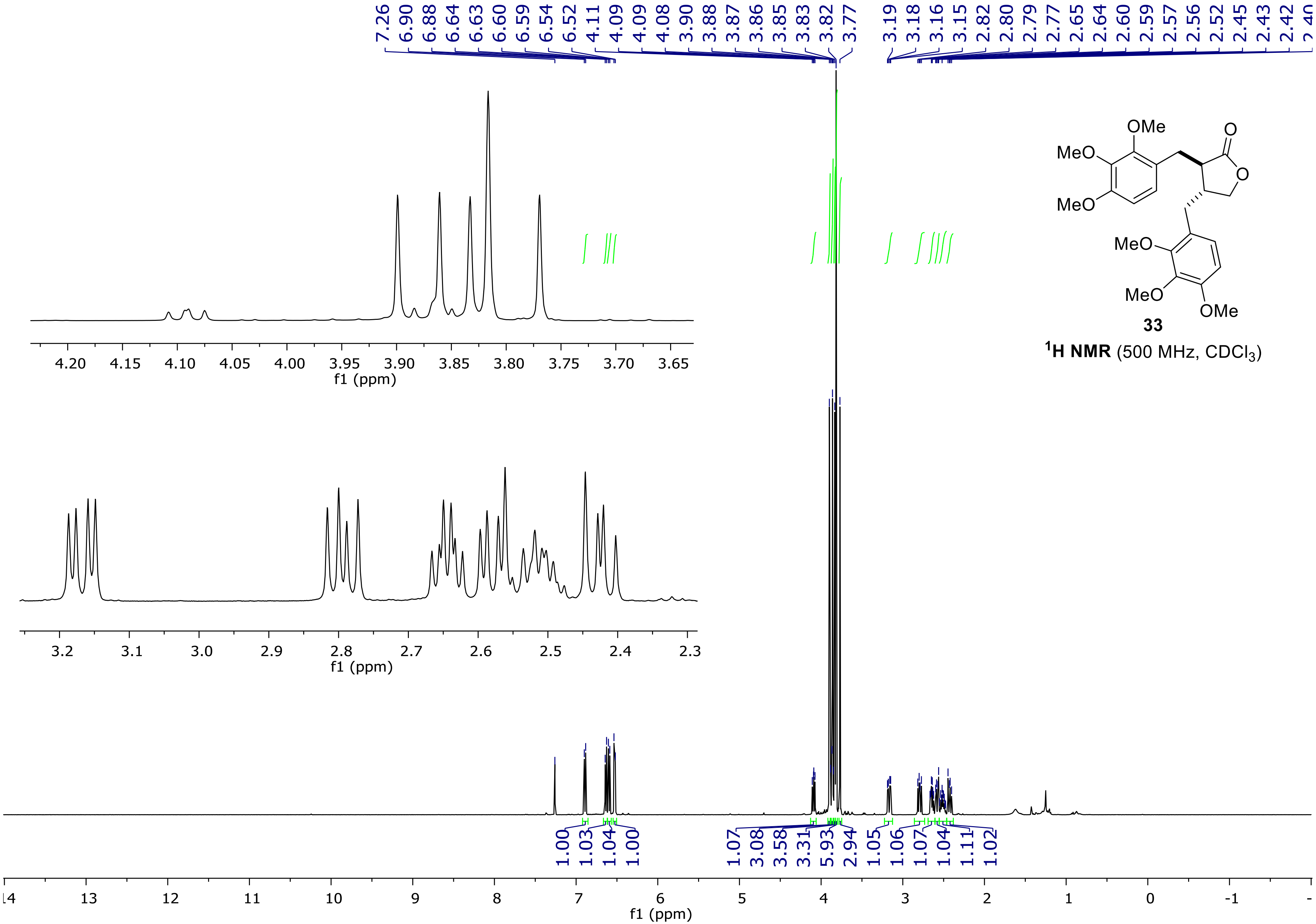

33

^{1}H NMR (500 MHz, $CDCl_3$)

Alfonzo 104

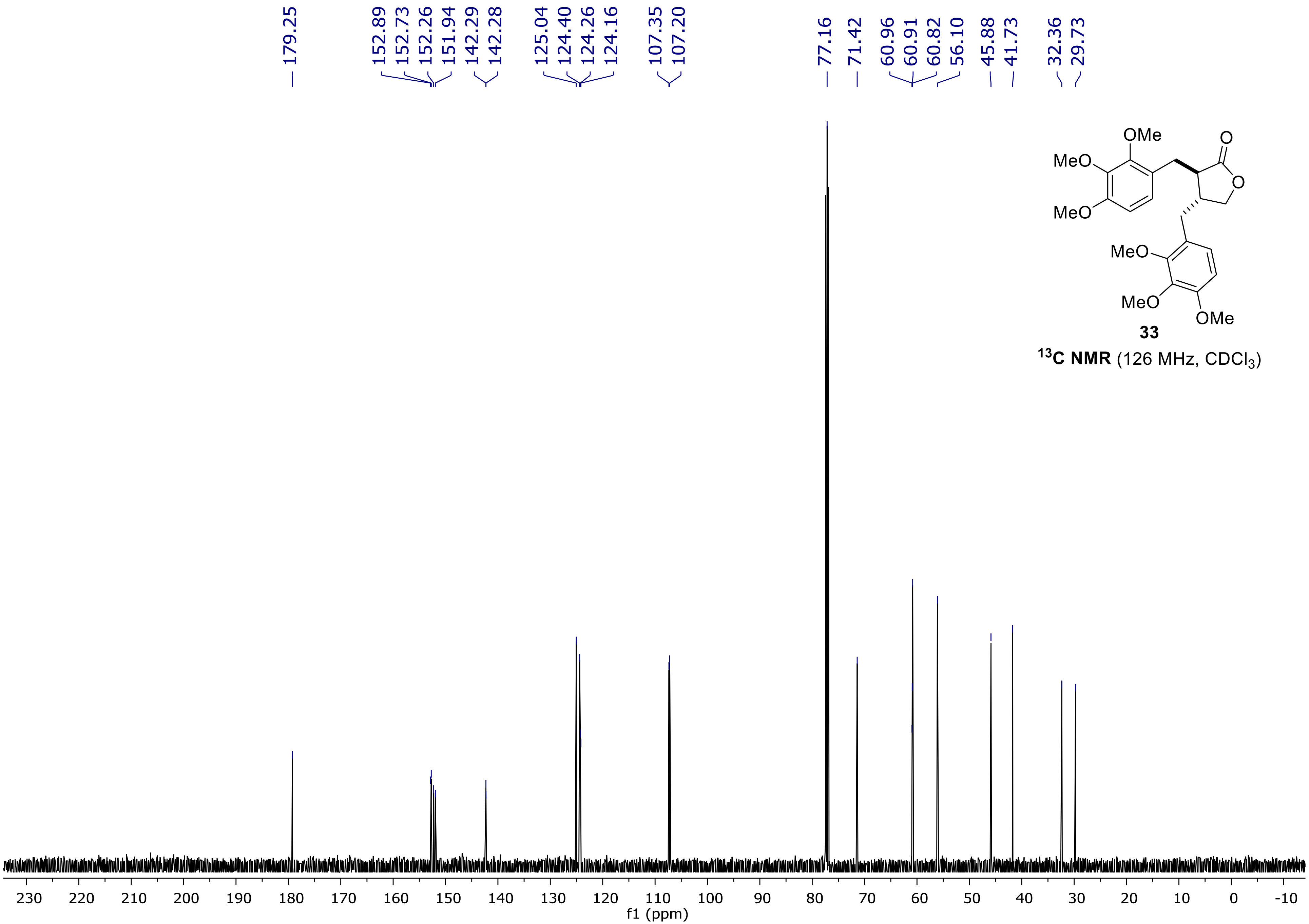
179.25
152.89
152.73
152.26
151.94
142.29
142.28
125.04
124.40
124.26
124.16
107.35
107.20
77.16
71.42
60.96
60.91
60.82
56.10
45.88
41.73
32.36
29.73
OMe
MeO
MeO
O
O
MeO
MeO
OMe
33
^{13}C NMR (126 MHz, CDCl$_3$)
230 220 210 200 190 180 170 160 150 140 130 120 110 100 90 80 70 60 50 40 30 20 10 0 -10
f1 (ppm)

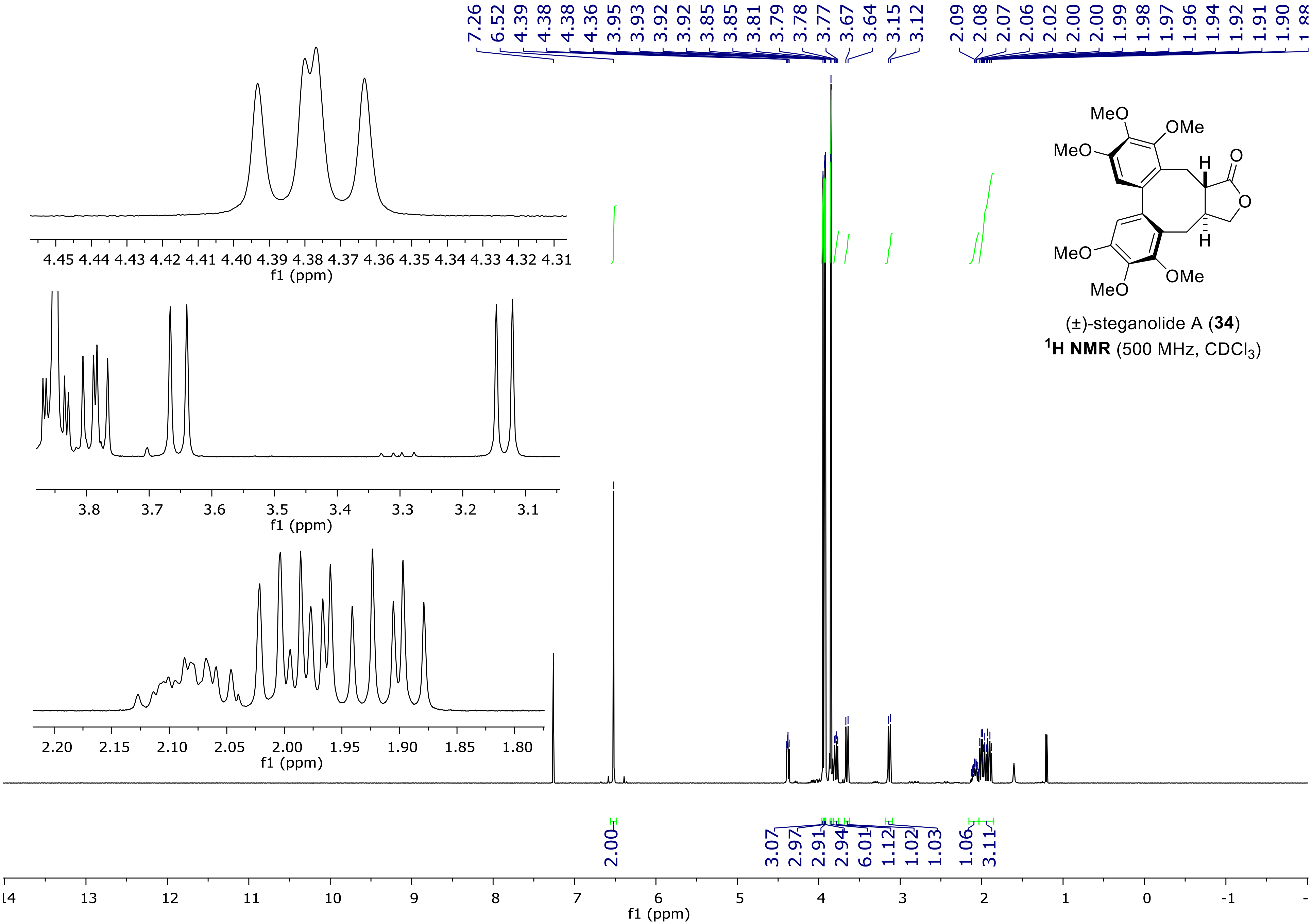

(±)-steganolide A (**34**)
^{1}H NMR (500 MHz, $CDCl_3$)

Alfonzo 106

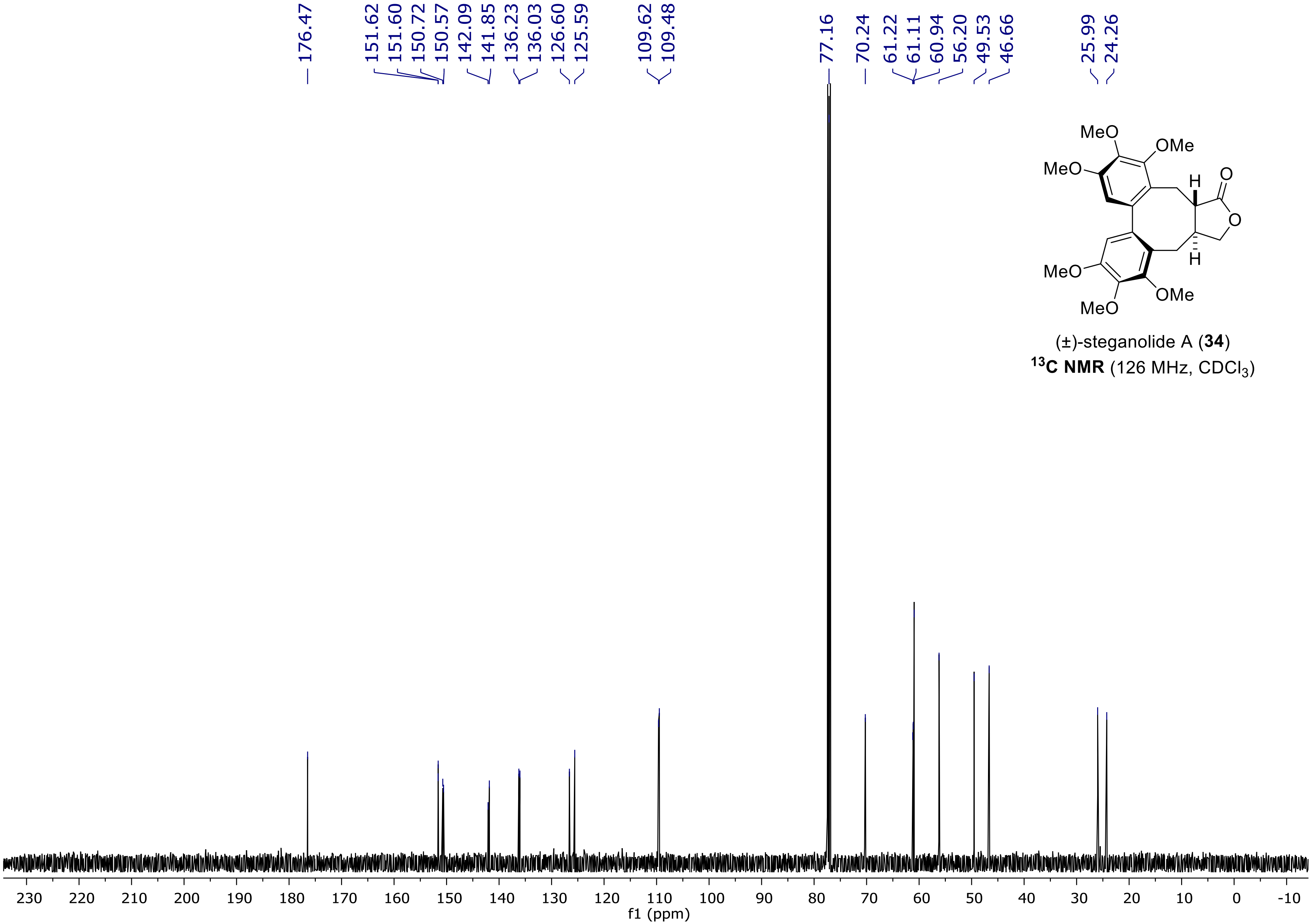

(±)-steganolide A (**34**)

^{13}C NMR (126 MHz, $CDCl_3$)

Alfonzo 107

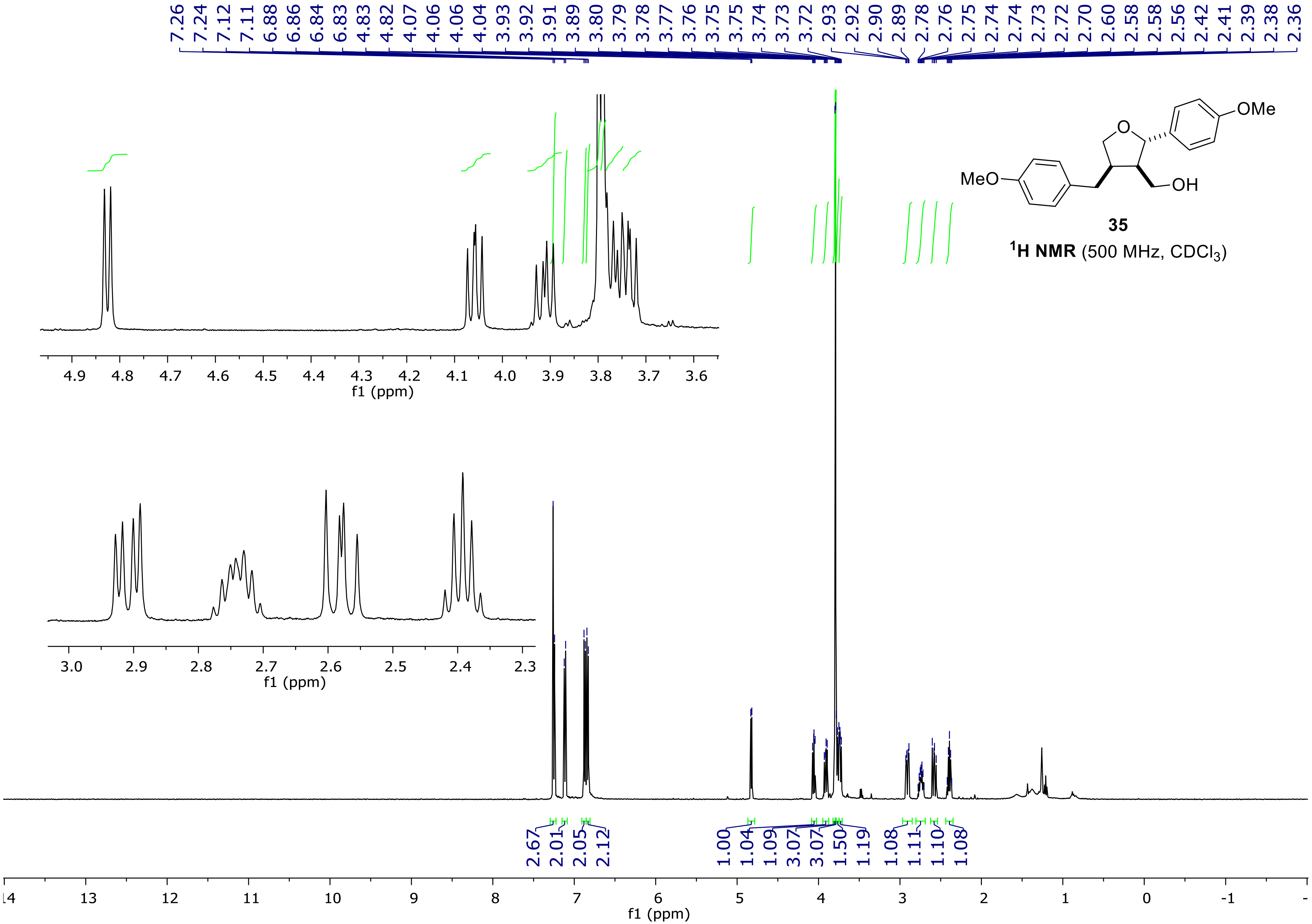

35

^{1}**H NMR** (500 MHz, $CDCl_3$)

Alfonzo 108

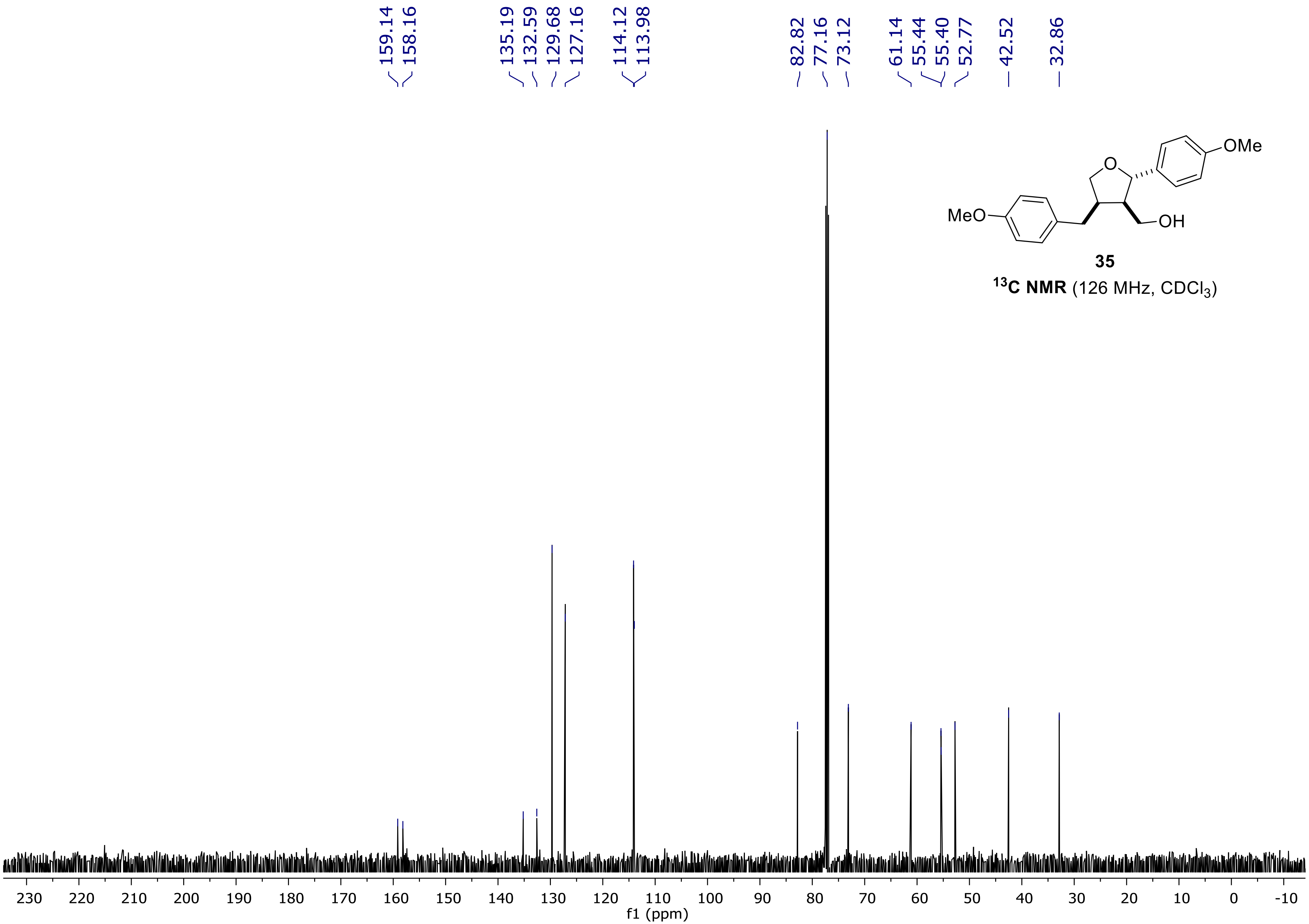

35

^{13}C NMR (126 MHz, $CDCl_3$)

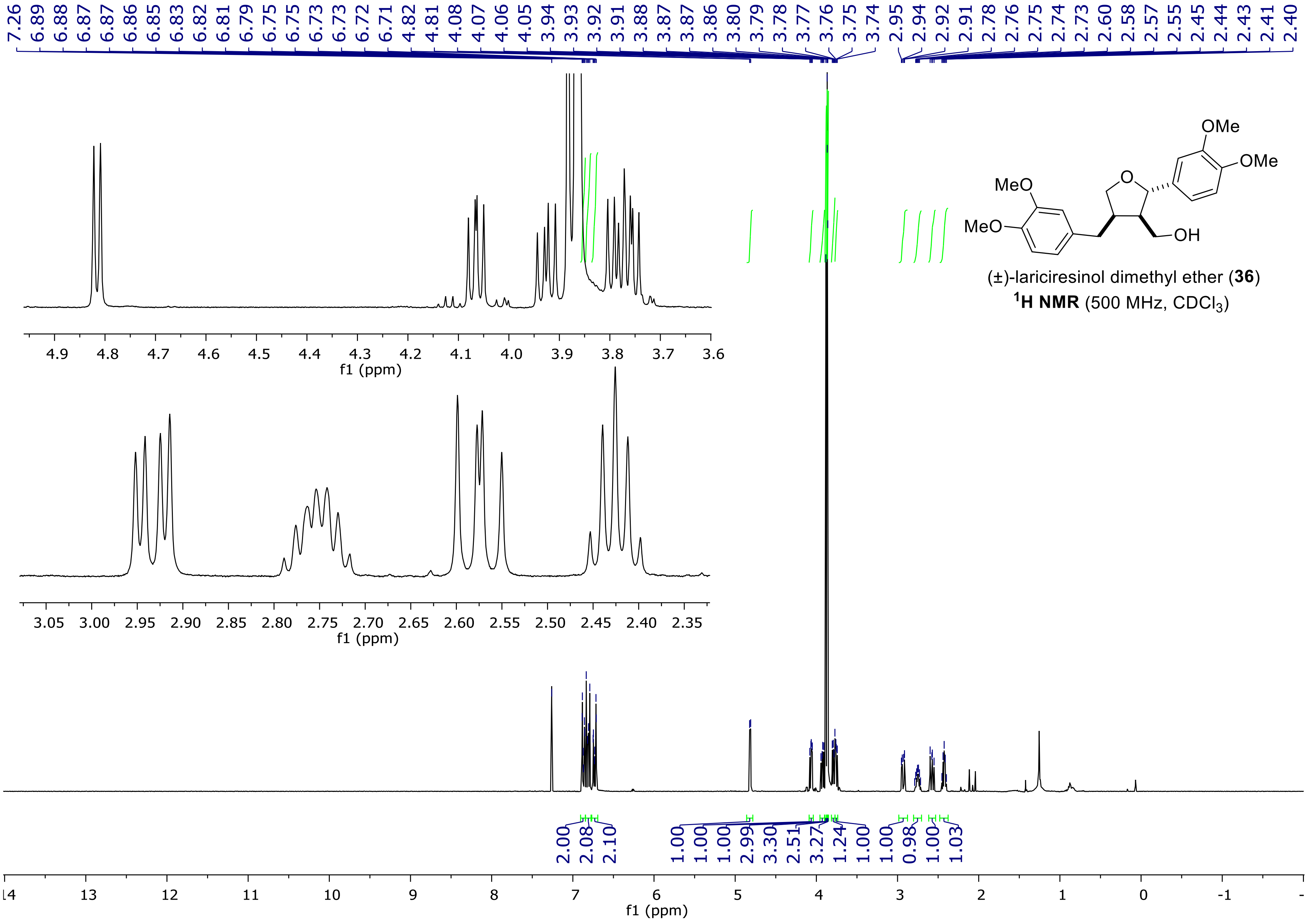

(±)-lariciresinol dimethyl ether (**36**)

^{1}H NMR (500 MHz, $CDCl_3$)

Alfonzo 110

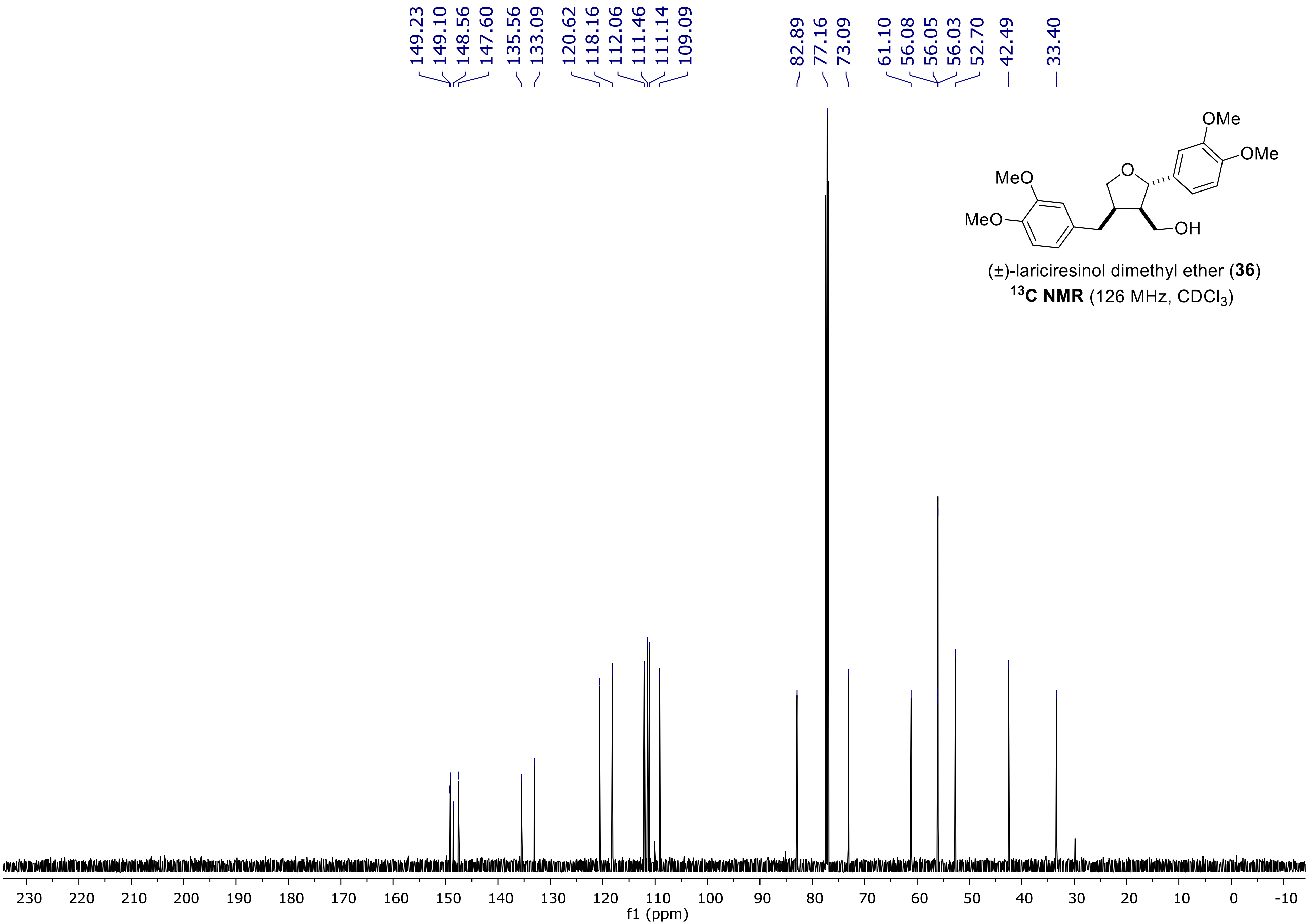

(±)-lariciresinol dimethyl ether (**36**)

^{13}C NMR (126 MHz, $CDCl_3$)

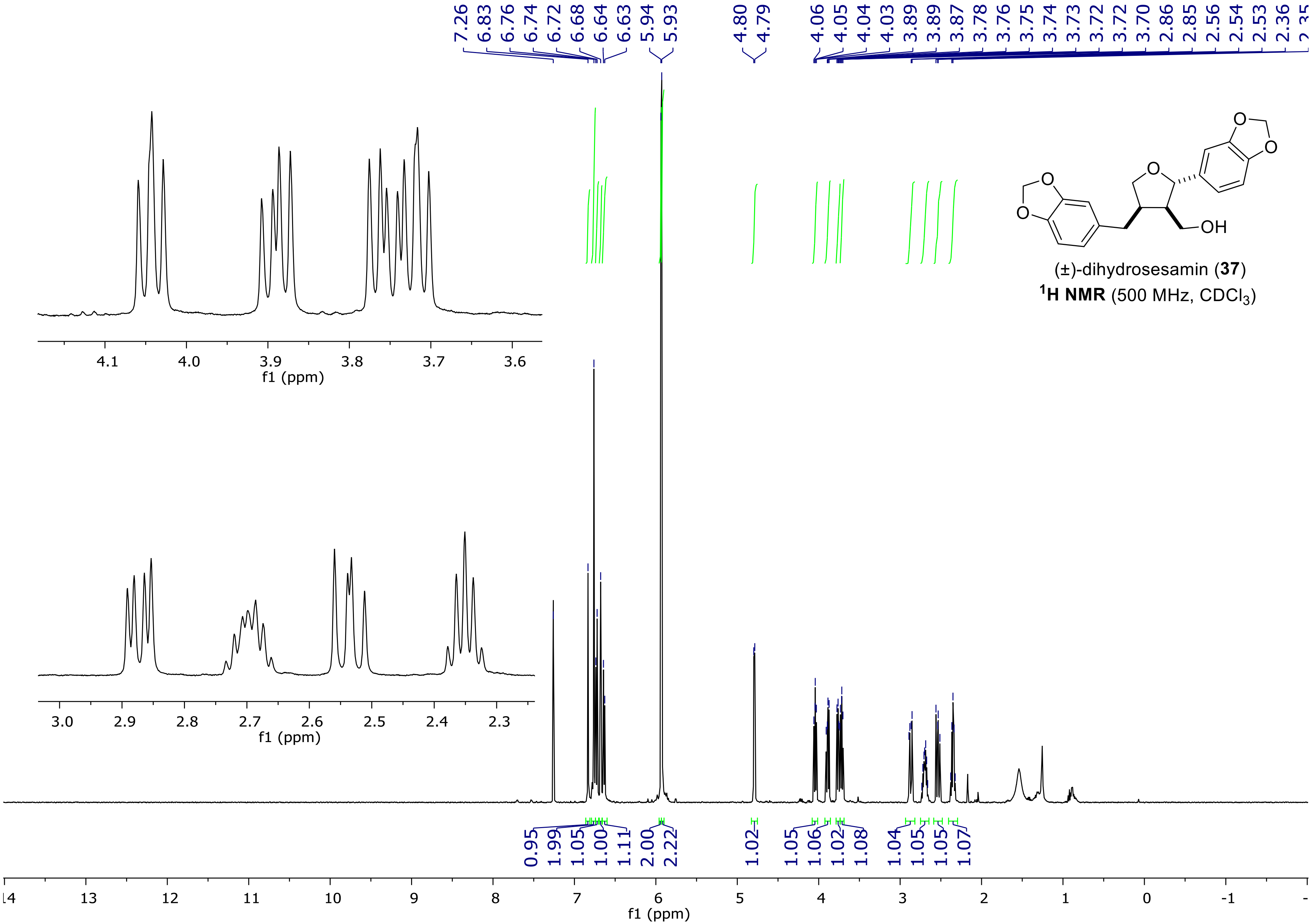

(±)-dihydrosesamin (**37**)

^{1}H NMR (500 MHz, $CDCl_3$)

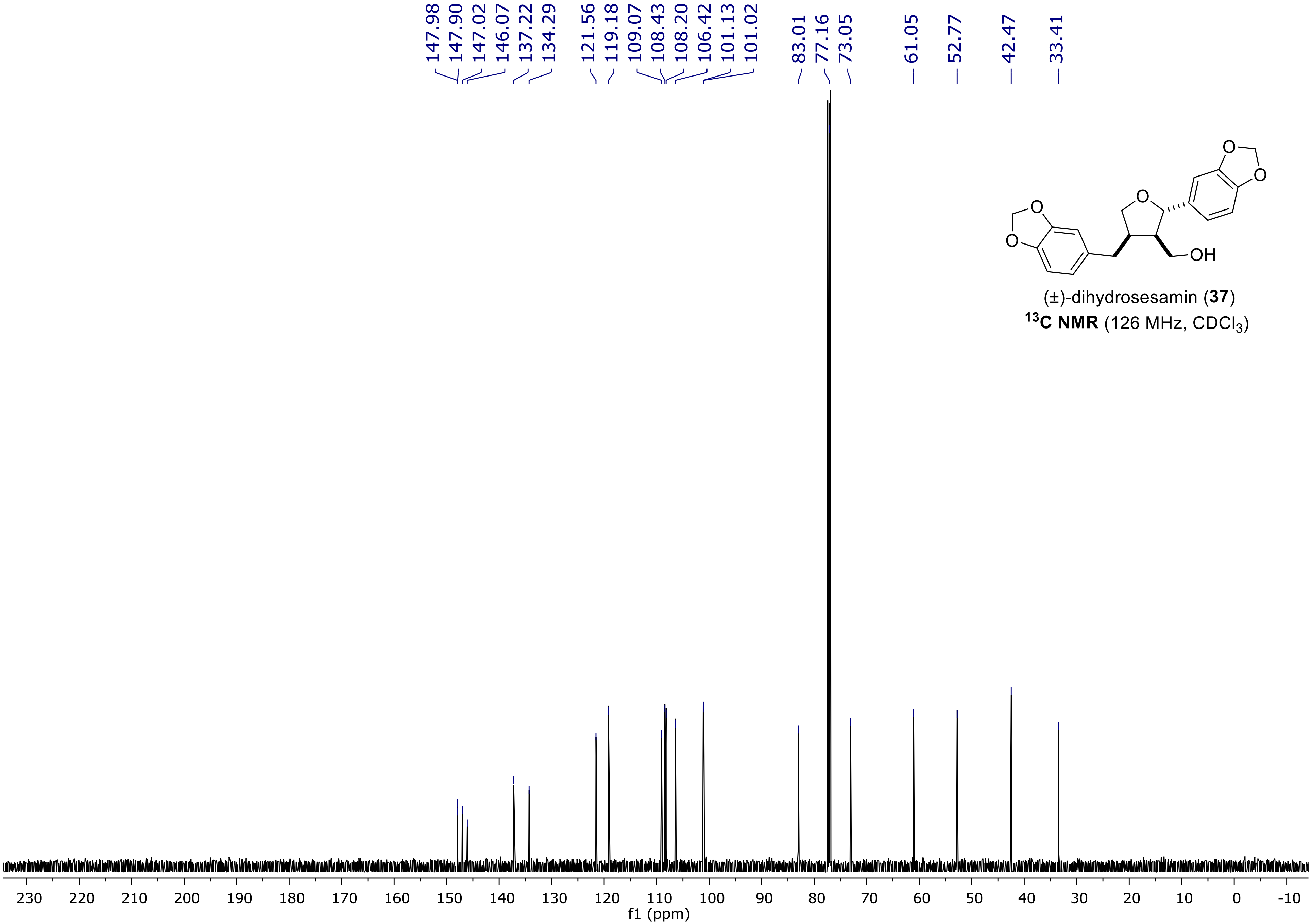

(±)-dihydrosesamin (**37**)

^{13}C NMR (126 MHz, $CDCl_3$)

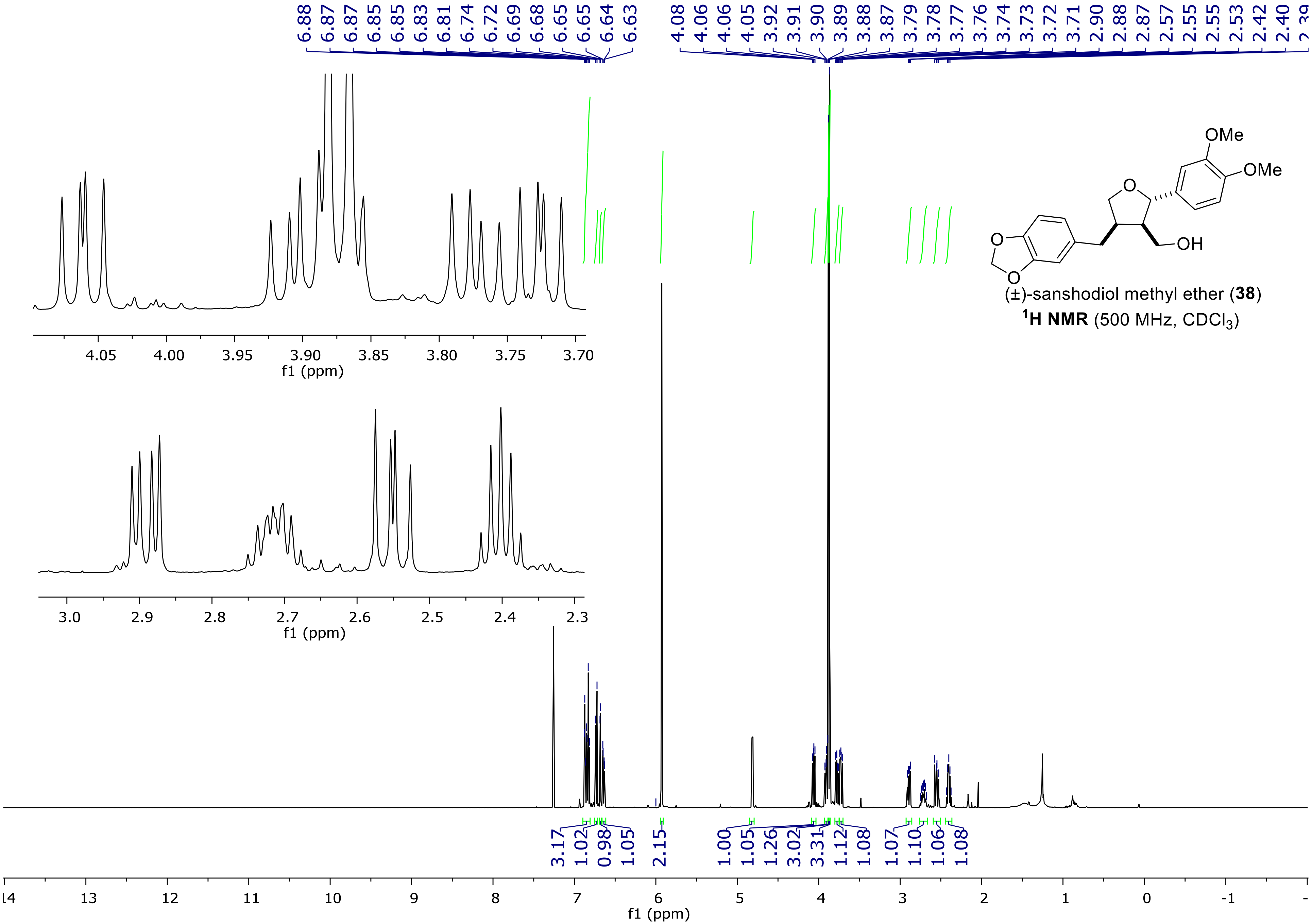

(±)-sanshodiol methyl ether (**38**)

^{1}H NMR (500 MHz, $CDCl_3$)

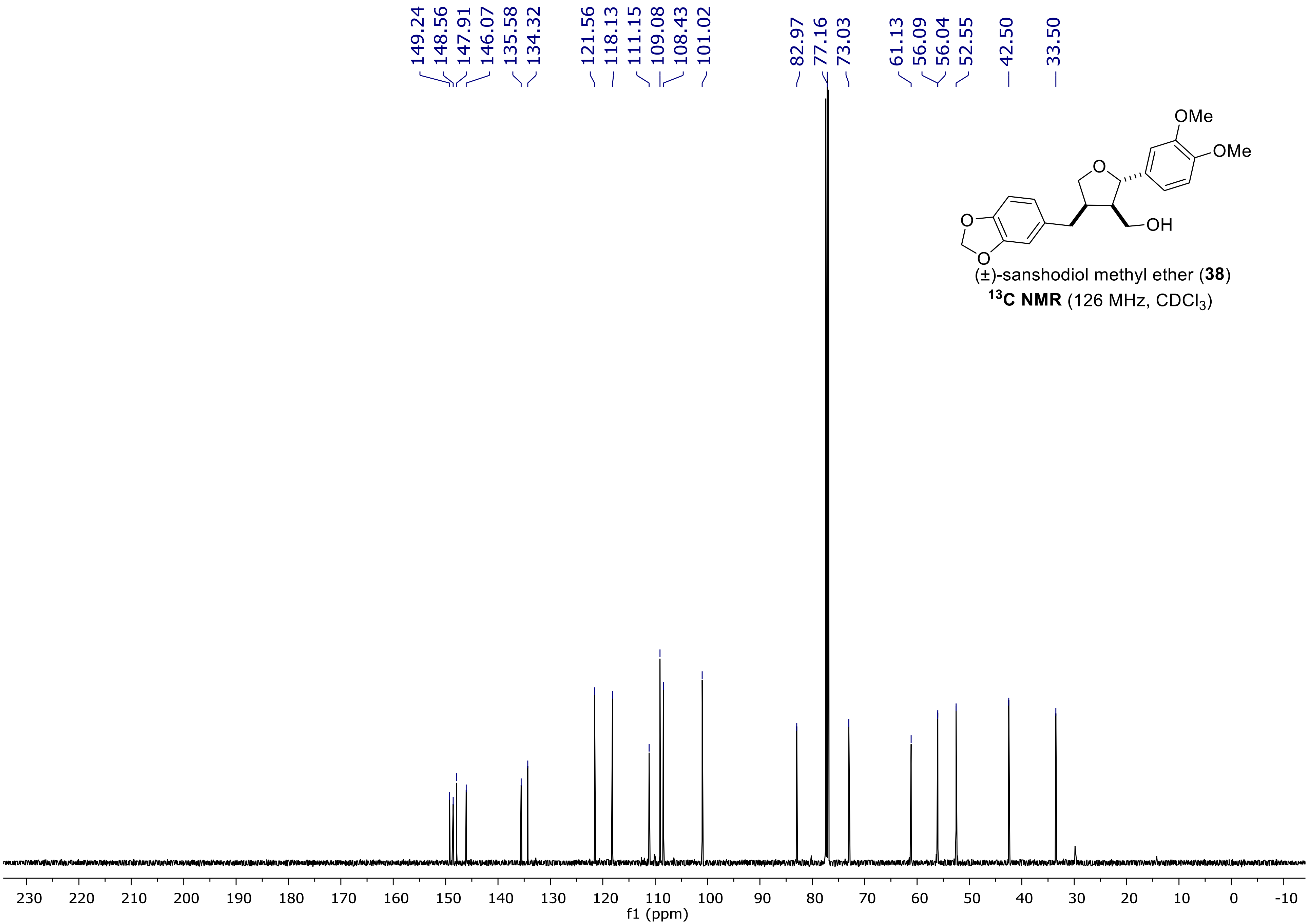

(±)-sanshodiol methyl ether (**38**)
^{13}C NMR (126 MHz, $CDCl_3$)

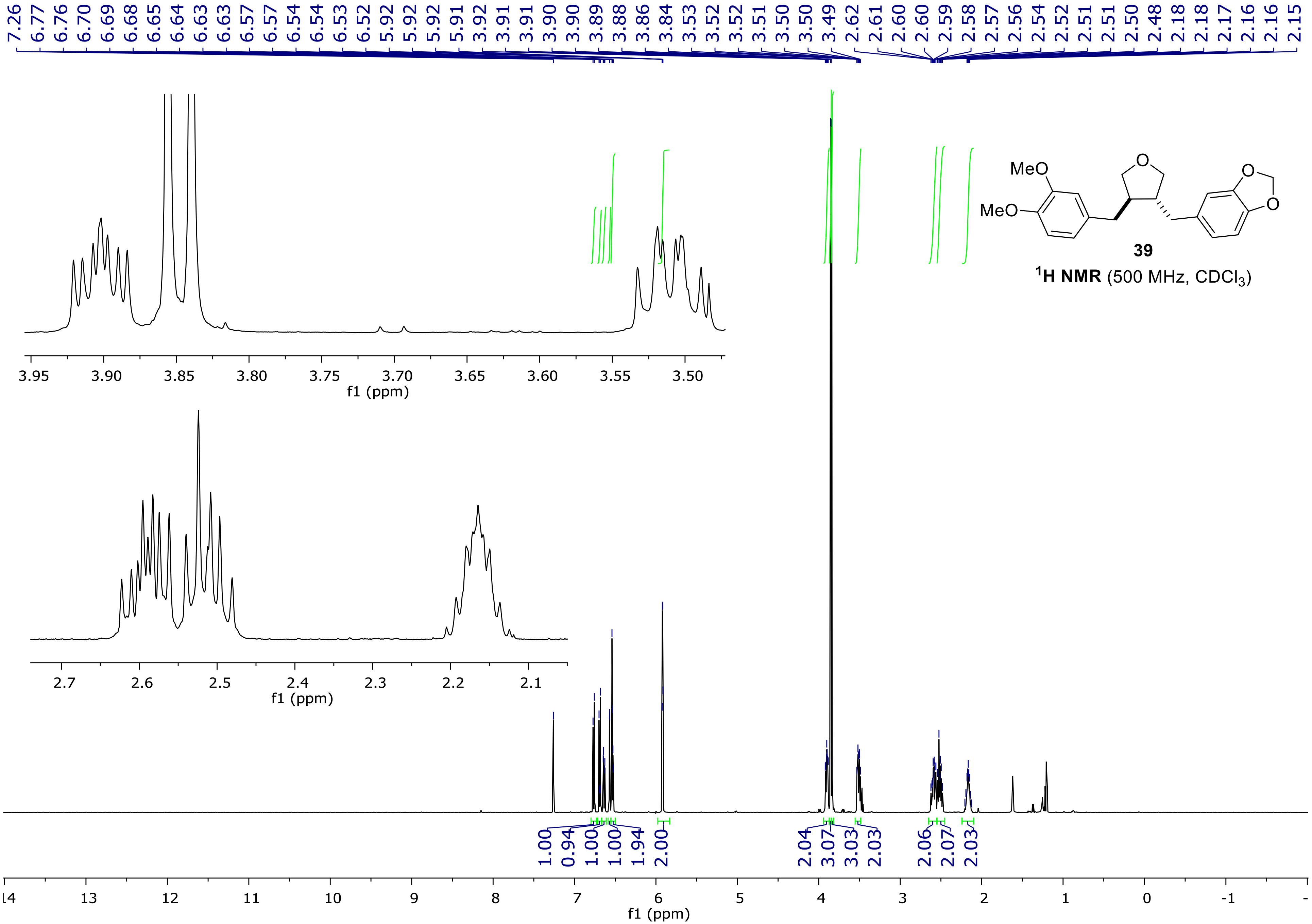

39

^{1}**H NMR** (500 MHz, $CDCl_3$)

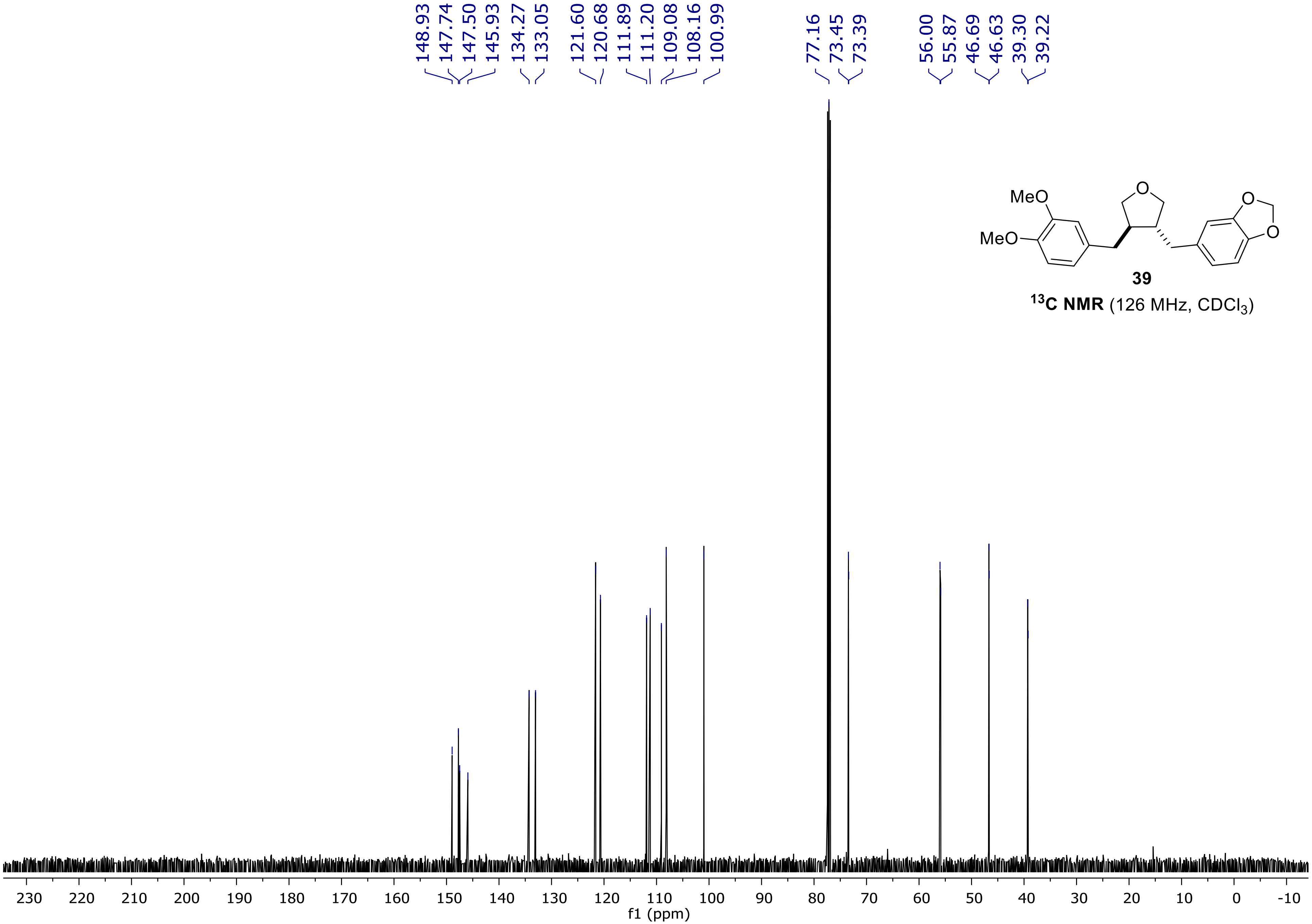

39

^{13}C NMR (126 MHz, $CDCl_3$)

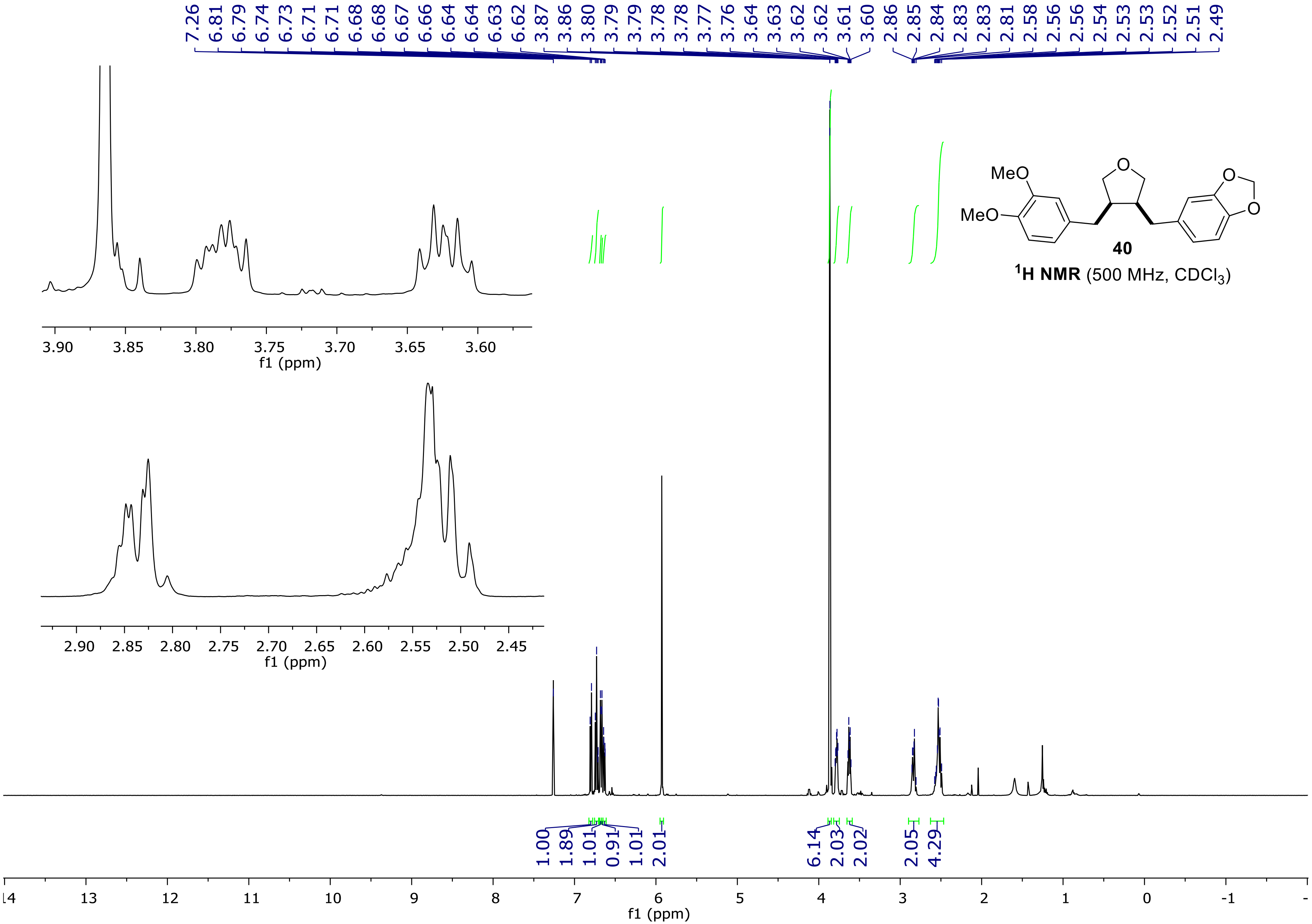

40

^{1}H NMR (500 MHz, $CDCl_3$)

Alfonzo 118

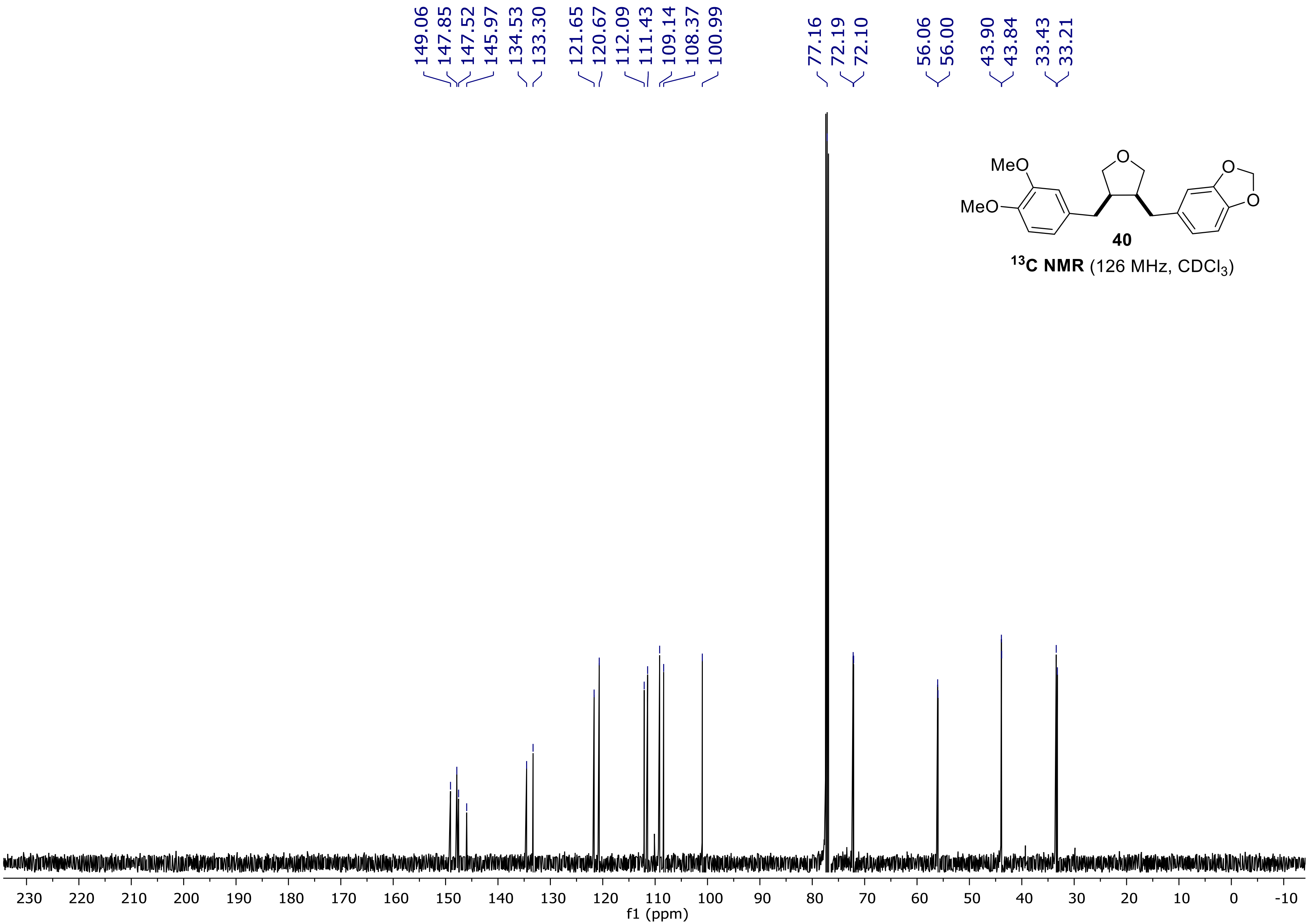

149.06
147.85
147.52
145.97
134.53
133.30
121.65
120.67
112.09
111.43
109.14
108.37
100.99
77.16
72.19
72.10
56.06
56.00
43.90
43.84
33.43
33.21
MeO
MeO
O
O
O
40
^{13}C NMR (126 MHz, CDCl$_3$)
230
220
210
200
190
180
170
160
150
140
130
120
110
100
90
80
70
60
50
40
30
20
10
0
-10
f1 (ppm)

Alfonzo 119

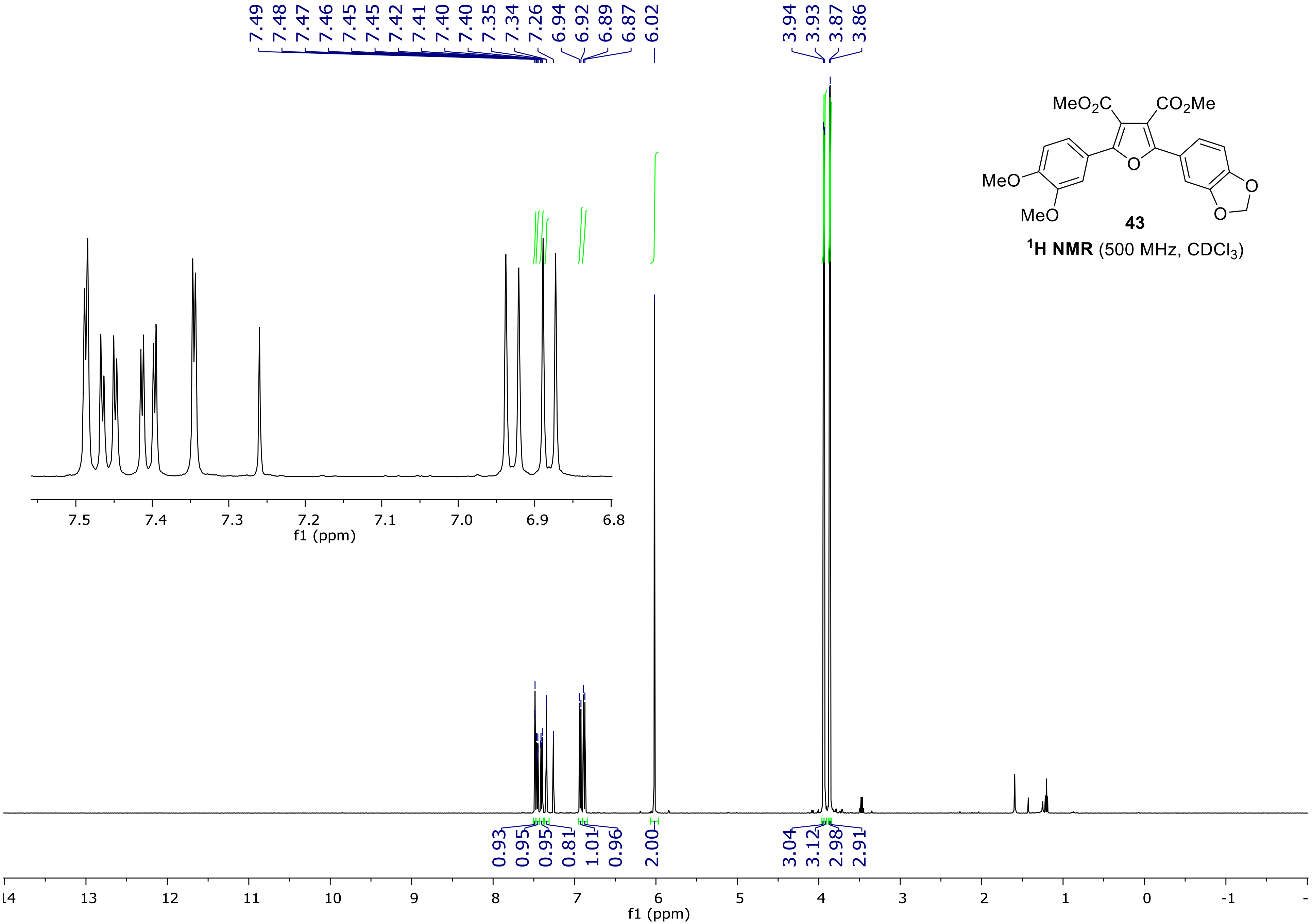

7.49 7.48 7.47 7.46 7.45 7.45 7.42 7.41 7.40 7.40 7.35 7.34 7.26 6.94 6.92 6.89 6.87 6.02
3.94 3.93 3.87 3.86
MeO2C
CO2Me
MeO
MeO
O
43
1H NMR (500 MHz, CDCl3)
7.5 7.4 7.3 7.2 7.1 7.0 6.9 6.8
f1 (ppm)
0.93 0.95 0.95 0.81 1.01 0.96 2.00
3.04 3.12 2.98 2.91
13 12 11 10 9 8 7 6 5 4 3 2 1 0 -1
f1 (ppm)

Alfonzo 120

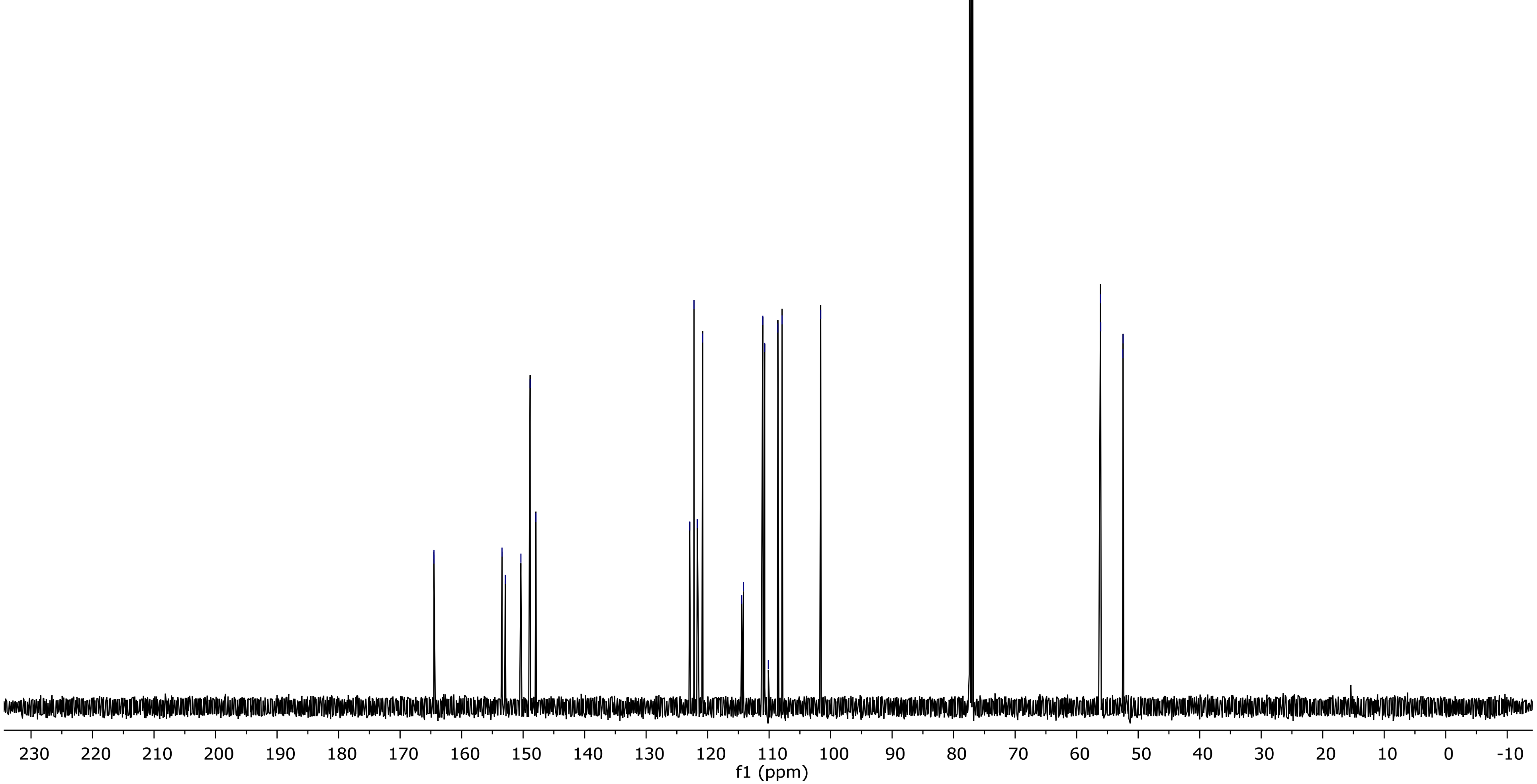

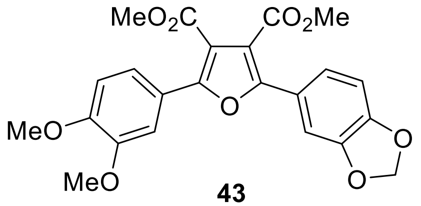

43

^{13}C NMR (126 MHz, $CDCl_3$)

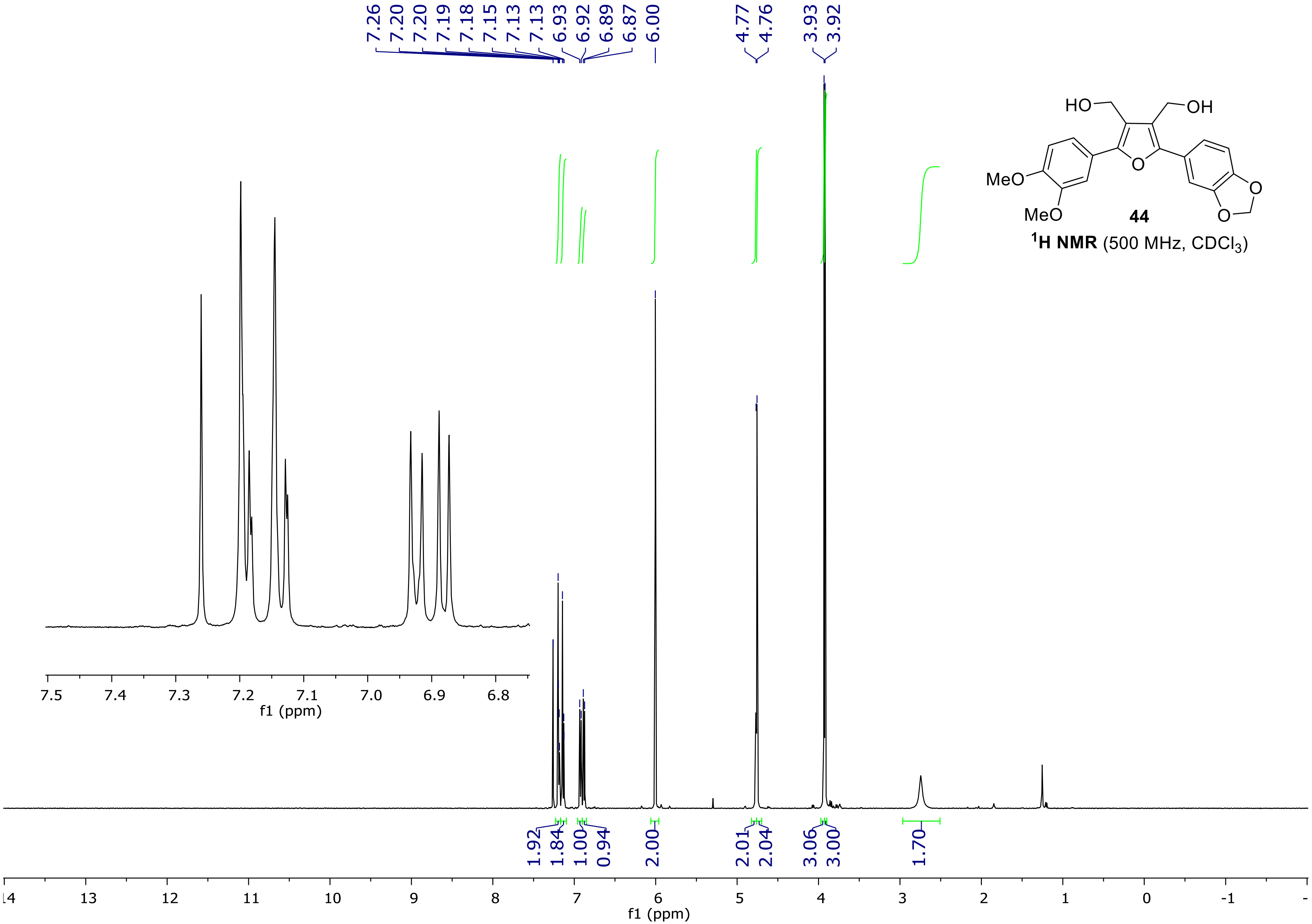

44

^{1}H NMR (500 MHz, $CDCl_3$)

Alfonzo 122

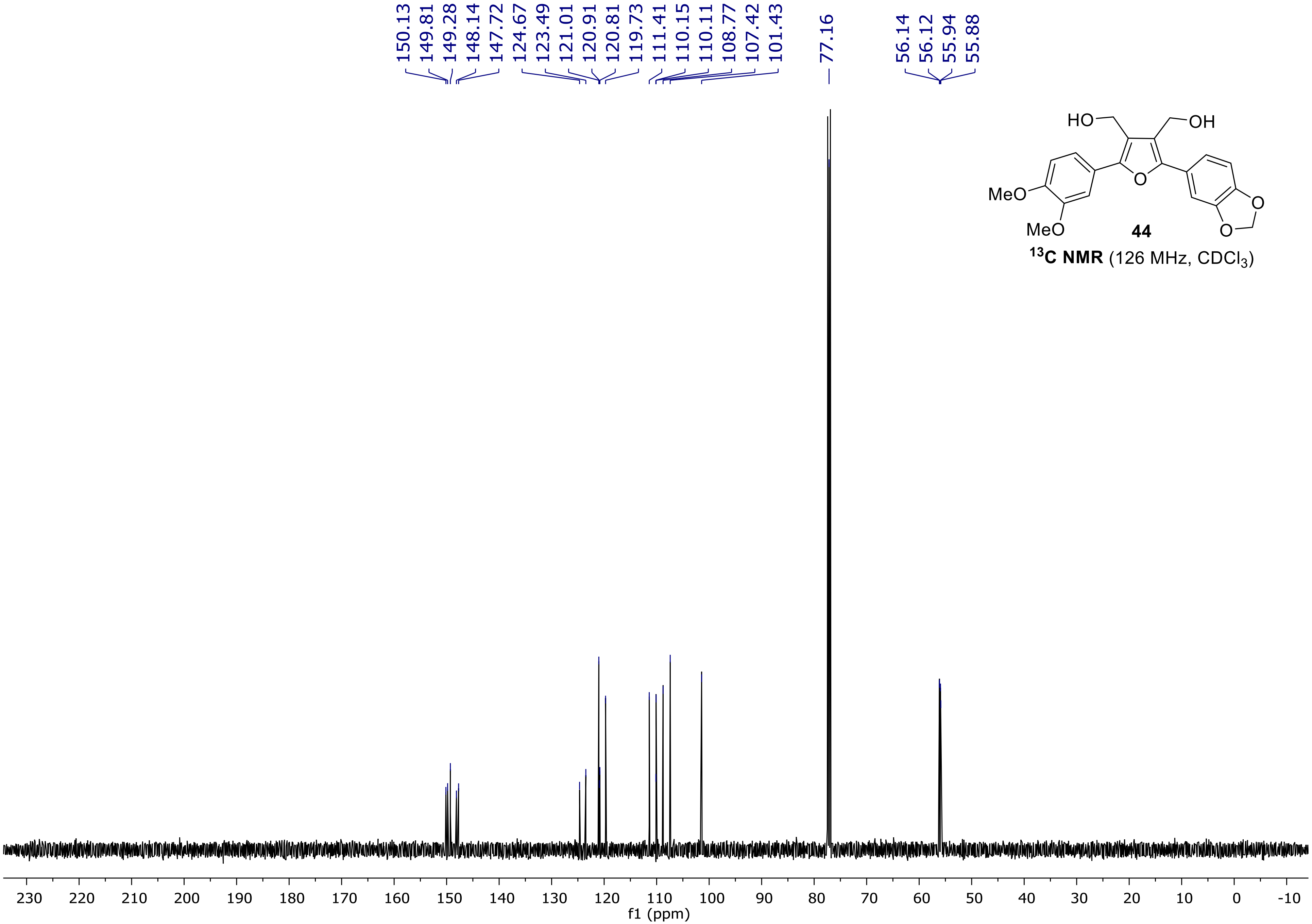

44

^{13}C NMR (126 MHz, $CDCl_3$)

Alfonzo 123

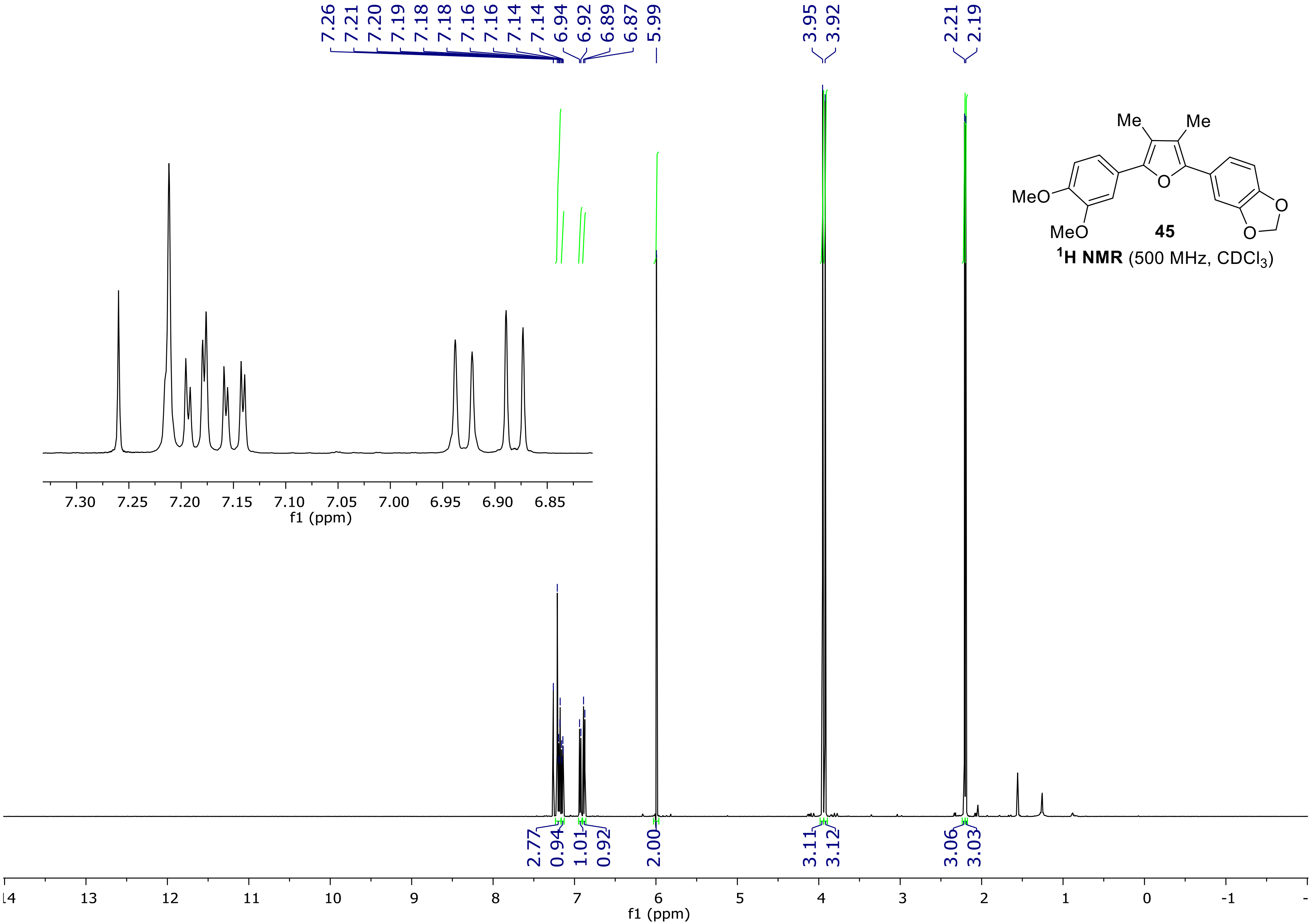

^{1}H NMR (500 MHz, $CDCl_3$)

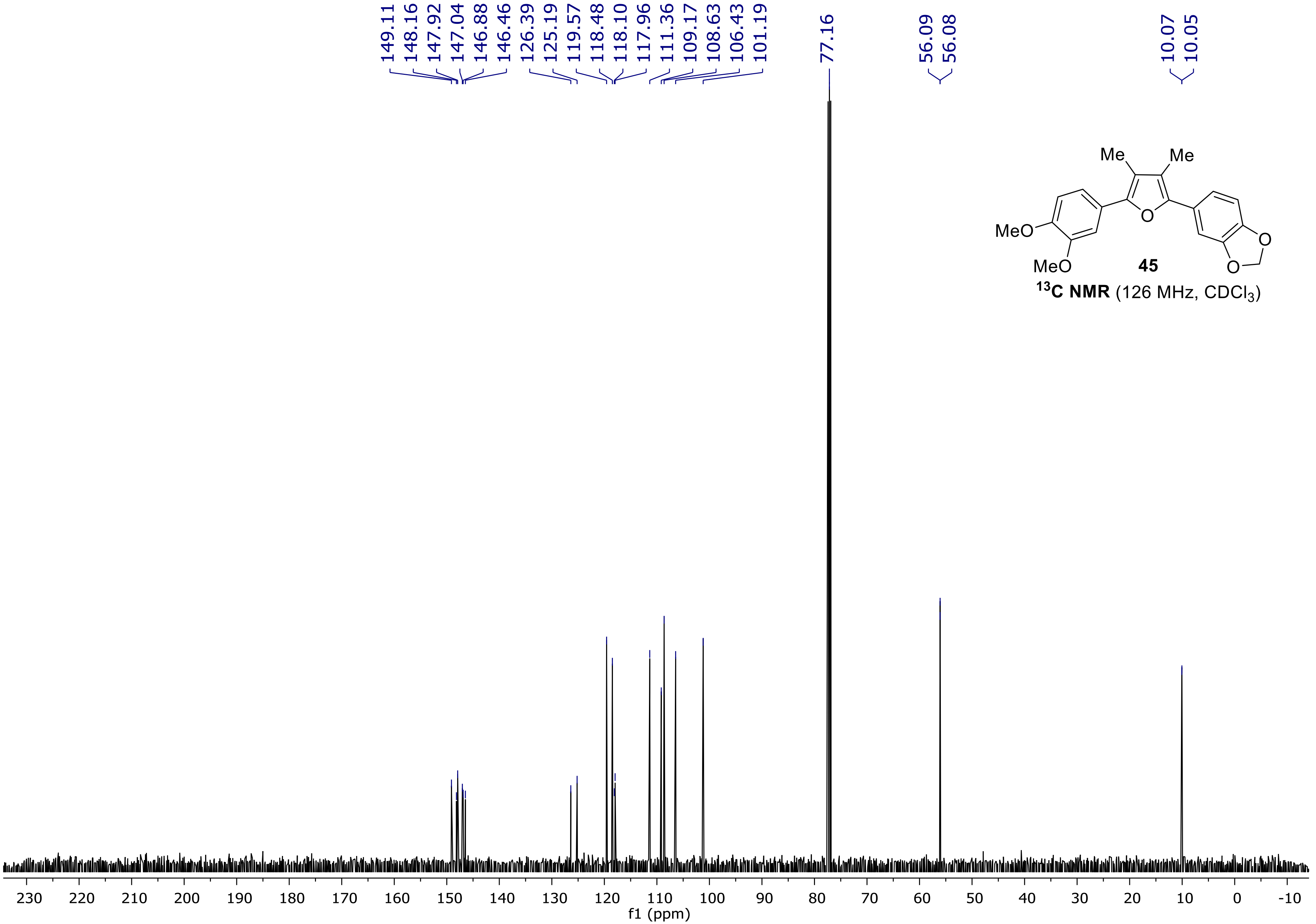

45

^{13}C NMR (126 MHz, $CDCl_3$)

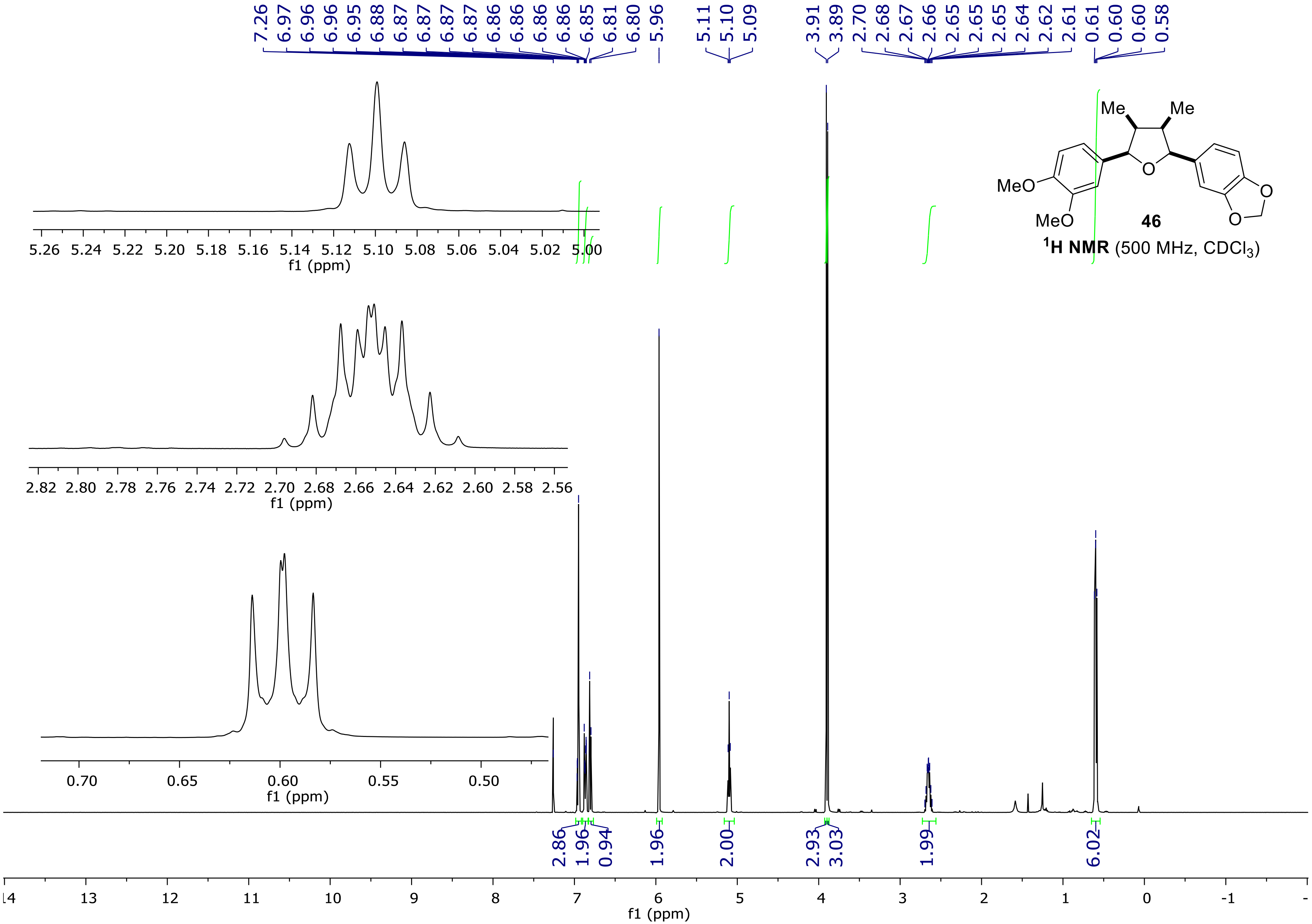

7.26 6.97 6.96 6.96 6.95 6.88 6.87 6.87 6.87 6.86 6.86 6.86 6.86 6.85 6.81 6.80 5.96 5.11 5.10 5.09 3.91 3.89 2.70 2.68 2.67 2.66 2.65 2.65 2.65 2.64 2.62 2.61 0.61 0.60 0.60 0.58
Me
Me
MeO
MeO
O
O
O
46
^{1}H NMR (500 MHz, CDCl$_3$)
f1 (ppm)
2.86 1.96 0.94 1.96 2.00 2.93 3.03 1.99 6.02

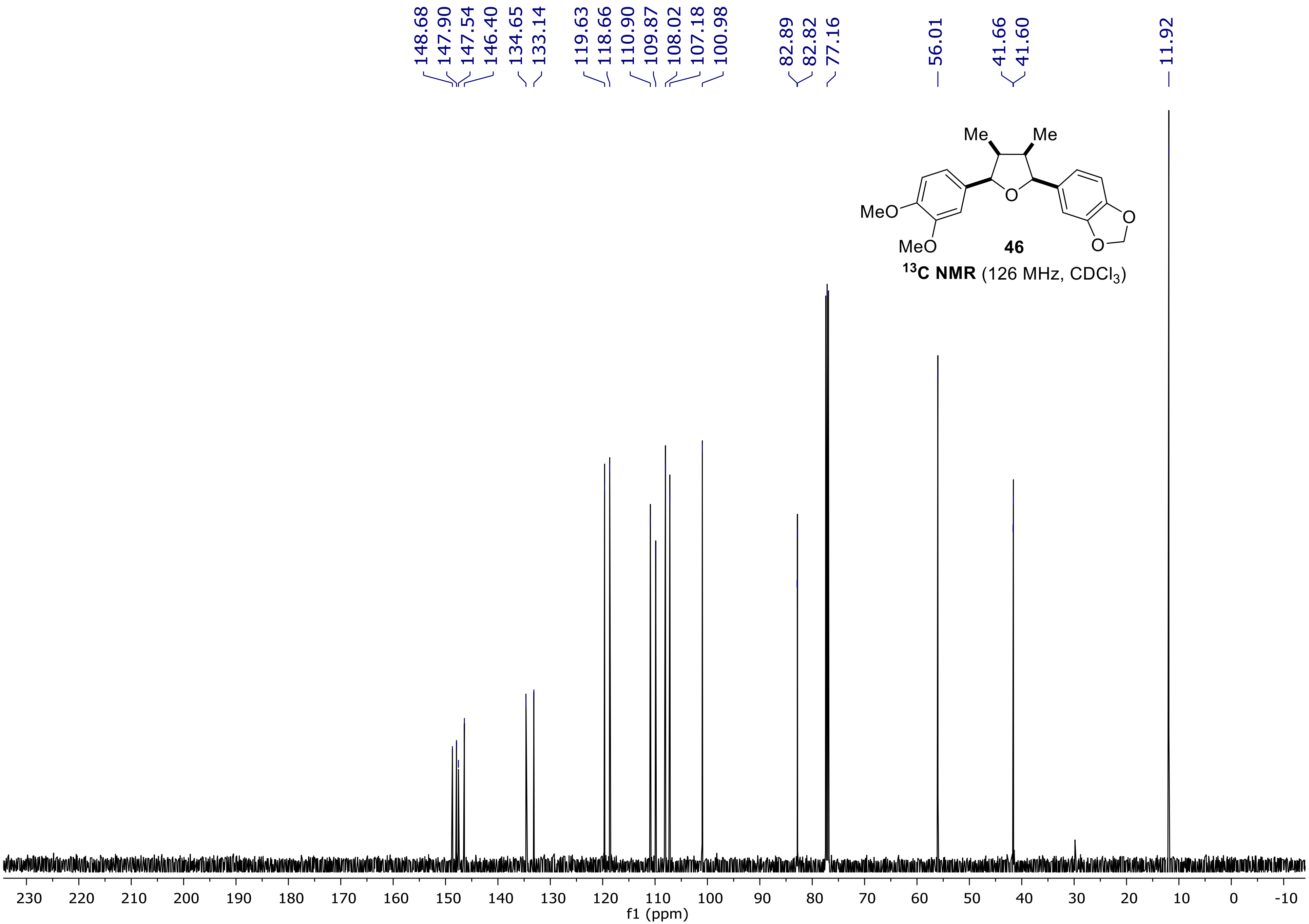
148.68
147.90
147.54
146.40
134.65
133.14
119.63
118.66
110.90
109.87
108.02
107.18
100.98
82.89
82.82
77.16
56.01
41.66
41.60
11.92
Me
Me
MeO
MeO
O
O
O
46
13C NMR (126 MHz, CDCl3)
230 220 210 200 190 180 170 160 150 140 130 120 110 100 90 80 70 60 50 40 30 20 10 0 -10
f1 (ppm)

Alfonzo 127

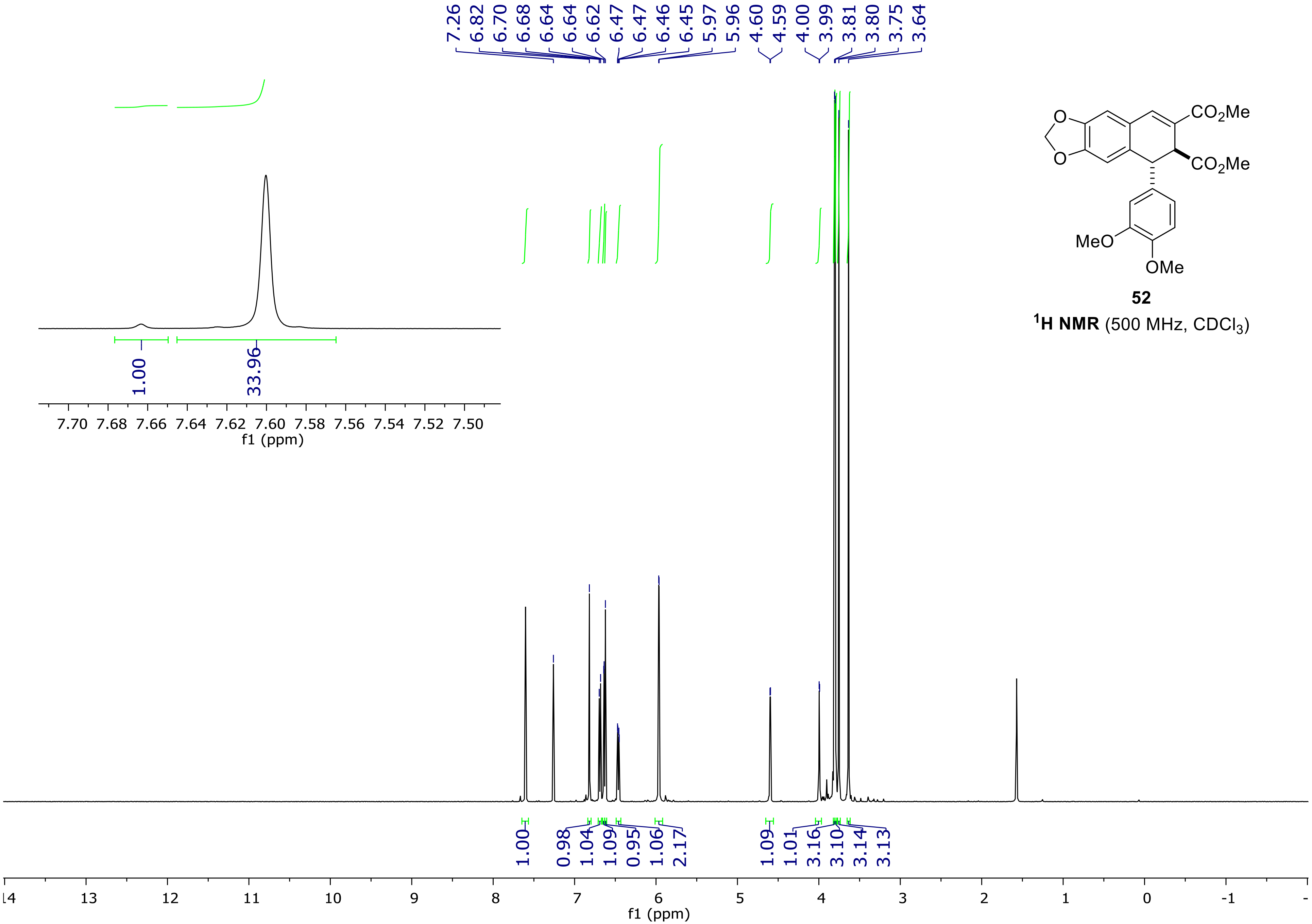

52

^{1}H NMR (500 MHz, $CDCl_3$)

Alfonzo 128

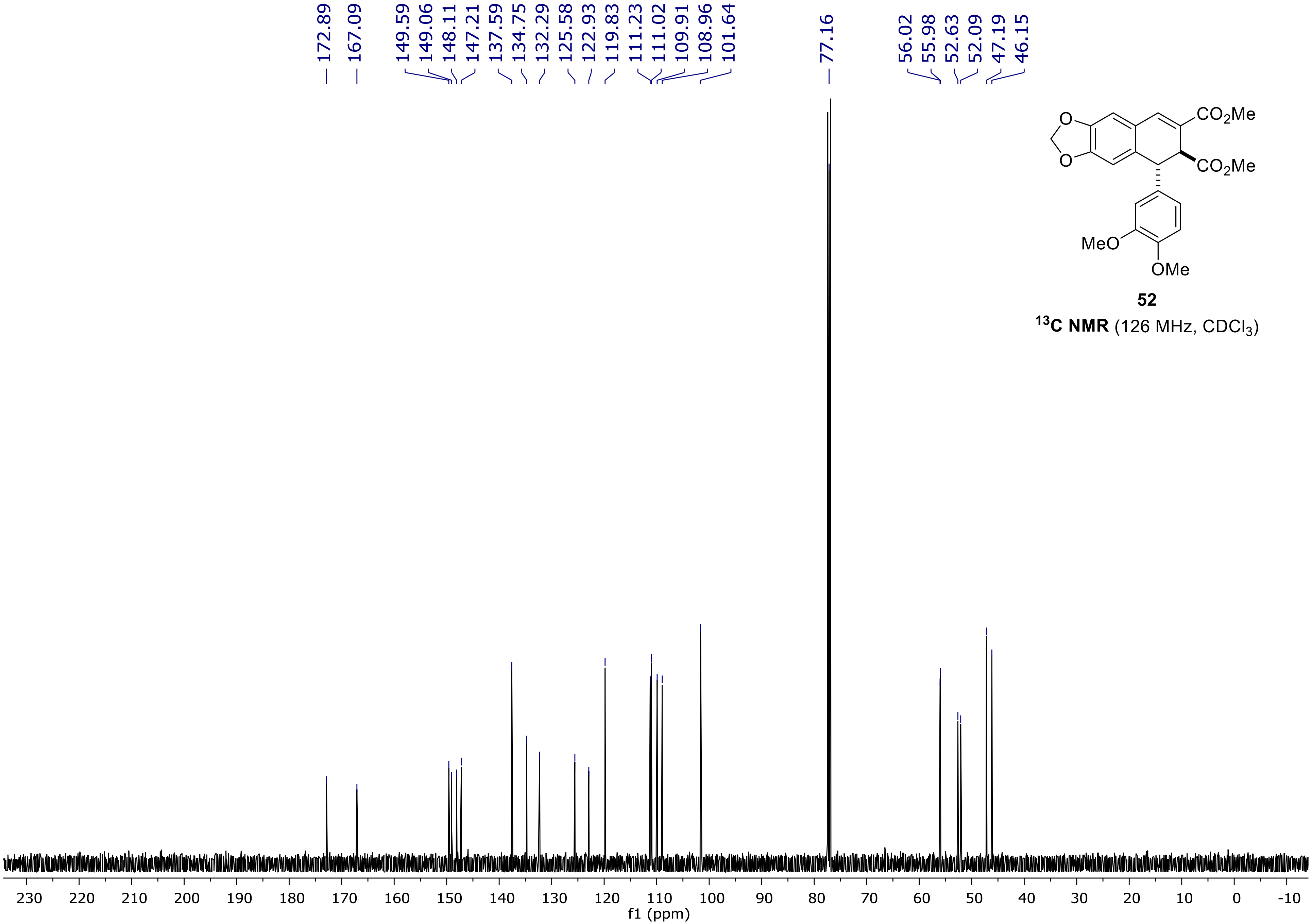

52

^{13}C NMR (126 MHz, $CDCl_3$)

Alfonzo 129

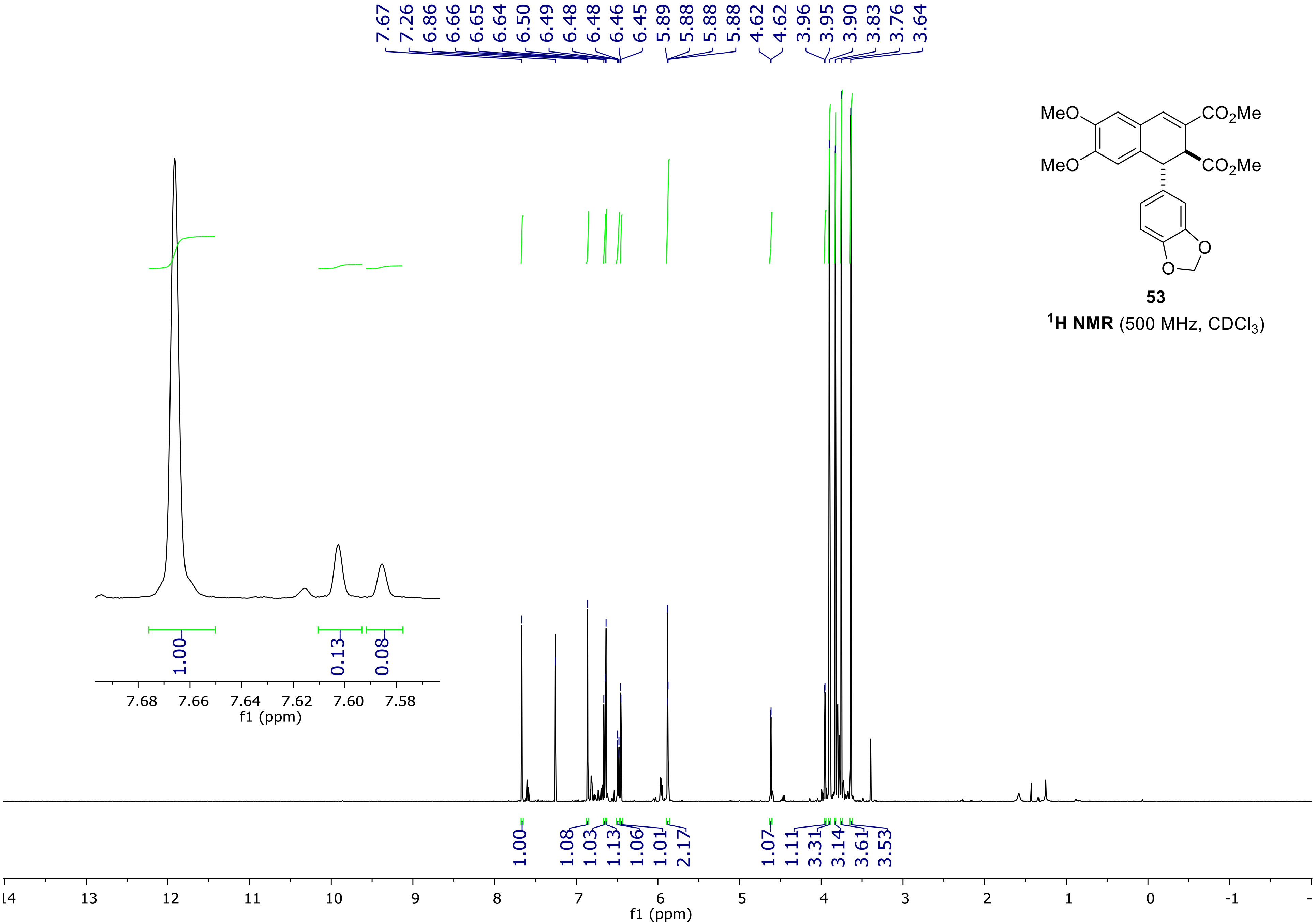

53

^{1}H NMR (500 MHz, $CDCl_3$)

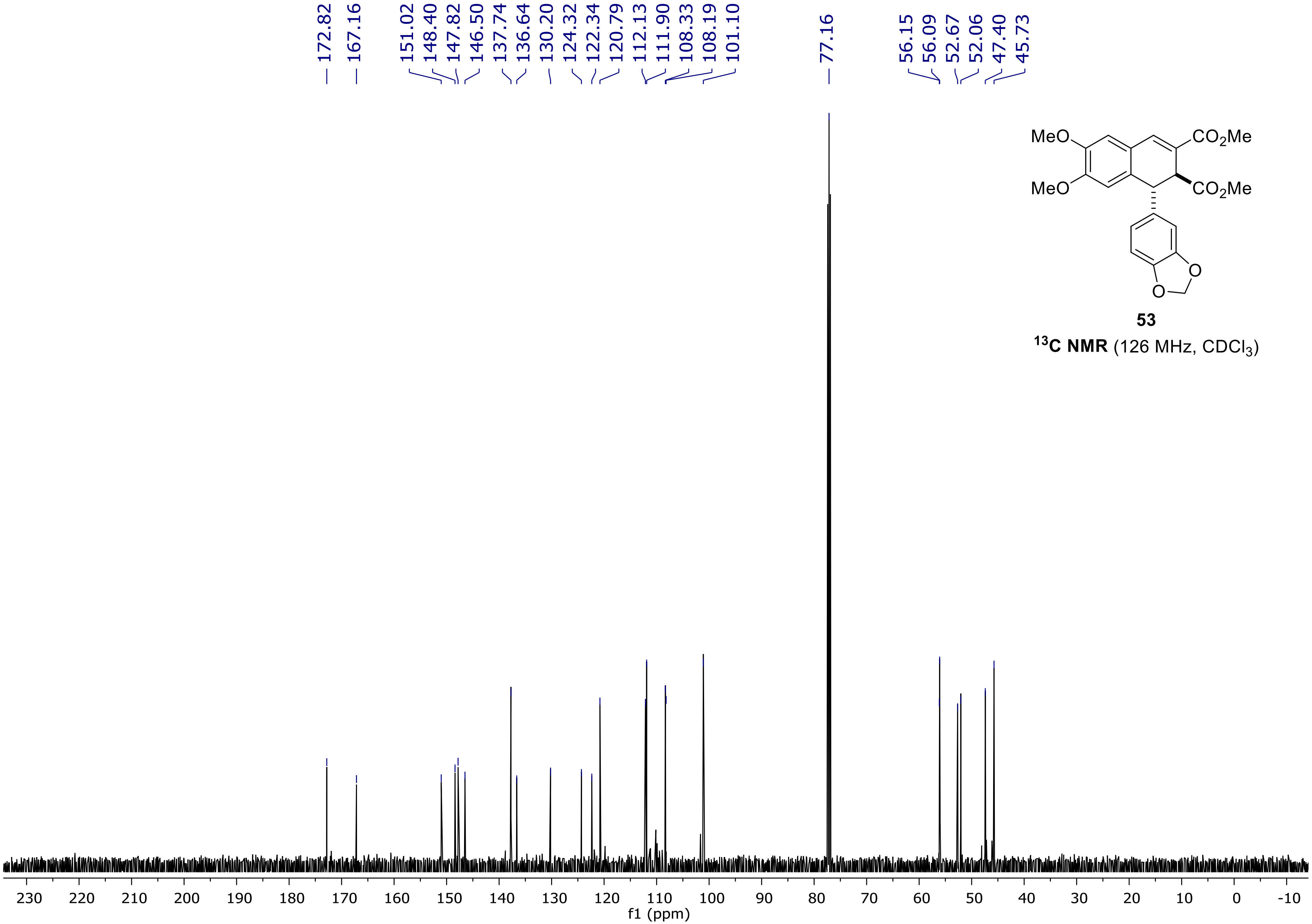

53

^{13}C NMR (126 MHz, $CDCl_3$)

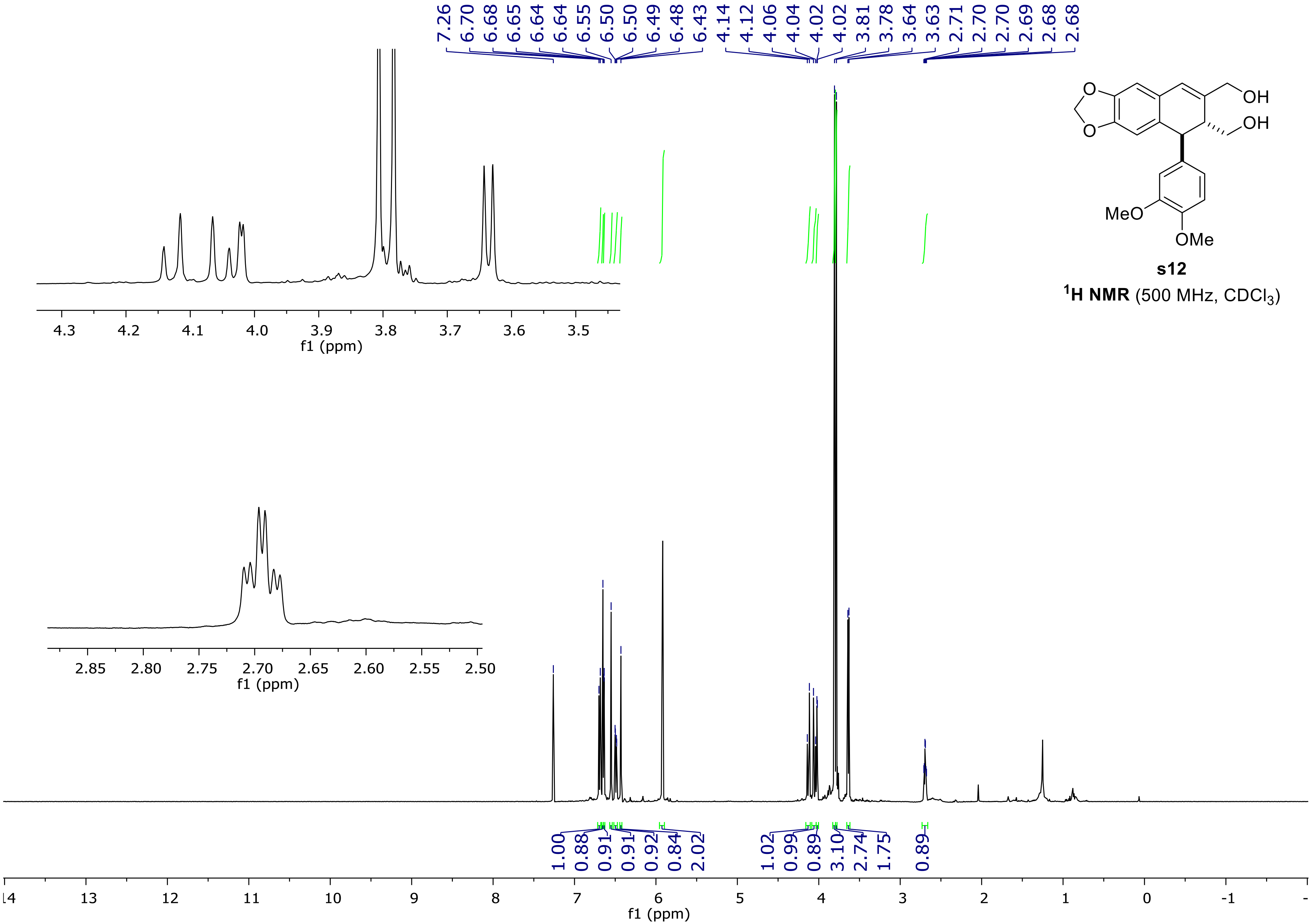

s12

^{1}H NMR (500 MHz, $CDCl_3$)

Alfonzo 132

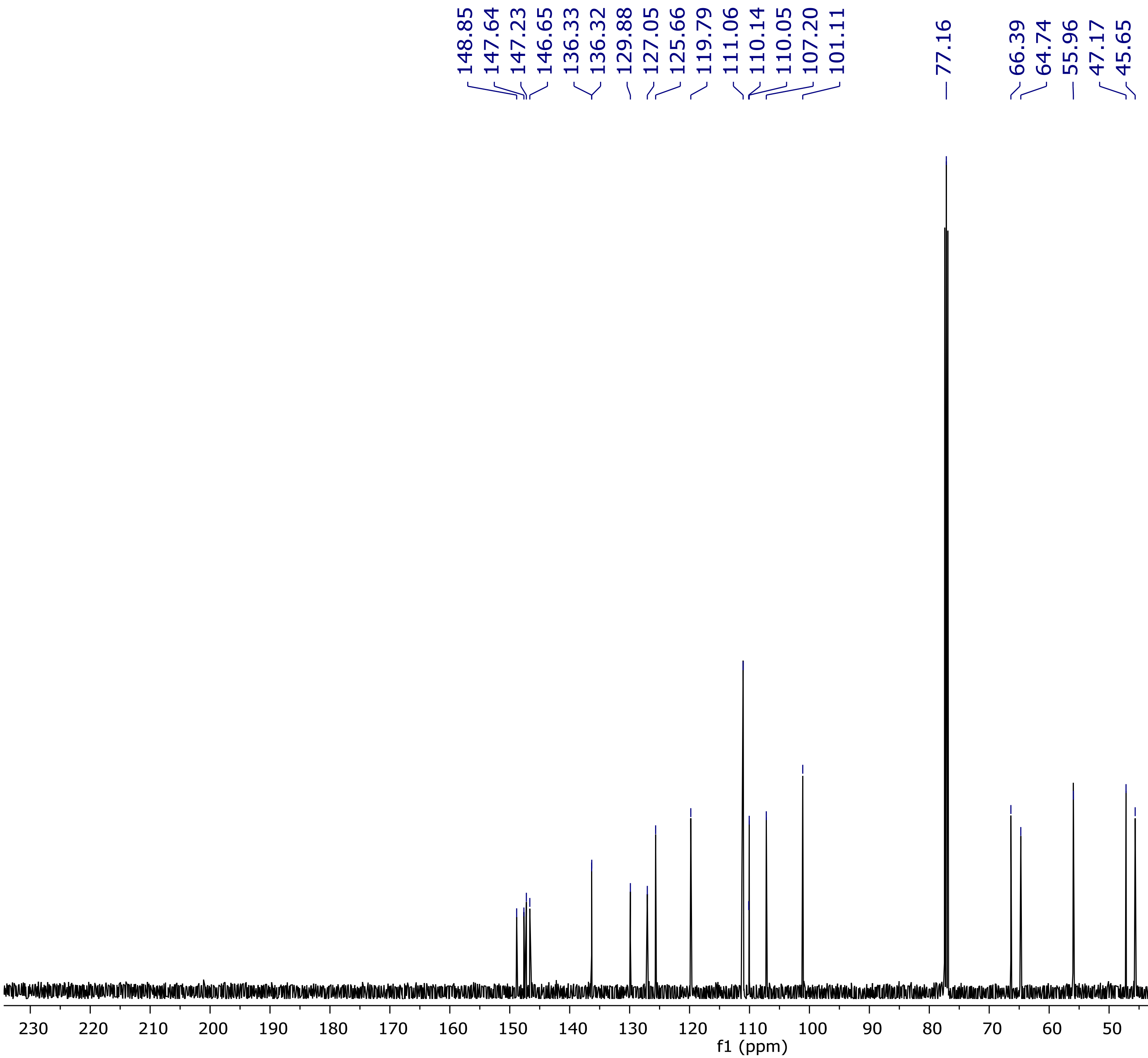

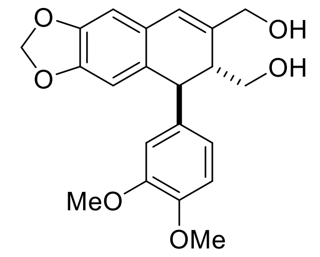

s12

^{13}C NMR (126 MHz, $CDCl_3$)

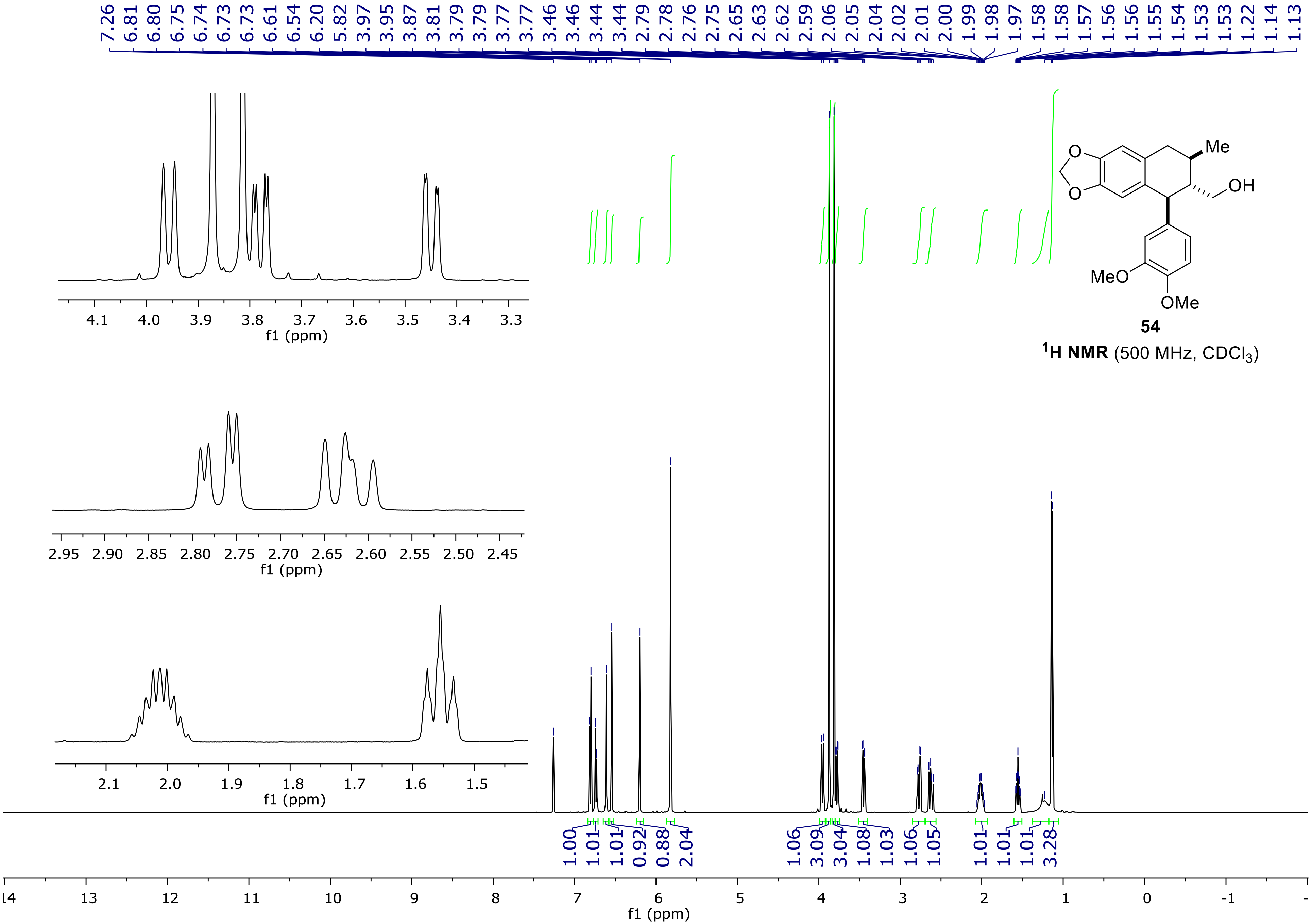
7.26 6.81 6.80 6.75 6.74 6.73 6.73 6.61 6.54 6.20 5.82 3.97 3.95 3.87 3.81 3.79 3.79 3.77 3.77 3.46 3.46 3.44 3.44 2.79 2.78 2.76 2.75 2.65 2.63 2.62 2.59 2.06 2.05 2.04 2.02 2.01 2.00 1.99 1.98 1.97 1.58 1.58 1.57 1.56 1.56 1.55 1.54 1.53 1.53 1.22 1.14 1.13
Me
O
O
OH
MeO
OMe
54
¹H NMR (500 MHz, CDCl₃)
1.00 1.01 1.01 0.92 0.88 2.04 1.06 3.09 3.04 1.08 1.03 1.06 1.05 1.01 1.01 1.01 3.28
f1 (ppm)

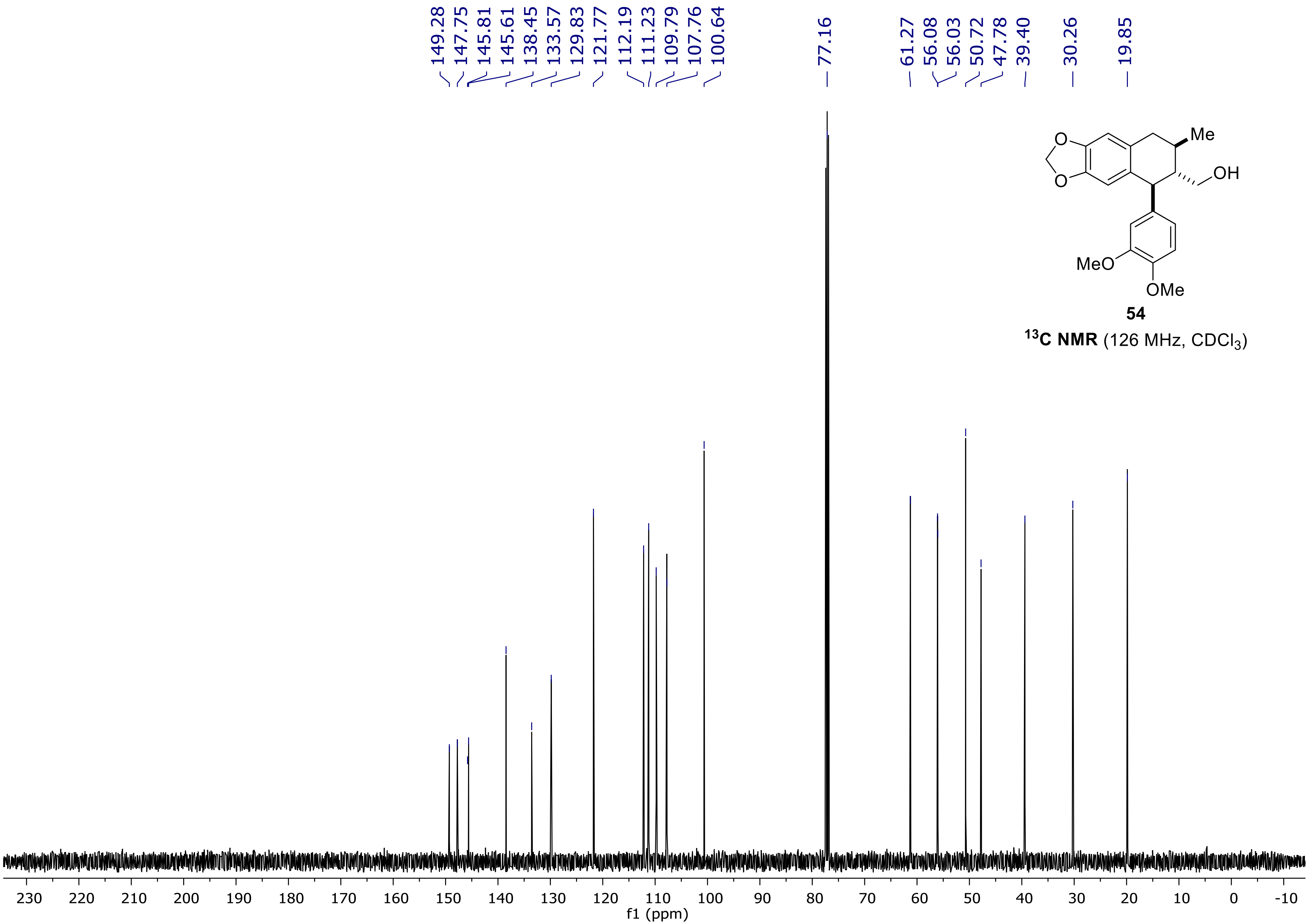
149.28
147.75
145.81
145.61
138.45
133.57
129.83
121.77
112.19
111.23
109.79
107.76
100.64
77.16
61.27
56.08
56.03
50.72
47.78
39.40
30.26
19.85
Me
OH
MeO
OMe
54
^{13}C NMR (126 MHz, CDCl$_3$)
230 220 210 200 190 180 170 160 150 140 130 120 110 100 90 80 70 60 50 40 30 20 10 0 -10
f1 (ppm)

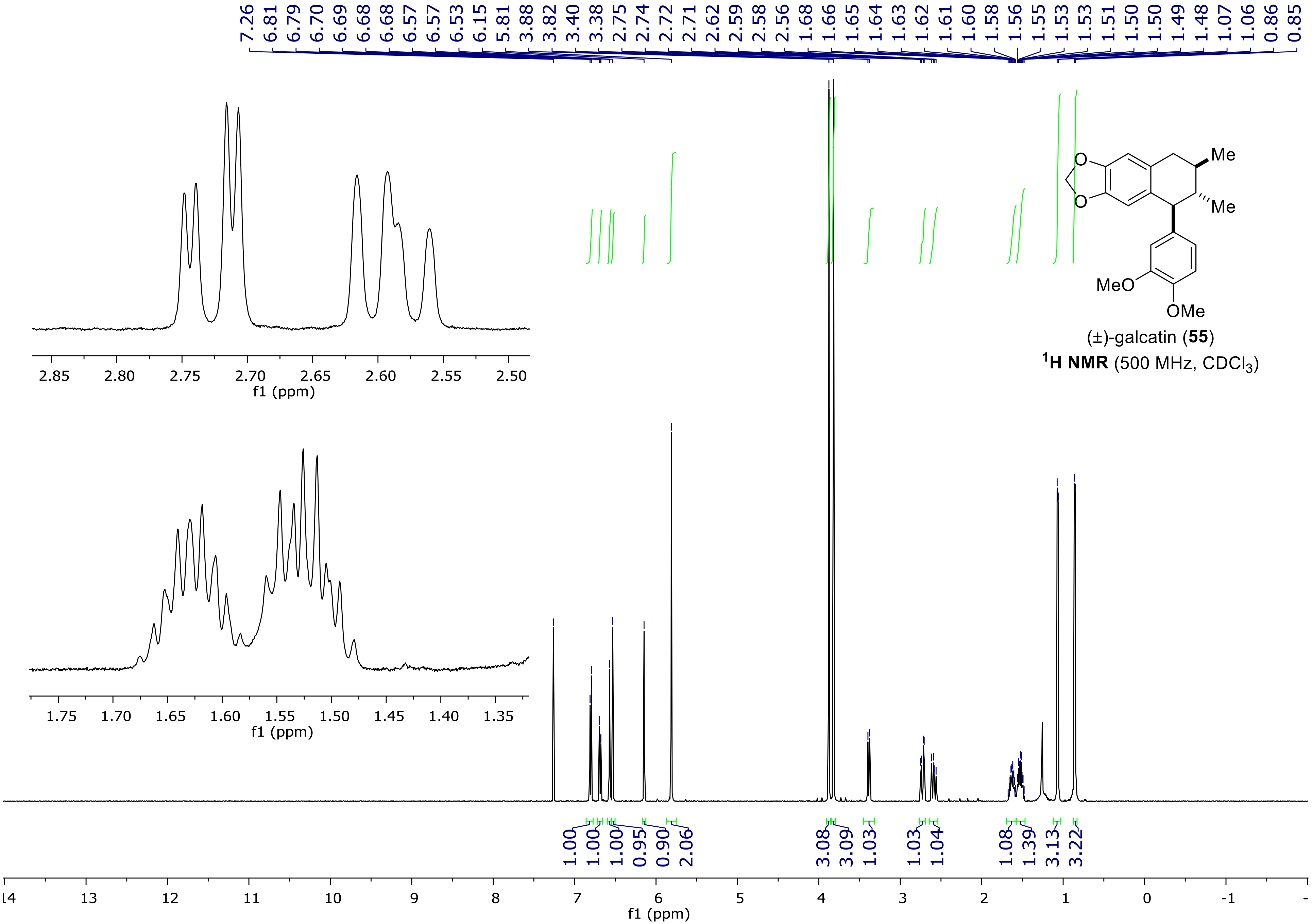

7.26 6.81 6.79 6.70 6.69 6.68 6.68 6.57 6.57 6.53 6.15 5.81 3.88 3.82 3.40 3.38 2.75 2.74 2.72 2.71 2.62 2.59 2.58 2.56 1.68 1.66 1.65 1.64 1.63 1.62 1.61 1.60 1.58 1.56 1.55 1.53 1.53 1.51 1.50 1.50 1.49 1.48 1.07 1.06 0.86 0.85
Me
Me
O
O
MeO
OMe
(±)-galcatin (55)
1H NMR (500 MHz, CDCl3)
f1 (ppm)
1.00 1.00 1.00 0.95 0.90 2.06 3.08 3.09 1.03 1.03 1.04 1.08 1.39 3.13 3.22

Alfonzo 136

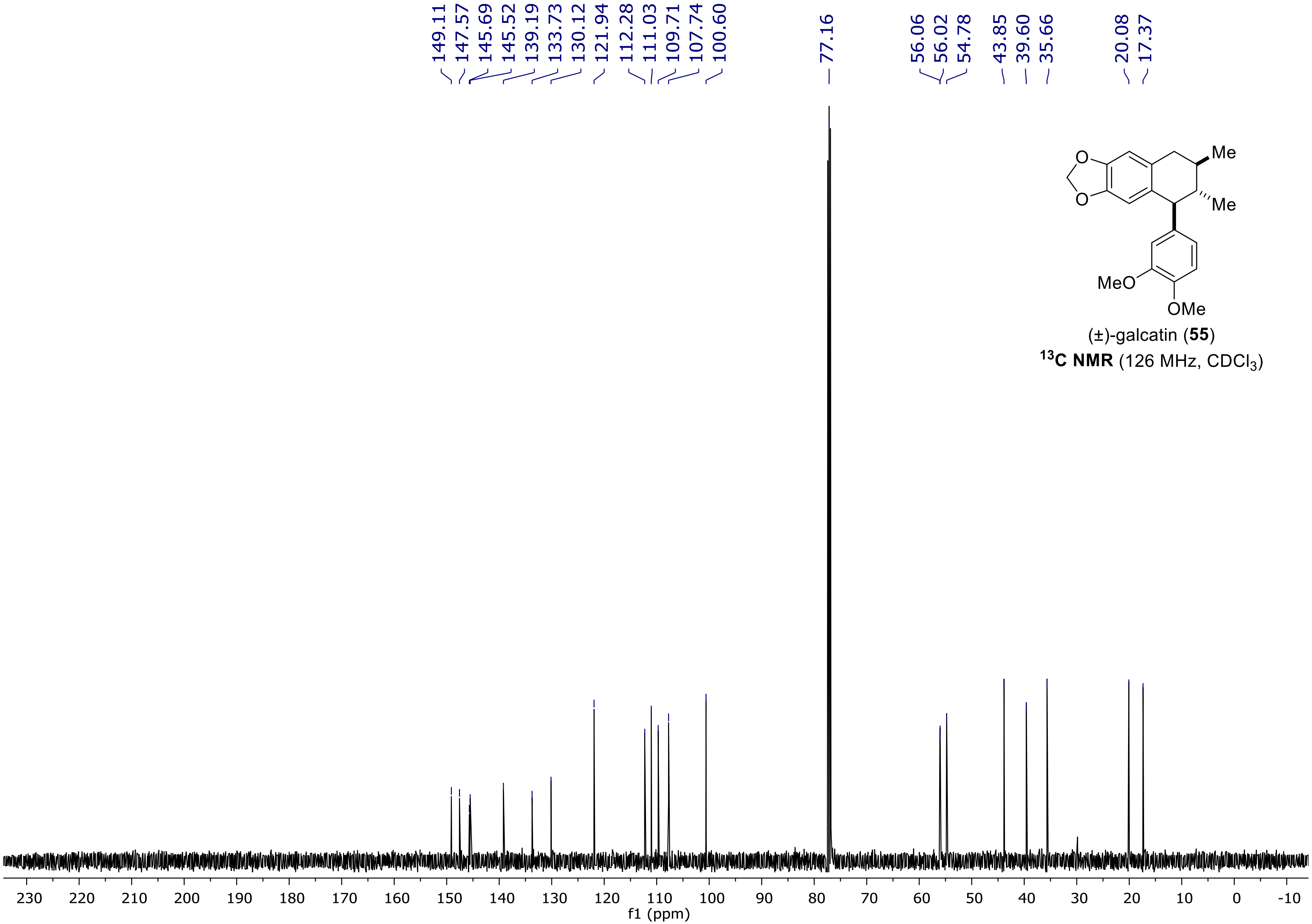

(±)-galcatin (**55**)

^{13}C NMR (126 MHz, $CDCl_3$)

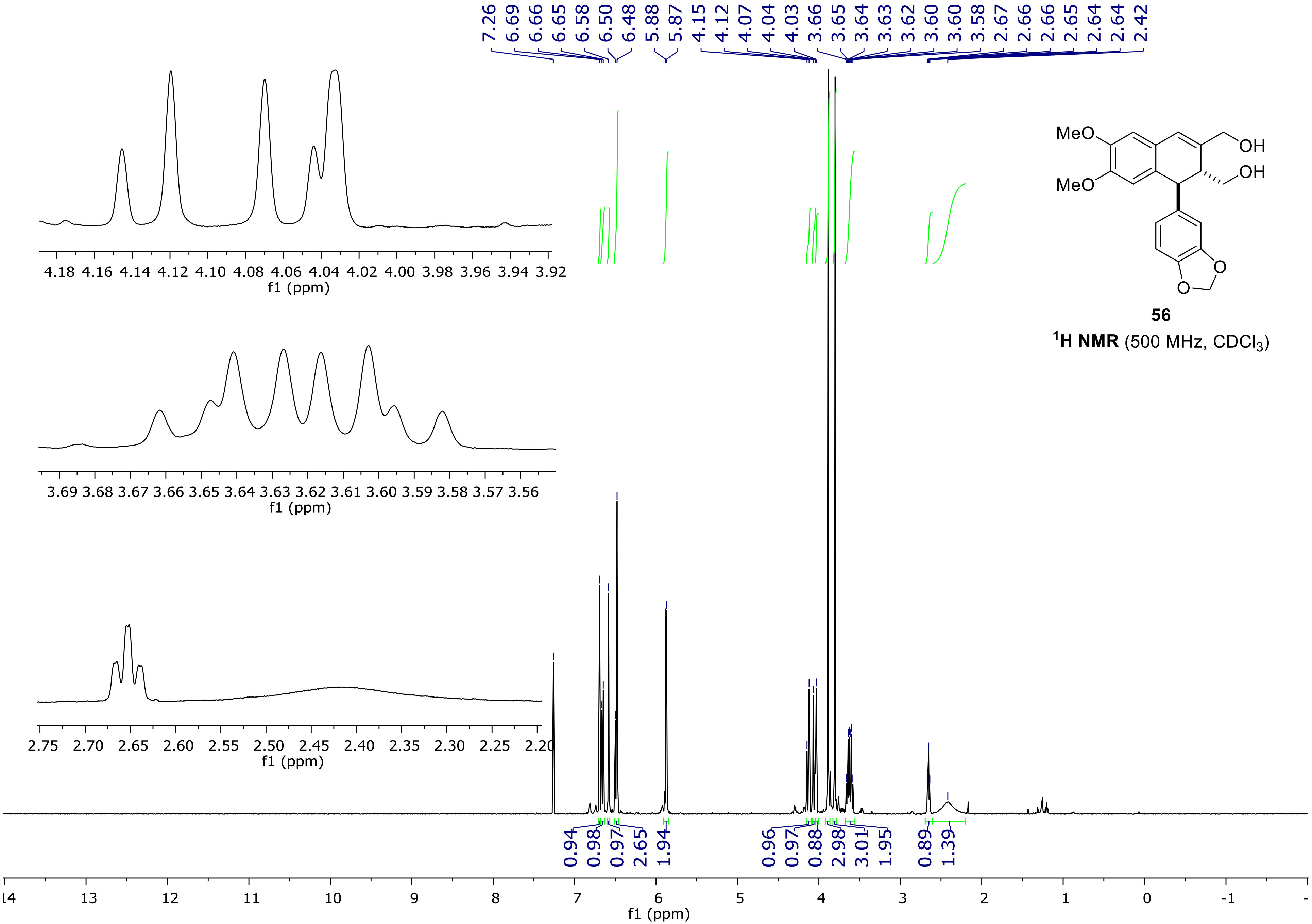

56

^{1}H NMR (500 MHz, $CDCl_3$)

Alfonzo 138

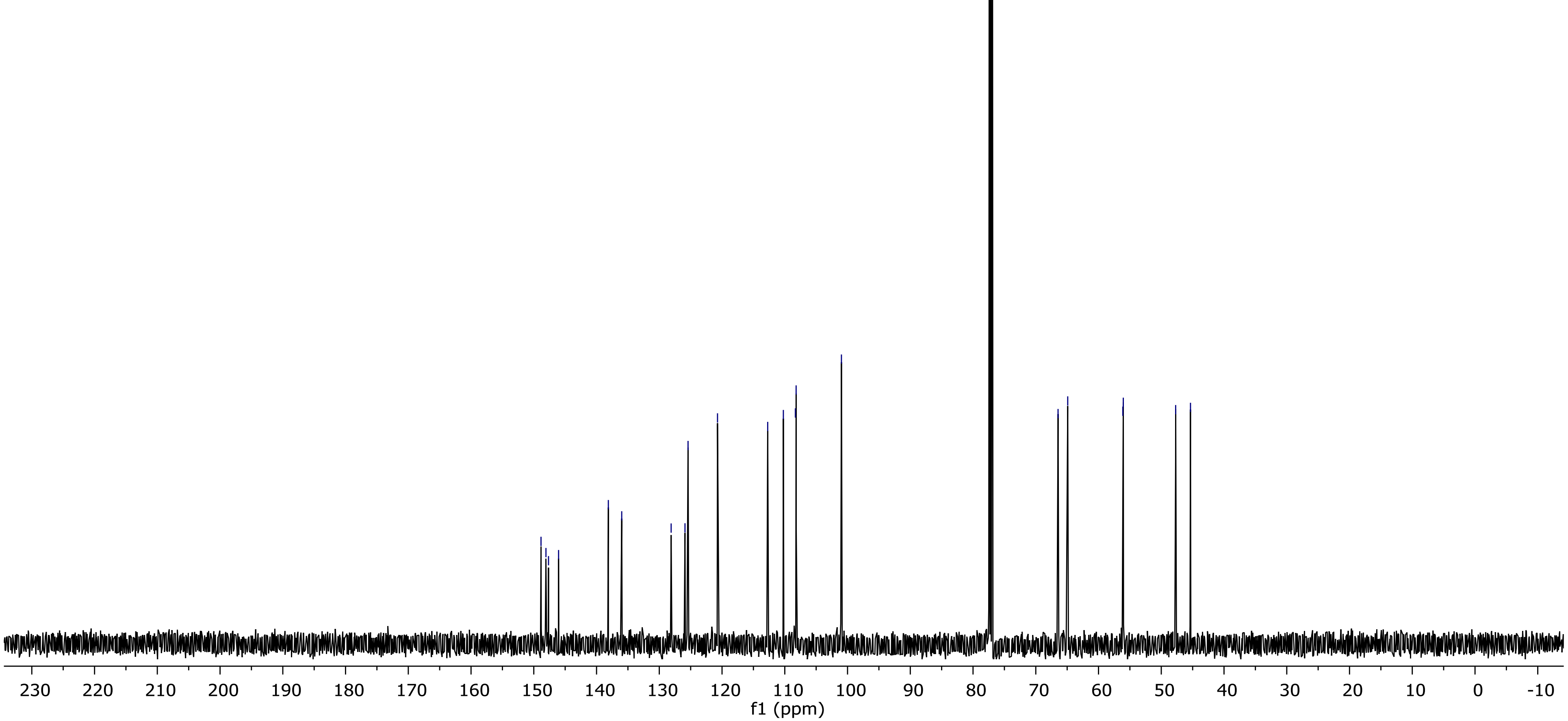

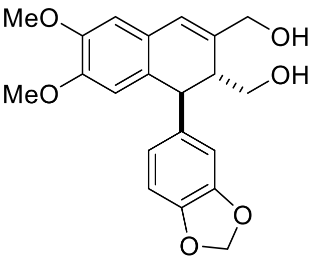

56

^{13}C NMR (126 MHz, $CDCl_3$)

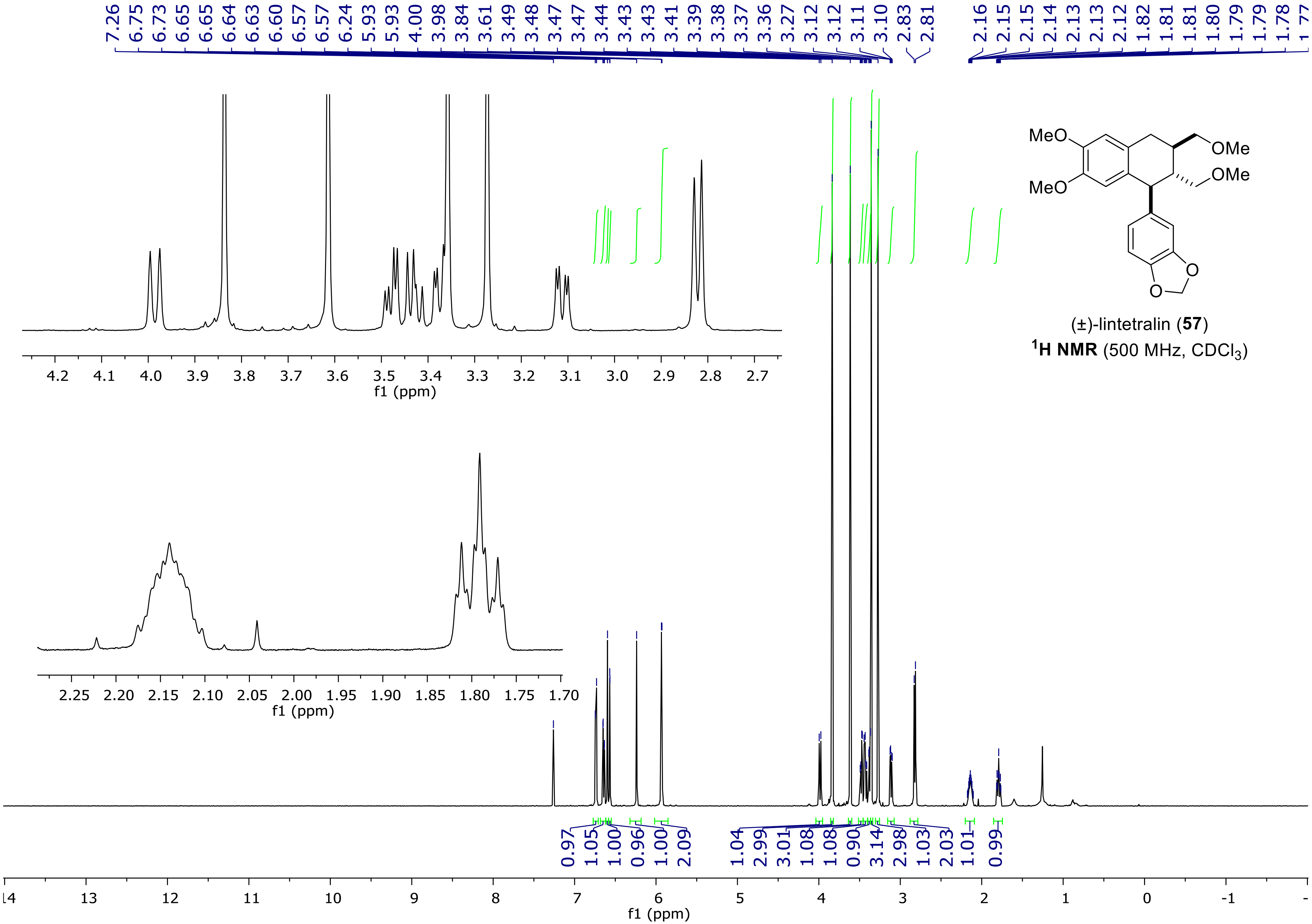

(±)-lintetralin (**57**)

^{1}H NMR (500 MHz, $CDCl_3$)

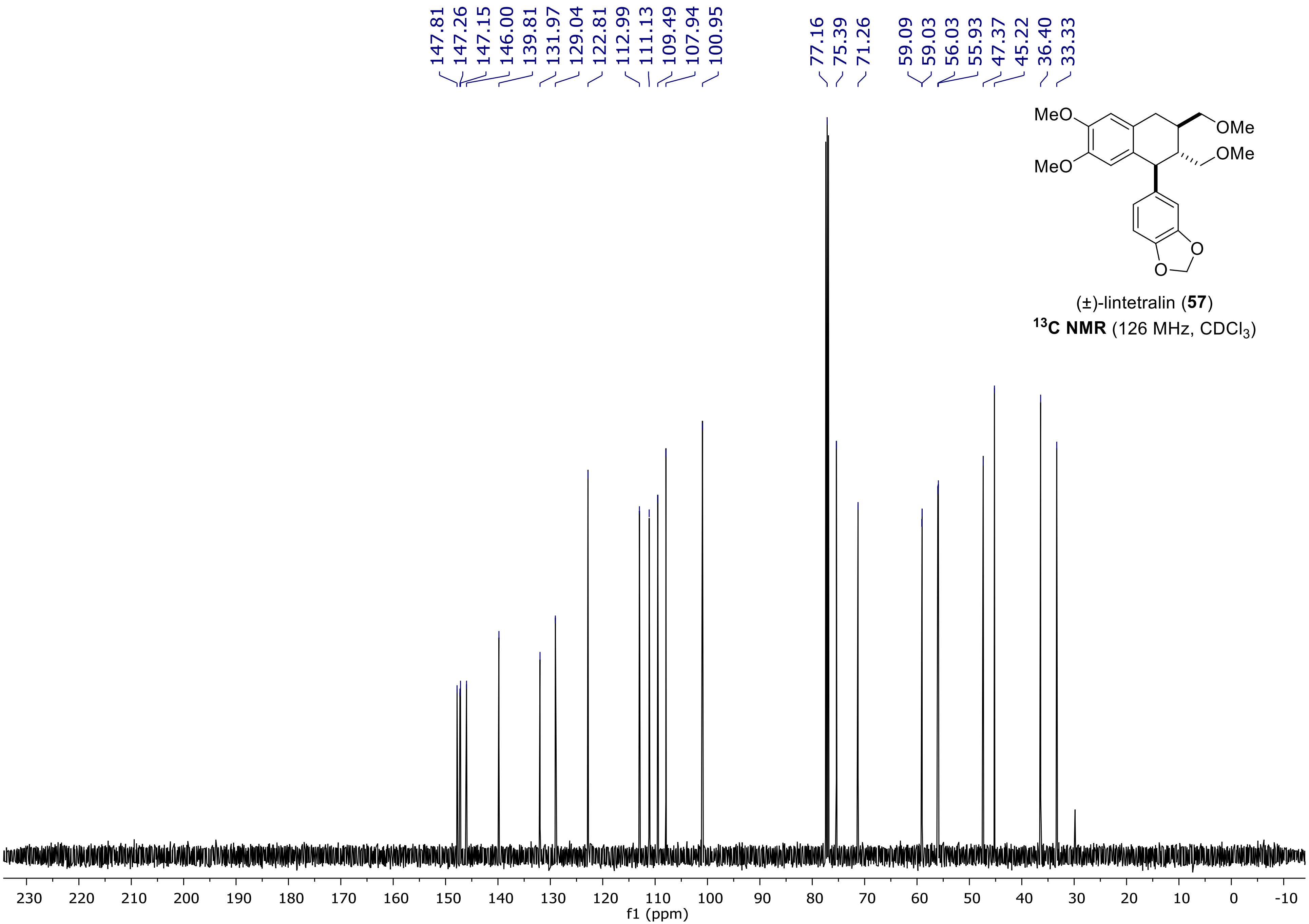

(±)-lintetralin (**57**)

^{13}C NMR (126 MHz, $CDCl_3$)

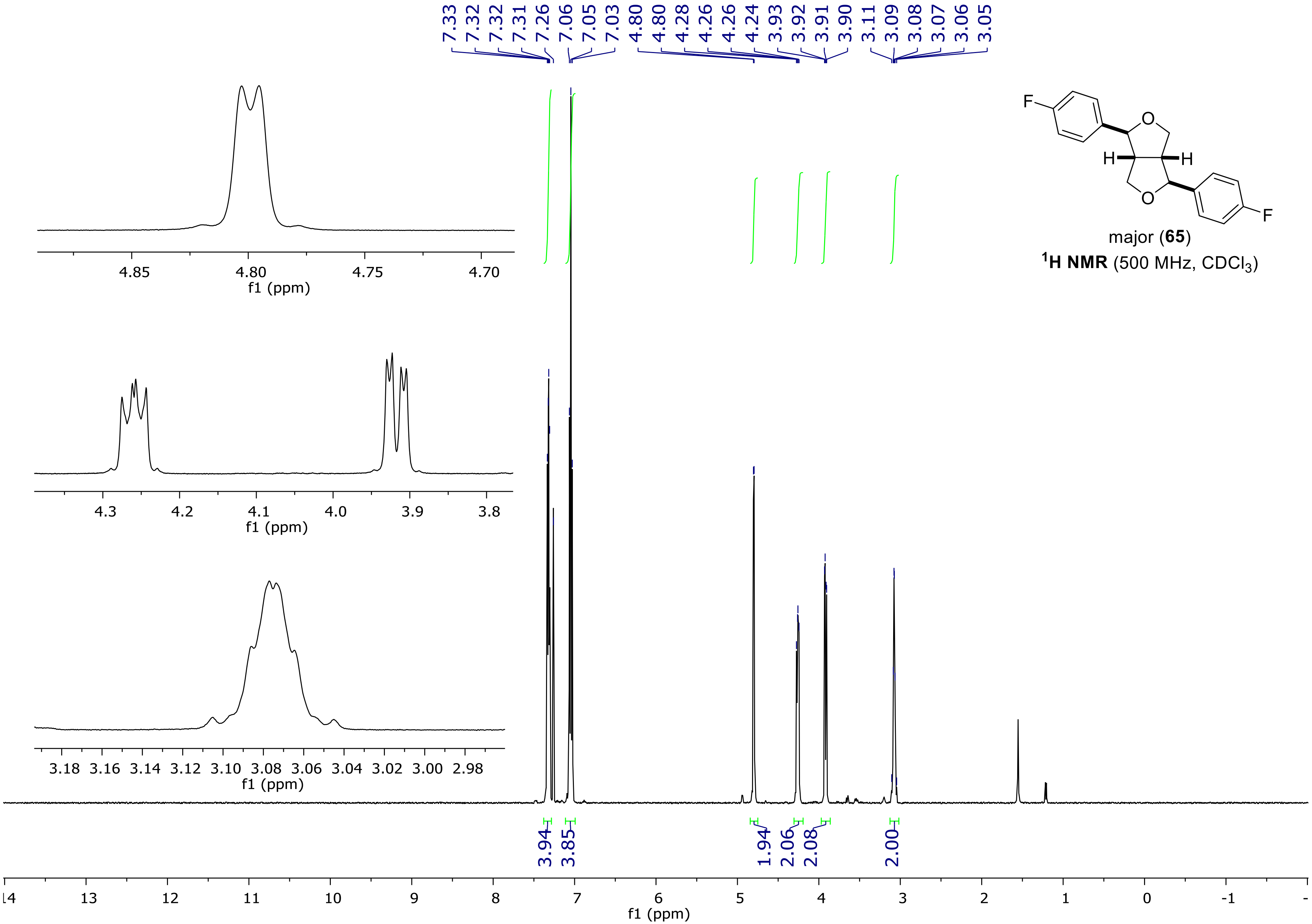

major (**65**)

^{1}H NMR (500 MHz, $CDCl_3$)

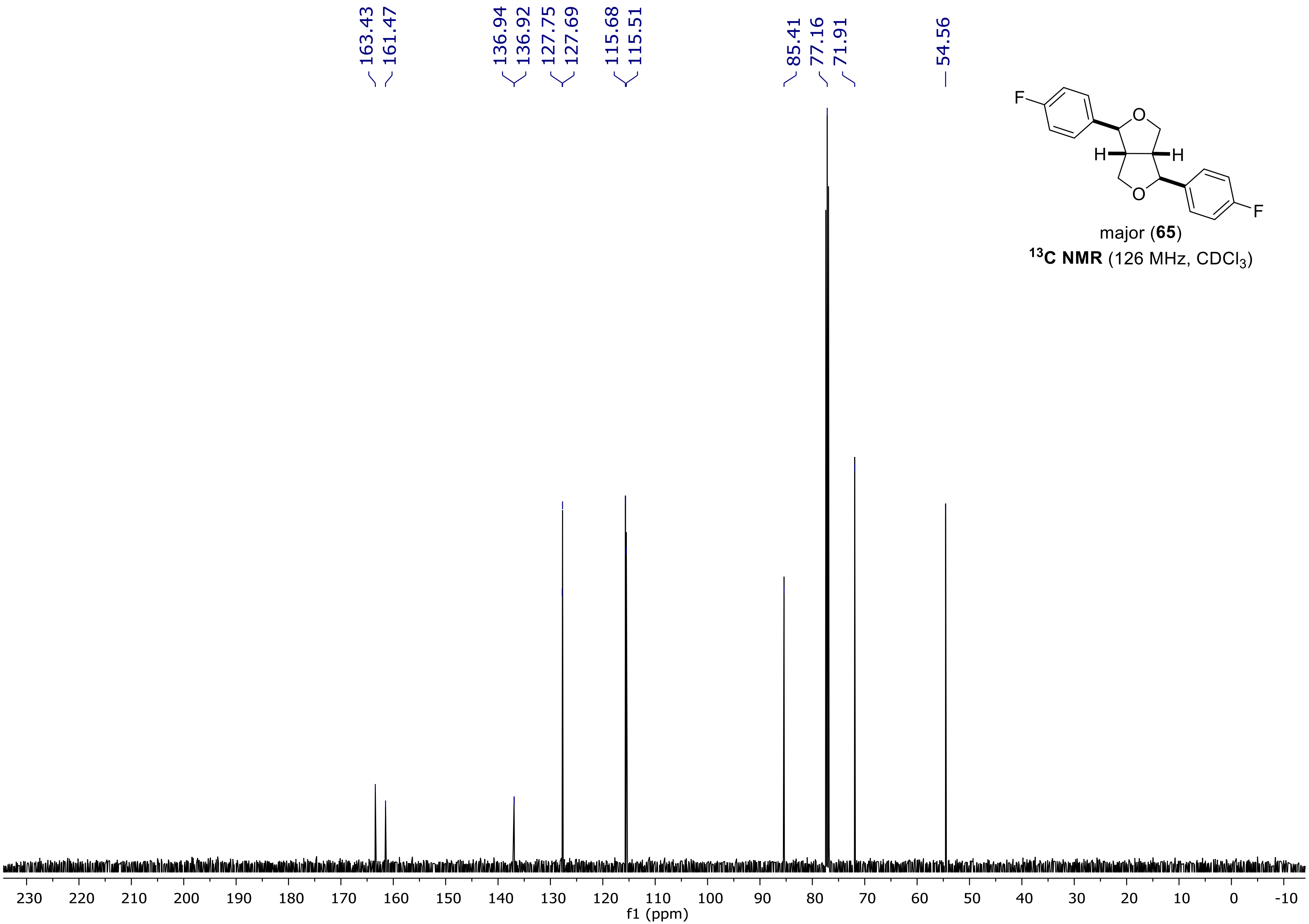

major (**65**)

^{13}C NMR (126 MHz, $CDCl_3$)

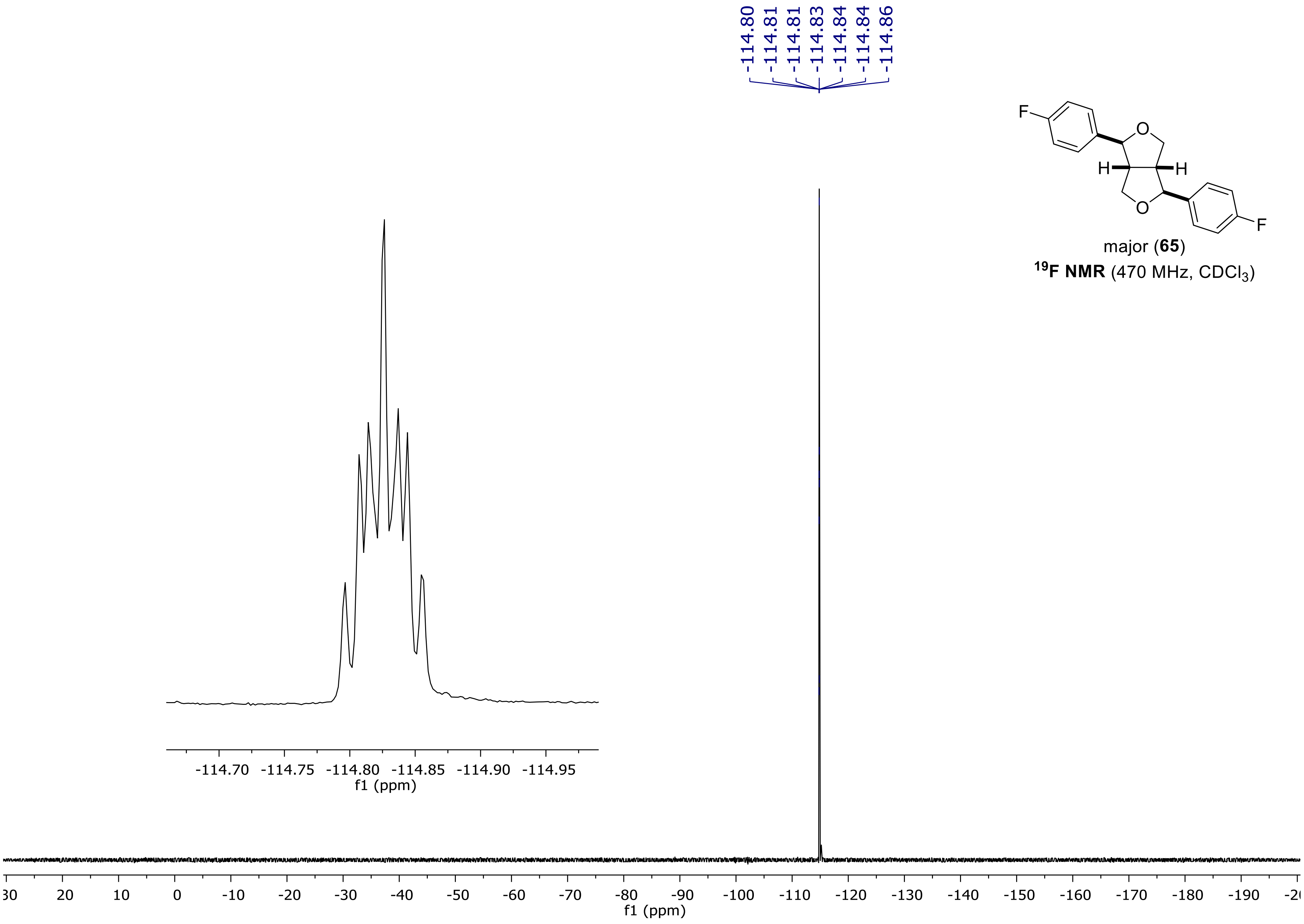

major (**65**)

^{19}F NMR (470 MHz, $CDCl_3$)

Alfonzo 144

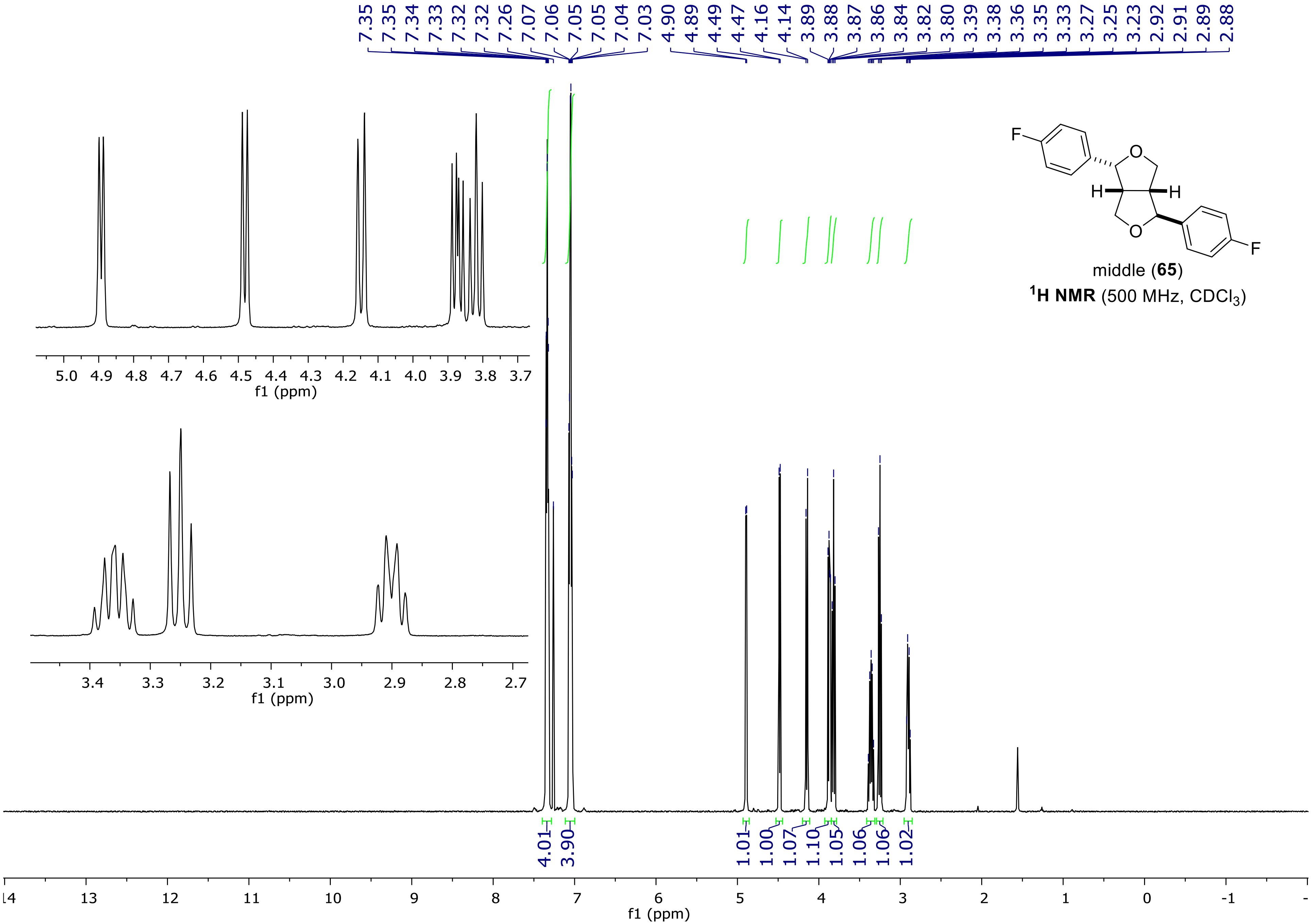
7.35
7.35
7.34
7.33
7.32
7.32
7.26
7.07
7.06
7.05
7.05
7.04
7.03
4.90
4.89
4.49
4.47
4.16
4.14
3.89
3.88
3.87
3.86
3.84
3.82
3.80
3.39
3.38
3.36
3.35
3.33
3.27
3.25
3.23
2.92
2.91
2.89
2.88
F
O
H
H
O
F
middle (65)
1H NMR (500 MHz, CDCl3)
f1 (ppm)
4.01
3.90
1.01
1.00
1.07
1.10
1.05
1.06
1.06
1.02

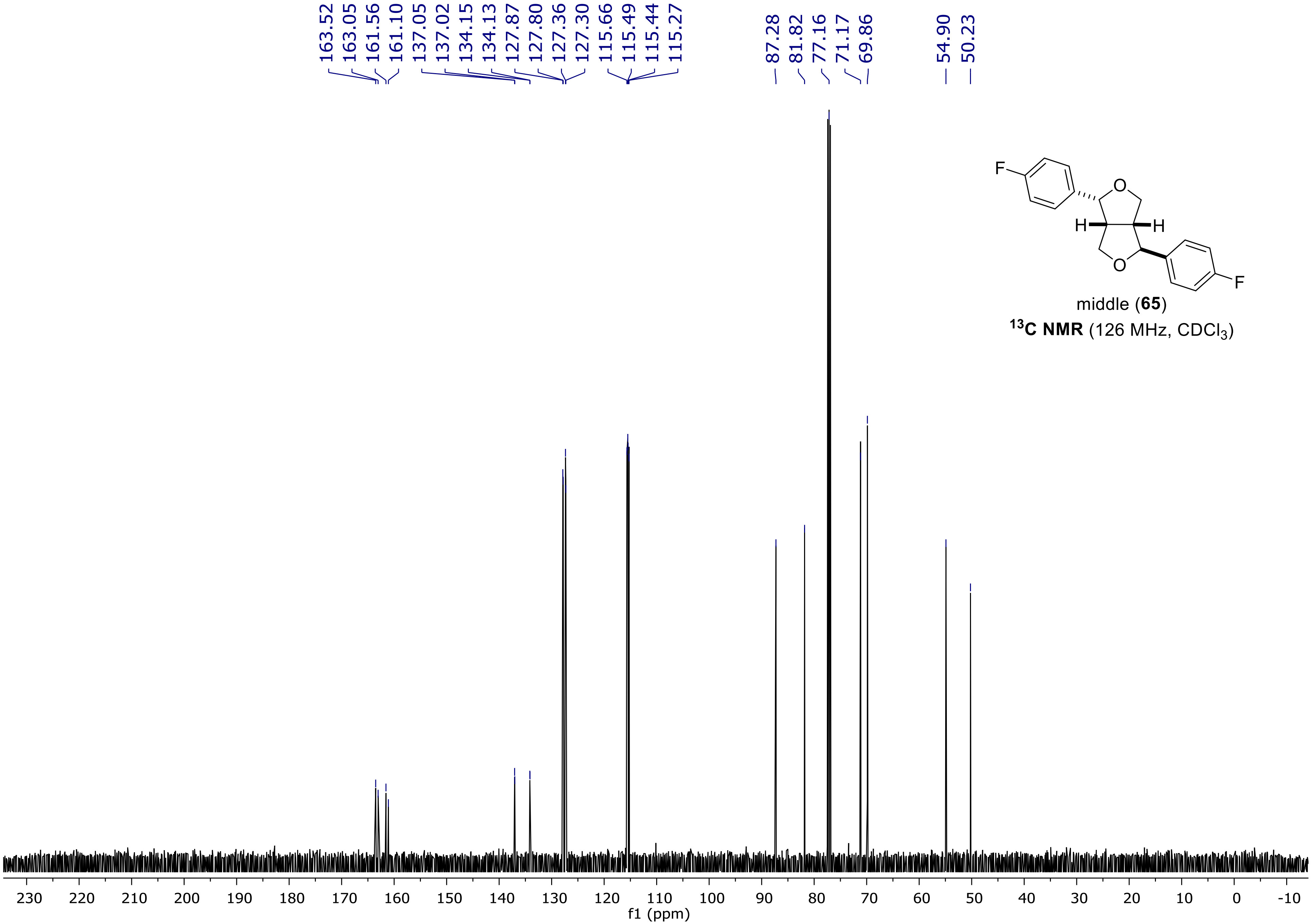

middle (**65**)

^{13}C NMR (126 MHz, $CDCl_3$)

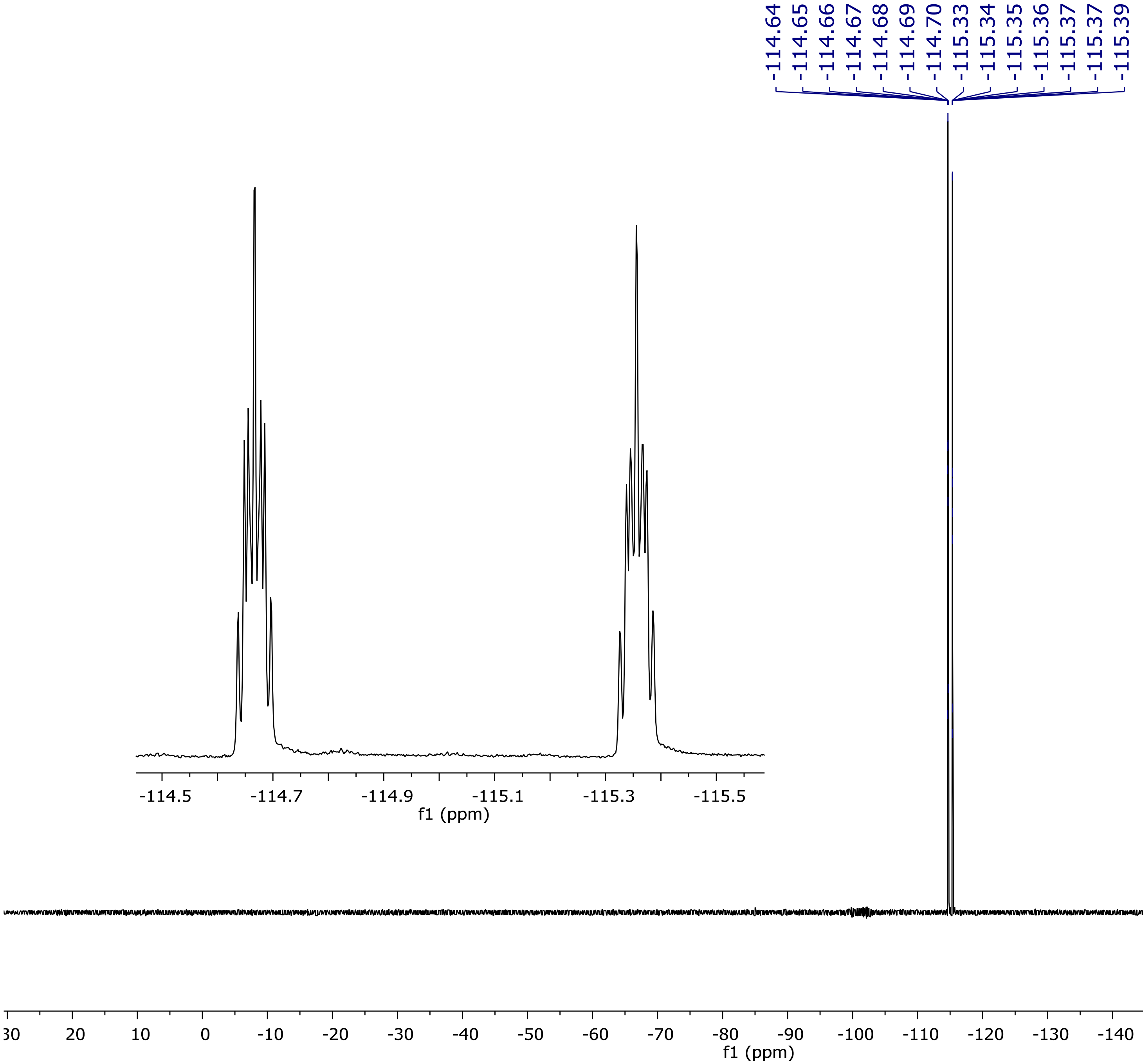

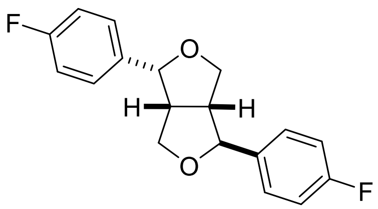

middle (**65**)

^{19}F NMR (470 MHz, $CDCl_3$)

Alfonzo 147

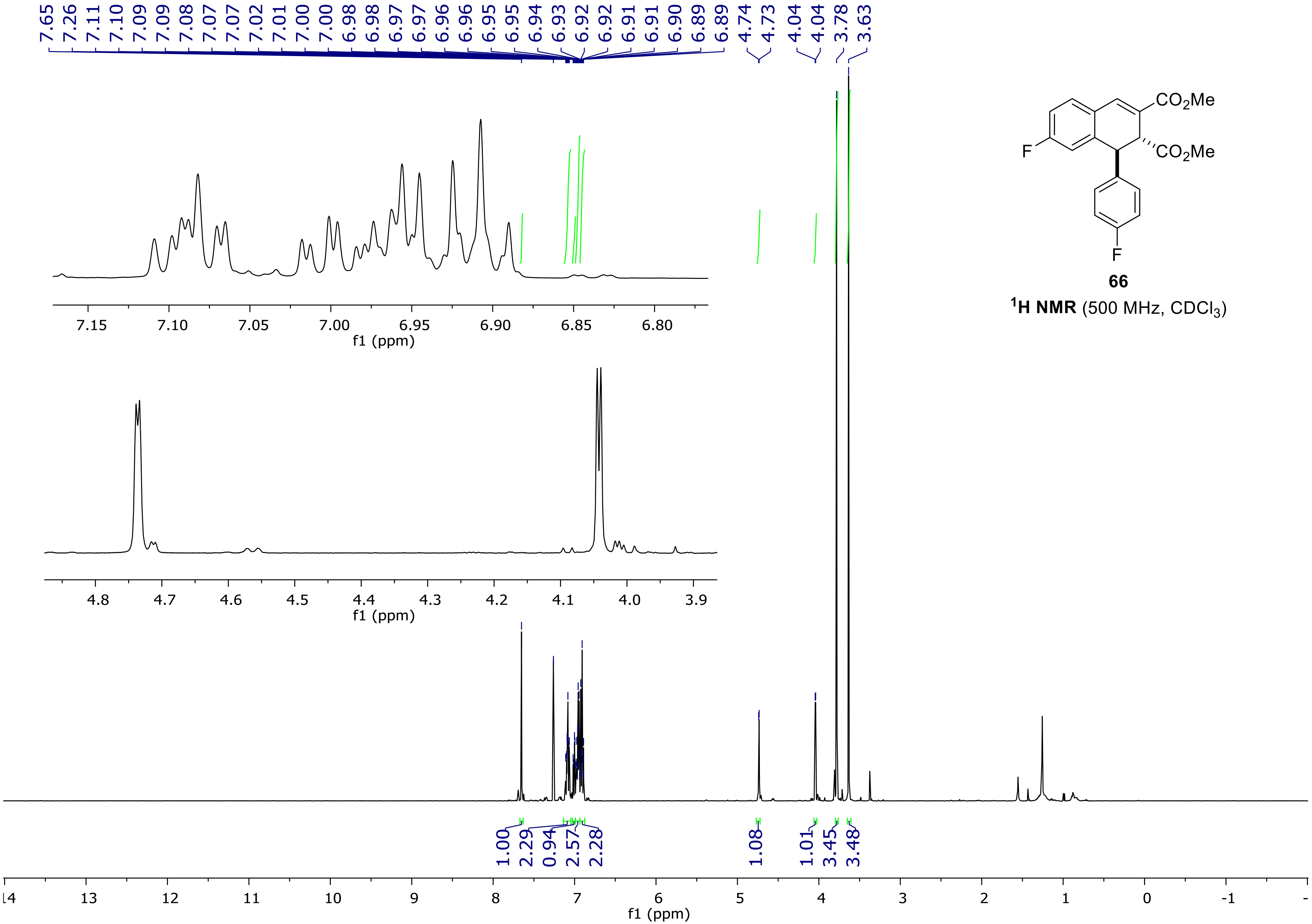

66

^{1}H NMR (500 MHz, $CDCl_3$)

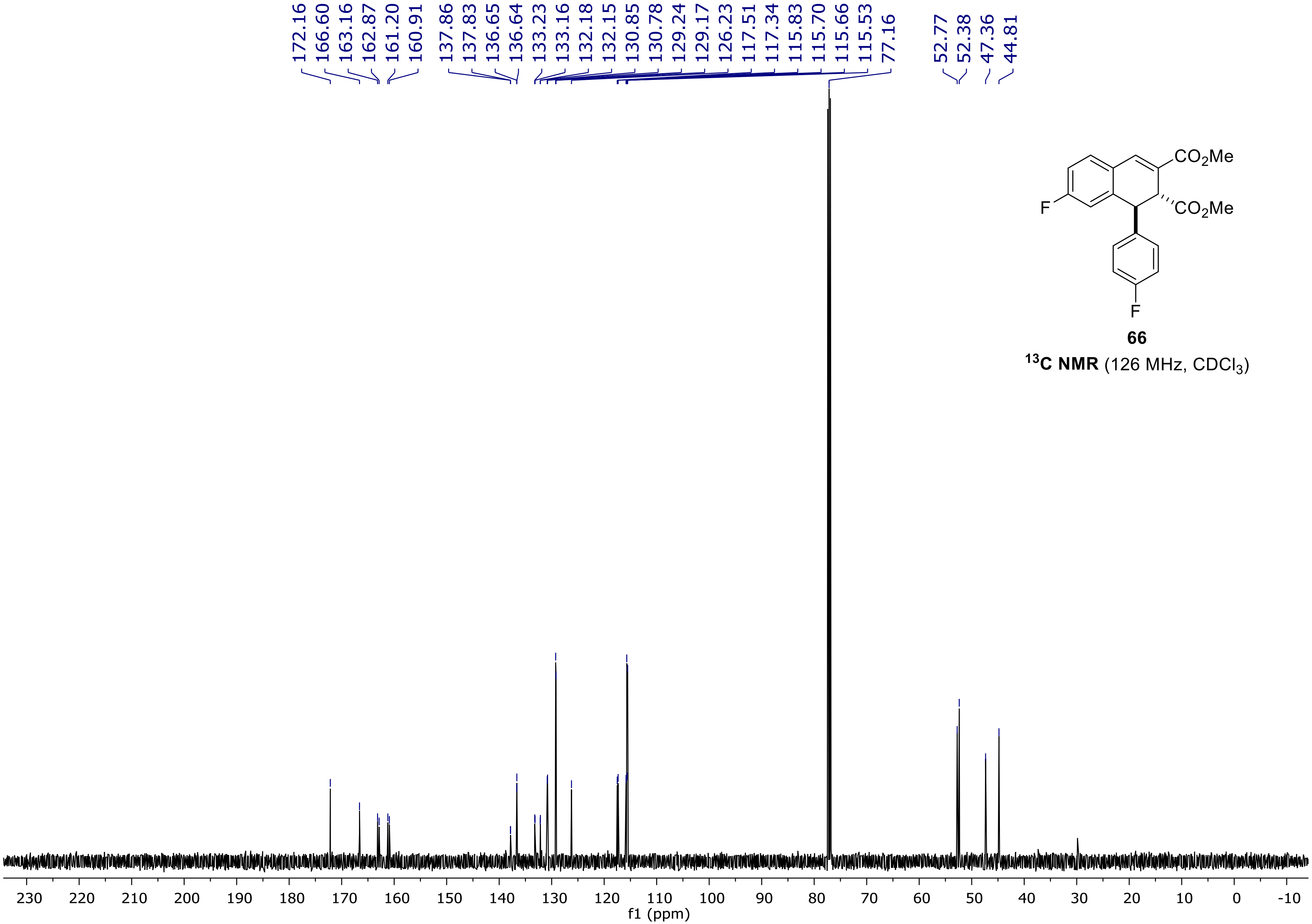

66

^{13}C NMR (126 MHz, $CDCl_3$)

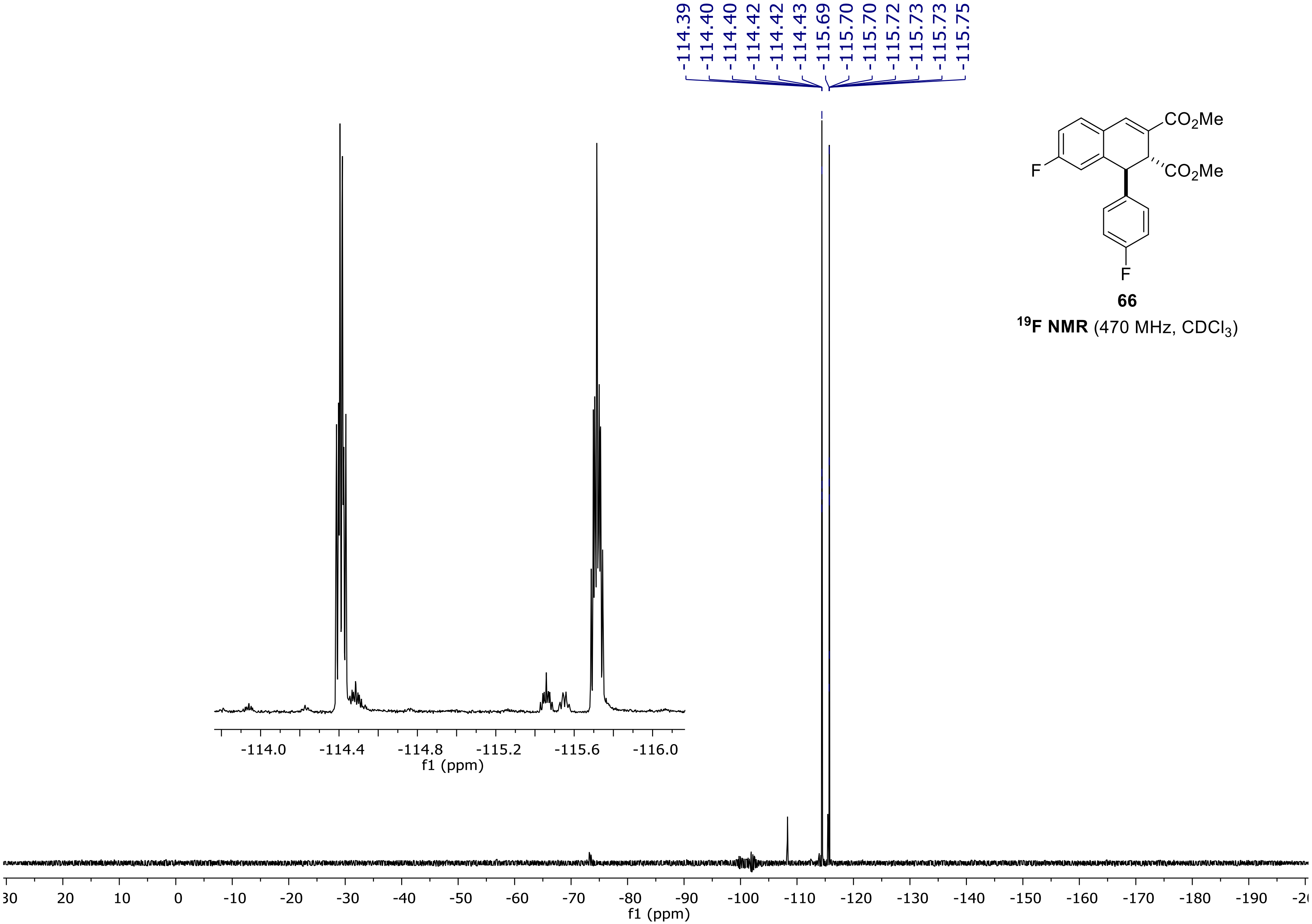

66

^{19}F NMR (470 MHz, $CDCl_3$)

Alfonzo 150

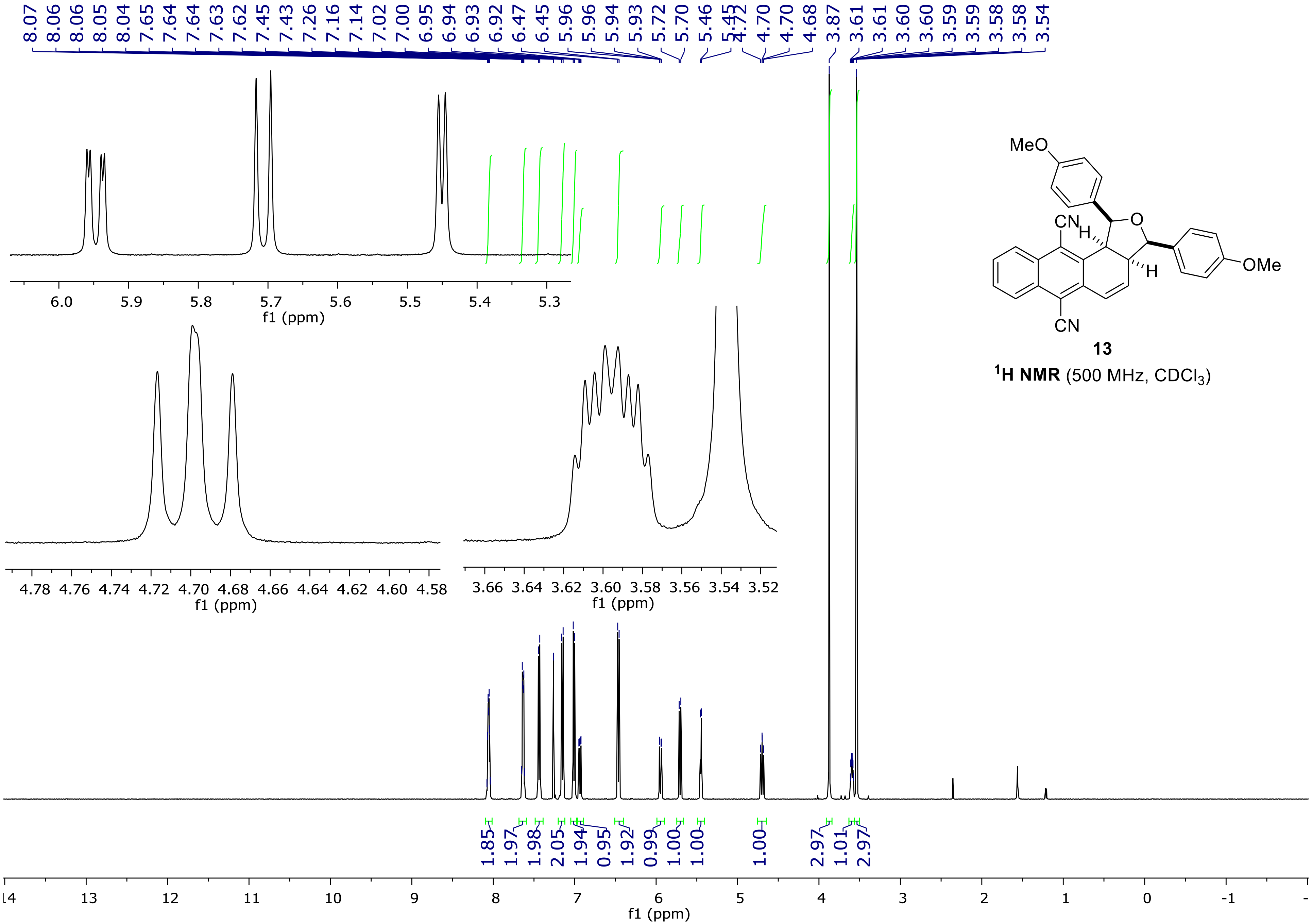

8.07 8.06 8.06 8.05 8.04 7.65 7.64 7.64 7.63 7.62 7.45 7.43 7.26 7.16 7.14 7.02 7.00 6.95 6.94 6.93 6.92 6.47 6.45 5.96 5.96 5.94 5.93 5.72 5.70 5.46 5.45 4.72 4.70 4.70 4.68 3.87 3.61 3.61 3.60 3.60 3.59 3.59 3.58 3.58 3.54
MeO
CN
H
O
H
OMe
CN
13
^{1}H NMR (500 MHz, $CDCl_3$)
1.85 1.97 1.98 2.05 1.94 0.95 1.92 0.99 1.00 1.00 1.00 2.97 1.01 2.97
f1 (ppm)

Alfonzo 151

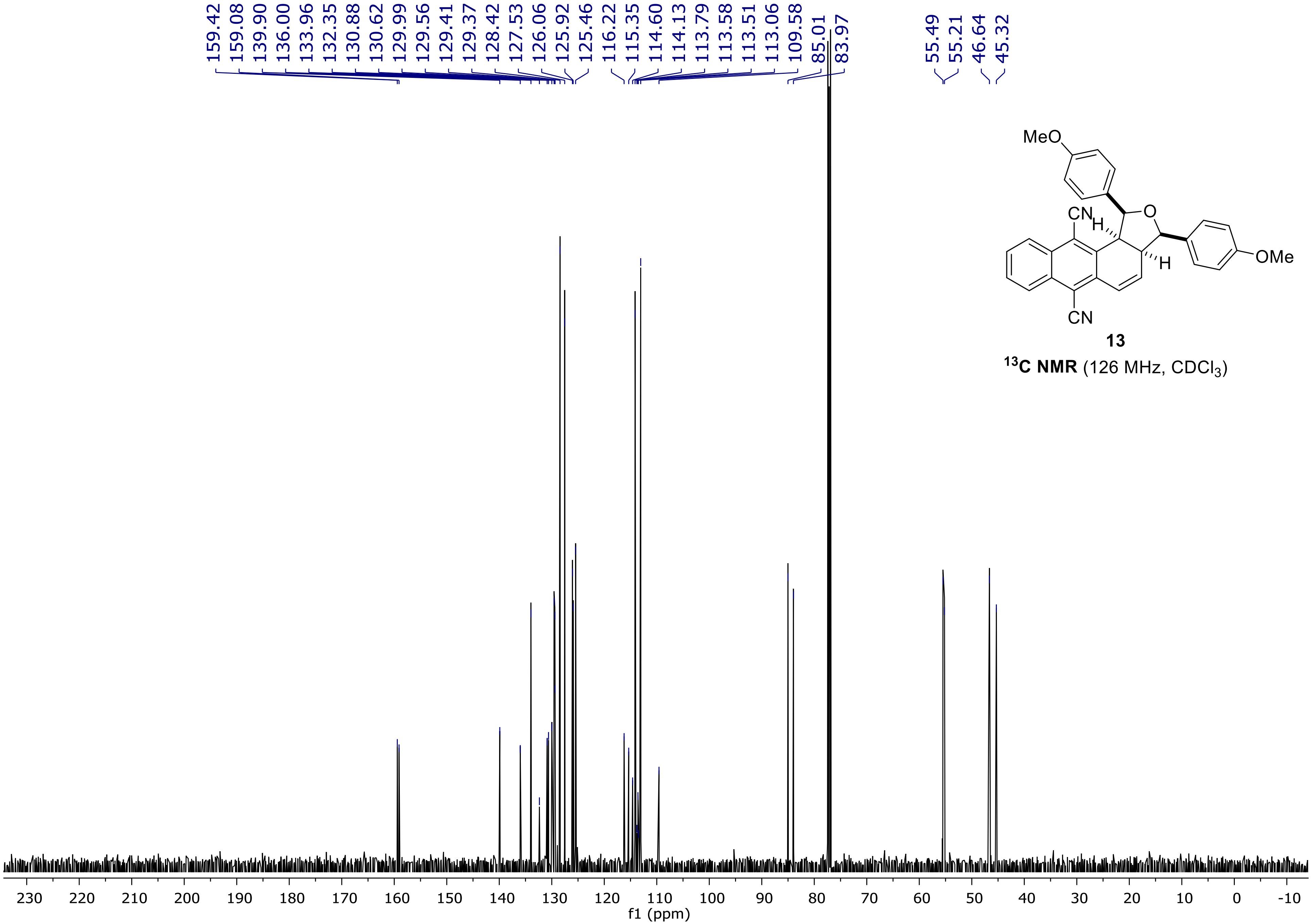

13

^{13}**C NMR** (126 MHz, $CDCl_3$)

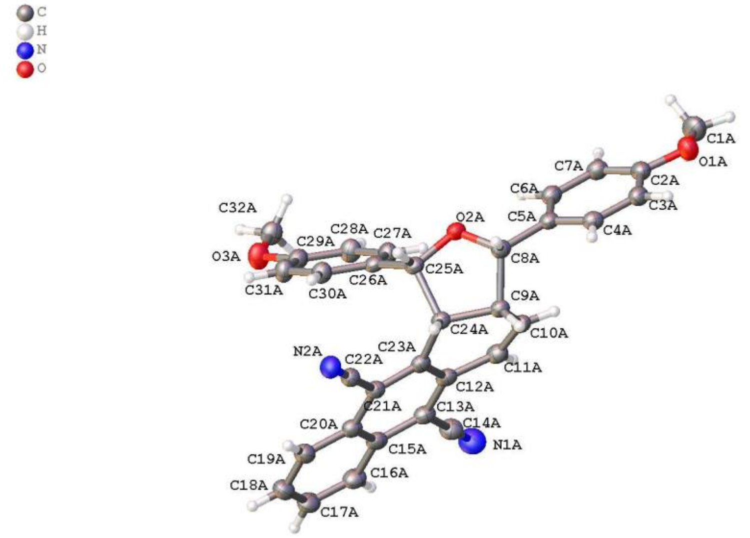

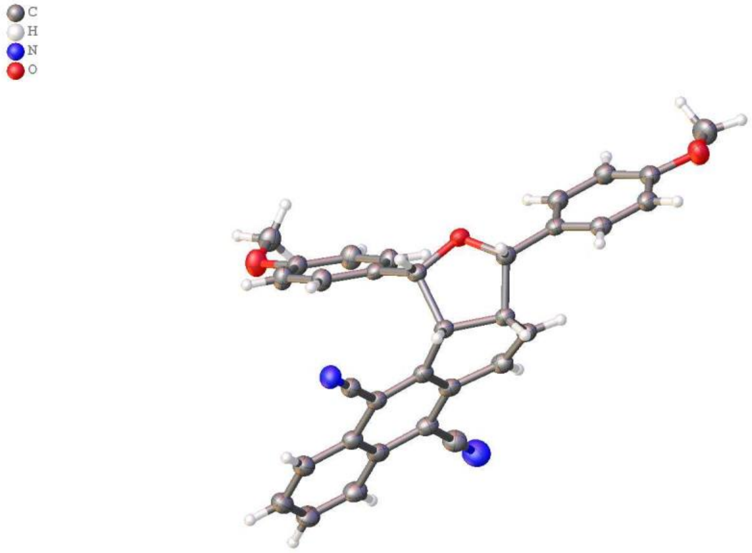

Computing details

Data collection: *APEX3* (Bruker, 2016); cell refinement: *APEX3* (Bruker, 2016); data reduction: *SAINT* (Bruker, 2016); program(s) used to solve structure: ShelXT (Sheldrick, 2015); program(s) used to refine structure: *SHELXL* (Sheldrick, 2015); molecular graphics: Olex2 (Dolomanov *et al.*, 2009); software used to prepare material for publication: Olex2 (Dolomanov *et al.*, 2009).

References

Sheldrick, G. M. (2008). *A Brief History of SHELX*. Acta Cryst. A**64**, 112-122.

Sheldrick, G. M. (2015). *Crystal Structure Refinement with SHELXL*. Acta Cryst. C**71**, 3-8.

Bruker (2006). *SAINT*. Bruker Analytical X-ray Instruments Inc., Madison, Wisconsin, USA.

Bruker (2016). *APEX3*. Bruker Analytical X-ray Instruments Inc., Madison, Wisconsin, USA.

Krause, L., *et al.* (2015). *Comparison of Silver and Molybdenum Microfocus X-ray Sources for Single-Crystal Structure Determination*. J. Appl. Cryst. **48**, 3-10 (2015).

Dolmanov, O. V., *et al.* (2009). *OLEX2: A complete structure solution, refinement and analysis program*. J. Appl. Cryst. **42**, 339-341.

(twin4_transformed)

Crystal data

$C_{32}H_{24}N_2O_3$	*F*(000) = 2032
M_r = 484.53	D_x = 1.303 Mg m^{-3}
Monoclinic, $P2_1/c$	Cu *K*□ radiation, □ = 1.54178 Å
a = 29.638 (3) Å	Cell parameters from 2513 reflections
b = 9.9154 (9) Å	□ = 5.2–65.7°
c = 17.199 (2) Å	□ = 0.67 mm^{-1}
□ = 102.137 (8)°	*T* = 100 K
V = 4941.3 (10) Å^3	Prism
Z = 8	0.34 × 0.22 × 0.12 mm

Data collection

Bruker X8 Proteum-R diffractometer	59476 independent reflections
Radiation source: rotating anode	47057 reflections with *I* > 2□(*I*)
Montel monochromator	□$_{max}$ = 66.8°, □$_{min}$ = 4.6°
□ and □ scans	*h* = -35□35
Absorption correction: multi-scan TWINABS-2012/1 (Bruker,2012) was used for absorption correction.<br>Final HKLF 4 output contains 121173 reflections, Rint = 0.1178 (63919 with I > 3sig(I), Rint = 0.1091)	*k* = -11□11
T_{min} = 0.650, T_{max} = 0.840	*l* = -20□20
59476 measured reflections	

Refinement

Refinement on F^2	Primary atom site location: dual
Least-squares matrix: full	Hydrogen site location: inferred from

	neighbouring sites
$R[F^2 > 2□(F^2)] = 0.075$	H-atom parameters constrained
$wR(F^2) = 0.220$	$w = 1/[□^2(F_o^2) + (0.1131P)^2]$<br>where $P = (F_o^2 + 2F_c^2)/3$
$S = 1.06$	$(□/□)_{max} < 0.001$
59476 reflections	$□□_{max} = 0.26$ e Å^{-3}
672 parameters	$□□_{min} = -0.35$ e Å^{-3}
0 restraints	

Special details

Geometry. All esds (except the esd in the dihedral angle between two l.s. planes) are estimated using the full covariance matrix. The cell esds are taken into account individually in the estimation of esds in distances, angles and torsion angles; correlations between esds in cell parameters are only used when they are defined by crystal symmetry. An approximate (isotropic) treatment of cell esds is used for estimating esds involving l.s. planes.
Refinement. Refined as a 2-component twin.

Fractional atomic coordinates and isotropic or equivalent isotropic displacement parameters (Å²) for (twin4_transformed)

	x	*y*	*z*	U_{iso}*/U_{eq}
O2B	0.52398 (7)	1.1070 (2)	0.31849 (13)	0.0285 (5)
O2A	0.02304 (7)	0.4084 (2)	0.20918 (13)	0.0287 (5)
O1B	0.30599 (7)	1.0697 (2)	0.20225 (16)	0.0394 (6)
O3B	0.67191 (8)	1.2591 (2)	0.62826 (15)	0.0369 (6)
O1A	-0.19533 (7)	0.4730 (2)	0.15326 (16)	0.0418 (6)
O3A	0.16140 (7)	0.2471 (3)	-0.00728 (16)	0.0387 (6)
N2A	0.18833 (9)	0.5864 (3)	0.35263 (18)	0.0349 (6)
N2B	0.69037 (9)	0.9289 (3)	0.30915 (18)	0.0371 (7)
C13B	0.59213 (10)	0.6932 (3)	0.5003 (2)	0.0284 (7)
C26A	0.09411 (10)	0.3937 (3)	0.16504 (19)	0.0267 (6)
N1A	0.05791 (11)	0.9164 (3)	-0.0545 (2)	0.0472 (8)
N1B	0.54667 (10)	0.5672 (3)	0.59119 (19)	0.0437 (7)
C8A	-0.00063 (10)	0.5000 (3)	0.25111 (18)	0.0276 (7)
H8A	0.007290	0.477833	0.307885	0.033*
C5B	0.44898 (10)	1.0369 (3)	0.2451 (2)	0.0281 (7)
C31B	0.64125 (10)	1.1720 (3)	0.41948 (19)	0.0295 (7)
H31B	0.654855	1.177033	0.375517	0.035*
C23A	0.09863 (10)	0.6787 (3)	0.19713 (19)	0.0268 (7)

C26B	0.59696 (10)	1.1193 (3)	0.41042 (19)	0.0266 (7)
C3B	0.37300 (10)	0.9986 (3)	0.1638 (2)	0.0312 (7)
H3B	0.354368	0.964263	0.117769	0.037*
C22A	0.16967 (10)	0.6326 (3)	0.2926 (2)	0.0291 (7)
C20B	0.66702 (10)	0.7443 (3)	0.4701 (2)	0.0288 (7)
C21A	0.14609 (10)	0.6891 (3)	0.21832 (19)	0.0272 (7)
C9A	0.02101 (9)	0.6380 (3)	0.23840 (19)	0.0261 (6)
H9A	0.017205	0.700676	0.280604	0.031*
C4A	-0.08081 (10)	0.5358 (3)	0.2707 (2)	0.0294 (7)
H4A	-0.068032	0.573359	0.319962	0.035*
C9B	0.52010 (10)	0.8797 (3)	0.28160 (19)	0.0271 (7)
H9B	0.516795	0.820969	0.234922	0.033*
C10A	0.00076 (10)	0.6971 (3)	0.1592 (2)	0.0283 (7)
H10A	-0.031201	0.704610	0.145351	0.034*
C30B	0.66542 (10)	1.2169 (3)	0.4923 (2)	0.0308 (7)
H30B	0.695044	1.251472	0.496986	0.037*
C12B	0.56933 (10)	0.7653 (3)	0.4346 (2)	0.0273 (7)
C21B	0.64295 (10)	0.8180 (3)	0.4031 (2)	0.0282 (7)
C2B	0.35319 (10)	1.0619 (3)	0.2210 (2)	0.0316 (7)
C15B	0.64138 (10)	0.6804 (3)	0.5203 (2)	0.0296 (7)
C11B	0.51898 (10)	0.7682 (3)	0.4119 (2)	0.0298 (7)
H11B	0.501752	0.734951	0.447011	0.036*
C23B	0.59540 (10)	0.8306 (3)	0.38447 (19)	0.0275 (7)
C8B	0.50042 (10)	1.0209 (3)	0.25647 (19)	0.0278 (7)
H8B	0.509543	1.046045	0.206846	0.033*
C15A	0.14970 (10)	0.8099 (3)	0.0954 (2)	0.0304 (7)
C5A	-0.05193 (10)	0.4872 (3)	0.2227 (2)	0.0273 (7)
C25A	0.07075 (10)	0.4422 (3)	0.22971 (19)	0.0265 (6)
H25A	0.084503	0.394665	0.278940	0.032*
C24A	0.07254 (10)	0.6000 (3)	0.24793 (19)	0.0253 (6)
H24A	0.087793	0.613159	0.303742	0.030*
C13A	0.10049 (10)	0.8022 (3)	0.0759 (2)	0.0301 (7)
C30A	0.16235 (10)	0.3096 (3)	0.1248 (2)	0.0337 (7)
H30A	0.193448	0.286509	0.137676	0.040*
C29A	0.13784 (10)	0.2976 (3)	0.0469 (2)	0.0307 (7)
C31A	0.14050 (10)	0.3558 (3)	0.1831 (2)	0.0314 (7)
H31A	0.156998	0.361821	0.235291	0.038*

C7B	0.38089 (10)	1.1109 (3)	0.2903 (2)	0.0323 (7)
H7B	0.367733	1.152243	0.328693	0.039*
C24B	0.57176 (9)	0.9131 (3)	0.31409 (19)	0.0258 (6)
H24B	0.588181	0.898166	0.270983	0.031*
C12A	0.07510 (10)	0.7392 (3)	0.1249 (2)	0.0278 (7)
C6B	0.42872 (10)	1.0978 (3)	0.3019 (2)	0.0297 (7)
H6B	0.447335	1.130381	0.348443	0.036*
C25B	0.57153 (9)	1.0705 (3)	0.33057 (19)	0.0262 (6)
H25B	0.584661	1.115947	0.289823	0.031*
C29B	0.64564 (10)	1.2107 (3)	0.5588 (2)	0.0302 (7)
C22B	0.66949 (10)	0.8800 (3)	0.3508 (2)	0.0305 (7)
C20A	0.17296 (10)	0.7532 (3)	0.1684 (2)	0.0293 (7)
C4B	0.42042 (10)	0.9873 (3)	0.1760 (2)	0.0298 (7)
H4B	0.433533	0.945982	0.137571	0.036*
C27B	0.57776 (10)	1.1119 (3)	0.4776 (2)	0.0315 (7)
H27B	0.548369	1.075816	0.473107	0.038*
C28B	0.60163 (11)	1.1575 (3)	0.5511 (2)	0.0344 (7)
H28B	0.588142	1.152241	0.595185	0.041*
C11A	0.02467 (10)	0.7401 (3)	0.1067 (2)	0.0301 (7)
H11A	0.008990	0.771451	0.057438	0.036*
C3A	-0.12827 (10)	0.5289 (3)	0.2458 (2)	0.0326 (7)
H3A	-0.147134	0.561961	0.278349	0.039*
C6A	-0.07191 (10)	0.4303 (3)	0.1499 (2)	0.0302 (7)
H6A	-0.052986	0.396849	0.117536	0.036*
C2A	-0.14791 (10)	0.4728 (3)	0.1725 (2)	0.0332 (7)
C10B	0.49718 (10)	0.8175 (3)	0.3419 (2)	0.0290 (7)
H10B	0.465130	0.812601	0.329825	0.035*
C19A	0.22174 (10)	0.7595 (3)	0.1898 (2)	0.0343 (8)
H19A	0.237393	0.722413	0.237557	0.041*
C7A	-0.11990 (11)	0.4222 (3)	0.1238 (2)	0.0333 (7)
H7A	-0.132801	0.383711	0.074888	0.040*
C32A	0.13408 (11)	0.2079 (3)	-0.0823 (2)	0.0360 (8)
H32D	0.153356	0.164787	-0.113307	0.054*
H32E	0.119937	0.286194	-0.109943	0.054*
H32F	0.110557	0.146150	-0.074098	0.054*
C14A	0.07660 (11)	0.8653 (3)	0.0036 (2)	0.0346 (7)
C32B	0.64840 (12)	1.2844 (3)	0.6915 (2)	0.0387 (8)

H32A	0.621295	1.337422	0.671793	0.058*
H32B	0.668488	1.332570	0.733378	0.058*
H32C	0.639641	1.200261	0.711607	0.058*
C27A	0.07053 (10)	0.3842 (3)	0.0870 (2)	0.0321 (7)
H27A	0.039755	0.410567	0.073661	0.039*
C16B	0.66448 (11)	0.6039 (3)	0.5859 (2)	0.0343 (7)
H16B	0.647658	0.562612	0.619247	0.041*
C19B	0.71570 (10)	0.7292 (3)	0.4878 (2)	0.0333 (7)
H19B	0.733262	0.770958	0.455859	0.040*
C28A	0.09171 (10)	0.3365 (3)	0.0283 (2)	0.0348 (7)
H28A	0.075117	0.330374	-0.023878	0.042*
C14B	0.56618 (11)	0.6239 (3)	0.5501 (2)	0.0324 (7)
C16A	0.17542 (11)	0.8732 (3)	0.0456 (2)	0.0358 (8)
H16A	0.160440	0.911138	-0.002370	0.043*
C18B	0.73697 (11)	0.6536 (3)	0.5515 (2)	0.0383 (8)
H18B	0.768897	0.644111	0.562257	0.046*
C17B	0.71132 (11)	0.5903 (4)	0.6009 (2)	0.0393 (8)
H17B	0.726248	0.538819	0.643831	0.047*
C17A	0.22259 (11)	0.8786 (3)	0.0679 (2)	0.0393 (8)
H17A	0.239436	0.921249	0.035143	0.047*
C18A	0.24571 (11)	0.8208 (3)	0.1394 (2)	0.0382 (8)
H18A	0.277761	0.824039	0.153056	0.046*
C1B	0.28325 (12)	1.1078 (4)	0.2646 (3)	0.0489 (10)
H1BA	0.250661	1.092772	0.247691	0.073*
H1BB	0.288829	1.201621	0.276865	0.073*
H1BC	0.295007	1.054708	0.311139	0.073*
C1A	-0.21684 (12)	0.4336 (4)	0.0744 (3)	0.0487 (10)
H1AA	-0.205349	0.488501	0.036883	0.073*
H1AB	-0.249629	0.445205	0.066882	0.073*
H1AC	-0.210011	0.340625	0.066476	0.073*

Atomic displacement parameters (Å^2) for (twin4_transformed)

	U^{11}	U^{22}	U^{33}	U^{12}	U^{13}	U^{23}
O2B	0.0292 (10)	0.0214 (10)	0.0337 (13)	0.0010 (8)	0.0039 (9)	-0.0022 (9)
O2A	0.0294 (10)	0.0213 (10)	0.0367 (13)	-0.0004 (8)	0.0098 (9)	-0.0023 (9)
O1B	0.0294 (11)	0.0377 (13)	0.0508 (16)	-0.0019 (9)	0.0077 (11)	-0.0041 (11)

O3B	0.0419 (12)	0.0380 (13)	0.0295 (14)	-0.0029 (10)	0.0048 (11)	-0.0048 (10)
O1A	0.0289 (11)	0.0428 (14)	0.0527 (16)	-0.0010 (9)	0.0063 (11)	-0.0069 (12)
O3A	0.0359 (12)	0.0481 (13)	0.0324 (15)	0.0054 (10)	0.0082 (11)	-0.0096 (10)
N2A	0.0328 (13)	0.0353 (15)	0.0360 (17)	0.0037 (11)	0.0056 (13)	0.0058 (13)
N2B	0.0383 (14)	0.0334 (16)	0.0426 (18)	0.0015 (11)	0.0149 (14)	0.0061 (13)
C13B	0.0341 (15)	0.0226 (15)	0.0295 (17)	-0.0012 (12)	0.0087 (13)	-0.0016 (13)
C26A	0.0303 (14)	0.0189 (14)	0.0306 (17)	0.0001 (11)	0.0058 (13)	0.0022 (13)
N1A	0.0515 (17)	0.053 (2)	0.0370 (19)	-0.0014 (14)	0.0079 (15)	0.0101 (16)
N1B	0.0497 (17)	0.0460 (18)	0.0378 (18)	-0.0028 (14)	0.0150 (14)	0.0089 (14)
C8A	0.0314 (15)	0.0242 (15)	0.0287 (18)	0.0005 (11)	0.0100 (14)	-0.0005 (14)
C5B	0.0333 (15)	0.0215 (15)	0.0295 (17)	0.0012 (11)	0.0061 (13)	0.0043 (13)
C31B	0.0341 (15)	0.0268 (16)	0.0292 (17)	-0.0004 (12)	0.0100 (13)	0.0017 (14)
C23A	0.0309 (15)	0.0217 (15)	0.0275 (17)	-0.0002 (11)	0.0057 (13)	-0.0034 (13)
C26B	0.0310 (15)	0.0168 (14)	0.0323 (18)	0.0022 (11)	0.0073 (13)	0.0018 (13)
C3B	0.0357 (16)	0.0229 (16)	0.0341 (18)	-0.0033 (12)	0.0048 (14)	0.0010 (13)
C22A	0.0271 (14)	0.0265 (16)	0.0344 (19)	-0.0004 (12)	0.0078 (14)	0.0003 (14)
C20B	0.0336 (15)	0.0209 (14)	0.0328 (19)	0.0013 (12)	0.0091 (14)	-0.0029 (13)
C21A	0.0300 (15)	0.0227 (15)	0.0289 (17)	-0.0003 (11)	0.0065 (13)	0.0001 (13)
C9A	0.0289 (14)	0.0215 (15)	0.0275 (17)	-0.0004 (11)	0.0053 (12)	-0.0010 (12)
C4A	0.0332 (16)	0.0260 (16)	0.0293 (17)	-0.0016 (12)	0.0072 (13)	-0.0006 (13)
C9B	0.0305 (15)	0.0227 (15)	0.0280 (17)	0.0010 (11)	0.0055 (13)	-0.0029 (13)
C10A	0.0269 (14)	0.0218 (15)	0.0343 (18)	0.0004 (11)	0.0019 (13)	0.0004 (13)
C30B	0.0283 (15)	0.0292 (17)	0.035 (2)	-0.0022 (12)	0.0063 (14)	-0.0004 (14)
C12B	0.0324 (15)	0.0214 (15)	0.0286 (18)	0.0016 (11)	0.0076 (13)	-0.0024 (13)
C21B	0.0327 (15)	0.0209 (15)	0.0322 (18)	0.0015 (11)	0.0098 (13)	-0.0001 (13)

C2B	0.0300 (15)	0.0254 (16)	0.0390 (19)	-0.0017 (12)	0.0064 (14)	0.0040 (14)
C15B	0.0354 (16)	0.0221 (15)	0.0316 (18)	0.0015 (12)	0.0076 (14)	-0.0013 (13)
C11B	0.0325 (15)	0.0229 (16)	0.036 (2)	-0.0008 (12)	0.0131 (14)	0.0008 (13)
C23B	0.0328 (15)	0.0197 (15)	0.0313 (18)	0.0019 (11)	0.0097 (13)	-0.0025 (13)
C8B	0.0316 (15)	0.0229 (16)	0.0286 (18)	-0.0003 (11)	0.0059 (13)	0.0012 (13)
C15A	0.0369 (16)	0.0228 (15)	0.0334 (19)	-0.0029 (12)	0.0113 (14)	-0.0032 (14)
C5A	0.0287 (15)	0.0207 (15)	0.0328 (18)	-0.0002 (11)	0.0070 (13)	0.0023 (13)
C25A	0.0279 (14)	0.0216 (15)	0.0292 (17)	0.0002 (11)	0.0044 (12)	0.0023 (13)
C24A	0.0277 (14)	0.0223 (15)	0.0255 (16)	-0.0003 (11)	0.0049 (12)	-0.0004 (12)
C13A	0.0377 (16)	0.0242 (15)	0.0288 (18)	-0.0006 (12)	0.0079 (14)	0.0003 (14)
C30A	0.0293 (15)	0.0358 (17)	0.0347 (19)	0.0047 (13)	0.0041 (14)	-0.0042 (15)
C29A	0.0327 (15)	0.0274 (16)	0.0334 (19)	0.0018 (12)	0.0103 (14)	-0.0013 (14)
C31A	0.0310 (15)	0.0322 (17)	0.0297 (18)	0.0021 (12)	0.0035 (13)	-0.0015 (14)
C7B	0.0351 (16)	0.0274 (16)	0.0360 (19)	0.0011 (12)	0.0107 (14)	-0.0013 (14)
C24B	0.0284 (14)	0.0220 (15)	0.0286 (17)	0.0001 (11)	0.0091 (13)	-0.0007 (13)
C12A	0.0344 (15)	0.0214 (14)	0.0273 (18)	-0.0018 (12)	0.0056 (14)	-0.0022 (12)
C6B	0.0333 (15)	0.0259 (15)	0.0289 (18)	-0.0013 (12)	0.0043 (13)	-0.0009 (13)
C25B	0.0294 (14)	0.0212 (15)	0.0290 (17)	0.0010 (11)	0.0084 (13)	0.0037 (13)
C29B	0.0350 (16)	0.0250 (16)	0.0299 (18)	0.0015 (12)	0.0052 (14)	-0.0002 (14)
C22B	0.0296 (15)	0.0250 (16)	0.0365 (19)	0.0033 (12)	0.0062 (14)	0.0000 (14)
C20A	0.0334 (16)	0.0235 (15)	0.0319 (19)	-0.0012 (12)	0.0087 (14)	-0.0022 (12)
C4B	0.0370 (16)	0.0216 (15)	0.0313 (18)	0.0012 (12)	0.0081 (14)	0.0012 (13)
C27B	0.0316 (15)	0.0285 (16)	0.0362 (19)	-0.0042 (12)	0.0111 (14)	-0.0011 (14)
C28B	0.0411 (17)	0.0335 (18)	0.0310 (19)	-0.0017 (13)	0.0129 (14)	-0.0004 (15)

C11A	0.0333 (16)	0.0265 (15)	0.0278 (18)	0.0004 (12)	0.0003 (14)	0.0015 (13)
C3A	0.0325 (16)	0.0274 (16)	0.041 (2)	-0.0003 (12)	0.0137 (14)	-0.0006 (15)
C6A	0.0328 (15)	0.0264 (16)	0.0325 (18)	0.0013 (12)	0.0093 (14)	-0.0003 (14)
C2A	0.0285 (15)	0.0268 (16)	0.044 (2)	-0.0005 (12)	0.0058 (14)	0.0015 (15)
C10B	0.0280 (14)	0.0207 (15)	0.0387 (19)	-0.0003 (11)	0.0077 (13)	-0.0006 (13)
C19A	0.0328 (16)	0.0304 (16)	0.041 (2)	-0.0024 (13)	0.0103 (15)	-0.0001 (14)
C7A	0.0355 (16)	0.0270 (17)	0.036 (2)	-0.0010 (12)	0.0054 (14)	-0.0024 (14)
C32A	0.0383 (17)	0.0374 (19)	0.032 (2)	0.0038 (14)	0.0054 (15)	-0.0051 (16)
C14A	0.0394 (17)	0.0354 (18)	0.0297 (19)	-0.0031 (14)	0.0089 (15)	0.0033 (15)
C32B	0.055 (2)	0.0322 (19)	0.030 (2)	-0.0030 (14)	0.0116 (16)	0.0001 (15)
C27A	0.0277 (15)	0.0339 (17)	0.0337 (19)	0.0040 (12)	0.0040 (13)	-0.0002 (14)
C16B	0.0401 (17)	0.0289 (17)	0.0336 (19)	0.0023 (13)	0.0073 (15)	0.0052 (14)
C19B	0.0335 (16)	0.0291 (17)	0.038 (2)	0.0014 (12)	0.0088 (15)	0.0015 (14)
C28A	0.0331 (16)	0.0390 (19)	0.0301 (18)	0.0028 (13)	0.0014 (14)	-0.0045 (15)
C14B	0.0372 (16)	0.0285 (16)	0.0319 (18)	0.0011 (13)	0.0081 (14)	0.0014 (14)
C16A	0.0442 (18)	0.0293 (17)	0.037 (2)	-0.0009 (13)	0.0148 (15)	0.0042 (15)
C18B	0.0334 (16)	0.0375 (19)	0.042 (2)	0.0050 (13)	0.0040 (15)	0.0020 (16)
C17B	0.0408 (18)	0.0351 (18)	0.040 (2)	0.0077 (14)	0.0034 (15)	0.0080 (16)
C17A	0.0425 (18)	0.0331 (18)	0.048 (2)	-0.0027 (14)	0.0218 (16)	0.0052 (16)
C18A	0.0332 (16)	0.0343 (18)	0.050 (2)	-0.0049 (13)	0.0143 (16)	-0.0016 (16)
C1B	0.0365 (18)	0.050 (2)	0.064 (3)	0.0020 (15)	0.0185 (18)	-0.001 (2)
C1A	0.0344 (17)	0.047 (2)	0.058 (3)	-0.0011 (15)	-0.0044 (17)	-0.0069 (19)

Geometric parameters (Å, º) for (twin4_transformed)

O2B—C8B	1.428 (4)	C9A—C24A	1.548 (4)
O2B—C25B	1.427 (3)	C4A—C5A	1.395 (4)
O2A—C8A	1.432 (4)	C4A—C3A	1.383 (4)

O2A—C25A	1.424 (3)	C9B—C8B	1.543 (4)
O1B—C2B	1.370 (4)	C9B—C24B	1.551 (4)
O1B—C1B	1.433 (4)	C9B—C10B	1.488 (4)
O3B—C29B	1.369 (4)	C10A—C11A	1.330 (5)
O3B—C32B	1.431 (4)	C30B—C29B	1.391 (5)
O1A—C2A	1.374 (4)	C12B—C11B	1.461 (4)
O1A—C1A	1.426 (5)	C12B—C23B	1.428 (4)
O3A—C29A	1.371 (4)	C21B—C23B	1.384 (4)
O3A—C32A	1.425 (4)	C21B—C22B	1.450 (4)
N2A—C22A	1.159 (4)	C2B—C7B	1.384 (5)
N2B—C22B	1.147 (4)	C15B—C16B	1.411 (5)
C13B—C12B	1.387 (5)	C11B—C10B	1.333 (5)
C13B—C15B	1.433 (4)	C23B—C24B	1.506 (4)
C13B—C14B	1.441 (4)	C15A—C13A	1.428 (4)
C26A—C25A	1.507 (4)	C15A—C20A	1.415 (5)
C26A—C31A	1.396 (4)	C15A—C16A	1.409 (4)
C26A—C27A	1.380 (5)	C5A—C6A	1.387 (5)
N1A—C14A	1.153 (4)	C25A—C24A	1.594 (4)
N1B—C14B	1.149 (4)	C13A—C12A	1.390 (4)
C8A—C9A	1.546 (4)	C13A—C14A	1.439 (5)
C8A—C5A	1.501 (4)	C30A—C29A	1.388 (5)
C5B—C8B	1.504 (4)	C30A—C31A	1.381 (4)
C5B—C6B	1.387 (4)	C29A—C28A	1.392 (4)
C5B—C4B	1.395 (5)	C7B—C6B	1.396 (4)
C31B—C26B	1.391 (4)	C24B—C25B	1.587 (4)
C31B—C30B	1.380 (5)	C12A—C11A	1.461 (4)
C23A—C21A	1.381 (4)	C29B—C28B	1.388 (4)
C23A—C24A	1.501 (4)	C20A—C19A	1.416 (4)
C23A—C12A	1.422 (5)	C27B—C28B	1.388 (5)
C26B—C25B	1.501 (4)	C3A—C2A	1.388 (5)
C26B—C27B	1.393 (4)	C6A—C7A	1.401 (4)
C3B—C2B	1.396 (5)	C2A—C7A	1.391 (5)
C3B—C4B	1.381 (4)	C19A—C18A	1.372 (5)
C22A—C21A	1.435 (5)	C27A—C28A	1.379 (5)
C20B—C21B	1.424 (5)	C16B—C17B	1.364 (5)
C20B—C15B	1.414 (4)	C19B—C18B	1.367 (5)
C20B—C19B	1.418 (4)	C16A—C17A	1.371 (5)

C21A—C20A	1.437 (4)	C18B—C17B	1.401 (5)
C9A—C10A	1.488 (4)	C17A—C18A	1.398 (5)
C25B—O2B—C8B	105.7 (2)	C20A—C15A—C13A	118.0 (3)
C25A—O2A—C8A	107.0 (2)	C16A—C15A—C13A	122.5 (3)
C2B—O1B—C1B	117.1 (3)	C16A—C15A—C20A	119.4 (3)
C29B—O3B—C32B	116.5 (2)	C4A—C5A—C8A	118.9 (3)
C2A—O1A—C1A	117.3 (3)	C6A—C5A—C8A	122.6 (3)
C29A—O3A—C32A	116.2 (2)	C6A—C5A—C4A	118.4 (3)
C12B—C13B—C15B	122.6 (3)	O2A—C25A—C26A	109.2 (2)
C12B—C13B—C14B	120.1 (3)	O2A—C25A—C24A	105.7 (2)
C15B—C13B—C14B	117.3 (3)	C26A—C25A—C24A	117.2 (2)
C31A—C26A—C25A	120.4 (3)	C23A—C24A—C9A	115.4 (2)
C27A—C26A—C25A	121.5 (3)	C23A—C24A—C25A	113.3 (2)
C27A—C26A—C31A	118.1 (3)	C9A—C24A—C25A	103.1 (2)
O2A—C8A—C9A	103.2 (2)	C15A—C13A—C14A	118.2 (3)
O2A—C8A—C5A	110.6 (2)	C12A—C13A—C15A	122.6 (3)
C5A—C8A—C9A	116.8 (2)	C12A—C13A—C14A	119.2 (3)
C6B—C5B—C8B	122.1 (3)	C31A—C30A—C29A	119.9 (3)
C6B—C5B—C4B	118.5 (3)	O3A—C29A—C30A	116.7 (3)
C4B—C5B—C8B	119.4 (3)	O3A—C29A—C28A	124.1 (3)
C30B—C31B—C26B	121.4 (3)	C30A—C29A—C28A	119.2 (3)
C21A—C23A—C24A	120.4 (3)	C30A—C31A—C26A	121.2 (3)
C21A—C23A—C12A	119.0 (3)	C2B—C7B—C6B	119.4 (3)
C12A—C23A—C24A	120.6 (3)	C9B—C24B—C25B	103.5 (2)
C31B—C26B—	120.3 (3)	C23B—C24B—C9B	115.5 (2)

C25B			
C31B—C26B—C27B	117.9 (3)	C23B—C24B—C25B	114.1 (2)
C27B—C26B—C25B	121.9 (3)	C23A—C12A—C11A	118.8 (3)
C4B—C3B—C2B	119.6 (3)	C13A—C12A—C23A	119.3 (3)
N2A—C22A—C21A	179.3 (3)	C13A—C12A—C11A	121.8 (3)
C15B—C20B—C21B	118.8 (3)	C5B—C6B—C7B	121.1 (3)
C15B—C20B—C19B	118.3 (3)	O2B—C25B—C26B	110.0 (2)
C19B—C20B—C21B	122.8 (3)	O2B—C25B—C24B	105.3 (2)
C23A—C21A—C22A	119.0 (3)	C26B—C25B—C24B	117.4 (2)
C23A—C21A—C20A	122.5 (3)	O3B—C29B—C30B	116.2 (3)
C22A—C21A—C20A	118.5 (3)	O3B—C29B—C28B	124.6 (3)
C8A—C9A—C24A	101.5 (2)	C28B—C29B—C30B	119.3 (3)
C10A—C9A—C8A	112.2 (2)	N2B—C22B—C21B	179.8 (4)
C10A—C9A—C24A	113.1 (2)	C15A—C20A—C21A	118.6 (3)
C3A—C4A—C5A	120.7 (3)	C15A—C20A—C19A	119.6 (3)
C8B—C9B—C24B	101.2 (2)	C19A—C20A—C21A	121.9 (3)
C10B—C9B—C8B	111.7 (2)	C3B—C4B—C5B	121.1 (3)
C10B—C9B—C24B	113.5 (3)	C28B—C27B—C26B	121.3 (3)
C11A—C10A—C9A	125.3 (3)	C29B—C28B—C27B	120.0 (3)
C31B—C30B—C29B	120.2 (3)	C10A—C11A—C12A	121.3 (3)
C13B—C12B—C11B	121.6 (3)	C4A—C3A—C2A	120.4 (3)
C13B—C12B—C23B	119.5 (3)	C5A—C6A—C7A	121.6 (3)
C23B—C12B—C11B	118.8 (3)	O1A—C2A—C3A	115.5 (3)
C20B—C21B—	118.4 (3)	O1A—C2A—C7A	124.4 (3)

C22B			
C23B—C21B—C20B	123.1 (3)	C3A—C2A—C7A	120.1 (3)
C23B—C21B—C22B	118.5 (3)	C11B—C10B—C9B	125.1 (3)
O1B—C2B—C3B	114.8 (3)	C18A—C19A—C20A	119.4 (3)
O1B—C2B—C7B	125.0 (3)	C2A—C7A—C6A	118.8 (3)
C7B—C2B—C3B	120.2 (3)	N1A—C14A—C13A	179.2 (3)
C20B—C15B—C13B	117.7 (3)	C28A—C27A—C26A	121.3 (3)
C16B—C15B—C13B	122.6 (3)	C17B—C16B—C15B	120.5 (3)
C16B—C15B—C20B	119.7 (3)	C18B—C19B—C20B	120.5 (3)
C10B—C11B—C12B	121.5 (3)	C27A—C28A—C29A	120.2 (3)
C12B—C23B—C24B	120.8 (3)	N1B—C14B—C13B	178.0 (4)
C21B—C23B—C12B	118.3 (3)	C17A—C16A—C15A	119.8 (3)
C21B—C23B—C24B	120.9 (3)	C19B—C18B—C17B	120.9 (3)
O2B—C8B—C5B	110.9 (2)	C16B—C17B—C18B	120.1 (3)
O2B—C8B—C9B	103.7 (2)	C16A—C17A—C18A	120.9 (3)
C5B—C8B—C9B	116.4 (2)	C19A—C18A—C17A	120.9 (3)

Document origin: *publCIF* [Westrip, S. P. (2010). *J. Apply. Cryst.*, **43**, 920-925].
